# Comparing biparametric to multiparametric MRI in the diagnosis of clinically significant prostate cancer in biopsy-naive men (PRIME): a prospective, international, multicentre, non-inferiority within-patient, diagnostic yield trial protocol

**DOI:** 10.1136/bmjopen-2022-070280

**Published:** 2023-04-05

**Authors:** Aqua Asif, Arjun Nathan, Alexander Ng, Pramit Khetrapal, Vinson Wai-Shun Chan, Francesco Giganti, Clare Allen, Alex Freeman, Shonit Punwani, Paula Lorgelly, Caroline S Clarke, Chris Brew-Graves, Nicola Muirhead, Mark Emberton, Ridhi Agarwal, Yemisi Takwoingi, Jonathan J Deeks, Caroline M Moore, Veeru Kasivisvanathan, PRIME Trial Group, John Wilkinson

**Affiliations:** 1 Division of Surgery and Interventional Science, University College London, London, UK; 2 Clinical Effectiveness Unit, Royal College of Surgeons of England, London, UK; 3 Department of Urology, Whipps Cross University Hospital, London, UK; 4 Department of Radiology, University College London Hospitals NHS Foundation Trust, London, UK; 5 Department of Histopathology, University College London Hospitals NHS Foundation Trust, London, UK; 6 Centre for Medical Imaging, University College London, London, UK; 7 Institute of Epidemiology and Health Care, University College London, London, UK; 8 School of Population Health, The University of Auckland, Auckland, New Zealand; 9 Research Department of Primary Care and Population Health, University College London, London, UK; 10 National Cancer Imaging Translational Accelerator, University College London, London, UK; 11 Department of Urology, University College London Hospitals NHS Foundation Trust, London, UK; 12 Test Evaluation Research Group, Institute of Applied Health Research, University of Birmingham, Birmingham, UK; 13 NIHR Birmingham Biomedical Research Centre, University Hospitals Birmingham NHS Foundation Trust, Birmingham, UK; 1 Division of Surgery and Interventional Science, University College London, London, UK; 2 UCL Medical School, University College London, London, UK; 3 National Cancer Imaging Translational Accelerator, University College London, London, UK; 4 Department of Radiology, University College London Hospitals NHS Foundation Trust, London, UK; 5 Department of Urology, University College London Hospitals NHS Foundation Trust, London, UK; 6 New York Presbyterian - Weil Cornell Medicine, New York, New York, USA; 7 Department of Radiological Sciences, Sapienza University of Rome, Rome, Italy; 8 Department of Urology, University of Helsinki and Helsinki University Hospital, Helsinki, Finland; 9 Department of Surgery, UConn Health, Farmington, Connecticut, USA; 10 Department of Urology, Mayo Clinic, Rochester, Minnesota, USA; 11 Department of Radiology, Mayo Clinic, Rochester, Minnesota, USA; 12 Department of Radiology, University of Cambridge, Cambridge, UK; 13 Department of Radiology, Sorbonne Université, Assistance Publique-Hôpitaux de Paris, Hôpital Pitié-Salpêtrière, Paris, France; 14 Department of Radiology, Herlev Gentofte University Hospital, Herlev, Denmark; 15 Department of Urological Research, Herlev Gentofte University Hospital, Herlev, Denmark; 16 Joint Department of Medical Imaging, University Medical Imaging Toronto (UMIT), University of Toronto, Toronto, Ontario, Canada; 17 Martini-Klinik, Prostate Cancer Center Hamburg-Eppendorf, Hamburg, Germany; 18 Urology Department, Reina Sofía University Hospital, Maimonides Institute of Biomedical Research of Cordoba (IMIBIC), University of Cordoba (UCO), Cordoba, Spain; 19 Department of Surgery, Monash University, Melbourne, Victoria, Australia; 20 Department of Histopathology, University College London Hospital NHS Foundation Trust, London, UK; 21 Department of Primary Care and Population Health, University College London, London, UK; 22 Department of Urology, Skåne University Hospital, Malmö, Sweden; 23 EAU Research Foundation, Arnhem, The Netherlands

**Keywords:** Prostate disease, Magnetic resonance imaging, RADIOLOGY & IMAGING, Protocols & guidelines, Urological tumours

## Abstract

**Introduction:**

Prostate MRI is a well-established tool for the diagnostic work-up for men with suspected prostate cancer (PCa). Current recommendations advocate the use of multiparametric MRI (mpMRI), which is composed of three sequences: T2-weighted sequence (T2W), diffusion-weighted sequence (DWI) and dynamic contrast-enhanced sequence (DCE). Prior studies suggest that a biparametric MRI (bpMRI) approach, omitting the DCE sequences, may not compromise clinically significant cancer detection, though there are limitations to these studies, and it is not known how this may affect treatment eligibility. A bpMRI approach will reduce scanning time, may be more cost-effective and, at a population level, will allow more men to gain access to an MRI than an mpMRI approach.

**Methods:**

Prostate Imaging Using MRI±Contrast Enhancement (PRIME) is a prospective, international, multicentre, within-patient diagnostic yield trial assessing whether bpMRI is non-inferior to mpMRI in the diagnosis of clinically significant PCa. Patients will undergo the full mpMRI scan. Radiologists will be blinded to the DCE and will initially report the MRI using only the bpMRI (T2W and DWI) sequences. They will then be unblinded to the DCE sequence and will then re-report the MRI using the mpMRI sequences (T2W, DWI and DCE). Men with suspicious lesions on either bpMRI or mpMRI will undergo prostate biopsy. The main inclusion criteria are men with suspected PCa, with a serum PSA of ≤20 ng/mL and without prior prostate biopsy. The primary outcome is the proportion of men with clinically significant PCa detected (Gleason score ≥3+4 or Gleason grade group ≥2). A sample size of at least 500 patients is required. Key secondary outcomes include the proportion of clinically insignificant PCa detected and treatment decision.

**Ethics and dissemination:**

Ethical approval was obtained from the National Research Ethics Committee West Midlands, Nottingham (21/WM/0091). Results of this trial will be disseminated through peer-reviewed publications. Participants and relevant patient support groups will be informed about the results of the trial.

**Trial registration number:**

NCT04571840.

**Strengths and limitations of this study**

- Prostate Imaging Using MRI±Contrast Enhancement (PRIME) is a pragmatic, prospective, international, multicentre trial being carried out in a range of different healthcare settings.
- Its within-patient design allows patients to act as their own control, improving the efficiency and power of the trial compared with a randomised study.
- Its within-patient design allows the impact of the dynamic contrast-enhanced sequences (DWIs) on staging decisions and treatment eligibility to be made at an individual patient level.
- PRIME will be one of the first trials to carry out quality control in the performance of sites’ DWIs prior to their involvement in the trial.
- As both biparametric and multiparametric targeted biopsies are carried out in the same patient, it is possible for the performance of one technique to influence the other.

## Introduction

This protocol was written according to SPIRIT (Standard Protocol Items: Recommendations for Interventional Trials) guidelines.^1^ MRI is widely established as the gold standard diagnostic imaging modality for detecting clinically significant prostate cancer (PCa).^2^ The landmark PRECISION (Prostate Evaluation for Clinically Important Disease: Sampling Using Image Guidance or Not?) trial established the benefit of detecting clinically significant PCa using MRI and targeting biopsies based on MRI findings.^3^ The National Prostate Cancer Audit data from England showed that only 62% of patients undergo prostate MRI before biopsy, despite level 1 evidence to support the use of MRI.^2–4^

Current recommendations for the use of MRI for detection of PCa focus on the use of multiparametric MRI (mpMRI).^2 3^ mpMRI consists of three sequences: T2-weighted sequence (T2W), diffusion-weighted sequence (DWI) and dynamic contrast-enhanced sequence (DCE). On the DCE sequences, cancer-suspicious areas can demonstrate early wash-in, enhancement and rapid wash-out of contrast.^5–8^ The DCE sequences involve administering gadolinium contrast via an intravenous cannula. Therefore, it increases scanning time and healthcare costs compared with a biparametric MRI (bpMRI) approach where only T2W and DWI are used. While gadolinium is in widespread use, literature suggests it may accumulate in the basal ganglia, though its clinical relevance is not fully understood.^9 10^ In patients who are likely to get repeated scans over their lifetime, there may be no advantage of using the additional contrast if the bpMRI option is as good as the mpMRI option.

Removing the DCE sequences from the MRI protocol has been suggested as a potential avenue to improve the cost-effectiveness of using MRI in the diagnostic pathway for PCa,^11 12^ and the reduced scanning time required may improve the number of men with suspected PCa accessing an MRI scan. Using bpMRI has demonstrated similar detection rates of PCa as mpMRI, but current evidence is limited primarily to retrospective, single-centre studies.^12 13^ The few prospective studies have not been typically robustly designed to evaluate the role of DCE in PCa detection.^13 14^

The Prostate Imaging Using MRI±Contrast Enhancement (PRIME) trial aims to assess whether bpMRI is non-inferior to mpMRI in the detection of clinically significant PCa. PRIME may redefine the standard of care diagnostic test for men with suspicion of PCa and may allow many more patients who need access to an MRI to get one.

### Objectives

The primary objective is to compare the detection of clinically significant PCa (Gleason score ≥3+4 or Gleason grade group ≥2) using bpMRI±targeted biopsy with mpMRI±targeted biopsy.

Key secondary objectives are:

- To compare the proportion of men who have clinically insignificant PCa (Gleason score 3+3 or Gleason grade group 1) detected for bpMRI versus mpMRI.
- To compare the proportion of men with non-suspicious MRIs for bpMRI versus mpMRI.
- To compare the proportion of men with indeterminately scored MRI as reported by bpMRI and mpMRI.
- To compare the proportion of men with MRIs of adequate standard for reporting for bpMRI versus mpMRI.
- To compare the diagnostic test performance for bpMRI versus mpMRI.
- To compare radiological staging for bpMRI versus mpMRI.
- To compare treatment eligibility decisions for bpMRI when compared with mpMRI.
- To compare diagnostic performance of bpMRI and mpMRI when using the Likert scoring system in comparison to the Prostate Imaging Reporting and Data System (PI-RADS) V.2.1 scoring system.
- To compare the cost effectiveness of bpMRI when compared with mpMRI.

### Trial design

The PRIME trial is designed as a prospective, multicentre, within-patient, diagnostic yield trial, assessing whether bpMRI is non-inferior to mpMRI for the diagnosis of clinically significant PCa in biopsy-naive men. A paired cohort design was chosen rather than a randomised trial design for the following reasons:

- More efficient design (sevenfold lower sample size required) with equivalent quality of evidence in the setting of a diagnostic study.
- Patients act as their own control due to the within-patient design, thus allowing us to draw conclusions regarding the value of DCE sequences on a per patient level.
- Allows for the evaluation of the impact of contrast on staging decisions and treatment eligibility decisions at an individual patient level.
- Patients get the benefit of having targeted biopsies based on the information from both bpMRI and mpMRI information, whereas with a randomised study, patients randomised to one technique will be denied of potential benefit of the other.

## Methods and analysis

### Trial setting

We expect centres that perform PCa diagnostics and management from the following countries to take part: Argentina, Australia, Belgium, Brazil, Canada, Denmark, France, Finland, Germany, Italy, the Netherlands, Singapore, Spain, UK and USA. Sites will be required to undergo a period of quality control prior to including patients to ensure minimum acceptable standards for the conduct of mpMRI, reporting and targeted biopsy.

### Eligibility criteria

Patients will be considered eligible for registration into this trial if they fulfil all of the inclusion criteria and none of the exclusion criteria (box 1).

Box 1 **Eligibility criteria**

- Inclusion criteria.

- Men at least 18 years of age referred with clinical suspicion of PCa.
- Serum prostate-specific antigen of ≤20 ng/mL.
- Fit to undergo all procedures listed in the protocol.
- Able to provide written informed consent.

- Exclusion criteria.

- Prior prostate biopsy.
- Prior treatment for PCa.
- Prior prostate MRI on a previous encounter.
- Contraindication to MRI (eg, claustrophobia, some pacemakers).
- Contraindication to prostate biopsy.
- Unfit to undergo any procedures listed in the protocol.

PCa, prostate cancer.

### Interventions

#### MRI conduct

MRI will be conducted with 1.5 or 3.0 T with pelvic-phased array coils, with or without endorectal coils. The PRECISION study quality control highlighted that the image quality of the DCE sequences was the most variable sequence across sites.^3^ Therefore, to give DCE a reasonable chance of demonstrating whether it has value, MRI scanner approval for use in the study will be made on the basis of central review of MRI images, using the Prostate Imaging Quality (PI-QUAL) scoring system.^15^ In brief, PI-QUAL is a 5-point Likert scoring system, where 1 indicates no sequences are of diagnostic quality and 5 implies that each sequence individually is of optimal diagnostic quality. The objective criteria used to determine PI-QUAL scores are derived from internationally published minimum standards for MRI conduct.^16^ If necessary, sites will be given recommendations to improve image quality and will be re-evaluated after optimisation for participation in the study.

#### Reporting of MRI

**Figure 1:**
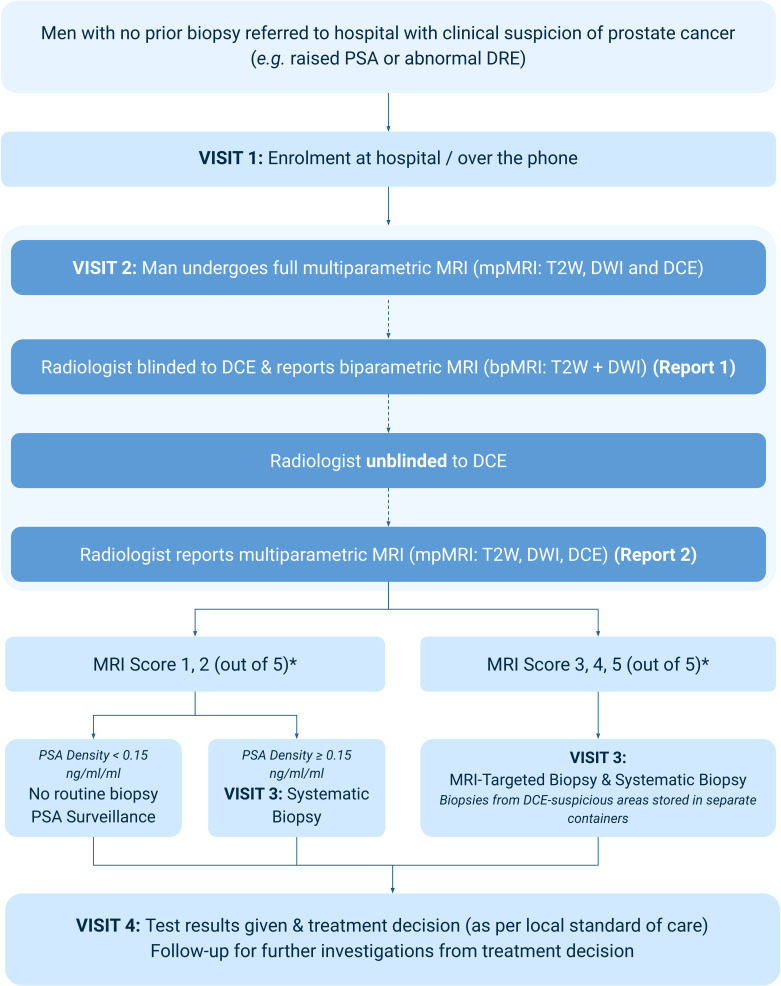
The Prostate Imaging Using MRI±Contrast Enhancement trial schema—the approach prior to MRI. *indicates a 1–5 scale of suspicion for the likelihood that clinically significant PCa is present, with 5 representing the greatest score of suspicion. For MRI to be non-suspicious it needs to be scored 1 or 2 on both Likert and PI-RADS V.2.1 systems. For MRI to be suspicious it can be scored 3, 4 or 5 on either Likert or PI-RADS V.2.1 systems. bpMRI, biparametric MRI; DCE, dynamic contrast-enhanced sequence; DRE, digital rectal examination; DWI, diffusion-weighted sequence; mpMRI, multiparametric MRI; PI-RADS, Prostate Imaging Reporting and Data System; PSA, prostate-specific antigen; T2W, T2-weighted sequence.

Patients will undergo (or will have undergone) standard of care mpMRI as per their local protocol. The radiologists participating in this trial will be blinded to the DCE sequences and will report the MRI using only the biparametric (T2W and DWI) sequences in report 1. After reporting the bpMRI, the same radiologist will be unblinded to the DCE sequences and will re-report the MRI using the mpMRI sequences (T2W, DWI and DCE) in report 2 (figure 1).

The MRIs and lesions are scored on a 1–5 scale of suspicion for the likelihood that clinically significant PCa is present, with 5 representing the greatest score of suspicion. Both the traditional Likert and PI-RADS V.2.1 scoring systems will be used to identify any suspicious lesions in the prostate. Suspicious areas (Likert or PI-RADS V.2.1 score ≥3) on either bpMRI or mpMRI will undergo targeted biopsy of the prostate, with cores from contrast-enhanced suspicious areas stored separately.

A summary of the rules for reporting MRI scans in the PRIME trial is listed in box 2. Please see online supplemental appendix 1 for our model reporting proformas, which radiologists participating in the PRIME trial will use to label lesions.

10.1136/bmjopen-2022-070280.supp1 Supplementary data

Box 2 **Summary of MRI reporting rules**

**Report 1 (bpMRI: T2W and DWI).**

1. The radiologist reporting this will be blinded to DCE, with verification of this via an independent person or an automated system (Medical Image Merge (MIM) by MIM Software Inc.).
2. The radiologist should then interpret the bpMRI sequences *blinded* to DCE.
3. Up to four suspicious areas (score ≥3 out of 5 on the Likert or PI-RADS V.2.1 scoring system) can be marked on report 1; if there are more, the four *most suspicious* should only be marked on.
4. The location of the suspicious areas should be labelled according to the PI-RADS V.2.1 41 sector diagram.
5. Once report 1 (bpMRI: T2W and DWI) has been done, this *cannot be altered* after looking at the DCE.

**Report 2 (mpMRI: T2W, DWI and DCE).**

1. The same radiologist must report both reports 1 and 2.
2. They will then be *unblinded* to the DCE sequence.
3. The radiologist should now complete report 2.
4. The location of the suspicious areas should be similarly labelled according to the PI-RADS V.2.1 41-sector diagram as previously mentioned.
5. On report 2, each of the existing lesions are additionally labelled as one of

**bpMRI positive, mpMRI positive.**

This occurs when a lesion scores 3, 4 or 5 on both bpMRI and mpMRI based on *either* Likert or PI-RADS V.2.1 scoring systems.

**bpMRI positive, mpMRI negative.**

This occurs when a lesion scores 3, 4 or 5 on bpMRI on either Likert or PI-RADS V.2.1 scoring systems, but also scores as 1 or 2 on mpMRI on both Likert and PI-RADS V.2.1 scoring systems.

**bpMRI negative, mpMRI positive.**

There are two instances in which *new targets* may be labelled and drawn onto report 2:

1. *New* lesions scoring 3, 4 or 5, identified by DCE not previously identified on bpMRI should be marked on as new lesions as *DCE targets* and *bpMRI negative, mpMRI positive*.
2. Lesions that appear *larger* on DCE should be treated as two separate targets.
  - One target depicts the completely overlapping segment from report 1 (bpMRI positive, mpMRI positive).
  - The non-overlapping part which would otherwise not be sampled should be labelled as a *new target* (bpMRI negative, mpMRI positive). This is a subjective decision by the radiologist. A typical example of when to declare this as a separate target is if the non-overlapping part enters an adjacent sector on the PI-RADS V.2.1 sector diagram.

A biopsy plan is recommended by the radiologist thereafter for the biopsy operator to follow.

#### Non-suspicious bpMRI and mpMRI

Men whose MRIs do not show suspicious areas on bpMRI and mpMRI (ie, scored 1 or 2 on Likert and PI-RADS V.2.1) will be stratified by PSA density. Men with PSA density of <0.15 ng/mL/mL will not undergo biopsy and men with PSA density of ≥0.15 ng/mL/mL will undergo systematic biopsy.

### Prostate biopsy procedures

#### MRI-targeted biopsy

Men will undergo MRI-targeted biopsy if either their bpMRI or mpMRI identifies a suspicious lesion which scores ≥3 on either Likert or PI-RADS V.2.1. Four targeted cores will be taken per suspicious lesion, and these should be stored and labelled in separate containers to ensure cancer detection from separate suspicious areas is ascertained.

#### Systematic biopsy

Systematic biopsies should be performed after targeted biopsies, with six cores taken from the contralateral side of the MRI lesion, focused on sampling the peripheral zone of the prostate. If there are bilateral MRI lesions or midline lesions, then no systematic biopsies are necessary.

Please see online supplemental appendix 2 for a detailed overview of how our biopsies will be conducted.

10.1136/bmjopen-2022-070280.supp2 Supplementary data

#### Prostate histopathology

Both the Gleason score and the Gleason grade group will be reported for the overall biopsy and for each individual target lesion.

#### Pre-trial assessments

For all patients, patient referral would follow clinical suspicion of PCa (eg, raised PSA or abnormal digital rectal examination). To confirm a patient’s eligibility, screening will be undertaken. Patients can enter the trial either before or after they have had their mpMRI scan. If patients are recruited after an mpMRI scan has been carried out, this will only be permitted if the MRI has not been seen by any clinician.

#### Registration procedures

Following consent and confirmation of eligibility, trial processes can commence. The patient will be registered and assigned a trial ID using a central online database (Marvin by XClinical).

#### Intervention procedures

All patients will undergo a full mpMRI scan. This includes T2W, DWI and DCE sequences.

#### Follow-up for results

If bpMRI and mpMRI are non-suspicious and PSA density is <0.15 ng/mL/mL, the patient will be counselled for standard of care follow-up, typically consisting of PSA surveillance. If a decision for prostate biopsy or other tests is made, these results will be recorded after which the participant completes the trial.

#### Multidisciplinary team (MDT) decision making for treatment eligibility

Treatment decisions will be per local standard of care, based on pathology results, and will be recorded. Subsequently, a virtual MDT meeting will be conducted and treatment eligibility decisions blinded to the DCE will be recorded. Once a decision has been recorded, the clinicians will be unblinded to the DCE sequence and the impact that this information makes on treatment eligibility will be evaluated.

#### MRI and pathology quality control

Quality control will be carried out at the end of the study by the PRIME chief radiologists reviewing the original MRIs, who will assess the MRI quality and re-report the MRI blinded to the study reports. Anonymised pathology slides from a proportion of patients may also be reviewed by central pathologists. Any slides assessed outside of the originating site will be returned to the original site after quality control. Quality control results will be reported but will not influence patient management or outcomes.

#### Cost-effectiveness

A within-trial incremental cost-effectiveness analysis will be conducted to calculate the difference in mean cost per diagnosis of clinically significant PCa if a strategy of bpMRI were adopted instead of the current mpMRI standard of care, over a time horizon of 30 days. The difference in cost of avoiding each additional case of clinically insignificant PCa diagnosed may also be calculated.

Costs of procedures will be estimated by applying standard unit costs to resource use data captured within the trial plus other procedures that would be offered to patients in either pathway. Estimates of the resources used (procedures, tests, radiotherapy, chemotherapy, other therapies, surveillance visits and other care events) on the two treatment pathways will be obtained for the theoretical bpMRI cohort using decisions made initially by the MDT with information from the bpMRI scan and any biopsies as a result of that scan, and estimates of the treatment pathway resources used in the theoretical mpMRI cohort will be made subsequently by the MDT on viewing additional information from the mpMRI scan and any further biopsies performed as a result of that scan. This thought experiment is required due to the ethical requirement to use all available information, that is, not just bpMRI and biopsies or just mpMRI and biopsies, when making the actual treatment decision with the patient.

The analysis perspective will be that of the NHS and personal social services. Standard unit costs (eg, NHS reference costs) will be supplemented by unit cost data from the participating trial sites. A microcosting study to provide this information will be undertaken in a small number of sites as part of the trial to investigate the resources employed to deliver bpMRI and mpMRI scans. This information will allow us to understand the MRI booking system, consumption of consumables and staff time as related to delivering bpMRI and mpMRI scans.

Depending on the within-trial cost-effectiveness findings, consideration will be given to extending this analysis using decision analytical modelling to estimate quality-adjusted life-years gained over a lifetime horizon. Quality of life information will be estimated from anonymised patient-level data by the same group from an earlier study in this instance.

### Outcomes

#### Primary outcome

The primary outcome will be the proportion of men with clinically significant PCa detected—any pattern 4 disease on any core (ie, Gleason score ≥3+4 or Gleason grade group ≥2). The time frame for assessment will be when biopsy results are available, at an expected average of 30 days post biopsy.

#### Secondary outcomes

Table 1 lists our secondary outcomes.

**Table 1:** Secondary outcomes in Prostate Imaging Using MRI±Contrast Enhancement

Outcome	Time frame for assessment
Proportion of men with clinically insignificant cancer (Gleason score 3+3/Gleason grade group 1)	When biopsy results are available, at an expected average of 30 days post biopsy
Agreement between bpMRI and mpMRI for score of suspicion	When MRI results are available, at an expected average of 30 days post MRI
Proportion of bpMRI scans and mpMRI whose quality was deemed adequate for reporting	When MRI results are available, at an expected average of 30 days post MRI
Agreement between bpMRI and mpMRI for radiological staging decision	When MRI results are available, at an expected average of 30 days post MRI
Agreement between bpMRI and mpMRI for treatment eligibility	When treatment eligibility is discussed in a multidisciplinary meeting, at an expected average of 30 days post biopsy
Test performance characteristics for bpMRI and mpMRI when using the Likert scoring system in comparison to the PI-RADS scoring system	When biopsy results are available, at an expected average of 30 days post MRI
Proportion of men with clinically significant cancer missed by bpMRI-targeted and mpMRI-targeted biopsies and detected by systematic biopsy	When biopsy results are available, at an expected average of 30 days post biopsy
Cost-effectiveness of bpMRI compared with mpMRI (cost per diagnosis of prostate cancer)	At an expected average of 30 days post intervention

bpMRI, biparametric MRI; mpMRI, multiparametric MRI; PI-RADS, Prostate Imaging Reporting and Data System.

### Sample size

The margin of clinical unimportance to allow a conclusion of non-inferiority of bpMRI to mpMRI to be made was set at 5 percentage points; that is, if the lower bound of the 95% CIs for the difference in detection rates of bpMRI-targeted biopsy compared with mpMRI-targeted biopsy is above −5 percentage points, then bpMRI will be deemed as non-inferior.

Using simulation, we used an mpMRI underlying probability of detecting clinically significant cancer of 38%^3^ and the following two key probabilities to determine the sample size:

- The probability that a patient found to have no suspicious lesions on bpMRI or have no clinically significant PCa on bpMRI-targeted biopsy will have clinically significant PCa on mpMRI-targeted biopsy.
- The probability that a patient found to have no suspicious lesions on mpMRI or have no clinically significant PCa on mpMRI-targeted biopsy will have clinically significant PCa on bpMRI-targeted biopsy.

Assuming the probability of A is greater than the probability of B, and applying McNemar’s test in each of 1000 simulation runs for each combination of probabilities A and B ranging from 0 to 0.05, a sample size of 400 patients gives more than 90% power across these probabilities of A and B. Accounting for 20% dropout or exclusion after enrolment, the study will require at least 500 patients.

#### Recruitment

At each participating site, enrolment will occur at outpatient clinics. With at least 25 sites, it is estimated that the trial will complete within 24 months of commencement. The trial opened for recruitment in April 2022 and the estimated completion date is April 2024.

#### Data collection methods

The electronic case report form (eCRF) system Marvin by XClinical will be used to collect data.

#### Patient-reported outcome measures

The International Index of Erectile Function and the International Prostate Symptom Score will be used to assess baseline erectile function and lower urinary tract symptoms, respectively. These questionnaires will aid the MDT decision making for treatment eligibility.

#### Patient retention

It is estimated that loss to follow-up will be no more than 20% due to the expected short time interval between enrolment and end of study. It is expected that the majority of patients will complete the trial within 4–6 weeks (tables 2 and 3).

**Table 2:** Participant timeline in the trial: the timeline for men enrolled to the trial prior to undergoing MRI

	Contact with patient				
	Visit 0*	Visit 1	Visit 2	Visit 3	Visit 4
Screening	X	X			
PIS given	X	X			
Consent	X	X			
IIEF-5 and IPSS questionnaires	X	X			
mpMRI			X		
Radiologists report bpMRI (T2W and DWI only)			X		
Radiologists report mpMRI (T2W, DWI and DCE)			X		
MRI-targeted biopsy and systematic biopsy				X	
Test results given and treatment decision					X
Follow-up for further investigations from treatment decision					X
Serious adverse event	Complete as required at any time following registration				
Withdrawal form	Complete as required at any time following registration				

*Visit 0 is an optional teleconsult, depending on local practice. Note that, where applicable, more than one visit can take place on the same day, depending on local practice (eg, in centres where an MRI is performed on the same day as subsequent biopsies).

bpMRI, biparametric MRI; DCE, dynamic contrast-enhanced sequence; DWI, diffusion-weighted sequence; IIEF-5, International Index of Erectile Function; IPSS, International Prostate Symptom Score; mpMRI, multiparametric MRI; PIS, patient information sheet; T2W, T2-weighted sequence.

**Table 3:** Participant timeline in the trial: the timeline for men enrolled after undergoing mpMRI as part of routine care

	Contact with patient				
	Visit 0	Visit 1	Visit 2	Visit 3	Visit 4
Screening		X			
PIS given		X			
Consent		X			
IIEF-5 and IPSS questionnaires	X				
mpMRI		X			
Radiologists report bpMRI (T2W and DWI only)		X			
Radiologists report mpMRI (T2W, DWI and DCE)		X			
MRI-targeted biopsy and systematic biopsy			X		
Test results given and treatment decision					X
Follow-up for further investigations from treatment decision					X
Serious adverse event	Complete as required at any time following registration				
Withdrawal form	Complete as required at any time following registration				

bpMRI, biparametric MRI; DCE, dynamic contrast-enhanced sequence; DWI, diffusion-weighted sequence; IIEF-5, International Index of Erectile Function; IP*SS*, International Prostate Symptom Score; mpMRI, multiparametric MRI; PIS, patient information sheet; T2W, T2-weighted sequence.

#### Statistical methods

A statistical analysis plan will be finalised before our database lock and before any statistical analysis occurs. A consort diagram will be presented. All continuous variables will be described using the mean and SD, or median and IQR, as appropriate. Categorical variables will be described using frequencies and percentages. Baseline characteristics will be examined and presented for those with and those without clinically significant PCa. The assumptions underpinning the statistical methods used will be assessed. The use of transformations will be considered to satisfy statistical assumptions.

#### Primary outcome analysis

The primary outcome is the difference in the proportion of men with clinically significant PCa, as detected by bpMRI-targeted biopsy compared with mpMRI-targeted biopsy. The proportion of men with clinically significant PCa, Gleason score of ≥3+4 or Gleason grade group of ≥2, detected by bpMRI-targeted biopsy, is defined as the number of men with clinically significant PCa identified on bpMRI-targeted biopsy divided by the number of men undergoing bpMRI. Similarly, the proportion of men with clinically significant PCa detected by mpMRI-targeted biopsy is defined as the number of men with clinically significant PCa identified on mpMRI-targeted biopsy divided by the number of men undergoing mpMRI. Methods that account for the paired nature of the data such as McNemar’s test will be used to compare bpMRI and mpMRI.

#### Secondary outcome analysis

The proportion of men with clinically insignificant cancer (any cancer core with Gleason score 3+3 or Gleason grade group 1) detected by bpMRI-targeted biopsy will be compared with that of mpMRI-targeted biopsy. The proportion of men with clinically insignificant cancer detected by bpMRI-targeted biopsy is defined as the number of men with clinically insignificant PCa identified on bpMRI-targeted biopsy divided by the number of men undergoing bpMRI. Similarly, the proportion of men with clinically insignificant cancer detected by mpMRI-targeted biopsy is defined as the number of men with clinically insignificant PCa identified on mpMRI-targeted biopsy divided by the number of men undergoing mpMRI. The same analytical approach described for clinically significant PCa will be applied.

The number and proportion of men scoring 1 or 2 (non-suspicious) or 3 (indeterminate) on bpMRI and mpMRI will be reported. A two-way table will be produced to show the agreement between the two MRI results using the Likert scoring system on a scale of 1–5.

The number and proportion of men with adequate standard of reporting on bpMRI and mpMRI will be reported.

A two-way table will be produced to show the number and proportion of patients with each radiological stage of bpMRI and mpMRI. Similarly, we will report the number and proportion of patients eligible for different treatment options following discussion of the bpMRI and mpMRI results in the MDT meeting.

Using histopathology as the reference standard, sensitivity, specificity, positive predictive value and negative predictive value with 95% CI of bpMRI and mpMRI will be reported. The following assumptions will be made, where non-suspicious MRI refers to a score of 1 or 2; suspicious MRI refers to a score of 3, 4 or 5 on the Likert and PI-RADS V.2.1 scoring systems; and absence of clinically significant cancer refers to a combination of clinical insignificant and no cancer.

The number and proportion of men with clinically significant cancer detected by systematic biopsy and not detected by bpMRI and mpMRI with targeted biopsy will be reported. A two-way table will be produced to show a comparison between systematic biopsy (no biopsy, clinically significant cancer, clinically insignificant cancer and no cancer) and the two MRI results with targeted biopsy (no biopsy, clinically significant cancer, clinically insignificant cancer and no cancer).

#### Sensitivity and other planned analyses

The primary outcome analysis will be repeated with a definition of clinically significant PCa being any primary pattern 4 disease with a Gleason score of 4+3 or a Gleason grade group of 3.

#### Monitoring

The National Cancer Imaging Translational Accelerator (NCITA) Global Prostate Trial Steering Committee (TSC) is responsible for the governance of the PRIME Study. A subgroup of independent TSC members form the data monitoring subcommittee (DMSC).

#### Roles and responsibilities of the TSC

The TSC’s role is to act as the oversight body for up to five PCa studies on behalf of the sponsor and funders. In addition, the independent members will form a DMSC to review safety. The role of the TSC is to provide oversight for the studies and advice through its chair to the chief investigators while working in tandem with the DMSC, sponsor, funders and host institution on all aspects of the studies. The rights, safety and well-being of the study participants are the most important consideration and should prevail over the interests of science and society.

#### Harms

Adverse events (AEs) will be defined as ‘any untoward medical occurrence in a clinical trial subject undergoing any intervention in the trial, which does not necessarily have a causal relationship with this treatment’.

Serious adverse events (SAEs) will be defined as ‘any untoward medical occurrence as a result of any intervention in the trial that:

- Results in death,
- Is life-threatening
- Requires hospitalisation or prolongation of existing inpatients’ hospitalisation, results in persistent or significant disability or incapacity’.

AEs and SAEs will be recorded until 30 days post biopsy. In the event that the patient does not undergo biopsy, AEs and SAEs should be recorded until 30 days post MRI.

Unexpected AEs will be recorded by a member of the research team or clinical team on an AE report form or eCRF. All SAEs must be recorded on an SAE report form or eCRF, which must be sent to the coordinating trial unit within 24 hours of knowledge of the SAE. Both AEs and SAEs should be recorded in the medical notes.

### Ethics and approval

The UK National REC (West Midlands Black Country Research Ethics Committee, Nottingham) gave favourable approval for PRIME protocol V.2.0 on 28 June 2021 (ref: 21/WM/0091). All participating centres have gained local and ethical approvals prior to receiving a site initiation visit and approval by the sponsor to open for recruitment.

### Patient and public involvement

Patients and public members were involved in defining the research question, evaluation of the research proposal, suggesting modifications to the trial, reviewing the patient information sheet, consent form and general practitioner letter. Patient groups and charities will also be involved in the dissemination of results.

### Consent

The clinical teams managing patients with suspected PCa who are referred to their centre will identify potential trial participants. Patient information sheets will be provided to patients. Members of staff who are trained to obtain informed consent, as indicated by the principal investigator (PI) on the delegation log for that site, will obtain the informed consent. A model patient information sheet is shown in online supplemental appendix 3.

10.1136/bmjopen-2022-070280.supp3 Supplementary data

### Confidentiality

The data of the participants will be recorded into the eCRF system and analysed without any personal identifiers by pseudoanonymised coded information. A site’s source documents and identification lists will be archived in a secured facility at that centre.

### Dissemination

Results of this trial will be disseminated through national and international conferences and papers. Authorship criteria will be based on recommendations of the International Committee of Medical Journal Editors. The participants and relevant patient support groups will be informed about the results of the trial.

### Access to data

Only authorised individuals within the PRIME Clinical Operations Group have access to the final data set. Individual PIs have access to their own data but not that of other sites.

**WHO Trial Registration Dataset**

Please see table 4 for the WHO trial registration dataset.

**Table 4:** WHO trial registration dataset

Data category	Information
Primary registry and trial identifying number	ClinicalTrials.gov: NCT04571840
Date of registration in the primary registry	1 October 2020
Sources of monetary or material support	Prostate Cancer UK. The John Black Charitable Foundation. European Association of Urology Research Foundation. The Dieckmann Foundation.
Primary sponsor	University College London
Secondary sponsor(s)	N/A
Contact for public queries	Mr Veeru Kasivisvanathan veeru.kasi@ucl.ac.uk Division of Surgery and Interventional Science, University College London, Third Floor, Charles Bell House, 43–45 Foley Street, London, W1W 7TS
Contact for scientific queries	Mr Veeru Kasivisvanathan veeru.kasi@ucl.ac.uk Division of Surgery and Interventional Science, University College London, Third Floor, Charles Bell House, 43–45 Foley Street, London, W1W 7TS
Public title/short title	Prostate Imaging Using MRI +/- Contrast Enhancement
Acronym	PRIME
Scientific title	A trial assessing whether bpMRI is non-inferior to multiparametric MRI in the diagnosis of clinically significant PCa
Countries of recruitment	Argentina Australia Belgium Brazil Canada Denmark France Finland Germany Italy The Netherlands Singapore Spain UK USA
Health condition or problem studied	Prostate neoplasm
Interventions	Device: MRI Diagnostic test: multiparametric MRI±prostate biopsy. Diagnostic test: bpMRI±prostate biopsy.
Intervention description	Active comparator: mpMRI. MRI with T2W, DWI and DCE followed by prostate biopsy if indicated on MRI and clinical findings. Diagnostic test: mpMRI±prostate biopsy. Experimental: bpMRI. MRI with T2W and DWI followed by prostate biopsy if indicated on MRI and clinical findings. Diagnostic test: bpMRI±prostate biopsy.
Key inclusion and exclusion criteria	Inclusion criteria Men at least 18 years of age referred with clinical suspicion of PCa. Serum PSA ≤20 ng/mL. Fit to undergo all procedures listed in the protocol. Able to provide written informed consent. Exclusion criteria Prior prostate biopsy. Prior treatment for PCa. Prior prostate MRI on a previous encounter. Contraindication to MRI. Contraindication to prostate biopsy. Unfit to undergo any procedures listed in the protocol.
Study type	Interventional Allocation: non-randomised Intervention model: single group assignment Intervention model description: within-person controlled, paired cohort, diagnostic evaluation study; participants undergo two index tests and a reference test Masking: single (care provider) Masking description: Radiologist assessing MRI for suspicion of PCa is blinded to the contrast sequence when reporting the bpMRI. After this report, they are unblinded to the contrast sequence and report the multiparametric MRI. All biopsies conducted as a result of MRI findings will be labelled as bpMRI and mpMRI, and diagnostic accuracy will be assessed against histological findings.
Date of first enrolment	5 April 2022
Target sample size	500
Recruitment status	Recruiting
Primary outcome(s)	Proportion of men with clinically significant cancer
Key secondary outcomes	Proportion of men with clinically insignificant cancer (Gleason score 3+3/Gleason grade group 1). Agreement between bpMRI and mpMRI for score of suspicion. Proportion of bpMRI scans and mpMRI whose quality was deemed adequate for reporting. Agreement between bpMRI and mpMRI for radiological staging decision. Agreement between bpMRI and mpMRI for treatment eligibility. Test performance characteristics for bpMRI and mpMRI when using the Likert scoring system in comparison to the PI-RADS scoring system. Proportion of men with clinically significant cancer missed by bpMRI and mpMRI-targeted biopsies and detected by systematic biopsy. Cost-effectiveness of bpMRI compared with mpMRI (cost per diagnosis of PCa).

bpMRI, biparametric MRI; DCE, dynamic contrast-enhance sequence; DWI, diffusion-weighted sequence; mpMRI, multiparametric MRI; PCa, prostate cancer; PRIME, Prostate Imaging Using MRI±Contrast Enhancement; T2W, T2-weighted sequence.

### Current Protocol Version

The current protocol is V.2.0, issued 27 April 2021. The current protocol amendment number is 01. For full amendment history, please see table 5.

**Table 5:** Revision chronology for amendments to protocol

Protocol version to date	Reasons for amendments
V.1.0, issued 24 August 2020	Original protocol
V.2.0, issued 27 April 2021	Main reasons for amendment: minor changes to make existing trial documents clearer. Main changes: Updated Section 18 Record Keeping and Archiving. Added the sentence, ‘Identifiable data will be kept by the site for 10 years, and non-identifiable data will be kept for a minimum of 20 years’. Version number and date added to all pages.

### Roles and Responsibilities

Please see table 6 for roles and responsibilities of the trial sponsor and involved committees.

**Table 6:** Roles and responsibilities in the prime trial

Role	Details and responsibilities
Trial sponsor	University College London (UCL) Sponsor’s Edge reference: 135 819 Email: Rand.D@uclh.nhs.uk The trial sponsor did not provide any funding for the study. UCL has the role of research governance sponsor of PRIME. UCL adopted the study as sponsor after the UCL CCTU carried out a trial adoption process which involved the UCL CCTU reviewing the protocol to ensure it conformed to high standards of trial conduct and met the governance requirements of UCL. The UCL CCTU is responsible for oversight of the trial. The sponsor plays no role in data collection, management, analysis and interpretation of data, writing of the report or the decision to submit the report for publication.
PRIME operations group	The PRIME operations group consists of the CI, the Clinical Operations Group, National Cancer Imaging Translational Accelerator, the UCL Surgical and Interventional Trials Unit and the electronic case report form database managers. This group is responsible for Study planning. Preparation of protocol and revisions. Assistance with international review board/independent ethics committee applications. Preparation of investigators brochure and CRFs. Organisation of steering committee meetings. Provide annual progress reports to the ethics committee. Reporting serious adverse events to the sponsor and ethics committee when necessary. Responsible for trial master file. Budget administration and contractual issues with individual centres. Advice for PIs. Site initiation visits. Data verification and management. Central monitoring and resolving data queries with clinicians and nurses at the trial sites. Maintenance of the trial information technology system. Publication of study reports.
PI	At each participating site, the PI is responsible for the conduct of the clinical trial to ensure the safety of participants and the reliability and robustness of the data generated. They will be responsible for identification, recruitment, data collection and completion of CRFs, along with follow-up of trial patients and adherence to trial protocol. The PIs as leader of the research team may delegate their duties to members of their team.
Global TSC	The NCITA global prostate TSC is responsible for the governance of the PRIME study, and they have delegated safety to a DMSC. Roles and responsibilities To act as the oversight body for up to five prostate cancer studies on behalf of the sponsor and funders. In addition, the independent members will form a subcommittee to review safety. The role of the TSC is to provide oversight for the studies and provide advice through its chair to the CIs, work in tandem with the DMSC, sponsor, funders and host institution on all aspects of the studies. The rights, safety and well-being of the study participants are the most important consideration and should prevail over the interests of science and society.

CCTU, Comprehensive Clinical Trials Unit; CI, chief investigator; DMSC, data monitoring subcommittee; PI, principal investigator; TSC, trial steering committee; UCL, University College London.

## Supplementary Material

Reviewer comments

Author's manuscript

## Data Availability

Data sharing not applicable as no datasets generated and/or analysed for this Protocol.
