# Supplementary material for: Comparing biparametric to multiparametric MRI in the diagnosis of clinically significant prostate cancer in biopsy-naive men (PRIME): a prospective, international, multicentre, non-inferiority within-patient, diagnostic yield trial protocol
Source: BMJ Open. 2023 Apr 5;13(4):e070280. doi: 10.1136/bmjopen-2022-070280 (PMC10083803; doi:10.1136/bmjopen-2022-070280)
Supplement: Supplementary data [file bmjopen-2022-070280supp001.pdf]

TRIAL IDENTIFIER: PARTICIPANT INITIALS:

Reporting Proforma (bpMRI):

Report 1 – Biparametric MRI (bpMRI) Report

This report should be completed **without looking at the contrast sequence.**

If applicable, complete the Target boxes & link these to your drawings of the Targets (e.g. with lines / colours)

Target 1	Likert:<br>PIRADS:

Target 2	Likert:<br>PIRADS:

Target 3	Likert:<br>PIRADS:

Target 4	Likert:<br>PIRADS:

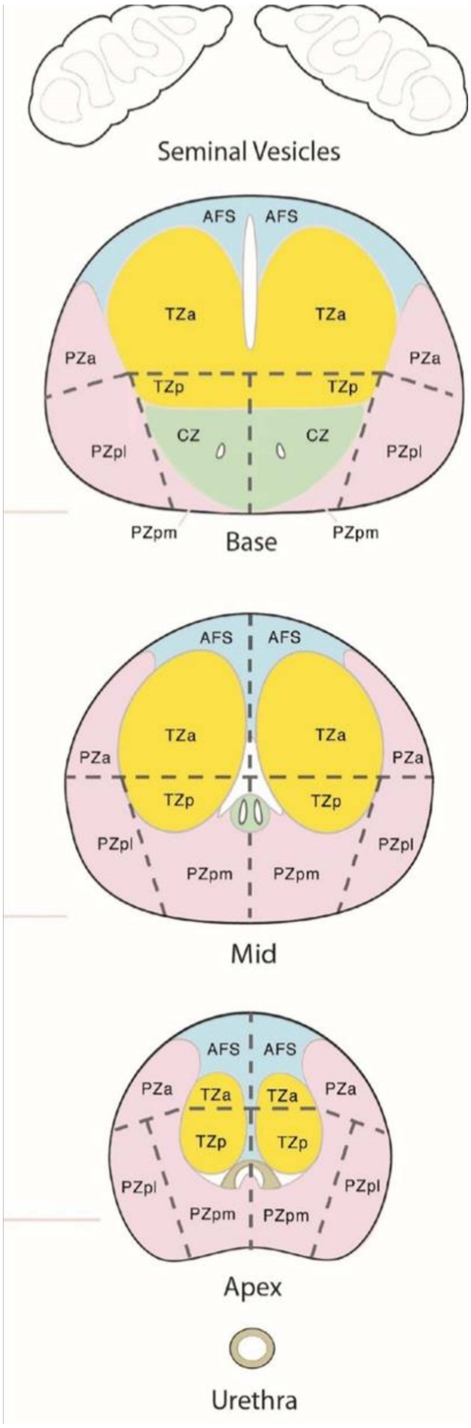

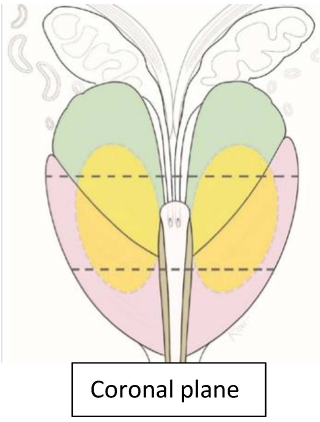

Coronal plane

Reminder of Likert Score: Likelihood of target containing significant cancer:

1 = Highly unlikely

2 = Unlikely

3 = Equivocal

4 = Likely

5 = Highly Likely

In the case of diffuse changes on **both sides** of the prostate scoring ≥3 (Likert), the diffuse changes on each side of the prostate can be arbitrarily treated as **separate targets. "**Diffuse change" is defined as an intermediate or low T2 signal that occupies the majority of at least one side of the peripheral zone, without a defined border

Asif A, *et al. BMJ Open* 2023; 13:e070280. doi: 10.1136/bmjopen-2022-070280

TRIAL IDENTIFIER: PARTICIPANT INITIALS:

1. Radiologists should **first** annotate, draw and label the diagram on the first page with **up to 3** suspicious areas scoring ≥ 3 on the Likert scale (L) of suspicion (1–5). Clinical information is permitted to be used to influence the score.
2. Radiologists should then score suspicious areas **strictly** using the PI-RADS v2.1 (P) criteria, **without** allowing clinical information to influence the score.
3. If an additional area of suspicion is identified when scoring with PI-RADS v2.1 that was **not** present on Likert, please draw on this 4th suspicious area.

A maximum of **4 targets** can be drawn on this report.

---

1. Every lesion **must have both** a Likert and PI-RADS v2.1 score marked on.
2. Mark the **most suspicious** area, "Target 1".
   a. Mark the **next most suspicious area**, "Target 2".
   b. Mark the **subsequent most suspicious area**, "Target 3" and so on.
3. **On the diagram above, every** lesion drawn must have the following marked and labelled:
   a. Target number
   b. Likert score
   c. PI-RADS v2.1 score
4. Please then insert these into **Table 1** and fill out the rest of the proforma.

e.g. Target 1. Likert 3. PI-RADS 1.

MRI Scanner and Clinical Information

<table>
<tr><td>Patient age (years):</td><td></td><td>PSA (ng/ml):</td><td></td><td rowspan="3">Which MRI scanner was used?
1. ☐ SCANNER ONE
2. ☐ SCANNER TWO
3. ☐ SCANNER THREE</td></tr>
<tr><td>MRI volume of prostate (ml):</td><td></td><td>PSA Density (ng/ml/ml):</td><td></td></tr>
<tr><td>Field Strength of Magnet</td><td>☐ 1.5T ☐ 3T</td><td></td><td></td></tr>
</table>

Confirmation of blinding

Confirmation by another individual / system that the radiologist is **blinded** to DCE images (**mandatory**)	☐ Yes	☐ No

---

Asif A, *et al. BMJ Open* 2023; 13:e070280. doi: 10.1136/bmjopen-2022-070280

TRIAL IDENTIFIER: PARTICIPANT INITIALS:

Table 1. Please only enter Targets below if the Likert or PI-RADS v2.1 score is ≥ 3.

**TARGET SPECIFIC INFORMATION**	TARGET 1	TARGET 2	TARGET 3	TARGET 4
Location of suspicious area(s) (select **one** option):	☐ Right<br>☐ Left<br>☐ Bilateral	☐ Right<br>☐ Left<br>☐ Bilateral	☐ Right<br>☐ Left<br>☐ Bilateral	☐ Right<br>☐ Left<br>☐ Bilateral
Location in prostate according to PI-RADS v2.1 41-sector diagram (select **the one main** location which contains the target):	☐ Base<br>☐ Mid<br>☐ Apex<br>☐ Seminal Vesicle	☐ Base<br>☐ Mid<br>☐ Apex<br>☐ Seminal Vesicle	☐ Base<br>☐ Mid<br>☐ Apex<br>☐ Seminal Vesicle	☐ Base<br>☐ Mid<br>☐ Apex<br>☐ Seminal Vesicle
Main sector which contains the lesion according to PI-RADS v2.1 41-sector diagram (write **one**, *e.g.* "PZpl"):				
Likert score of suspicion (1–5):				
PI-RADS v2.1 score of suspicion (1–5):				
Target appearance (select **one**):<br>The default is focal, unless there is diffuse change in the peripheral zone	☐ Focal<br>☐ Diffuse	☐ Focal<br>☐ Diffuse	☐ Focal<br>☐ Diffuse	☐ Focal<br>☐ Diffuse
Biaxial diameter on sequence where it was largest, in axial plane (mm x mm):				
Sequence used to measure biaxial diameter (select **one**):	☐ T2<br>☐ High b<br>☐ ADC	☐ T2<br>☐ High b<br>☐ ADC	☐ T2<br>☐ High b<br>☐ ADC	☐ T2<br>☐ High b<br>☐ ADC

Please complete the **overall scores** <u>regardless</u> of whether there are any Targets identified above:

**Overall patient Likert score**<br>Enter the highest Likert score		**Overall patient PIRADS v2.1 score**<br>Enter the highest PI-RADS v2.1 score	

If there are no Targets scoring ≥ 3 on either scoring system, then the overall Likert and PI-RADS v2.1 score will be 1 or 2.

TRIAL IDENTIFIER: PARTICIPANT INITIALS:

Table 2. Staging information. Complete **only if** a Target has been identified above:

Radiological stage:	☐ T2a	☐ T2b	☐ T2c	☐ T3a	☐ T3b	☐ T4
	Radiological T3a = unequivocal extracapsular disease					
Likelihood of **right**-sided extracapsular spread*:<br>1 = highly **unlikely**, 3 = equivocal, 5 = highly **likely**	☐ 1	☐ 2	☐ 3	☐ 4	☐ 5	
Likelihood of **left**-sided extracapsular spread*:	☐ 1	☐ 2	☐ 3	☐ 4	☐ 5	
Likelihood of **right** seminal vesicle involvement:	☐ 1	☐ 2	☐ 3	☐ 4	☐ 5	
Likelihood of **left** seminal vesicle involvement:	☐ 1	☐ 2	☐ 3	☐ 4	☐ 5	
Likelihood of urethral sphincter involvement:	☐ 1	☐ 2	☐ 3	☐ 4	☐ 5	
Likelihood of bladder neck involvement:	☐ 1	☐ 2	☐ 3	☐ 4	☐ 5	
Likelihood of rectal involvement:	☐ 1	☐ 2	☐ 3	☐ 4	☐ 5	

**See* PI-RADS v2.1 guidelines for examples of features suggestive of extracapsular spread.

MRI Quality: Please **complete** this for **all** MRIs regardless of whether a Target was identified:

Was there a problem with the quality of the T2W sequence?	☐ Yes			☐ No
Was there a problem with the quality of the DWI sequence?	☐ Yes			☐ No
If there were problems, please describe these (tick **all** that apply):<br>For T2W:<br>For DWI:	☐ Rectal air<br>☐ Rectal air	☐ Movement artefact<br>☐ Movement artefact	☐ Prosthesis<br>☐ Prosthesis	☐ Other<br>☐ Other
If other, please describe:				
Was the quality of the scan sufficient for you to make a diagnostic assessment?	☐ Yes	☐ No		
Hypothetically, if this patient only had this biparametric MRI scan:<br>• Would you typically have recommended a **repeat bpMRI?**	☐ Yes	☐ No		
• Would you typically have recommended a **contrast sequence** to be done?	☐ Yes	☐ No		

Radiologist (Forename, Surname):		Date of MRI:	
		Date of Report:	

TRIAL IDENTIFIER: PARTICIPANT INITIALS:

Reporting Proforma (mpMRI):

Report 2 – Multiparametric MRI (mpMRI) Report

The same radiologist should annotate the diagrams below after they are **unblinded** to the **DCE sequence**. This report will be used by the biopsy operator to perform **targeted biopsy**.

A total of **maximum 8 suspicious areas scoring ≥ 3 on either Likert or PI-RADS v2.1** can be annotated in this report.

PART ONE: TARGETS SEEN ON BPMRI

1. First, copy any targets drawn on **Report 1** (bpMRI) onto this report (**Report 2 – mpMRI**).
   a. Draw them on the diagram.
   b. Specify their biparametric MRI status (bpMRI +ve or bpMRI -ve) when you label each lesion.
   c. Add the information about each target to **Table 1** as indicated.
2. Upon viewing the **DCE findings**, for each of these lesions, please specify their multi-parametric MRI status (mpMRI +ve or mpMRI -ve) on the diagram then specify **updated** Likert (L) and PI-RADS v2.1 (P) scores on mpMRI.

Flow diagram: how to complete this proforma for lesions identified on bpMRI (Report 1)

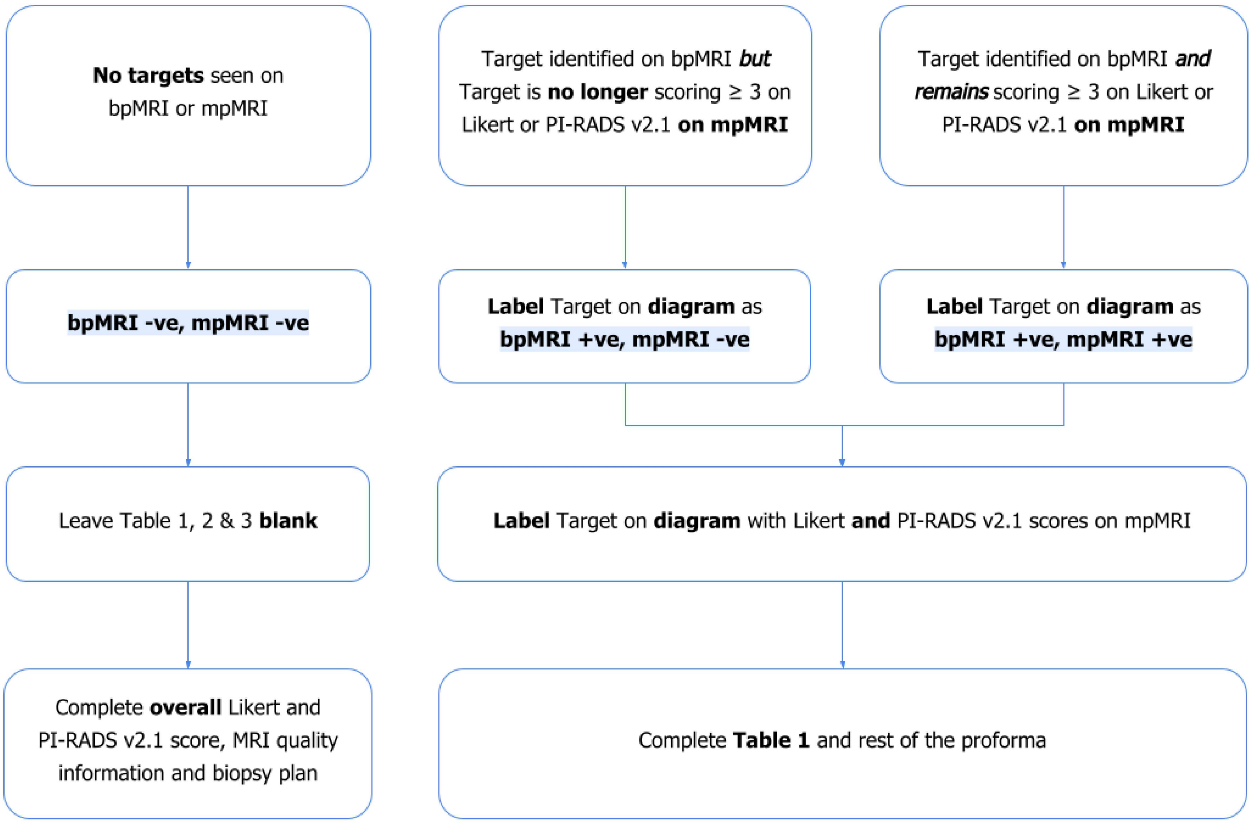

Asif A, *et al. BMJ Open* 2023; 13:e070280. doi: 10.1136/bmjopen-2022-070280

TRIAL IDENTIFIER: PARTICIPANT INITIALS:

Please draw any Targets on this diagram
and label them according to the flow diagram on Page 1

If applicable, complete the Target boxes & link these to your drawings of the Targets (e.g. with lines / colours)

Target 1	**bpMRI**	**mpMRI**
	Likert:	Likert:
	PIRADS:	PIRADS:

Target 2	**bpMRI**	**mpMRI**
	Likert:	Likert:
	PIRADS:	PIRADS:

Target 3	**bpMRI**	**mpMRI**
	Likert:	Likert:
	PIRADS:	PIRADS:

Target 4	**bpMRI**	**mpMRI**
	Likert:	Likert:
	PIRADS:	PIRADS:

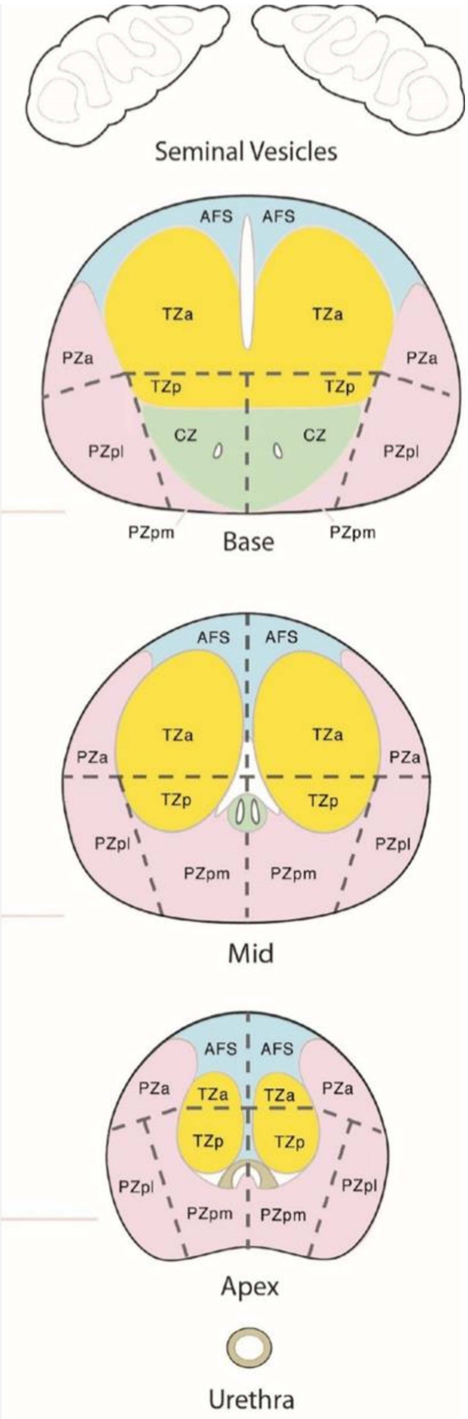

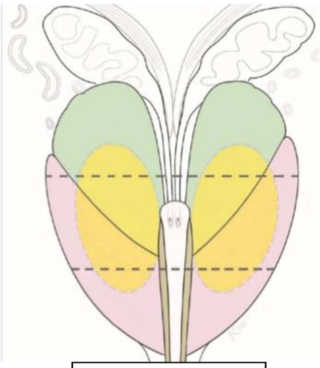

Coronal plane

**DCE-** Target 1	Likert:
	PIRADS:

**DCE-** Target 2	Likert:
	PIRADS:

**DCE-** Target 2	Likert:
	PIRADS:

**DCE-** Target 4	Likert:
	PIRADS:

In the case of diffuse changes on **both sides** of the prostate scoring ≥3 (Likert), the diffuse changes on each side of the prostate can be arbitrarily treated as **separate Targets. "**Diffuse change" is defined as an intermediate or low T2 signal that occupies the majority of at least one side of the peripheral zone, without a defined border.

TRIAL IDENTIFIER: PARTICIPANT INITIALS:

MRI Scanner and Clinical Information. Complete for all patients:

Patient age (years)		PSA (ng/ml):	
MRI volume of prostate (ml):		PSA Density (ng/ml/ml):	

Table 1. Information from Targets **originally** identified on the **biparametric MRI** (if applicable):

**TARGET SPECIFIC INFORMATION**	TARGET 1	TARGET 2	TARGET 3	TARGET 4
**COPY FROM REPORT 1 (BPMRI):**				
Location of suspicious area(s) (select **one** option):	☐ Right ☐ Left ☐ Bilateral	☐ Right ☐ Left ☐ Bilateral	☐ Right ☐ Left ☐ Bilateral	☐ Right ☐ Left ☐ Bilateral
Location in prostate according to PI-RADS v2.1 41-sector diagram (select **the one main** location which contains the target):	☐ Base ☐ Mid ☐ Apex ☐ Seminal Vesicle	☐ Base ☐ Mid ☐ Apex ☐ Seminal Vesicle	☐ Base ☐ Mid ☐ Apex ☐ Seminal Vesicle	☐ Base ☐ Mid ☐ Apex ☐ Seminal Vesicle
Main sector which contains the lesion according to PI-RADS v2.1 41-sector diagram (write **one** sector, *e.g.* "PZpl"):				
Biparametric MRI Likert score (1–5):				
Biparametric MRI PI-RADS v2.1 score (1–5):				
**RE-ASSESS, TAKING INTO ACCOUNT INFORMATION FROM DCE SEQUENCE (MPMRI):**				
Multiparametric MRI Likert score (1–5):				
Multiparametric MRI PI-RADS v2.1 score (1–5):				
Target appearance (select **one**):	☐ Focal ☐ Diffuse	☐ Focal ☐ Diffuse	☐ Focal ☐ Diffuse	☐ Focal ☐ Diffuse
Biaxial diameter on sequence where it was largest, in axial plane (mm x mm):				
Sequence used to measure biaxial diameter (select **one**):	☐ T2 ☐ High b ☐ ADC ☐ DCE	☐ T2 ☐ High b ☐ ADC ☐ DCE	☐ T2 ☐ High b ☐ ADC ☐ DCE	☐ T2 ☐ High b ☐ ADC ☐ DCE

TRIAL IDENTIFIER: PARTICIPANT INITIALS:

PART TWO: NEW DCE-TARGETS ON DYNAMIC CONTRAST ENHANCED SEQUENCE

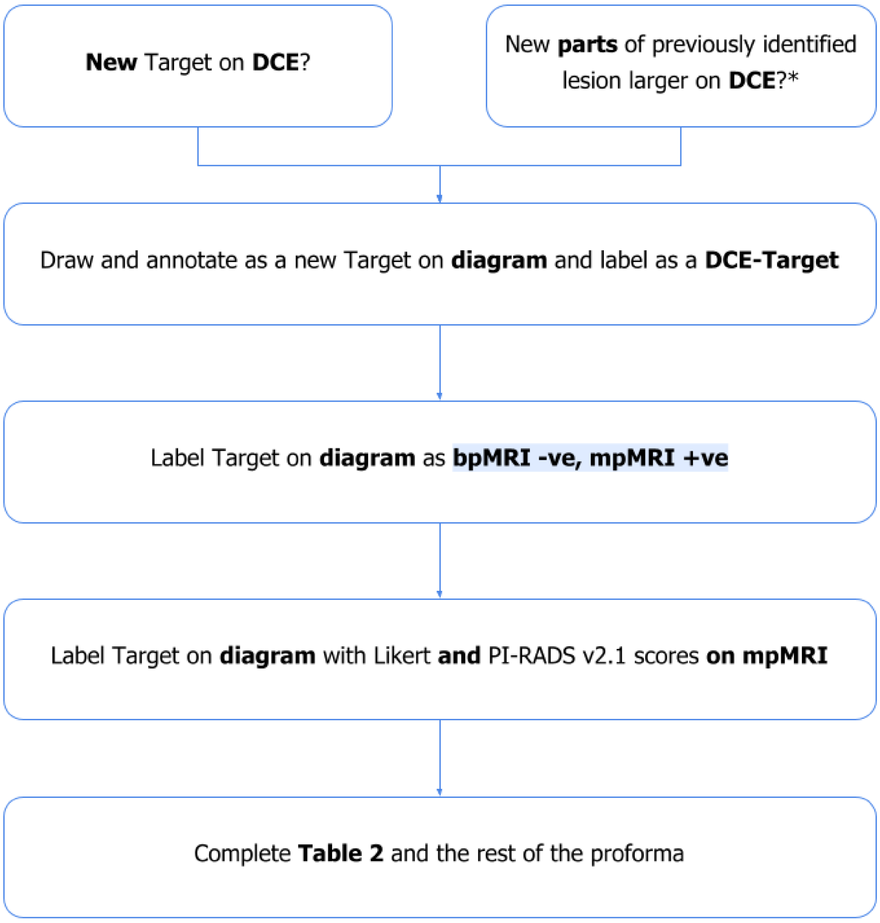

Please note: this is a **subjective decision** by the radiologist as to whether new parts of an existing lesion on bpMRI would need to be declared as **a new target** in order **not to be missed on biopsy**. A clear example of when to declare a new DCE-Target would be if the non-overlapping part of the lesion on DCE crosses into a new sector on the PI-RADSv2.1 sector diagram

---

5. Any new targets should be labelled **DCE-Target-x.**
   a. The first new, most suspicious, target should be **DCE-Target-1**. The second if applicable, **DCE-Target-2** and so on.
6. A maximum of **4 new targets** can be drawn on this report (**Report 2**).
   a. Thus, a maximum of **8 targets** can be drawn in total (4 carried over from **Report 1** and 4 new DCE targets).
7. **On the diagram on Page 2, every** lesion drawn must have the following marked and labelled:
   a. Target number
   b. bpMRI status (positive or negative)
   c. mpMRI status (positive or negative)
   d. Likert score for mpMRI
   e. PI-RADS v2.1 score for mpMRI

   e.g. DCE-Target-1. bpMRI negative. mpMRI positive. Likert 4. PI-RADS 2.
8. Then complete **Table 2** and the rest of the MRI proforma.

TRIAL IDENTIFIER: PARTICIPANT INITIALS:

Table 2. Information from Targets identified **ONLY** by DCE, which were **not** identified on the **biparametric MRI** (if applicable). If there are no DCE-Targets then leave Table 2 blank & move onto overall patient Likert & PI-RADs scores):

**TARGET SPECIFIC INFORMATION**	DCE-TARGET 1	DCE-TARGET 2	DCE-TARGET 3	DCE-TARGET 4
DCE-Target (select if **new** lesion or **part of existing lesion bigger on DCE**):	☐ New<br>☐ Existing	☐ New<br>☐ Existing	☐ New<br>☐ Existing	☐ New<br>☐ Existing
Location of suspicious area(s) (select **one**):	☐ Right<br>☐ Left<br>☐ Bilateral	☐ Right<br>☐ Left<br>☐ Bilateral	☐ Right<br>☐ Left<br>☐ Bilateral	☐ Right<br>☐ Left<br>☐ Bilateral
Location in prostate according to PI-RADS v2.1 41-sector diagram (select **the one main** location which contains the target):	☐ Base<br>☐ Mid<br>☐ Apex<br>☐ Seminal Vesicle	☐ Base<br>☐ Mid<br>☐ Apex<br>☐ Seminal Vesicle	☐ Base<br>☐ Mid<br>☐ Apex<br>☐ Seminal Vesicle	☐ Base<br>☐ Mid<br>☐ Apex<br>☐ Seminal Vesicle
Main sector which contains the lesion according to PI-RADS v2.1 41-sector diagram (write **one**, *e.g.* "PZpl"):				
Multiparametric MRI Likert score (1–5):				
Multiparametric MRI PI-RADS v2.1 score (1–5):				
Target appearance (select **one**):	☐ Focal<br>☐ Diffuse	☐ Focal<br>☐ Diffuse	☐ Focal<br>☐ Diffuse	☐ Focal<br>☐ Diffuse
Biaxial diameter on dominant sequence in axial plane (mm x mm):				
Looking back again at the T2W and DWI **only**, is the DCE-target identified here actually visible on the bpMRI?	☐ No<br>☐ Yes	☐ No<br>☐ Yes	☐ No<br>☐ Yes	☐ No<br>☐ Yes
If you answered **Yes**, please specify whether the lesion was missed on 1st look ***or*** whether it was seen but scored a 1 or 2 on PI-RADS v2.1 **and** Likert	☐ Missed on 1st look<br>☐ Seen on 1st look but scored a 1 or 2	☐ Missed on 1st look<br>☐ Seen on 1st look but scored a 1 or 2	☐ Missed on 1st look<br>☐ Seen on 1st look but scored a 1 or 2	☐ Missed on 1st look<br>☐ Seen on 1st look but scored a 1 or 2

Asif A, *et al. BMJ Open* 2023; 13:e070280. doi: 10.1136/bmjopen-2022-070280

TRIAL IDENTIFIER: PARTICIPANT INITIALS:

Please complete the **overall scores** regardless of whether there are any Targets identified above:

**Overall patient Likert score**<br>Enter the highest Likert score on **either** biparametric MRI or multiparametric MRI		**Overall patient PI-RADS v2.1 score**<br>Enter the highest PI-RADS v2.1 score on **either** biparametric MRI or multiparametric MRI	

Please note: if a lesion was suspicious on biparametric MRI but **not** suspicious on mpMRI (*i.e.* bpMRI +ve, mpMRI -ve), it should still be biopsied if either the Likert or PI-RADS v2.1 score on bpMRI is ≥ 3. This highest score on either bpMRI or mpMRI should be entered above.

Table 3. Staging information. Complete **only** if a Target has been identified above. Select **one option** each time:

Radiological stage:	☐ T2a	☐ T2b	☐ T2c	☐ T3a	☐ T3b	☐ T4
	Radiological T3a = unequivocal extracapsular disease					
Likelihood of **right**-sided extracapsular spread*:<br>1 = highly **unlikely**, 3 = equivocal, 5 = highly **likely**	☐ 1	☐ 2	☐ 3	☐ 4	☐ 5	
Likelihood of **left**-sided extracapsular spread*:	☐ 1	☐ 2	☐ 3	☐ 4	☐ 5	
Capsular involvement on **DCE**:	☐ No	☐Yes, on **right**	☐Yes, on **left**	☐Yes, on **both sides**		
Likelihood of **right** seminal vesicle involvement:	☐ 1	☐ 2	☐ 3	☐ 4	☐ 5	
Likelihood of **left** seminal vesicle involvement:	☐ 1	☐ 2	☐ 3	☐ 4	☐ 5	
Seminal vesicle involvement on **DCE**:	☐ No	☐Yes, on **right**	☐Yes, on **left**	☐Yes, on **both sides**		
Likelihood of urethral sphincter involvement:	☐ 1	☐ 2	☐ 3	☐ 4	☐ 5	
Urethral sphincter involvement on **DCE**:	☐ No	☐Yes, on **right**	☐Yes, on **left**	☐Yes, on **both sides**		
Likelihood of bladder neck involvement:	☐ 1	☐ 2	☐ 3	☐ 4	☐ 5	
Bladder neck involvement on **DCE**:	☐ No	☐ Yes				
Likelihood of rectal involvement:	☐ 1	☐ 2	☐ 3	☐ 4	☐ 5	
Rectal wall involvement on **DCE**:	☐ No	☐ Yes				

**See* PI-RADS v2.1 guidelines for examples of features suggestive of extracapsular spread.

Asif A, *et al. BMJ Open* 2023; 13:e070280. doi: 10.1136/bmjopen-2022-070280

TRIAL IDENTIFIER: PARTICIPANT INITIALS:

MRI Quality. Please **complete** this for all MRIs regardless of whether a Target was identified:

Was there a problem with the quality of the DCE sequence?	☐ Yes			☐ No
If problems with DCE, please specify:<br>Tick **all** that apply	☐ Rectal air	☐ Movement artefact	☐ Prosthesis	☐ Other
If other, please describe:				
Was the quality of the scan sufficient for you to make a diagnostic assessment?	☐ Yes		☐ No	
Based on the quality of the mpMRI scan and your typical practice, would you recommend a repeat multiparametric MRI be performed?	☐ Yes		☐ No	

Biopsy protocol guidelines

It is **mandatory** to follow these recommendations below:

Number of MRI targets	Location of MRI targets in prostate	Number of MRI-targeted biopsy cores	Number of contralateral systematic cores	Total number of biopsy cores
0	If PSA Density is < 0.15ng/ml/ml			0
0	If PSA Density is ≥ 0.15ng/ml/ml, then 12 systematic biopsy cores are taken (6 from each side)			12
1	Unilateral	4	6	10
2	Unilateral	8	6	14
3	Unilateral	12	6	18
4–8	Unilateral	16–32	6	22–38
1	Bilateral (*e.g.* crossing midline)	4	0	4
2	Bilateral	8	0	8
3	Bilateral	12	0	12
4–8	Bilateral	16–32	0	16–32

Note: For 4–8 MRI targets, determine the number of MRI-targeted cores by using the principle of 4 cores per MRI target.

Asif A, *et al. BMJ Open* 2023; 13:e070280. doi: 10.1136/bmjopen-2022-070280

TRIAL IDENTIFIER: PARTICIPANT INITIALS:

Recommended Biopsy Plan for biopsy operator to follow

The radiologist should now complete this biopsy plan which should be passed directly to the person performing biopsy (if one is required) along with the labelled diagram on Page 2:

Even if radiologists do not typically write biopsy plans, we request they do this here following the protocol in the table above, in order to reduce errors between linking the MRI information to the protocol biopsy plan.

Number of MRI-targets to biopsy with MRI-targeted biopsy:<br>(*Note:* Targets which are only suspicious on bpMRI should still be biopsied. The number of MRI-targets for biopsy therefore includes MRI targets identified only on bpMRI, only on mpMRI or on both bpMRI and mpMRI and on either the Likert scoring system or the PIRADSv2.1 scoring system)	
Total number of MRI-targeted biopsy cores to be taken:<br>(*Note:* 4 biopsy cores should be taken per lesion)	
Total number of systematic biopsy cores to be taken:<br>(*Note:* Systematic cores should be peripheral zone-focused cores)	
Number of systematic cores to be taken from **right** side of prostate:<br>(*Note:* do not take systematic cores from the same side as an MRI target)	
Number of systematic cores to be taken from **left** side of prostate:<br>(*Note:* do not take systematic cores from the same side as an MRI target)	
Total number of systematic and targeted cores to be taken	

Radiologist<br>(Forename, Surname):		Date:	

Asif A, *et al. BMJ Open* 2023; 13:e070280. doi: 10.1136/bmjopen-2022-070280
