# Supplementary material for: Comparing biparametric to multiparametric MRI in the diagnosis of clinically significant prostate cancer in biopsy-naive men (PRIME): a prospective, international, multicentre, non-inferiority within-patient, diagnostic yield trial protocol
Source: BMJ Open. 2023 Apr 5;13(4):e070280. doi: 10.1136/bmjopen-2022-070280 (PMC10083803; doi:10.1136/bmjopen-2022-070280)
Supplement: Supplementary data [file bmjopen-2022-070280supp002.pdf]

Supplementary Appendix 2: Detailed PRIME Biopsy Plans

To be pragmatic and allow results to be generalisable to biopsy practice around the world, biopsies can be performed transperineally (**Figures 1** and **2**) or transrectally (**Figures 3 and 4**) as per local practice. We split this Appendix into these sections, respectively.

If there is an MRI lesion (scores 3, 4 or 5 on *either* Likert or PI-RADS v2.1 scoring systems), then MRI-targeted biopsy and some limited contralateral systematic biopsy should be performed. MRI-targeted biopsy should be performed **first,** with 4 cores per suspicious area. Then the systematic biopsy cores should be taken but avoid taking biopsies from the same side of the prostate that targeted biopsies were taken from.

Table of Contents

Systematic Transperineal Biopsy Schema 2
Non-suspicious MRI but a PSA Density of ≥ 0.15ng/mL/mL scenario 2
Suspicious MRI lesion scenarios 4
Single lesion scenario 4
Bilateral lesions scenario 4
Lesion crossing midline scenario 5
Bilateral diffuse change on Likert scoring scenario 5
New lesion is revealed on DCE sequence scenario 6
New part of existing lesion is revealed on DCE sequence scenario 6
Systematic Transrectal Biopsy Schema 7
Non-suspicious MRI but a PSA Density of ≥ 0.15ng/mL/mL scenario 7
Suspicious MRI lesion scenarios 9
Single lesion scenario 9
Bilateral lesions scenario 10
Lesion crossing midline scenario 11
Bilateral diffuse change on Likert scoring scenario 12
New lesion is revealed on DCE sequence scenario 13
New part of existing lesion is revealed on DCE sequence scenario 14
Summary Biopsy Guidelines 16

Designed by Aqua Asif

Systematic Transperineal Biopsy Schema

Figures 1 and **2A-F** depict examples of how to perform the systematic biopsy in the **absence** of an MRI lesion and in the **presence** of MRI lesions, respectively.

Non-suspicious MRI but a PSA Density of ≥ 0.15ng/mL/mL scenario

In patients with a **non-suspicious MRI but a PSA Density of ≥ 0.15ng/mL/mL**, 12-core systematic biopsy should be performed (**Figure 1**).

The number of systematic cores that should be taken per patient is **12.**

Systematic biopsy cores are taken from:

- Right anterior zone (2 cores)
- Right mid zone (2 cores)
- Right posterior zone (2 cores)
- Left anterior zone (2 cores)
- Left mid zone (2 cores)
- Left posterior zone (2 cores)

Systematic biopsy cores should be stored and labelled in a way that their **location** can be identified when the pathologist reports the result.

Figure 1. The transperineal biopsy schema for men with a **non-suspicious MRI** (scores 1 or 2 on both Likert and PI-RADS v2.1 scoring systems) *but* a PSA Density of ≥0.15ng/mL/mL, undergoing 12-core systematic biopsy.

Designed by Aqua Asif

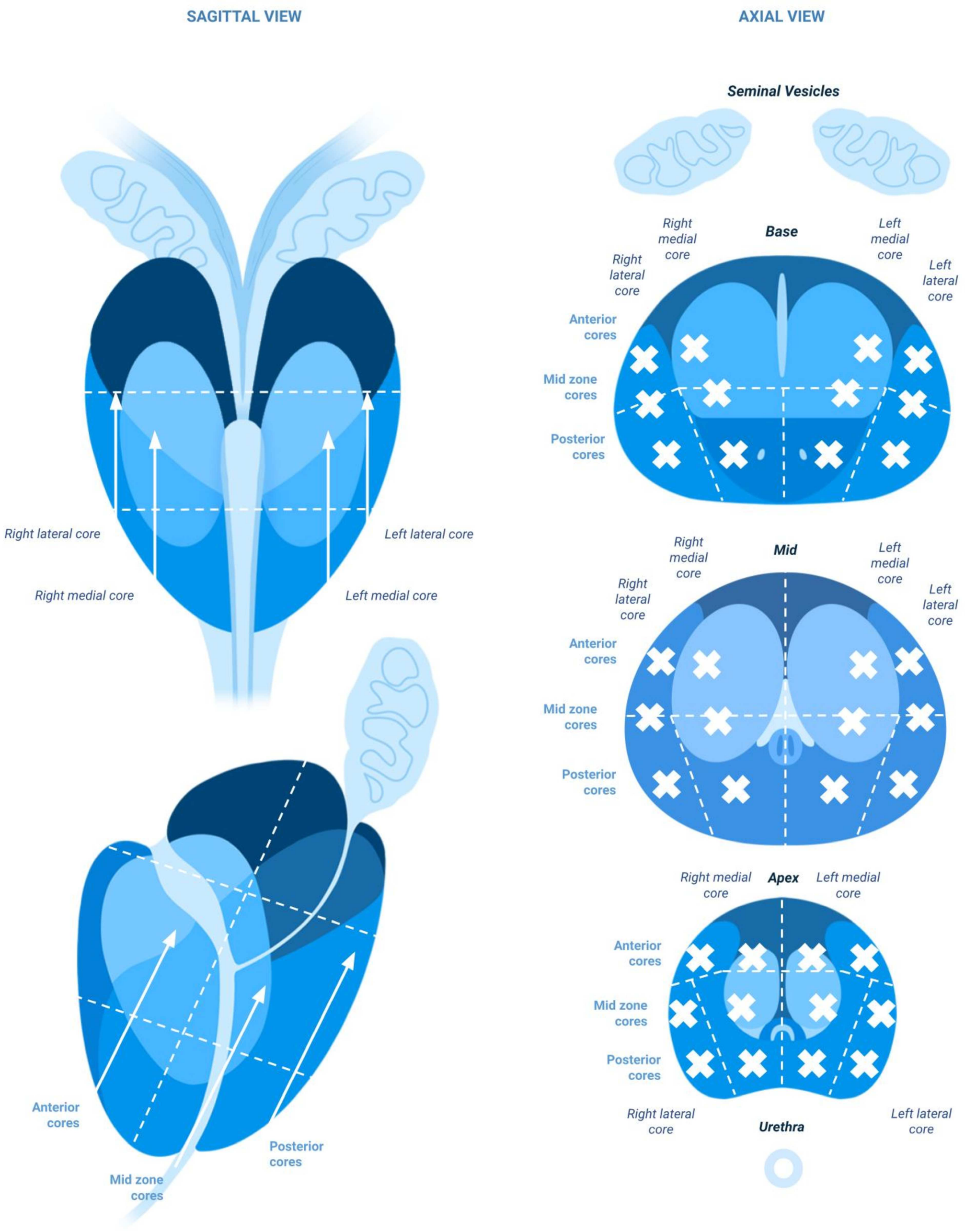

For each pair of biopsies – one core is more lateral, one core is more medial. From anterior—posterior, there are 3 planned rows of biopsies – anterior, mid zone, posterior. Avoid biopsy around the urethra.

Designed by Aqua Asif

Suspicious MRI lesion scenarios

Figure 2. Examples of how to perform transperineal biopsies in patients with an MRI Target (scores 3, 4 or 5 on *either* Likert or PI-RADS v2.1 scoring systems).

2A. Single lesion example.

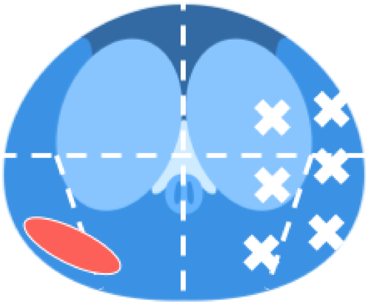

This is a single lesion in the right mid-gland peripheral zone posteromedially (PZ pm) and posterolaterally (PZ pl).

- Take **4 targeted biopsies** from the Target.
- Then take **6 peripheral zone focused biopsies** from the **contralateral** side.
- Do **not** resample the targeted biopsy side.

2B. Bilateral peripheral zone lesions example.

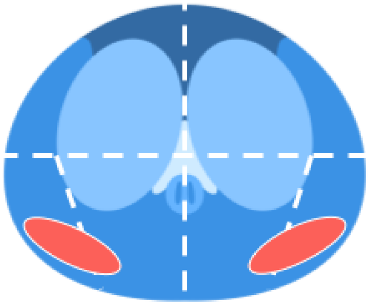

There are **two lesions**: one in right mid-gland, peripheral zone posteromedially and posterolaterally (PZ pm and PZ pl); one in left mid-gland, peripheral zone posteromedially and posterolaterally (PZ pm and PZ pl).

- Take **4 targeted biopsies** from ***each*** Target – *i.e.* **8 targeted biopsies** in **total**.
- **Do not take any systematic biopsies** as targeted biopsies are taken from both sides of the prostate.

Designed by Aqua Asif

PRIME

2C. Lesion crossing midline example.

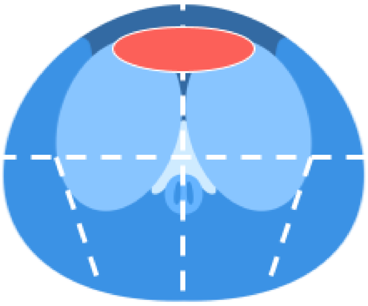

This is one lesion crossing the midline in the mid-gland, anterior fibromuscular stroma.

- Take **4 targeted biopsies** from the Target.
- **Do not take any systematic biopsies** as targeted biopsies are taken from both sides of the prostate.

2D. Bilateral diffuse change on Likert scoring example.

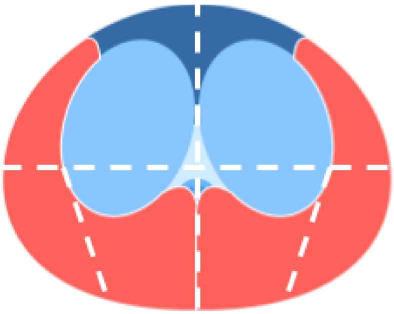

In the circumstance where on Likert scoring, the peripheral zone gives diffuse change, scoring 3 out of 5, arbitrarily **treat each peripheral zone** as a **different Target**.

- Take **4 targeted biopsies** from *each half* of the peripheral zone – *i.e.* **8 biopsies** in total.
- **Do not take any systematic biopsies** as targeted biopsies are taken from both sides of the prostate.

Designed by Aqua Asif

2E. A new lesion is revealed on DCE sequence example.

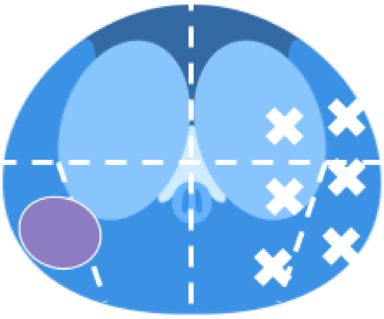

This is one lesion in the right mid-gland, peripheral zone posterolaterally. This **new Target** was specifically *not* suspicious (scored 1 or 2 on both Likert and PI-RADS v2.1) on bpMRI sequences (T2W and DWI). However, when the contrast sequence is revealed, the lesion appears to be suspicious (scored 3, 4 or 5 on Likert) on the dynamic contrast-enhanced (DCE) sequence than on the bpMRI.

- Thus, label the **new lesion** as a **DCE-Target**.
- Take **4 targeted biopsies** from **DCE-Target-1**.
- Then take **6 peripheral zone focused biopsies** from the **contralateral** side of the prostate.
- Do **not** resample the targeted biopsy side.

2F. A new **part** of an *existing* lesion is revealed on DCE sequence example.

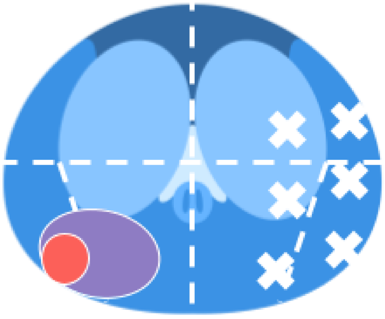

There are two lesions in this example. **Target 1** (**red**) was suspicious on **both** bpMRI and mpMRI. It is in the right mid-gland, peripheral zone, posterolaterally (PZ pl). It scores Likert 4 and PI-RADS v2.1 4.

However, when the contrast sequence is revealed, this lesion appears to be larger on the DCE sequence than on bpMRI. The part of the lesion that is **non-overlapping** would **not** have been

Designed by Aqua Asif

target biopsied if bpMRI alone was used. Thus, the second lesion (the non-overlapping part, **purple**) is called **DCE Target 1**. It is in the right mid-gland, peripheral zone, posteromedially (PZ pm).

Thus, the instructions are as follows in this instance:

- Take **4 targeted biopsies** from **Target 1**.
- Take **4 targeted biopsies** from **DCE Target 1**.
- Take **6 peripheral zone focused biopsies** from the **contralateral** side of the prostate.
- Do **not** resample the targeted biopsy side.

Systematic Transrectal Biopsy Schema

Figures 3 and **4** depict examples of how to perform the systematic biopsy in the **absence** of an MRI lesion and in the **presence** of MRI lesions, respectively.

Non-suspicious MRI but a PSA Density of ≥ 0.15ng/mL/mL scenario

In patients with a **non-suspicious MRI but a PSA Density of ≥ 0.15ng/mL/mL**, 12-core systematic biopsy should be performed (**Figure 3**).

If performing biopsies transrectally, systematic biopsy cores should be taken from:

- Right base (2 cores)
- Right mid gland (2 cores)
- Right apex (2 cores)
- Left base (2 cores)
- Left mid gland (2 cores)
- Left apex (2 cores)

Systematic biopsy cores should be stored and labelled in a way that their location can be identified when the pathologist reports the result.

The 12 systematic biopsies **should be focused on the peripheral zone.** The urethra should be avoided.

Designed by Aqua Asif

Figure 3. The transrectal biopsy schema for men with a **non-suspicious MRI** (scores 1 or 2 on both Likert and PI-RADS v2.1 scoring systems) *but* a PSA Density of ≥0.15ng/mL/mL, undergoing 12-core systematic biopsy.

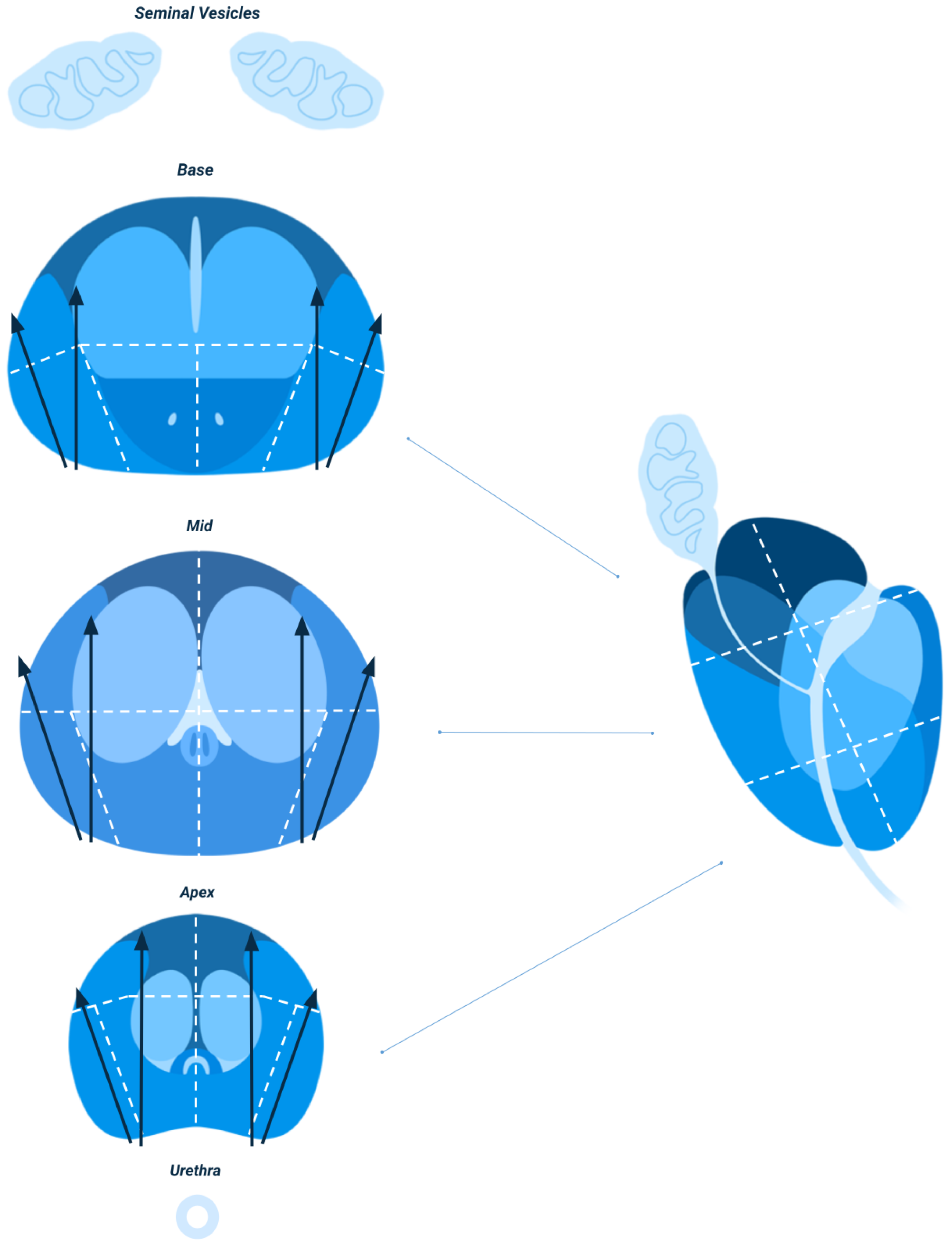

Designed by Aqua Asif

Suspicious MRI lesion scenarios

Figure 4. Examples of how to perform transrectal biopsies in patients with an MRI Target (scores 3, 4 or 5 on *either* Likert or PI-RADS v2.1 scoring systems).

4A. Single lesion example.

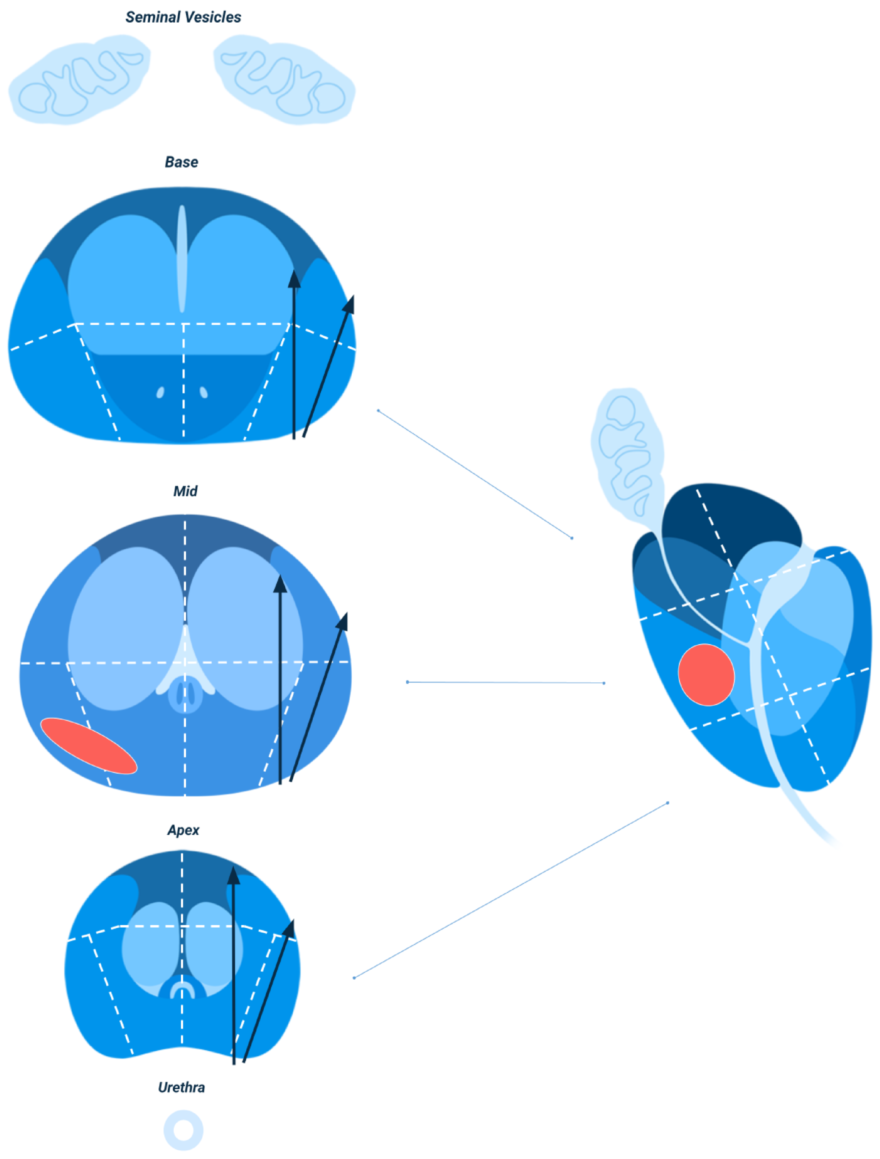

This is a single lesion in the right mid-gland peripheral zone posteromedially (PZ pm) and posterolaterally (PZ pl).

- Take **4 targeted biopsies** from the Target.
- Then take **6 peripheral zone focused biopsies** from the **contralateral** side.

Designed by Aqua Asif

- Do **not** resample the targeted biopsy side.

4B. Bilateral peripheral zone lesions example.

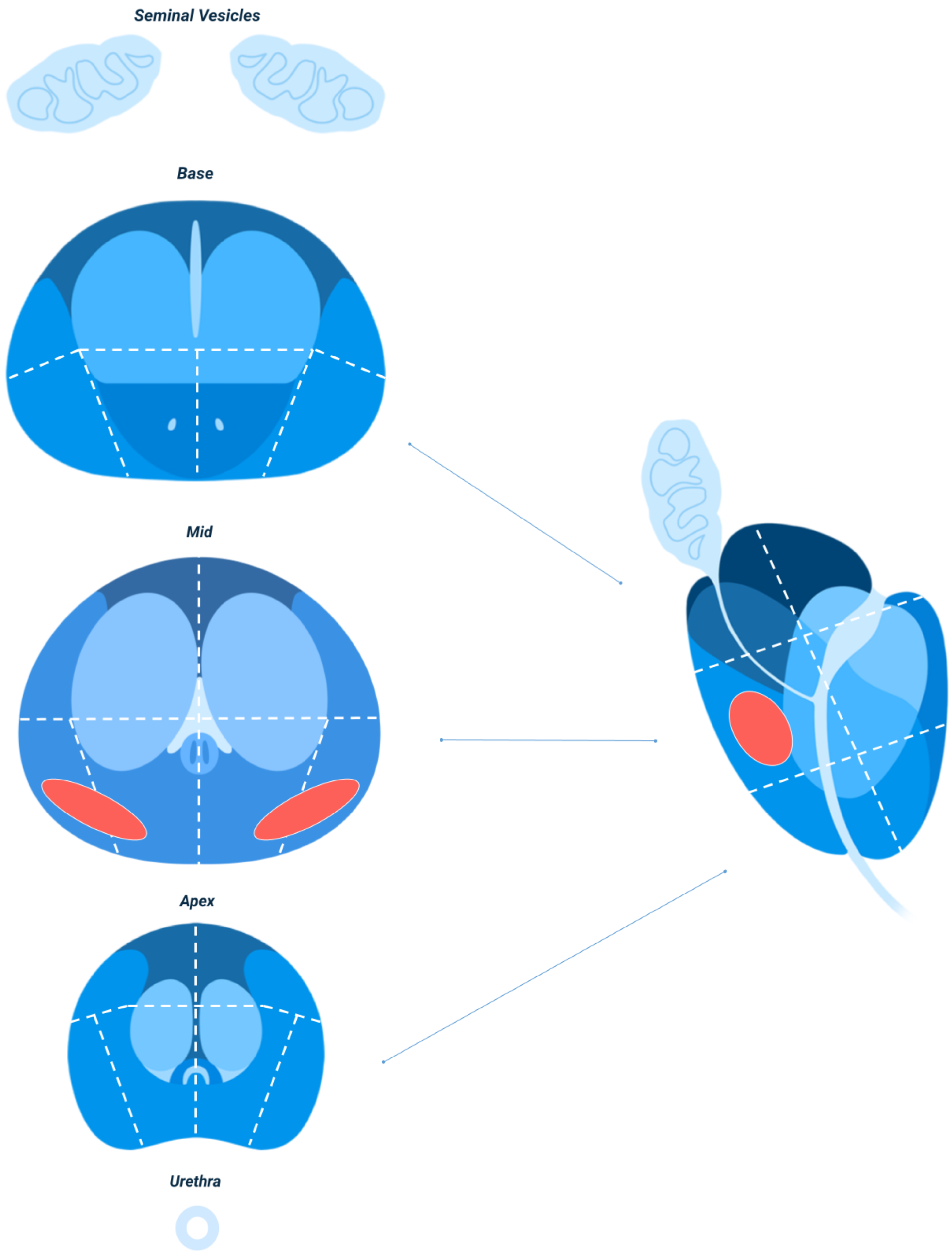

There are **two lesions**: one in right mid-gland, peripheral zone posteromedially and posterolaterally (PZ pm and PZ pl); one in left mid-gland, peripheral zone posteromedially and posterolaterally (PZ pm and PZ pl).

- Take **4 targeted biopsies** from ***each*** Target – *i.e.* **8 targeted biopsies** in **total**.

Designed by Aqua Asif

- **Do not take any systematic biopsies** as targeted biopsies are taken from both sides of the prostate.

4C. Lesion crossing midline example.

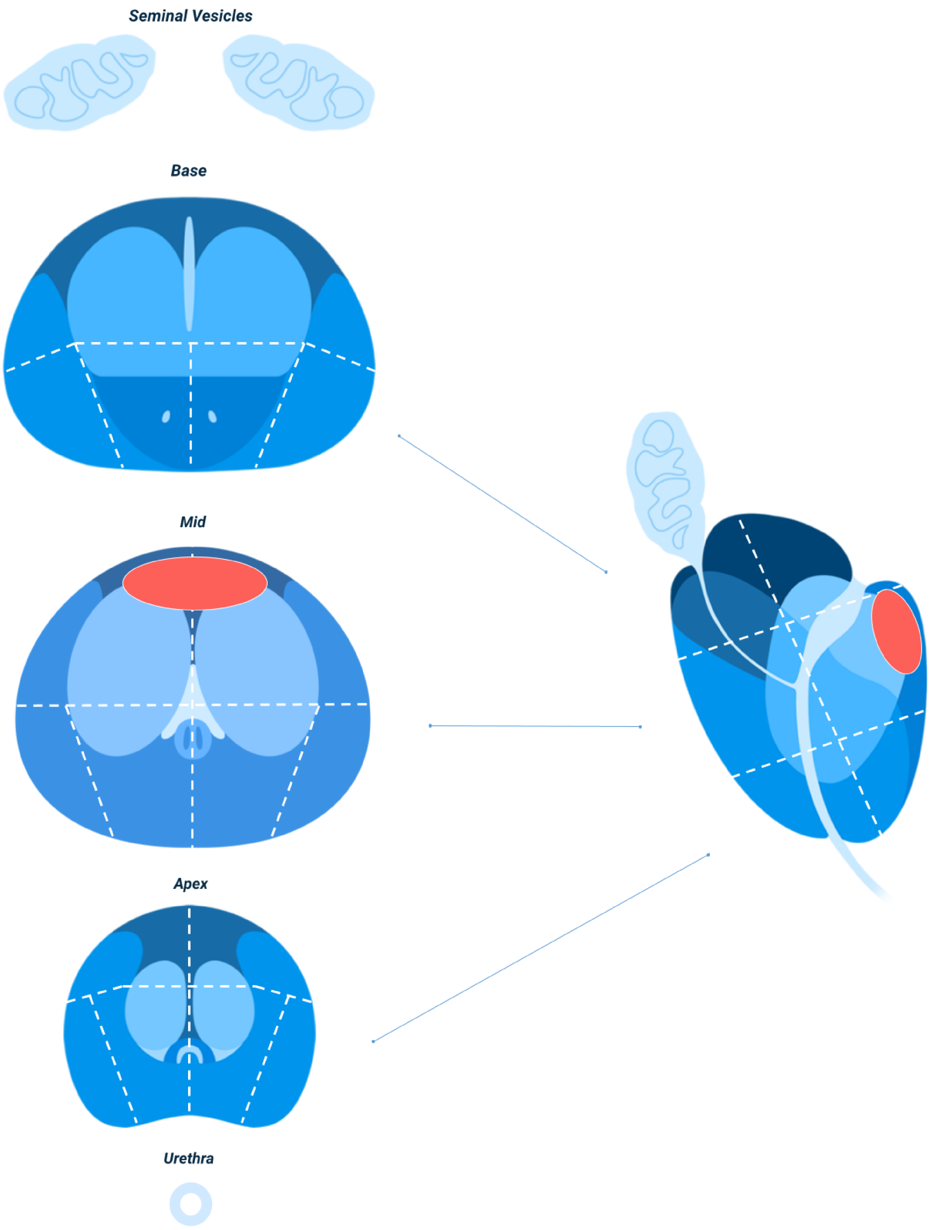

This is one lesion crossing the midline in the mid-gland, anterior fibromuscular stroma.

- Take **4 targeted biopsies** from the Target.

Designed by Aqua Asif

- **Do not take any systematic biopsies** as targeted biopsies are taken from both sides of the prostate.

4D. Bilateral diffuse change on Likert scoring example.

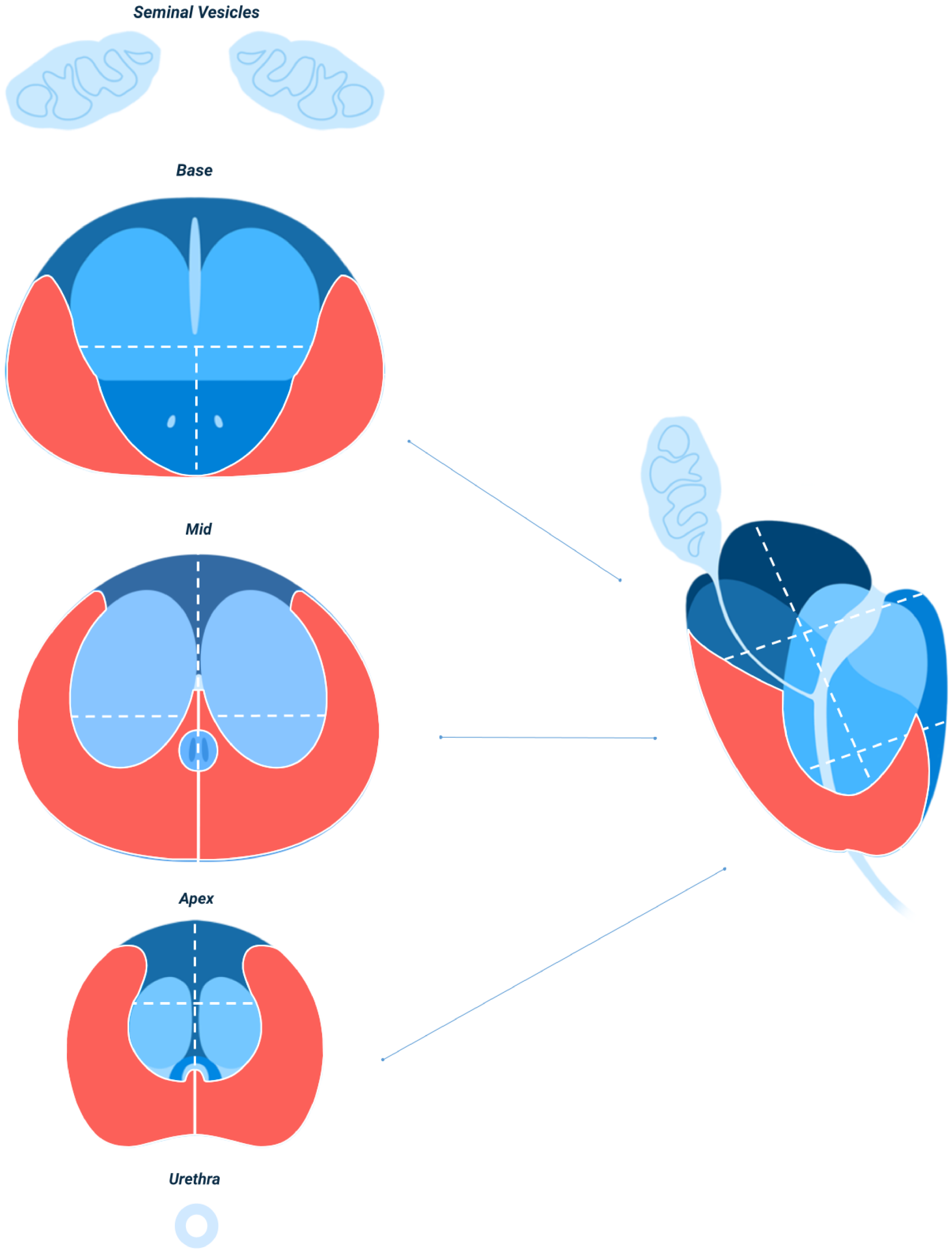

In the circumstance where on Likert scoring, the peripheral zone gives diffuse change, scoring 3 out of 5, arbitrarily **treat each peripheral zone** as a **different Target**.

Designed by Aqua Asif

- Take **4 targeted biopsies** from *each half* of the peripheral zone – *i.e.* **8 biopsies** in total.
- **Do not take any systematic biopsies** as targeted biopsies are taken from both sides of the prostate.

4E. A new lesion is revealed on DCE sequence example.

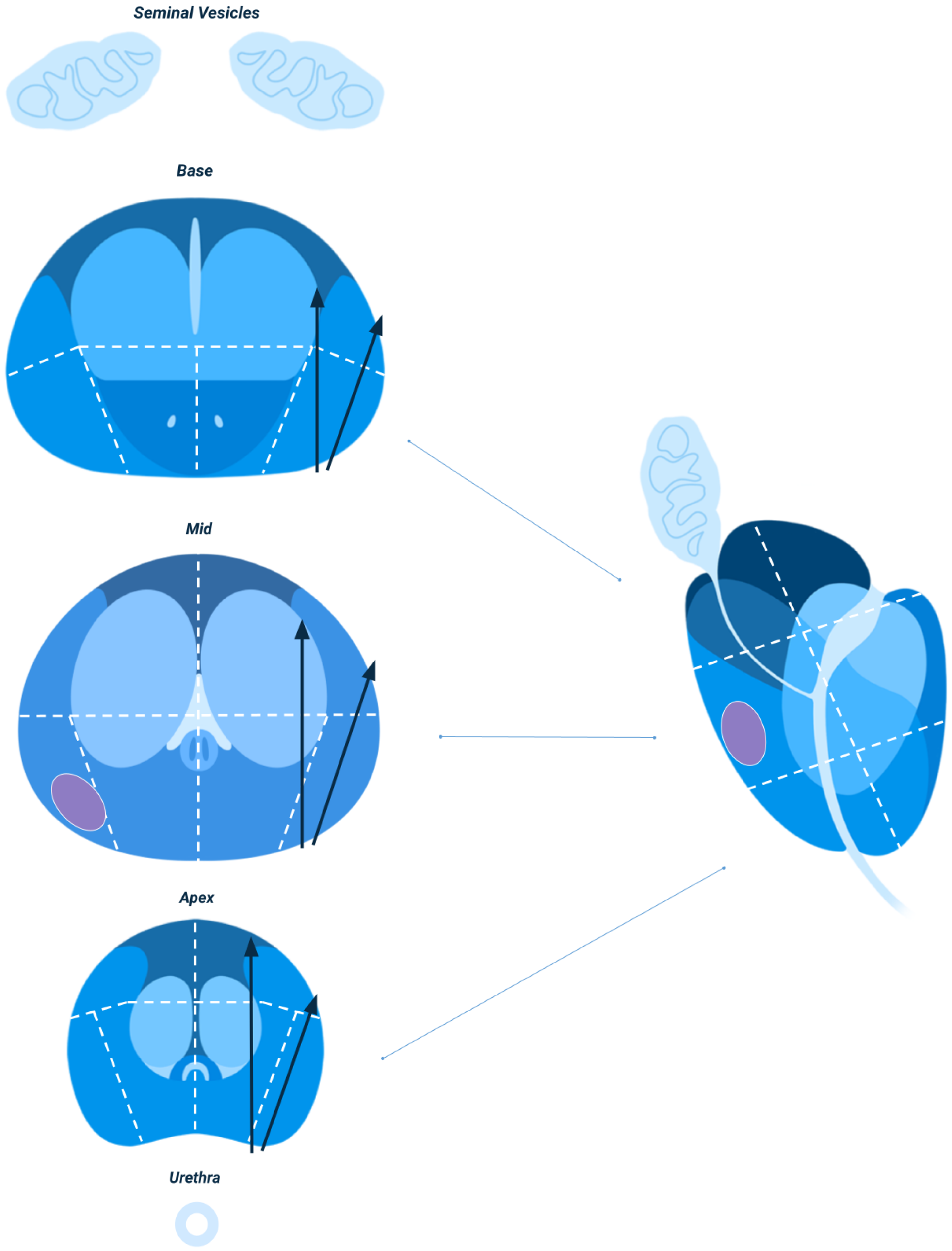

This is one lesion in the right mid-gland, peripheral zone posterolaterally. This **new Target** was specifically *not* suspicious (scored 1 or 2 on both Likert and PI-RADS v2.1) on bpMRI sequences (T2W and DWI). However, when the contrast sequence is revealed, the lesion

Designed by Aqua Asif

appears to be suspicious (scored 3, 4 or 5 on Likert) on the dynamic contrast-enhanced (DCE) sequence than on the bpMRI.

- Thus, label the **new lesion** as a **DCE-Target**.
- Take **4 targeted biopsies** from **DCE-Target-1**.
- Then take **6 peripheral zone focused biopsies** from the **contralateral** side of the prostate.
- Do **not** resample the targeted biopsy side.

4F. A new **part** of an *existing* lesion is revealed on DCE sequence example.

There are two lesions in this example. **Target 1** (**red**) was suspicious on **both** bpMRI and mpMRI. It is in the right mid-gland, peripheral zone, posterolaterally (PZ pl). It scores Likert 4 and PI-RADS v2.1 4.

However, when the contrast sequence is revealed, this lesion appears to be larger on the DCE sequence than on bpMRI. The part of the lesion that is **non-overlapping** would **not** have been target biopsied if bpMRI alone was used. Thus, the second lesion (the non-overlapping part, **purple**) is called **DCE Target 1**. It is in the right mid-gland, peripheral zone, posteromedially (PZ pm).

Thus, the instructions are as follows in this instance:

- Take **4 targeted biopsies** from **Target 1**.
- Take **4 targeted biopsies** from **DCE Target 1**.
- Take **6 peripheral zone focused biopsies** from the **contralateral** side of the prostate.
- Do **not** resample the targeted biopsy side.

Designed by Aqua Asif

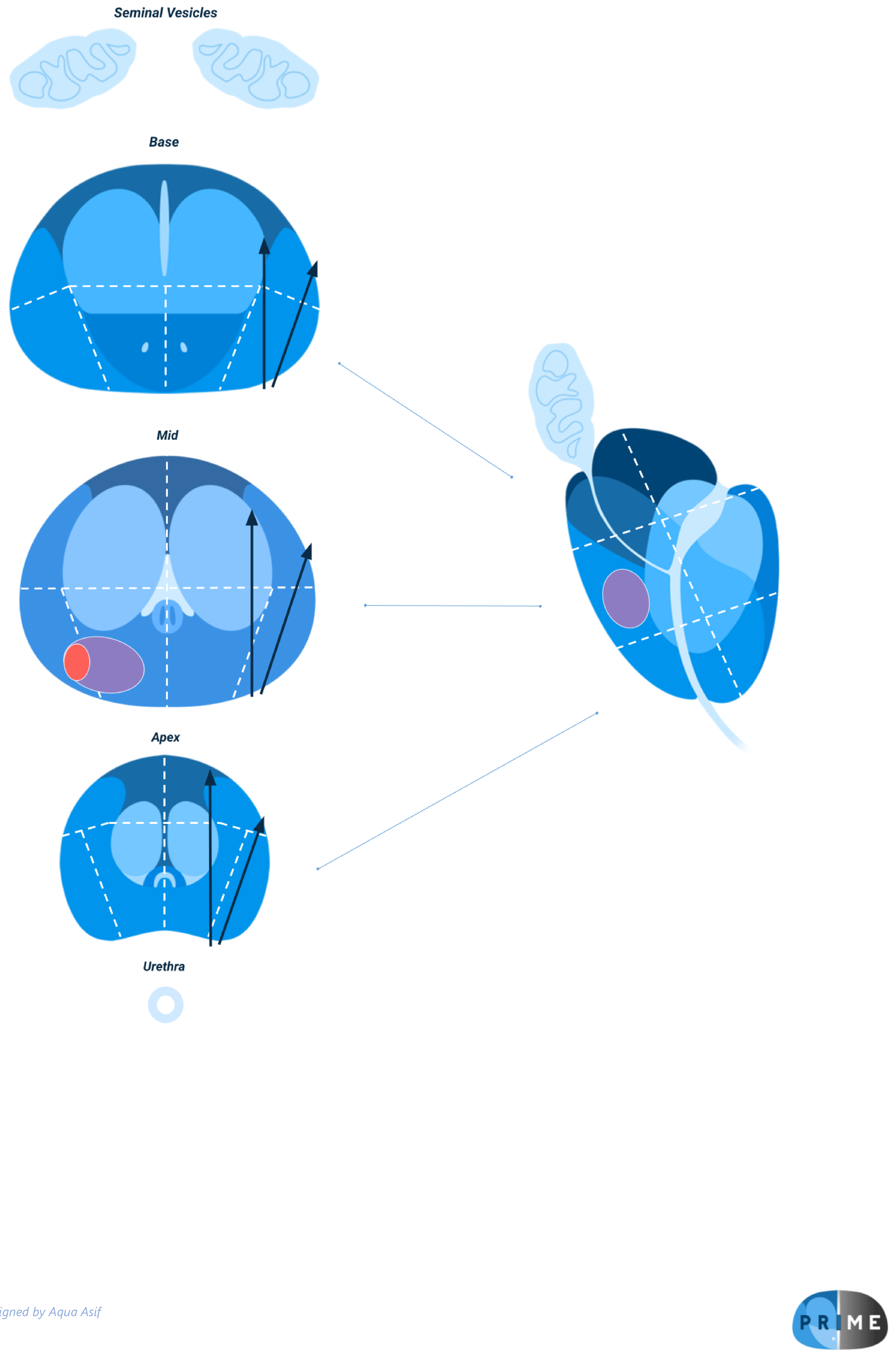
Seminal Vesicles
Base
Mid
Apex
Urethra
Designed by Aqua Asif

Summary Biopsy Guidelines

Number of MRI targets	Location of MRI targets in prostate	Number of MRI-targeted biopsy cores	Number of contralateral systematic cores	Total number of biopsy cores
**0**	If PSA Density is < 0.15ng/ml/ml			0
**0**	If PSA Density is ≥ 0.15ng/ml/ml, then 12 systematic biopsy cores are taken (6 from each side)			12
**1**	Unilateral	4	6	10
**2**	Unilateral	8	6	14
**3**	Unilateral	12	6	18
**4–8**	Unilateral	16–32	6	22–38
**1**	Bilateral (*e.g.* crossing midline)	4	0	4
**2**	Bilateral	8	0	8
**3**	Bilateral	12	0	12
**4–8**	Bilateral	16–32	0	16–32

Designed by Aqua Asif
