# Supplementary material for: Comparing biparametric to multiparametric MRI in the diagnosis of clinically significant prostate cancer in biopsy-naive men (PRIME): a prospective, international, multicentre, non-inferiority within-patient, diagnostic yield trial protocol
Source: BMJ Open. 2023 Apr 5;13(4):e070280. doi: 10.1136/bmjopen-2022-070280 (PMC10083803; doi:10.1136/bmjopen-2022-070280)
Supplement: Supplementary data [file bmjopen-2022-070280supp003.pdf]

Please present on local headed paper

REC Number:
IRAS Number: 282789

Subject Identification: ____________________
Study Number ;__________________________

CONSENT FORM

Title of Project: PRostate **I**maging using **M**RI +/- contrast **E**nhancement (PRIME)

Name of Researcher:

Please initial box

1. I confirm that I have read and understand the information sheet dated................... (version...........) for the above study. I have had the opportunity to consider the information, ask questions and have had these answered satisfactorily. ☐

2. I understand that my participation is voluntary and that I am free to withdraw at any time without giving any reason, without my medical care or legal rights being affected. ☐

3. I understand that relevant sections of my medical notes and data collected during the study, may be looked at by individuals from the sponsor of the trial (University College London), responsible persons authorised by the sponsor, from regulatory authorities, from the NHS Trust and from PRIME study researchers who may be outside of my local centre, where it is relevant to my taking part in this research. I give permission for these individuals to have access to my records. ☐

4. I agree to my GP being informed of my participation in the study. ☐

5. I give my permission for the PRIME research team at my local centre to hold identifiable information such as my name, address, date of birth, email address, mobile phone number, NHS number or other applicable hospital identifier. I understand this may be used to collect longer term healthcare information on me from national records, such as the Office for National Statistics, NHS Digital, Public Health England, and other applicable NHS information systems, or other relevant national databases. This data may be linked to my data from the PRIME study in future research. ☐

Asif A, *et al. BMJ Open* 2023; 13:e070280. doi: 10.1136/bmjopen-2022-070280

6. I give permission to be contacted for further information in the future. This may include requests to complete quality of life questionnaires or for ascertaining future health status, if required. ☐

7. I give permission for my samples to be sent to UCL by courier for quality control assessments. ☐

8. I give permission for my anonymized data to be used for teaching and educational purposes for healthcare professionals. ☐

9. I give my permission for my anonymized data to be shared with affiliated researchers and commercial partners who are approved by the PRIME study team for future research if deemed suitable by the PRIME Chief Investigator ☐

10. I give my permission to be approached for other studies in the future that may be relevant to me, and for my study data collected in PRIME to be used for this purpose. ☐

11. I agree to take part in the above study and to complete study procedures outlined in the patient information sheet provided. ☐

All boxes above must be initialed for consent to be valid

Name of Participant	Date	Signature
Name of Person taking consent	Date	Signature

When completed: 1 for participant; 1 (original) for researcher site file; 1 to be kept in medical notes.

IRAS Reference Number 282789
PRIME Consent Form Version 2.0 Dated 27APR2021

PLACE HOSPITAL LETTER HEAD ON FIRST PAGE ONLY.

Affix patient sticker / details here

Version 3.0 8 June 2021

This is the Patient Information Sheet for a Health Research Study called PRIME

Study Short Title: Prostate Imaging using MRI +/- contrast Enhancement

Study acronym: **PRIME**

Chief Investigator: Mr Veeru Kasivisvanathan

UCL Reference number: 135819

REC Reference number: 21/WM/0091

IRAS Number: 282789

We would like to invite you to take part in our research study. Before you decide we would like you to understand why you are being invited, why the research is being done and what it would involve for you. One of our team will go through the information sheet with you and answer any questions you have. Talk to others about the study if you wish.

Part 1 tells you the purpose of this study and what will happen to you if you take part. Part 2 gives you more detailed information about the conduct of the study.

Ask us if there is anything that is not clear. Please take as much time as you need to consider the study.

Asif A, *et al. BMJ Open* 2023; 13:e070280. doi: 10.1136/bmjopen-2022-070280

Part 1

1. Why have I been invited?

You are being invited because you may require further investigation of your prostate with an MRI scan and / or a prostate biopsy. You have not been diagnosed with cancer but an MRI and / or a biopsy may be required to establish whether you do or do not have cancer. The clinical Urology team that you have been referred to has informed us that you may be eligible for this study.

2. What is the purpose of the study?

The standard way of diagnosing prostate cancer is to carry out a multiparametric prostate MRI scan and prostate biopsy. This type of MRI scan normally involves an injection of contrast into one of your veins.

Another type of MRI scan (biparametric) can be performed that does not require contrast, and therefore does not require the insertion of a cannula. We currently do not know for certain whether using this type of MRI will allow us to detect the same, more or less prostate cancer than if we use the standard (multiparametric) type of MRI. Current evidence supports the idea that using biparametric MRI may detect a similar amount of cancer to when it is not used but one advantage is it may allow a man to have a scan without contrast.

The main purpose of this study is to assess if biparametric MRI can provide similar information to multiparametric MRI. You will undergo a multiparametric MRI with a contrast injection, which is the typical method used for investigating the prostate for the presence of cancer. The doctor reviewing your scan will be asked to review the MRI scan in a particular order so that they can tell whether the additional information given by the contrast injection helps identifies prostate cancer.

If there is a suspicious area in the prostate on the MRI, a few biopsies can be directed at where the suspicious area is thought to be, also using an ultrasound probe in the back passage. If there is no suspicious area on the MRI and if you at low risk of harbouring cancer, which occurs in about 30% of men, then no biopsy will be taken at all.

3. Do I have to take part?

It is up to you to decide to join the study. We will describe the study and go through this information sheet. If you agree to take part, we will then ask you to sign a consent form. You are free to withdraw at any time without giving a reason. This will not affect the standard of care you receive.

4. What are the benefits to me of taking part in this study?

The healthcare team carrying out the tests in the study are experienced in carrying out and interpreting these tests. The research team will ensure your tests are carried out as quickly as possible and will be a point of contact for you should you have any concerns or questions.

The information we get from this study will help improve the diagnosis of prostate cancer for men in the future.

5. What type of study is this?

This is a study evaluating the accuracy of diagnostic tests. In this trial, you will have the same investigation (multiparametric MRI) as your hospital normally does to investigate the prostate, but the doctor interpreting your scan will be asked to report this in a particular order. The full information will be available to the doctors as it would normally be available if you were not taking part in the study.

You will be required to attend a screening visit with a member of the research team who will spend around 40 minutes explaining what is involved in the study and making sure you are

Asif A, *et al. BMJ Open* 2023; 13:e070280. doi: 10.1136/bmjopen-2022-070280

eligible for the study. Where possible, all study visits that do not require a journey to the hospital will be performed remotely (e.g. over the phone or video call).

6. What will happen to me if I take part?

After you have attended the screening visit, if you are eligible to take part in the study, you will asked to visit the hospital 2-3 times in total, which is the same as if you were not taking part in the study. After you consent to participating in the study, you will be asked to complete two short questionnaires which will ask about any symptoms related to your prostate that you may be having. These are questionnaires that are typically used as part of routine care. You would only undergo tests that you would normally have as part of routine care if you were not taking part in the study.

If you have not already had a prostate MRI, you will have one within a few weeks after the screening visit. The MRI takes about 40 minutes. Alternatively, it is possible that you are approached for the study after you have had your prostate MRI.

If you have an MRI with a high enough suspicion (MRI Score 3, 4 or 5) you will be booked for a biopsy following the MRI. If the MRI is non-suspicious but you are at high risk of having cancer because of a blood test result, (called your prostate specific antigen density) you will also undergo a prostate biopsy. If you do not need a biopsy (if your MRI is non-suspicious and your prostate specific antigen density is low) then you do not need to undergo a biopsy and we will explain this to you once your MRI results is available.

The biopsy procedure itself takes about 40 minutes and is typically carried out under local or general anaesthetic. Prostate biopsies, which take very small samples of prostate tissue, are taken from the prostate gland and sent to the lab to determine whether there is cancer there or not. If there is a suspicious area on the MRI scan, the MRI information will be used to influence where the biopsies are taken from. Software may be used to transfer additional information from the original MRI onto the screen when the biopsies are taken. In some centres, this would be exactly what you would normally get, and there would be no difference to standard of care. In other centres, their usual practice may be slightly different to this, and you may be required to have a few extra or fewer biopsies than what is typical in your usual centre. After the procedure, we then wait for the results and discuss treatment options with you in clinic at approximately 2-3 weeks after the biopsies.

Please note that the above time frames are suggested time frames and depending on clinical workload within the hospital, the time frame may be shorter or longer. This would be no different than if you were not part of the study.

Being involved in the study does not limit subsequent tests or treatment you may receive. If you do undergo further tests or treatment after the study is complete we may check the results of these on your records. We use the research data we have gathered from your involvement in the study to help us determine how good the diagnostic tests you have had are. We will work with other research teams to do this. We also ask your permission to use research data for teaching and education of other healthcare professionals. After completing the study, we also ask your permission to check your health through national databases. We may also contact you for further information in the future. This may include requests to complete quality of life questionnaires or for ascertaining future health status. All information which is collected about you during the course of the research will be kept strictly confidential, and any information about you which leaves the hospital will have your name and address removed so that you cannot be recognised. Please see Part 2 for further information on this.

7. What data will be collected and use of data

We will need to use information from your medical records for this research project. Your hospital will hold personal identifiable data on you. This information will include information such as age, PSA level, family history of medical conditions such as prostate cancer and examination findings. We allow the PRIME research team at your local site to hold

identifiable data on you, which will be for 10 years. Longer term data that may be requested from you include information on whether or not you have had further investigations or treatment for prostate problems and what the outcomes of those were as well as quality of life assessments. Non-identifiable data will be stored in the MARVIN database and the database will be transferred and stored at UCL within UCL's data safe haven. You will be given a subject number and a subject identifier, and this will be used on all your study records. The code for this number will be known to the investigators at your site so that the link between your name and the data we hold on the study database is not completely broken. Any paperwork for the study will be kept in locked cupboards, staff access to these cupboards is strictly controlled.

In general, UCL, as a university and a study sponsor, uses personally-identifiable information to conduct research to improve health, care and services. As a publicly-funded organisation, we have to ensure that it is in the public interest when we use personally-identifiable information from people who have agreed to take part in research. This means that when you agree to take part in a research study, we will use your data in the ways needed to conduct and analyse the research study. Your rights to access, change or move your information are limited, as we need to manage your information in specific ways in order for the research to be reliable and accurate. If you withdraw from the study, we will keep the information about you that we have already obtained. To safeguard your rights, we will use the minimum personally-identifiable information possible.

Health and care research should serve the public interest, which means that we have to demonstrate that our research serves the interests of society as a whole. We do this by following the UK Policy Framework for Health and Social Care Research.

All data is managed in line with the Data Protection Act (2018) & General Data Protection Regulations (GDPR).

If you wish to raise a complaint on how we have handled your personal data, you can contact our Data Protection Officer who will investigate the matter. If you are not satisfied with our response or believe we are processing your personal data in a way that is not lawful you can complain to the Information Commissioner's Office (ICO).

UCL Data Protection Officer can be contacted on data-protection@ucl.ac.uk

8. What will I have to do?

You should attend your screening visit and if eligible for the study, await contact from the hospital for further dates of investigations. Unless otherwise advised by a doctor you should carry on with your normal activities and medication. Sometimes before a biopsy your doctor will prescribe you antibiotics and may ask you to stop blood-thinning medications.

You should undergo the necessary tests and biopsy procedures that you are advised to have by your doctor.

You should attend your follow up clinic appointment where we discuss your results. Treatment options will be discussed with you at the results clinic. In total you will typically be required to attend the hospital 2-3 times.

9. What are the alternatives for diagnosis?

An MRI scan and biopsies of the prostate if required are the standard ways in which prostate cancer is diagnosed.

10. What are the possible disadvantages and risks of taking part?

Asif A, *et al. BMJ Open* 2023; 13:e070280. doi: 10.1136/bmjopen-2022-070280

Being involved in the study is unlikely to expose you to additional risk than if you were not involved in the study but underwent the normal procedures for men referred for further investigation of prostate disease.

Risks of prostate biopsy include:

- Temporary discomfort in the back passage (most men)
- Blood in the urine – up to 2 weeks (most men)
- Blood in the semen – up to 3 months (most men)
- Blood in the back passage – up to 1 week (most men)
- Infection in the blood stream – 1-4 out of 100 men
- Urinary tract infection – 4 out of 100 men
- Urinary retention – 1 out of 100 men
- Adverse reaction to antibiotics – less than 1 in 100 men

Risks of MRI include:

- Discomfort from cannulation
- Allergic reaction:
    - Mild reaction e.g. rash, itching – less than 1 in 250 men
    - Moderate reaction e.g. nausea, omitting – less than 1 in 2000 men
    - Severe reaction e.g. breathing problems – less than 1 in 10000 men

In some centres, you would receive exactly what you would normally get outside of the study. In other centres, their usual practice may be slightly different to this, and you may be required to have a few extra or fewer biopsies than what is typical in your usual centre. However, there is no evidence that a few extra or fewer biopsies within the proposed study would result in additional adverse effects for you.

Before participating you should consider if this will affect any insurance you have and seek advice if necessary.

11. What should you do if you experience any problems during the study?

Though the risk is very low, if you do experience any possible signs of infection after biopsies (fevers and feeling generally unwell) then you should urgently go to your nearest accident and emergency department which is open 24 hours a day. If you are not able to pass urine you should urgently go to your nearest accident and emergency. If you are unsure about what to do or have any questions please call 0207 679 9092 between 9am and 5pm and a member of our team may be able to offer you advice or direct you to someone who can offer you advice.

If you experience any other untoward complication or need to see a doctor we would like to know about this so please let us know on the above number as soon as possible after the complication. For any emergencies at any time or if you are unable to contact a member of the research team, please attend your local accident and emergency for an assessment.

12. What happens when the research study stops?

Once the results of the MRI and, if required, biopsy are available you will be called to clinic to discuss them. Once a treatment decision is made, most men in the study will complete the study and your normal clinical team will continue to look after your care. Being part of the study does not prevent you from undergoing any further diagnostic test or treatment that your clinician would normally recommend.

13. What if there is a problem?

Any complaint about the way you have been dealt with during the clinical study or any possible harm you might suffer will be addressed. The detailed information concerning this is given in Part 2 of this information sheet. If you have any concerns or complaints you should contact a member of the research team in the first instance.

Asif A, *et al. BMJ Open* 2023; 13:e070280. doi: 10.1136/bmjopen-2022-070280

14. Will my taking part in the study be kept confidential?
Yes. We will follow ethical and legal practice and all information about you will be handled in confidence. The details are included in Part 2.

15. Will any costs I incur in travelling to study visits be reimbursed to me?
Reasonable transport costs that you incur to get to additional study visits (if any further visits are necessary) that are above what you would normally need if you were not part of the study may be reimbursed. Please contact your local study nurse or doctor or the Study Coordinator (details below) for further information on claiming.

16. Contact Details
If you have any further questions or need any further information please do no hesitate to contact the research team.

or the **Chief Investigator:**
Mr Veeru Kasivisvanathan MBBS BSc FRCS MSc PGCert PhD
[redacted]

This completes Part 1 of the Information Sheet.

If the information in Part 1 has interested you and you are considering participation, please read the additional information in Part 2 before making any decision.

Asif A, *et al. BMJ Open* 2023; 13:e070280. doi: 10.1136/bmjopen-2022-070280

Part 2

17. What if relevant new information becomes available?

Sometimes we get new information about the procedures being studied. If this happens and we feel it is important to your participation in the study, we will tell you about it and discuss whether you want to or should continue in the study. If you decide not to carry on, we will make arrangements for your care to continue. If you decide to continue in the study, we may ask you to sign an updated consent form. You can also find out if there is any new relevant information by visiting www.ncita.org.uk.

18. What will happen if I don't want to carry on with the study?

You can withdraw from the study at any point and it will not affect the care that you are given. We will use information collected about you up until your withdrawal. Kindly keep in contact with us to let us know your progress.

19. What if there is a problem?

If you have a concern about any aspect of this study, you should ask to speak to your research team who will do their best to answer your questions, please see point number 24. You can also contact the Chief Investigators on the number or address given earlier in this document. If you wish to complain by other means or have any concerns about any aspect of the way you have been approached or treated by members of staff or about any side effects (adverse events) you may have experienced due to your participation in the clinical study, the normal National Health Service complaints mechanisms are available to you. You can contact the hospital Patient Advice and Liaison Service (PALS). Your local PALS team can be contacted at the following number:

Local team to insert contact details of local PALS office here:

You can also contact NHS helpline at 111 which will be able to give you the number of your local PALS office if you are concerned.

Every care will be taken in the course of this clinical study. However in the unlikely event that you are injured by taking part, compensation may be available.

If you suspect that the injury is the result of the Sponsor's (University College London) or the hospital's negligence, then you may be able to claim compensation. After discussing with your study doctor, please make the claim in writing to Mr Veeru Kasivisvanathan who is the Chief Investigator for the clinical study and is based at University College London. The Chief Investigator will then pass the claim to the Sponsor's Insurers, via the Sponsor's office. You may have to bear the costs of the legal action initially, and you should consult a lawyer about this.

20. Will my taking part in this study be kept confidential?

If you consent to take part in this study, the records obtained while you are in this study as well as related health records will remain strictly confidential at all times. The information will be held securely on paper and electronically at your treating hospital under the provisions of the Data Protection Act 2018 and the General Data Protection Regulations 2018. The information will be made available to persons in the clinical and research teams treating you. Your name and personal details will not be passed to anyone else outside the clinical team, research team or the Sponsor, who is not involved in the study. No additional samples will be taken specially for research in this study. All The research team may verify results of tests carried out at your local hospital (for example MRI results or prostate biopsy results) by transferring and analysing a small number of samples collected to UCL. samples and information collected will be de-identified to you prior to transfer to UCL, so only non-identifiable data will be transferred to UCL. This includes some pathology glass slides, which will be reviewed at Dr Alex Freeman's laboratory at University College London (UCL), for quality control. Slides sent to UCL will be

Asif A, et al. BMJ Open 2023; 13:e070280. doi: 10.1136/bmjopen-2022-070280

not have your name assigned. Samples will be sent using one of UCL's preferred couriers, for both pick up and return.

Any data stored by the research team outside of your treating hospital will be kept at a secure location and will not contain information that can directly identify you. You will be allocated a study number, which will be used as a code to identify you on all study forms and data. The information will be linked to you so that if we did need to identify you for your safety or to clarify some information we would be able to by using a unique key, which will be known only to your local hospital team.

Your records will be available to people authorised to work on the study but may also need to be made available to people authorised by the Sponsor, which is the organisation responsible for ensuring that the study is carried out correctly. By signing the consent form, you agree to this access for the current study and any further research that may be conducted in relation to it, even if you withdraw from the current study. All will have a duty of confidentiality to you as a research participant.

If you withdraw consent from further study treatment, your data and samples will remain on file and will be included in the final study analysis.

In line with the regulations, at the end of the study your data will be securely archived for 20 years. Arrangements for confidential destruction will then be made.

Anonymised data collected during the study may be transferred for the purpose of processing or analysis to approved associated researchers and commercial partners within/outside the European Economic Area. The Sponsor of the study will take all reasonable steps to protect your privacy.

In the future we may publish our findings from the study in scientific journals, but you will not be identifiable in any publications.

21. Will my GP be informed of my involvement?
Because this study is not being carried out by your GP, we would like to inform them of your participation. If you agree to take part and agree to us contacting your GP, we will give him or her details of the study and inform them that you have chosen to participate in it. You will not be able to participate in this study if you do not give us this permission to inform your GP.

22. What will happen to the results of the research study?
The results of the study will be available after it finishes and will usually be published online in a medical journal and presented at a scientific conference, they will also be posted to. The data will be anonymous and it will not be possible to identify you in any report or publication. Sometimes the data may be used to teach other healthcare professionals how to treat patients in a similar position to you.

Should you wish to see the results, or the publication, please ask your study doctor or see the trial website on https://www.ucl.ac.uk/surgery/research/research-department-targeted-intervention/prime-trial-information, or the clinical trials units website www.ncita.org.uk.

23. Who is organising and funding the research?
The governance sponsor is University College London. The study is funded by Prostate Cancer UK, the European Association of Urology Research Foundation, the UK National Institute for Health Research via an Academic Clinical Lectureship to Dr Veeru Kasivisvanathan and the UK National Cancer Imaging Translational Accelerator.

24. Who has reviewed the study?

All research in the NHS is looked at by an independent group of people, called a Research Ethics Committee, to protect your interests. This study has been reviewed and given favorable opinion by National Research Ethics Service Committee _West Midlands - Black Country Research Ethics Committee. Patients and members of the public have also reviewed the study documents to ensure they are appropriate and well written.

25. Further information

You are encouraged to ask any questions you wish, before, during or after your investigations. If you have any questions about the study, please speak to your study nurse or doctor on the numbers specified below, who will be able to provide you with up to date information about the procedures involved. If you wish to read the research on which this study is based, please ask your study nurse or doctor.

Site Study staff contact details:

Principal Investigator (site) details:

Alternatively, if you or your relatives have any questions about this study you may wish to contact one of the following organisations that are independent of the hospital at which you are being treated:

Prostate Cancer UK – 0800 082 1616 - http://prostatecanceruk.org

Macmillan Cancer Support - 0808 808 0000 – http://www.macmillan.org.uk

If you decide you would like to take part then please read and sign the consent form. You will be given a copy of this information sheet and the consent form to keep. A copy of the consent form will be filed in your patient notes, one will be filed with the study records and one may be sent to the Research Sponsor.

You can have more time to think this over if you are at all unsure.

Thank you for taking the time to read this information sheet and to consider this study.

Asif A, *et al. BMJ Open* 2023; 13:e070280. doi: 10.1136/bmjopen-2022-070280
