# Supplementary material for: Comparing biparametric to multiparametric MRI in the diagnosis of clinically significant prostate cancer in biopsy-naive men (PRIME): a prospective, international, multicentre, non-inferiority within-patient, diagnostic yield trial protocol
Source: BMJ Open. 2023 Apr 5;13(4):e070280. doi: 10.1136/bmjopen-2022-070280 (PMC10083803; doi:10.1136/bmjopen-2022-070280)
Supplement: Author's manuscript [file bmjopen-2022-070280.draft_revisions.pdf]

BMJ Open

BMJ Open is committed to open peer review. As part of this commitment we make the peer review history of every article we publish publicly available.

When an article is published we post the peer reviewers' comments and the authors' responses online. We also post the versions of the paper that were used during peer review. These are the versions that the peer review comments apply to.

The versions of the paper that follow are the versions that were submitted during the peer review process. They are not the versions of record or the final published versions. They should not be cited or distributed as the published version of this manuscript.

BMJ Open is an open access journal and the full, final, typeset and author-corrected version of record of the manuscript is available on our site with no access controls, subscription charges or pay-per-view fees (http://bmjopen.bmj.com).

If you have any questions on BMJ Open's open peer review process please email info.bmjopen@bmj.com

BMJ Open

Comparing biparametric to multiparametric MRI in the diagnosis of clinically significant prostate cancer in biopsy-naive men (PRIME): a prospective, international, multicentre, non-inferiority, within-patient, diagnostic yield trial protocol

Journal:	*BMJ Open*
Manuscript ID	bmjopen-2022-070280
Article Type:	Protocol
Date Submitted by the Author:	19-Nov-2022

Complete List of Authors:	Asif, Aqua; University College London, Division of Surgery and Interventional Science<br>Nathan, Arjun; University College London, Division of Surgery and Interventional Science; Royal College of Surgeons of England, Clinical Effectiveness Unit<br>Ng, Alexander; University College London, Division of Surgery and Interventional Science<br>Khetrapal, Pramit; University College London, Division of Surgery and Interventional Science; Whipps Cross University Hospital, Department of Urology<br>Chan, Vinson Wai-Shun; University College London, Division of Surgery and Interventional Science<br>Giganti, Francesco; University College London, Division of Surgery and Interventional Science; University College London Hospitals NHS Foundation Trust, Department of Radiology<br>Allen, Clare; University College London, Division of Surgery and Interventional Science; University College London Hospitals NHS Foundation Trust, Department of Radiology<br>Freeman, Alex; University College London Hospitals NHS Foundation Trust, Department of Histopathology<br>Punwani, Shonit; University College London Hospitals NHS Foundation Trust, Department of Radiology; University College London, Centre for Medical Imaging<br>Lorgelly, Paula; University College London, Institute of Epidemiology and Health Care; The University of Auckland, School of Population Health<br>Clarke, Caroline; University College London, Research Department of Primary Care and Population Health<br>Brew-Graves, Chris; University College London, National Cancer Imaging Translational Accelerator<br>Muirhead, Nicola; University College London, National Cancer Imaging Translational Accelerator<br>Emberton, Mark; University College London, Division of Surgery and Interventional Science; University College London Hospitals NHS Foundation Trust, Department of Urology<br>Agarwal, Ridhi; University of Birmingham, Test Evaluation Research Group, Institute of Applied Health Research; University Hospitals Birmingham NHS Foundation Trust, NIHR Birmingham Biomedical

Research Centre
Takwoingi, Yemisi; University of Birmingham, Test Evaluation Research Group, Institute of Applied Health Research; University Hospitals Birmingham NHS Foundation Trust, NIHR Birmingham Biomedical Research Centre
Deeks, Jonathan; University of Birmingham, Test Evaluation Research Group, Institute of Applied Health Research; University Hospitals Birmingham NHS Foundation Trust, NIHR Birmingham Biomedical Research Centre
Moore, Caroline; University College London, Division of Surgery and Interventional Science; University College London Hospitals NHS Foundation Trust, Department of Urology
Kasivisvanathan, Veeru; University College London, Division of Surgery and Interventional Science; University College London Hospitals NHS Foundation Trust, Department of Urology
Trial Group, PRIME; University College London

Keywords: Prostate disease < UROLOGY, Magnetic resonance imaging < RADIOLOGY & IMAGING, RADIOLOGY & IMAGING, Protocols & guidelines < HEALTH SERVICES ADMINISTRATION & MANAGEMENT, Urological tumours < ONCOLOGY

SCHOLARONE™
Manuscripts

Comparing biparametric to multiparametric MRI in the diagnosis of clinically significant prostate cancer in biopsy-naive men (PRIME): a prospective, international, multicentre, non-inferiority within-patient, diagnostic yield trial protocol

Authors:

Aqua Asif[1†]
Arjun Nathan[1,2†]
Alexander Ng[1]
Pramit Khetrapal[1,3]
Vinson Wai-Shun Chan[1]
Francesco Giganti[1,4]
Clare Allen[1,4]
Alex Freeman[5]
Shonit Punwani[4,6]
Paula Lorgelly[7,8]
Caroline S Clarke[9]
Chris Brew-Graves[10]
Nicola Muirhead[10]
Mark Emberton[1,11]
Ridhi Agarwal[12,13]
Yemisi Takwoingi[12,13]
Jon Deeks[12,13]
Caroline M Moore[1,11]
Veeru Kasivisvanathan[1,11]
On behalf of the PRIME Trial Group*

[†]Joint first authors have made equal contribution to this study

*PubMed Indexed Collaborators (PRIME Trial Group)
John Wilkinson, Marimo Rossiter, Réka Novotta, Philip Ryan, Alex Kirkham, Louise Dickinson, Tushar Narain, Hazel McBain, Antonette Andrews, Daniel Margolis, Valeria Panebianco, Antti Ranniko, Peter Albertsen, Lance Mynderse, Adam Froemming, Naoki Takahasi, Tristan Barrett, Raphaele Renard Penna, Vibeke Løgager, Lars Boesen, Sangeet Ghai, Lars Budäus, Valeria Panebianco, Enrique Gómez Gómez, Jeremy Grummet, Tho Pham, Aiman Haider, Jing Yi Jessica Weng, Anders Bjartell, Wim Witjes, Christien Caris, Joke Van Egmond.

Affiliations:

1. Division of Surgery and Interventional Science, University College London, London, United Kingdom
2. Clinical Effectiveness Unit, The Royal College of Surgeons of England, United Kingdom
3. Department of Urology, Whipps Cross Hospital, Barts Health NHS Trust, London, United Kingdom
4. Department of Radiology, University College London Hospital NHS Foundation Trust, London, United Kingdom
5. Department of Pathology, University College London Hospitals NHS Foundations Trust, London, United Kingdom
6. Centre for Medical Imaging, University College London, London, United Kingdom
7. Institute of Epidemiology and Health Care, University College London, London, United Kingdom
8. School of Population Health, The University of Auckland, Auckland, 1023, New Zealand
9. Research Department of Primary Care and Population Health, University College London, London, United Kingdom

For peer review only - http://bmjopen.bmj.com/site/about/guidelines.xhtml

10. National Cancer Imaging Translational Accelerator, Division of Medicine, University College London, London, United Kingdom
11. Department of Urology, University College London Hospital NHS Foundation Trust, London, United Kingdom
12. Test Evaluation Research Group, Institute of Applied Health Research, University of Birmingham, Birmingham, United Kingdom
13. NIHR Birmingham Biomedical Research Centre, University Hospitals Birmingham NHS Foundation Trust, University of Birmingham, Birmingham, United Kingdom

Corresponding Author:
Veeru Kasivisvanathan (veeru.kasi@ucl.ac.uk)
@veerukasi
Division of Surgery and Interventional Science, University College London, London, United Kingdom

Keywords:
prostate cancer, diagnostic, MRI, prostate, biopsy

Current word count: 3658
Figure limit: No limits on figures or tables
Figure count: 1
Box count: 2
Table count: 5
Supplementary appendix count: 3

ARTICLE SUMMARY

STRENGTHS AND LIMITATIONS

- *Strength:* PRIME is a pragmatic, prospective, international, multicentre trial being carried out in a range of different healthcare settings
- *Strength:* Its within-patient design allows patients to act as their own control, improving the efficiency and power of the trial compared to a randomised study
- *Strength:* Its within-patient design allows the impact of the dynamic contrast enhanced sequences on staging decisions and treatment eligibility to be made at an individual patient level.
- *Strength:* PRIME will be one of the first trials to quality control the performance of sites' dynamic contrast enhanced sequences prior to their involvement in the trial
- *Limitation:* as both biparametric and multiparametric targeted biopsies are carried out in the same patient it is possible for the performance of one technique to influence the other

For peer review only - http://bmjopen.bmj.com/site/about/guidelines.xhtml

ABSTRACT

Introduction

Prostate MRI is a well-established tool for the diagnostic work-up for men with suspected prostate cancer. Current recommendations advocate the use of multiparametric MRI (mpMRI), which is comprised of three sequences: T2-weighted (T2W), diffusion-weighted (DWI), and dynamic contrast enhanced (DCE). Prior studies suggest that a biparametric MRI approach (bpMRI), omitting the DCE sequences, may not compromise clinically significant cancer detection, though there are limitations to these studies, and it is not known how this may affect treatment eligibility. A bpMRI approach will reduce scanning time, may be more cost effective and at a population level, allow more men to gain access to an MRI than a mpMRI approach.

Methods

PRIME is a prospective, international, multicentre within-patient diagnostic yield trial, assessing whether bpMRI is non-inferior to mpMRI in the diagnosis of clinically significant prostate cancer. Patients will undergo the full mpMRI scan. Radiologists will be blinded to the dynamic contrast enhanced sequence (DCE) and will initially report the MRI using only the bpMRI (T2W and DWI) sequences. They will then be unblinded to the DCE sequence and will then re-report the MRI using the mpMRI sequences (T2W, DWI and DCE). Men with suspicious lesion(s) on either bpMRI or mpMRI will undergo prostate biopsy. The main inclusion criteria is men with suspected prostate cancer, with a serum PSA of ≤ 20 ng/mL and no prior prostate biopsy. The primary outcome is the proportion of men with clinically significant prostate cancer detected (Gleason ≥ 3+4 or Gleason Grade Group ≥ 2). A sample size of at least 500 patients is required. Key secondary outcomes include the proportion of clinically insignificant prostate cancer detected and treatment decision.

Ethics and Dissemination Ethical approval was obtained from the National Research Ethics Committee West Midlands, Nottingham 21/WM/0091. Results of this trial will be disseminated through peer-reviewed publications. Participants and relevant patient support groups will be informed about the results of the trial.

Registration details NCT04571840

STUDY TITLE

Long Title: A trial assessing whether biparametric MRI is non-inferior to multiparametric MRI in the diagnosis of clinically significant prostate cancer
Short Title: Prostate Imaging Using MRI +/- Contrast Enhancement
Trial Acronym: PRIME

For peer review only - http://bmjopen.bmj.com/site/about/guidelines.xhtml

This protocol was written according to SPIRIT guidelines (1).

INTRODUCTION

Magnetic Resonance Imaging (MRI) is widely established as the gold standard diagnostic imaging modality for detecting clinically significant prostate cancer (PCa) (2). The landmark PRECISION study established the benefit of detecting clinically significant prostate cancer using MRI and targeting biopsies based on MRI findings (3). The National Prostate Cancer Audit data from England showed that only one in two men receive an MRI before biopsy in 2019 (5).

Current recommendations for the use of MRI for detection of PCa focus on the use of multiparametric MRI (mpMRI) (3, 4). mpMRI consists of three sequences: T2-weighted (T2W), diffusion-weighted (DWI) and dynamic contrast enhanced (DCE) sequences. On the DCE sequences, cancer-suspicious areas can demonstrate early wash-in, enhancement and rapid wash-out of contrast (6–8). The DCE sequences involve administering gadolinium contrast via an intravenous cannula. Therefore, it increases scanning time and healthcare costs compared to a bpMRI approach where only T2W and DWI are used. It is also known that the gadolinium contrast material accumulates in the basal ganglia, though its clinical relevance is not fully understood (9).

Removing the DCE sequences from the MRI protocol has been suggested as a potential avenue to improve the cost-effectiveness of using MRI in the diagnostic pathway for PCa (10, 11) and the reduced scanning time required may improve the number of men with suspected prostate cancer accessing an MRI scan. Using bpMRI has demonstrated similar detection rates of PCa as mpMRI but current evidence is limited primarily to retrospective, single-centre studies (11, 12). The few prospective studies have not been typically robustly designed to evaluate the role of DCE in prostate cancer detection (12, 13).

The PRIME trial aims to assess whether bp-MRI is non-inferior to mpMRI in the detection of clinically significant prostate cancer. PRIME may redefine the standard of care diagnostic test for men with suspicion of PCa and allow many more patients who need access to an MRI to get one.

Objectives

The primary objective is to compare the detection of clinically significant PCa (Gleason ≥ 3+4 or Gleason Grade Group ≥ 2) using bpMRI ± targeted biopsy with mpMRI ± targeted biopsy.

Key secondary objectives include:

- To compare the proportion of men who have clinically insignificant PCa (Gleason 3+3 or Gleason Grade Group 1) detected for bpMRI versus mpMRI
- To compare the proportion of men with non-suspicious MRIs for bpMRI versus mpMRI
- To compare the proportion of men with indeterminately-scored MRI as reported by bpMRI and mpMRI
- To compare the proportion of men with MRIs of adequate standard for reporting for bpMRI versus mpMRI
- To compare the diagnostic test performance for bpMRI versus mpMRI
- To compare radiological staging for bpMRI versus mpMRI
- To compare treatment eligibility decisions for bpMRI when compared with mpMRI
- To compare diagnostic performance of bpMRI and mpMRI when using the Likert scoring system in comparison to the PI-RADS v2.1 scoring system
- To compare the cost effectiveness of bpMRI when compared to mpMRI

For peer review only - http://bmjopen.bmj.com/site/about/guidelines.xhtml

Trial Design

The PRIME trial is designed as a prospective, multicentre, within-patient, diagnostic yield trial, assessing whether bpMRI is non-inferior to mpMRI for the diagnosis of clinically significant PCa in biopsy-naive men. A paired cohort design was chosen rather than a randomised trial design for the following reasons:

- More efficient design (sevenfold lower sample size required) with equivalent quality of evidence in the setting of a diagnostic study
- Patients act as their own control due to the within-patient design, thus allowing us to draw conclusions regarding the value of DCE sequences on a per patient level
- Allows for the evaluation of the impact of contrast on staging decisions and treatment eligibility decisions at an individual patient level
- Patients get the benefit of having targeted biopsies based on the information from both bpMRI and mpMRI information, whereas with a randomised study, patients randomised to one technique will be denied of potential benefit of the other

METHODS AND ANALYSIS

Trial Setting

We expect centres who perform prostate cancer diagnostics and management from the following countries to take part: Argentina, Australia, Belgium, Brazil, Canada, Denmark, France, Finland, Germany, Italy, Netherlands, Singapore, Spain, UK and USA. Sites will be required to undergo a period of quality control prior to including patients to ensure minimum acceptable standards for the conduct of mpMRI, reporting and targeted biopsy.

Eligibility Criteria

Patients will be considered eligible for registration into this trial if they fulfil all of the inclusion criteria and none of the exclusion criteria (**Box 1**).

Box 1 Eligibility criteria

Inclusion criteria

1. Men at least 18 years of age referred with clinical suspicion of prostate cancer
2. Serum PSA ≤ 20 ng/mL
3. Fit to undergo all procedures listed in protocol
4. Able to provide written informed consent

Exclusion criteria

1. Prior prostate biopsy
2. Prior treatment for prostate cancer
3. Prior prostate MRI on a previous encounter
4. Contraindication to MRI (*e.g.* claustrophobia, some pacemakers)
5. Contraindication to prostate biopsy
6. Unfit to undergo any procedures listed in protocol

Interventions

MRI Conduct

MRI will be conducted with 1.5T or 3.0T with pelvic-phased array coils, with or without endorectal coils. The PRECISION study quality control highlighted that the image quality of the DCE sequences was the most variable sequence across sites (3). Therefore, to give DCE a reasonable chance of demonstrating whether it has value, MRI scanner approval for use in the study will be made on the basis of central review of MRI images, utilising the Prostate Imaging Quality (PI-QUAL) scoring system (14). In brief, PI-QUAL is a 5-point Likert scoring system, where 1 indicates no sequences are of diagnostic quality and 5 implies that each sequence individually is of optimal diagnostic quality. The objective criteria used to determine PI-QUAL scores are derived from internationally published

For peer review only - http://bmjopen.bmj.com/site/about/guidelines.xhtml

minimum standards for MRI conduct (15). If necessary, sites will be given recommendations to improve image quality and will be re-evaluated after optimisation for participation in the study.

Reporting of MRI

Patients will undergo (or will have undergone) standard of care mpMRI as per their local protocol. The radiologists participating in this trial will be blinded to the DCE sequences and will report the MRI using only the biparametric (T2W and DWI) sequences in Report 1. After reporting the bpMRI, the same radiologist will be unblinded to the DCE sequences and will re-report the MRI using the mpMRI sequences (T2W, DWI and DCE) in Report 2 (**Figure 1**).

The MRIs and lesions are scored on a 1–5 scale of suspicion for the likelihood that clinically significant PCa is present, with 5 representing the greatest score of suspicion. Both the traditional Likert and PI-RADS v2.1 scoring systems will be used to identify any suspicious lesions in the prostate. Suspicious areas (Likert or PI-RADS v2.1 ≥3) on either bpMRI or mpMRI will undergo targeted biopsy of the prostate, with cores from contrast-enhanced suspicious areas stored separately.

A summary of the rules for reporting MRI scans in the PRIME trial is in **Box 2.** Please see **Supplementary Appendix 1** for our model reporting proformas, which radiologists participating in the PRIME trial will use to label lesions.

Non-suspicious bpMRI and mpMRI

Men whose MRIs do not show suspicious areas on bpMRI and mpMRI (i.e. scored 1 or 2 on Likert and PI-RADS v2.1) will be stratified by PSA density. Men with PSA density <0.15ng/mL/mL will not undergo biopsy and men with PSA density ≥0.15ng/mL/mL will undergo systematic biopsy.

Box 2 Summary of MRI reporting rules

Report 1 (biparametric MRI: T2W and DWI)

1. The radiologist reporting this will be blinded to DCE, with verification of this via an independent person or an automated system (MIM by MIM Software Inc)
2. The radiologist should then interpret the bpMRI sequences **blinded** to DCE
3. Up to 4 suspicious areas (score ≥ 3 out of 5 on the Likert or PI-RADS v2.1 scoring system) can be marked on Report 1 – if there are more, the four **most suspicious** should only be marked on
4. The location of the suspicious areas should be labelled according to the PI-RADS v2.1 41-sector diagram
5. Once Report 1 (biparametric MRI: T2W and DWI) has been done, this **cannot be altered** after looking at the DCE

Report 2 (multiparametric MRI: T2W, DWI and DCE)

1. The same radiologist must report both Report 1 and Report 2
2. They will then be **unblinded** to the DCE sequence
3. The radiologist should now complete Report 2
4. The location of the suspicious areas should be similarly labelled according to the PI-RADS v2.1 41-sector diagram as above
5. On Report 2, each of the existing lesions are additionally labelled as one of:

bpMRI positive, mpMRI positive

This occurs when a lesion scores 3, 4 or 5 on both bpMRI and mpMRI based on ***either*** Likert or PI-RADS v2.1 scoring systems

bpMRI positive, mpMRI negative

For peer review only - http://bmjopen.bmj.com/site/about/guidelines.xhtml

This occurs when a lesion scores 3, 4 or 5 on bpMRI on ***either*** Likert or PI-RADS v2.1 scoring systems, but also scores a 1 or 2 on mpMRI on both Likert and PI-RADS v2.1 scoring systems

bpMRI negative, mpMRI positive

There are two instances in which **new targets** may be labelled and drawn onto Report 2:

1. **New** lesions scoring 3, 4 or 5, identified by DCE not previously identified on bpMRI should be marked on as new lesions as **DCE Targets** and **bpMRI negative, mpMRI positive**

2. Lesions that appear **larger** on DCE should be treated as 2 separate targets
    - One target depicts the completely overlapping segment from Report 1 (bpMRI positive, mpMRI positive)
    - The non-overlapping part which would otherwise not be sampled should be labelled as a **new target** (bpMRI **negative**, mpMRI positive). This is a subjective decision by the radiologist. A typical example of when to declare this as a separate target is if the non-overlapping part enters an adjacent sector on the PI-RADS v2.1 sector diagram

A biopsy plan is recommended by the radiologist thereafter for the biopsy operator to follow.

Prostate Biopsy Procedures

MRI-Targeted Biopsy

Men will undergo MRI-targeted biopsy if either their bpMRI or mpMRI identifies a suspicious lesion which scores ≥3 on either Likert or PI-RADS v2.1. Four targeted cores will be taken per suspicious lesion, and these should be stored and labelled in separate containers to ensure cancer detection from separate suspicious areas are ascertained.

Systematic Biopsy

Systematic biopsies should be performed after targeted biopsies, with 6 cores taken from the contralateral side of the MRI lesion, focused on sampling the peripheral zone of the prostate. If there are bilateral MRI lesions or midline lesions, then no systematic biopsies are necessary.

Please see **Supplementary Appendix 2** for a detailed overview of how our biopsies will be conducted.

Prostate Histopathology

Both the Gleason score and the Gleason Grade Group will be reported for the overall biopsy and for each individual target lesion.

Pre-Trial Assessments

For all patients, patient referral would follow clinical suspicion of PCa (*e.g.* raised PSA or abnormal digital rectal examination). To confirm a patient's eligibility, screening will be undertaken. Patients can enter the trial either before or after they have had their mpMRI scan. If patients are recruited after an mpMRI scan has been carried out, this will only be permitted if the MRI has not been seen by any clinician.

Registration Procedures

Following consent and confirmation of eligibility, trial processes can commence. The patient will be registered and assigned a trial ID using a central online database (Marvin by XClinical).

Intervention Procedures

For peer review only - http://bmjopen.bmj.com/site/about/guidelines.xhtml

All patients will undergo a full mpMRI scan. This includes T2W, DWI and DCE sequences.

Follow-Up for Results

If bpMRI and mpMRI is non-suspicious and PSA density is <0.15 ng/mL/mL, the patient will be counselled for standard of care follow-up – typically consisting of PSA surveillance. If a decision for prostate biopsy or other tests is made, these results will be recorded after which the participant completes the trial.

Multidisciplinary Team Decision-Making for Treatment Eligibility

Treatment decisions will be per local standard of care, based on pathology results and will be recorded. Subsequently, a virtual multidisciplinary team meeting will be conducted and treatment eligibility decisions blinded to the DCE will be recorded. Once a decision has been recorded, the clinicians will be unblinded to the DCE sequence and the impact that this information makes on treatment eligibility will be evaluated.

MRI and Pathology Quality Control

Quality control will be carried out at the end of the study by the PRIME chief radiologists reviewing the original MRIs, who will assess the MRI quality and re-report the MRI blinded to the study reports. Anonymised pathology slides from a proportion of patients may also be reviewed by central pathologists. Any slides assessed outside of the originating site will be returned to the original site after quality control. Quality control results will be reported but will not influence patient management or outcomes.

Cost-Effectiveness

A within-trial incremental cost-effectiveness analysis will be conducted to calculate the difference in mean cost per diagnosis of clinically significant prostate cancer if a strategy of bpMRI were adopted instead of the current mpMRI standard of care, over a time horizon of 30 days. The difference in cost of avoiding each additional case of clinically insignificant prostate cancer diagnosed may also be calculated.

Costs of procedures will be estimated by applying standard unit costs to resource use data captured within the trial plus other procedures that would be offered to patients in either pathway. Estimates of the resources used (procedures, tests, radiotherapy, chemotherapy, other therapies, surveillance visits, and other care events) on the two treatment pathways will be obtained for the theoretical bpMRI cohort using decisions made initially by the MDT with information from the bpMRI scan and any biopsies as a result of that scan; and estimates of the treatment pathway resources used in the theoretical mpMRI cohort will be made subsequently by the MDT on viewing additional information from the mpMRI scan and any further biopsies performed as a result of that scan. This thought experiment is required due to the ethical requirement to use all available information, *i.e.* not just bpMRI and biopsies or just mpMRI and biopsies, when making the actual treatment decision with the patient.

The analysis perspective will be that of the NHS and personal social services. Standard unit costs (*e.g.* NHS Reference Costs) will be supplemented by unit cost data from the participating trial sites. A microcosting study to provide this information will be undertaken in a small number of sites as part of the trial to investigate the resources employed to deliver bpMRI and mpMRI scans. This information will allow us to understand the MRI booking system, consumption of consumables, and staff time as related to delivering bpMRI and mpMRI scans.

Depending on the within-trial cost-effectiveness findings, consideration will be given to extending this analysis using decision analytic modelling to estimate quality-adjusted life-years gained (QALYs) over

For peer review only - http://bmjopen.bmj.com/site/about/guidelines.xhtml

a lifetime horizon. Quality of life information will be estimated from anonymised patient-level data by the same group from an earlier study in this instance.

Outcomes

Primary Outcome

The primary outcome will be the proportion of men with clinically significant PCa detected – any pattern 4 disease on any core (*i.e.* Gleason ≥3+4 or Gleason Grade Group ≥2). The time frame for assessment: when biopsy results are available, at an expected average of 30 days post-biopsy.

Secondary Outcomes

Table 1 lists our secondary outcomes.

Sample Size

The margin of clinical unimportance to allow a conclusion of non-inferiority of bpMRI to mpMRI to be made was set at 5 percentage points – i.e. if the lower bound of the 95% confidence intervals (CIs) for the difference in detection rates of bpMRI-targeted biopsy compared to mpMRI-targeted biopsy is above -5 percentage points, then bpMRI will be deemed as non-inferior.

Using simulation, an mpMRI underlying probability of detecting clinically significant cancer of 38% (3) and the following, two key probabilities were used to determine the sample size:

A. The probability that a patient found to have no suspicious lesions on bpMRI or have no clinically significant PCa on bpMRI-targeted biopsy will have clinically significant PCa on mpMRI-targeted biopsy

B. The probability that a patient found to have no suspicious lesions on mpMRI or have no clinically significant PCa on mpMRI-targeted biopsy will have clinically significant PCa on bpMRI-targeted biopsy

Assuming the probability of A is greater than the probability of B, and applying McNemar's test in each of 1,000 simulation runs for each combination of probabilities A and B ranging from 0 to 0.05, a sample size of 400 patients gives more than 90% power across these probabilities of A and B. Accounting for 20% dropout or exclusion after enrolment, at least 500 patients will be required.

Recruitment

At each participating site, enrolment will occur at outpatient clinics. With at least 25 sites, it is estimated that the trial will complete within 24 months of commencement. The trial opened for recruitment in April 2022 and the estimated completion date is April 2024.

Data Collection Methods

The electronic case report form (eCRF) system Marvin by XClinical will be used to collect data.

Patient-Reported Outcome Measures

The International Index of Erectile Function (IIEF-5) and the International Prostate Symptom Score (IPSS) will be utilised to assess baseline erectile function and lower urinary tract symptoms, respectively. These questionnaires will aid the multidisciplinary team decision-making for treatment eligibility.

Patient Retention

It is estimated that loss to follow-up will be no more than 20% due to the expected short time interval between enrolment and end of study. It is expected that the majority of patients will complete the trial within 4 to 6 weeks (**Table 2A, Table 2B**).

For peer review only - http://bmjopen.bmj.com/site/about/guidelines.xhtml

Statistical Methods

A statistical analysis plan will be finalised before our database lock and before any statistical analysis occurs. A consort diagram will be presented. All continuous variables will be described using the mean and standard deviation, or median and interquartile range, as appropriate. Categorical variables will be described using frequencies and percentages. Baseline characteristics will be examined and presented for those with and those without clinically significant PCa. The assumptions underpinning the statistical methods used will be assessed. The use of transformations will be considered to satisfy statistical assumptions.

Primary Outcome Analysis

The primary outcome is the difference in the proportion of men with clinically significant PCa, as detected by bpMRI-targeted biopsy compared to mpMRI-targeted biopsy. The proportion of men with clinically significant PCa, Gleason ≥ 3+4 or Gleason Grade Group ≥ 2, detected by bpMRI-targeted biopsy is defined as the number of men with clinically significant PCa identified on bpMRI-targeted biopsy divided by the number of men undergoing bpMRI. Similarly, the proportion of men with clinically significant PCa detected by mpMRI-targeted biopsy is defined as the number of men with clinically significant PCa identified on mpMRI-targeted biopsy divided by the number of men undergoing mpMRI. Methods that account for the paired nature of the data such as McNemar's test will be used to compare bpMRI and mpMRI.

Secondary Outcome Analysis

The proportion of men with clinically insignificant cancer (any cancer core with Gleason 3+3 or Gleason grade group 1 detected by bpMRI-targeted biopsy will be compared to that of mpMRI-targeted biopsy. The proportion of men with clinically insignificant cancer detected by bpMRI-targeted biopsy is defined as the number of men with clinically insignificant prostate cancer identified on bpMRI-targeted biopsy divided by the number of men undergoing bpMRI. Similarly, the proportion of men with clinically insignificant cancer detected by mpMRI-targeted biopsy is defined as the number of men with clinically insignificant prostate cancer identified on mpMRI-targeted biopsy divided by the number of men undergoing mpMRI. The same analytical approach described for clinically significant PCa will be applied.

The number and proportion of men scoring 1 or 2 (non-suspicious) or 3 (indeterminate) on bpMRI and mpMRI will be reported. A two-way table will be produced to show the agreement between the two MRI results using the Likert scoring system on a scale of 1-5.

The number and proportion of men with adequate standard of reporting on bpMRI and mpMRI will be reported.

A two-way table will be produced to show the number and proportion of patients with each radiological stage of bpMRI and mpMRI. Similarly, we will report the number and proportion of patients eligible for different treatment options following discussion of the bpMRI and mpMRI results in the Multidisciplinary Team meeting.

Using histopathology as the reference standard, sensitivity, specificity, positive predictive value and negative predictive value with 95% CI of bpMRI and mpMRI will be reported. The following assumptions will be made, where non-suspicious MRI refers to a score of 1 or 2 and suspicious MRI refers to a score of 3, 4 or 5 on the Likert and PI-RADS v2.1 scoring systems and absence of clinically significant cancer refers to a combination of clinical insignificant and no cancer.

The number and proportion of men with clinically significant cancer detected by systematic biopsy and not detected by bpMRI and mpMRI with targeted biopsy will be reported. A two-way table will be

For peer review only

produced to show a comparison between systematic biopsy (no biopsy, clinically significant cancer, clinically insignificant cancer and no cancer) and the two MRI results with targeted biopsy (no biopsy, clinically significant cancer, clinically insignificant cancer and no cancer).

Sensitivity and Other Planned Analyses

The primary outcome analysis will be repeated with a definition of clinically significant PCa being any primary pattern 4 disease – Gleason 4+3 or Gleason Grade Group 3.

Monitoring

The NCITA Global Prostate Trial Steering Committee (TSC) is responsible for the governance of the PRIME Study. A sub-group of independent TSC members form the Data Monitoring Sub-Committee (DMSC).

Roles and responsibilities of the TSC

To act as the oversight body for up to five prostate cancer studies on behalf of the Sponsor and Funders. In addition, the independent members will form a DMSC to review safety. The role of the TSC is to provide oversight for the studies and provide advice through its Chair to the Chief Investigators (CI), whilst working in tandem with the DMSC, Sponsor, Funders and host institution on all aspects of the studies. The rights, safety and well-being of the study participants are the most important consideration and should prevail over the interests of science and society.

Harms

Adverse events (AEs) will be defined as 'any untoward medical occurrence in a clinical trial subject undergoing any intervention in the trial, which does not necessarily have a causal relationship with this treatment'.

Serious adverse events (SAEs) will be defined as 'any untoward medical occurrence as a result of any intervention in the trial that:

- Results in death,
- Is life-threatening
- Requires hospitalisation or prolongation of existing inpatients' hospitalisation, results in persistent or significant disability or incapacity'

AEs and SAEs will be recorded until 30 days post biopsy. In the event that the patient does not undergo biopsy, AEs and SAEs should be recorded until 30 days post-MRI.

Unexpected AEs will be recorded by a member of the research team or clinical team on an AE report form eCRF. All SAEs must be recorded on an SAE report form eCRF which must be sent to the coordinating trials unit within 24 hours of knowledge of the SAE. Both AEs and SAEs should be recorded in the medical notes.

Ethics and Approval

The UK National REC (West Midlands – Black Country Research Ethics Committee, Nottingham) gave favourable approval for PRIME protocol version 2.0 on 26 May 2021 (Ref:21/WM/0091). All participating centres have gained local and ethical approvals prior to receiving a site initiation visit and approval by the sponsor to open for recruitment.

Patient and Public Involvement

Patients and public members were involved in defining the research question, evaluation of the research proposal, suggesting modifications to the trial, reviewing the patient information sheet,

For peer review only - http://bmjopen.bmj.com/site/about/guidelines.xhtml

consent form and GP letter. Patient groups and charities will also be involved in the dissemination of results.

Consent

The clinical teams managing patients with suspected PCa who are referred to their centre will identify potential trial participants. Patient information sheets will be provided to patients. Members of staff who are trained to take informed consent, as indicated by the PI on the delegation log for that site, will take informed consent. A model patient information sheet is shown in **Supplementary Appendix 3**.

Confidentiality

The data of the participants will be recorded into the eCRF system and analysed without any personal identifiers, by pseudoanonymised coded information. A site's source documents and identification lists will be archived in a secured facility at that centre.

Dissemination

Results of this trial will be disseminated through national and international conferences and papers. Authorship criteria will be based on recommendations of the International Committee of Medical Journal Editors. The participants and relevant patient support groups will be informed about the results of the trial.

Access to Data

Only authorised individuals within the PRIME Clinical Operations Group have access to the final data set. Individual PIs have access to their own data but not that of other sites.

Declaration of Interests

AN is an academic clinical fellow funded by the National Institute for Health and Care Research. PK is an academic clinical fellow funded by the National Institute for Health and Care Research and The Urology Foundation. FG is a recipient of the 2020 Young Investigator Award (20YOUN15) funded by the Prostate Cancer Foundation / CRIS Cancer Foundation. SP is supported by the National Institute of Health and Care Research (NIHR), UCLH and UCL Biomedical Research Centre. ME receives research support from the National Institute of Health and Care Research (NIHR), UCLH and UCL Biomedical Research Centre. YT is funded by a UK NIHR Postdoctoral Fellowship and supported by the NIHR Birmingham Biomedical Research Centre. CMM is an NIHR Research Professor, and receives grants from MRC, CRUK, Movember, and Prostate Cancer UK. VK is funded by Prostate Cancer UK and The John Black Charitable Foundation. He receives speaker fees from the European Association of Urology, Singapore Urology Association, The Clinical Comms Group and Got IT consulting SL. All authors declare that there are no conflicts of interest. The views expressed in this publication are those of the authors and not necessarily those of the National Health Service, NIHR, or the Department of Health and Social Care.

WHO Trial Registration Dataset

Please see **Table 3** for the WHO trial registration dataset.

Current Protocol Version

The current protocol is V.2.0, issued 27 April 2021. The current protocol amendment number is 01. For full amendment history, please see **Table 4**.

For peer review only - http://bmjopen.bmj.com/site/about/guidelines.xhtml

Funding
The PRIME is primarily funded by Prostate Cancer UK (grant number: TLD-PF19-004) and The John Black Charitable Trust Travelling Prize Grant (grant number: TLD-PF19-004). The EAU Research Foundation (EAU RF) and The Dieckmann Foundation also supported costs for international sites.

Roles and Responsibilities
Please see **Table 5** for roles and responsibilities of the trial sponsor and involved committees.

Acknowledgements
We would like to thank our patients and funders, without whom we wouldn't be able to carry out this important study: Prostate Cancer UK & The John Black Charitable Trust, European Association of Urology Research Foundation and the Dieckmann Foundation. We thank all the international centres taking part in PRIME. We are grateful to EAU Research Foundation and the XClinical team for their support with the MARVIN database; and Sydney Lindner, Steven Lelie, Jessica Sternisa, Tyler Edwards, Adam Kulp, Jon Piper from the MIM Software Inc team. We are thankful for the trial oversight provided by our sponsor, University College London and the National Cancer Imaging Translational Accelerator trials unit.

Author Contributions
Study concept and design: ANg, AA, AN, VC, PK, FG, CA, AF, SP, PL, CSC, CBG, NM, ME, RA, YT, JD, CMM, VK. Drafting of manuscript: AA, AN, CSC, CBG, RA, YT, VK. Critical revision of the manuscript for important intellectual content: all authors. Supervision: CA, VK. All authors read and approved the final manuscript.

REFERENCES

1. A-W. Chan, JM. Tetzlaff, PC. Gøtzsche, et al. SPIRIT 2013 explanation and elaboration: Guidance for protocols of clinical trials. *BMJ*, 346 (2013), e7586, 10.1136/bmj.e7586.
2. N. Mottet, P. Cornford, R. Van Den Bergh, et al. EAU guidelines: prostate cancer. European Association of Urology, Arnhem, The Netherlands (2021). https://uroweb.org/guideline/prostate-cancer/
3. V. Kasivisvanathan, A.S. Rannikko, M. Borghi, et al. MRI-targeted or standard biopsy for prostate-cancer diagnosis. *N Engl J Med,* 378 (2018), pp. 1767-1777, 10.1056/NEJMoa1801993.
4. N. Mottet, P. Cornford, R. Van Den Bergh, et al. EAU guidelines: prostate cancer. European Association of Urology, Arnhem, The Netherlands (2021). https://uroweb.org/guideline/prostate-cancer/
5. National Prostate Cancer Audit - Annual Report 2019. https://www.npca.org.uk/reports/npca-annual-report-2019/
6. S. Verma, B. Turkbey, N. Muradyan, A. Rajesh, et al. Overview of dynamic contrast-enhanced MRI in prostate cancer diagnosis and management. *AJR Am J Roentgenol,* 198 (2012), pp. 1277–1288. 10.2214/AJR.12.8510
7. R. Berman, A. Brown, S. Chang, et al. DCE MRI of prostate cancer. *Abdom Radiol (NY),* 41 (2016), pp. 844-853. 10.1007/s00261-015-0589-3
8. M. Engelbrecht, H. Huisman, R. Laheij, et al. Discrimination of prostate cancer from normal peripheral zone and central gland tissue by using dynamic contrast-enhanced MR imaging. *Radiology*. 229 (2003), pp. 248-254. 10.1148/radiol.2291020200
9. M. van der Leest, E. Cornel, B. Israël, et al. Head-to-head comparison of transrectal ultrasound-guided prostate biopsy versus multiparametric prostate resonance imaging with subsequent magnetic resonance-guided biopsy in biopsy-naïve men with elevated prostate-specific antigen: a large prospective multicenter clinical study. *Eur Urol,* 75 (2019), pp. 570-578, 10.1016/j.eururo.2018.11.023

For peer review only - http://bmjopen.bmj.com/site/about/guidelines.xhtml

10. L. Boesen, N. Nørgaard, V. Løgager, et al. Assessment of the diagnostic accuracy of biparametric magnetic resonance imaging for prostate cancer in biopsy-naive men: the Biparametric MRI for Detection of Prostate Cancer (BIDOC) study. *JAMA Netw Open*, 1 (2018), p. e180219, 10.1001/jamanetworkopen.2018.0219
11. Z. Kang, X. Min, J. Weinreb, Q. Li, Z. Feng, L. Wang. Abbreviated biparametric versus standard multiparametric MRI for diagnosis of prostate cancer: a systematic review and meta-analysis. *Am J Roengenol*, 212 (2018), pp. 357-365, 10.2214/ajr.18.20103
12. M. Alabousi, J.P. Salameh, K. Gusenbauer, et al. Biparametric vs multiparametric prostate magnetic resonance imaging for the detection of prostate cancer in treatment-naïve patients: a diagnostic test accuracy systematic review and meta-analysis. *BJU Int*, 124 (2019), pp. 209-220, 10.1111/bju.14759
13. A. Ng, P. Khetrapal, V. Kasivisvanathan. Is It PRIME Time for Biparametric Magnetic Resonance Imaging in Prostate Cancer Diagnosis? *Eur Urol,* S0302-2838 (2022), pp. 01663-3, 10.1016/j.eururo.2022.02.021.
14. F. Giganti, C. Allen, M. Emberton, C.M. Moore, V. Kasivisvanathan. Prostate Imaging Quality (PI-QUAL): a new quality control scoring system for multiparametric magnetic resonance imaging of the prostate from the PRECISION trial. *Eur Urol Oncol,* 3 (2020), pp. 615-619, 10.1016/j.euo.2020.06.007.
15. J.C. Weinreb, J.O. Barentsz, P.L. Choyke, et al. PI-RADS Prostate Imaging - Reporting and Data System: 2015, Version 2. *Eur Urol*, 69 (2016), pp. 16-40, 10.1016/j.eururo.2015.08.052.

FIGURE LEGENDS

Figure 1 The PRIME trial schema – the approach prior to MRI.
(bpMRI = biparametric MRI, DCE = dynamic contrast enhanced sequence, DRE = digital rectal examination, DWI = diffusion-weighted sequence, mpMRI = multiparametric MRI, PSA = prostate specific antigen, T2W = T2-weighted sequence.)

TABLES

**Table 1** Secondary outcomes in PRIME	
**Outcome**	**Time frame for assessment**
Proportion of men with clinically insignificant cancer (Gleason grade 3+3 / Gleason grade group 1)	When biopsy results available, at an expected average of 30 days post-biopsy
Agreement between bpMRI and mpMRI for score of suspicion	When MRI results available, at an expected average of 30 days post-MRI
Proportion of bp-MRI scans and mpMRI whose quality was deemed adequate for reporting	When MRI results available, at an expected average of 30 days post-MRI
Agreement between bpMRI and mpMRI for radiological staging decision	When MRI results available, at an expected average of 30 days post-MRI
Agreement between bpMRI and mpMRI for treatment eligibility	When treatment eligibility is discussed in a multidisciplinary meeting, at an expected average of 30 days post biopsy.
Test performance characteristics for bpMRI and mpMRI when using the Likert scoring system in comparison to the PI-RADS scoring system	When biopsy results available, at an expected average of 30 days post-MRI
Proportion of men with clinically significant cancer missed by bpMRI and mpMRI-targeted biopsies and detected by systematic biopsy	When biopsy results available, at an expected average of 30 days post-biopsy

For peer review only - http://bmjopen.bmj.com/site/about/guidelines.xhtml

Cost-effectiveness of bpMRI compared to mpMRI (cost per diagnosis of prostate cancer)	At an expected average of 30 days post-intervention

(bpMRI = biparametric MRI; mpMRI = multiparametric MRI.)

Table 2A Participant timeline in the trial: the timeline for men enrolled to the trial prior to undergoing MRI

	**Contact with patient**				
	**Visit 0***	**Visit 1**	**Visit 2**	**Visit 3**	**Visit 4**
Screening	X	X			
PIS given	X	X			
Consent	X	X			
IIEF-5 and IPSS questionnaires	X	X			
Multiparametric MRI			X		
Radiologists reports bpMRI (T2W and DWI only)			X		
Radiologists reports mpMRI (T2W, DWI and DCE)			X		
MRI-targeted biopsy and systematic biopsy				X	
Test results given and treatment decision					X
Follow-up for further investigations from treatment decision					X
Serious adverse event	Complete as required at any time following registration				
Withdrawal form	Complete as required at any time following registration				

*(*Visit 0 is an optional teleconsult, depending on local practice. Note: where applicable, more than one visit can take place on the same day, depending on local practice (e.g. in centres where an MRI is performed on the same day as subsequent biopsies).*

IIEF-5 = The International Index of Erectile Function, IPSS = International Prostate Symptom Score, PIS = patient information sheet, bpMRI = biparametric MRI, mpMRI = multiparametric MRI, T2W = T2-weighted sequence, DWI = diffusion-weighted sequence, DCE = dynamic contrast-enhanced sequence.)

Table 2B Participant timeline in the trial: the timeline for men enrolled after undergoing multiparametric MRI as part of routine care

	**Contact with patient**				
	**Visit 0**	**Visit 1**	**Visit 2**	**Visit 3**	**Visit 4**
Screening		X			
PIS given		X			
Consent		X			
IIEF-5 and IPSS questionnaires	X				
Multiparametric MRI		X			
Radiologists reports bpMRI (T2W and DWI only)		X			
Radiologists reports mpMRI (T2W, DWI and DCE)		X			
MRI-targeted biopsy and systematic biopsy			X		
Test results given and treatment decision					X
Follow-up for further investigations from treatment decision					X

For peer review only

Serious adverse event	Complete as required at any time following registration
Withdrawal form	Complete as required at any time following registration

(IIEF-5 = The International Index of Erectile Function, IPSS = International Prostate Symptom Score, PIS = patient information sheet, bpMRI = biparametric MRI, mpMRI = multiparametric MRI, T2W = T2-weighted sequence, DWI = diffusion-weighted sequence, DCE = dynamic contrast-enhanced sequence.)

Table 3 WHO trial registration dataset

**Data category**	**Information**
Primary registry and trial identifying number	ClinicalTrials.gov: NCT04571840
Date of registration in the primary registry	October 1, 2020
Sources of monetary or material support	• Prostate Cancer UK<br>• The John Black Charitable Foundation<br>• European Association of Urology Research Foundation<br>• The Dieckmann Foundation
Primary sponsor	University College London
Secondary sponsor(s)	N/A
Contact for public queries	Mr Veeru Kasivisvanathan veeru.kasi@ucl.ac.uk<br>Div of Surgery & Interventional Sci,<br>University College London,<br>3rd Floor, Charles Bell House,<br>43-45 Foley Street, London, W1W 7TS
Contact for scientific queries	Mr Veeru Kasivisvanathan veeru.kasi@ucl.ac.uk<br>Div of Surgery & Interventional Sci,<br>University College London,<br>3rd Floor, Charles Bell House,<br>43-45 Foley Street, London, W1W 7TS
Public title / short title	Prostate Imaging Using MRI +/- Contrast Enhancement (PRIME)
Acronym	PRIME
Scientific title	A trial assessing whether biparametric MRI is non-inferior to multiparametric MRI in the diagnosis of clinically significant prostate cancer
Countries of recruitment	Argentina<br>Australia<br>Belgium<br>Brazil<br>Canada<br>Denmark<br>France<br>Finland<br>Germany<br>Italy<br>The Netherlands<br>Singapore

For peer review only

For peer review only - http://bmjopen.bmj.com/site/about/guidelines.xhtml

	Spain<br>UK<br>USA
Health condition(s) or problem(s) studied	Prostate neoplasm
Intervention(s)	Device: MRI<br>Diagnostic Test: Multiparametric MRI +/- prostate biopsy<br>Diagnostic Test: Biparametric MRI +/- prostate biopsy
Intervention description	1. Active comparator: Multiparametric MRI (mpMRI)<br>MRI with T2-weighted, diffusion weighted and dynamic contrast enhanced sequences followed by prostate biopsy if indicated on MRI and clinical findings Diagnostic Test: Multiparametric MRI +/- prostate biopsy<br><br>1. Experimental: Biparametric MRI (bpMRI)<br>MRI with T2-weighted and diffusion weighted sequences followed by prostate biopsy if indicated on MRI and clinical findings<br>Diagnostic Test: Biparametric MRI +/- prostate biopsy
Key inclusion and exclusion criteria	Inclusion Criteria:<br>1. Men at least 18 years of age referred with clinical suspicion of prostate cancer<br>2. Serum PSA ≤ 20ng/mL<br>3. Fit to undergo all procedures listed in protocol<br>4. Able to provide written informed consent<br><br>Exclusion Criteria:<br>1. Prior prostate biopsy<br>2. Prior treatment for prostate cancer<br>3. Prior prostate MRI on a previous encounter<br>4. Contraindication to MRI<br>5. Contraindication to prostate biopsy<br>6. Unfit to undergo any procedures listed in protocol
Study type	Interventional<br>Allocation: Non-Randomized<br>Intervention Model: Single Group Assignment<br>Intervention Model Description: Within-person controlled, paired cohort, diagnostic evaluation study. Participants undergo two index tests and a reference test.<br>Masking: Single (Care Provider)<br>Masking Description: Radiologist assessing MRI for suspicion of prostate cancer is blinded to the contrast sequence when reporting the biparametric MRI. After this report, they are unblinded to the contrast sequence and report the multiparametric MRI. All biopsies conducted as a result of MRI findings will be labelled as bpMRI and mpMRI, and diagnostic accuracy will be assessed against histology findings.
Date of first enrolment	05 April 2022
Target sample size	500
Recruitment status	Recruiting

For peer review only - http://bmjopen.bmj.com/site/about/guidelines.xhtml

Primary outcome(s)	Proportion of men with clinically significant cancer
Key secondary outcomes	• Proportion of men with clinically insignificant cancer (Gleason grade 3+3 / Gleason grade group 1)<br>• Agreement between bpMRI and mpMRI for score of suspicion<br>• Proportion of bp-MRI scans and mpMRI whose quality was deemed adequate for reporting<br>• Agreement between bpMRI and mpMRI for radiological staging decision<br>• Agreement between bpMRI and mpMRI for treatment eligibility<br>• Test performance characteristics for bpMRI and mpMRI when using the Likert scoring system in comparison to the PI-RADS scoring system<br>• Proportion of men with clinically significant cancer missed by bpMRI and mpMRI-targeted biopsies and detected by systematic biopsy<br>• Cost-effectiveness of bpMRI compared to mpMRI (cost per diagnosis of prostate cancer)

Table 4 Revision chronology for amendments to protocol

**Protocol version to date**	**Reasons for amendments**
V.1.0, issued 24 August 2020	Original protocol
V.2.0, issued 27 April 2021	Main reasons for amendment: minor changes to make existing trial documents clearer. Main changes:<br>1. Updated Section 18 Record Keeping and Archiving. Added the sentence, "Identifiable data will be kept by the site for 10 years, and non-identifiable data will be kept for a minimum of 20 years."<br>2. Version number and date added to all pages.

Table 5 Roles and responsibilities in the PRIME Trial

**Role**	**Details and responsibilities**
Trial sponsor	University College London (UCL)<br>Sponsor's Edge reference: 135819<br>Email: Rand.D@uclh.nhs.uk<br>The trial sponsor did not provide any funding for the study. University College London has the role of research governance sponsor of PRIME. UCL adopted the study as sponsor after the UCL CCTU carried out a trial adoption process which involved the UCL CCTU reviewing the protocol to ensure it conformed to high standards of trial conduct and met the governance requirements of UCL. The UCL CCTU is responsible for oversight of the trial. The sponsor plays no role in data collection, management, analysis and interpretation of data, writing of the report or the decision to submit the report for publication.
PRIME Operations Group	The PRIME Operations Group consists of the chief investigator, the Clinical Operations Group, National Cancer Imaging Translational Accelerator, the UCL Surgical and Interventional Trials Unit and the eCRF database managers. This group is responsible for:<br>• Study planning<br>• Preparation of protocol and revisions

For peer review only

	• Assistance with international review board/independent ethics committee applications<br>• Preparation of investigators brochure and CRFs<br>• Organisation of steering committee meetings<br>• Provide annual progress reports to the ethics committee<br>• Reporting serious adverse events to the sponsor and ethics committee when necessary<br>• Responsible for trial master file<br>• Budget administration and contractual issues with individual centres<br>• Advice for PIs<br>• Site initiation visits<br>• Data verification and management<br>• Central monitoring and resolving data queries with clinicians and nurses at the trial sites<br>• Maintenance of the trial Information Technology (IT) system<br>• Publication of study reports
Principal Investigator	At each participating site, the PI is responsible for the conduct of the clinical trial to ensure the safety of participants and the reliability and robustness of the data generated. They will be responsible for identification, recruitment, data collection and completion of CRFs, along with follow-up of trial patients and adherence to trial protocol. The PI as leader of the research team may delegate their duties to members of their team.
Global Trial Steering Committee	The NCITA global prostate trial steering committee (TSC) is responsible for the governance of the PRIME Study, and they have delegated safety to a data monitoring subcommittee (DMSC).<br><br>Roles and responsibilities:<br>To act as the oversight body for up to five prostate cancer studies on behalf of the Sponsor and Funders. In addition, the independent members will form a sub-committee to review safety. The role of the TSC is to provide oversight for the studies and provide advice through its Chair to the Chief Investigators (CI), work in tandem with the Data Monitoring Sub-Committee (DMSC), Sponsor, Funders and host institution on all aspects of the studies. The rights, safety and well-being of the study participants are the most important consideration and should prevail over the interests of science and society.

For peer review only

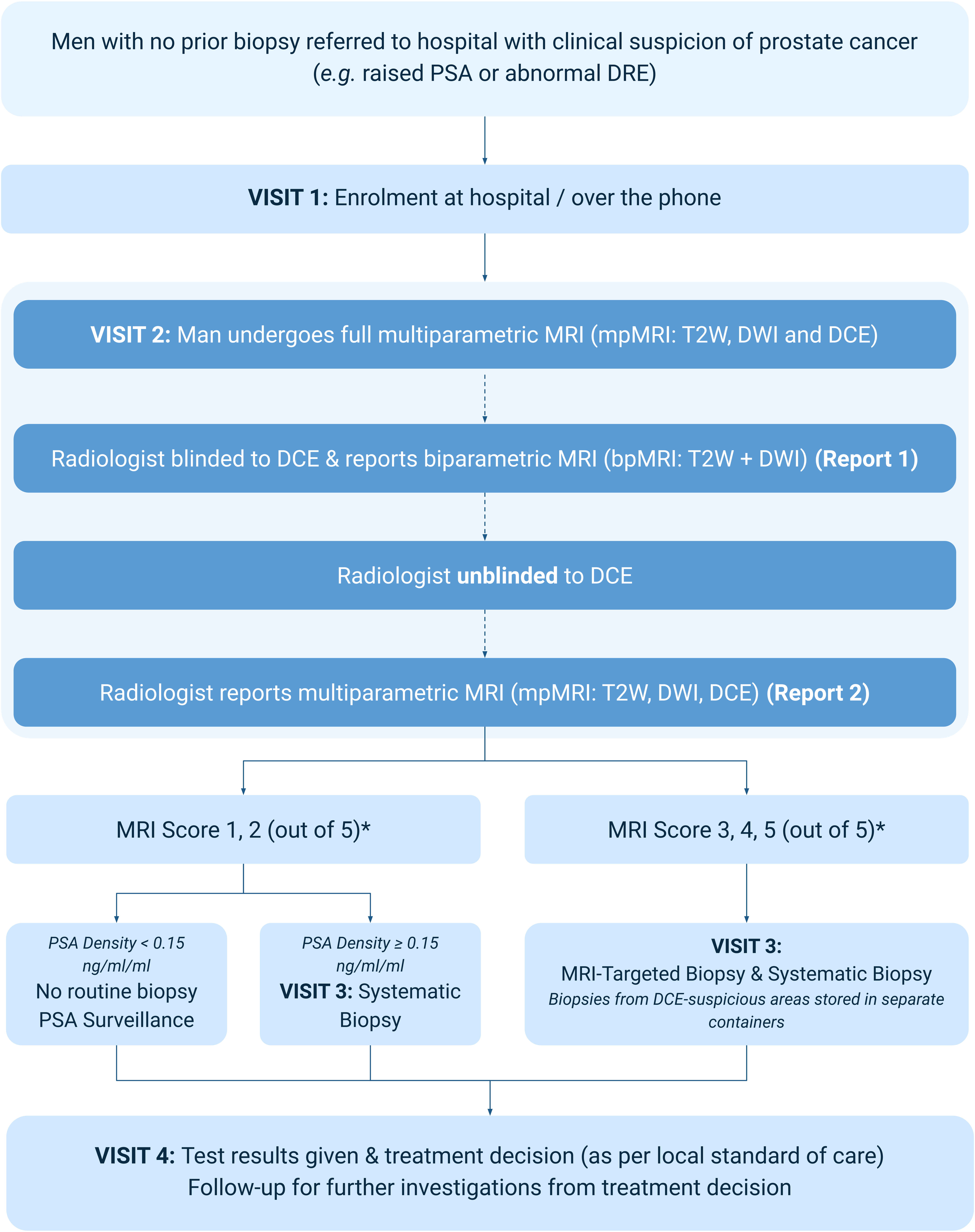
Men with no prior biopsy referred to hospital with clinical suspicion of prostate cancer (e.g. raised PSA or abnormal DRE)
VISIT 1: Enrolment at hospital / over the phone
VISIT 2: Man undergoes full multiparametric MRI (mpMRI: T2W, DWI and DCE)
Radiologist blinded to DCE & reports biparametric MRI (bpMRI: T2W + DWI) (Report 1)
Radiologist unblinded to DCE
Radiologist reports multiparametric MRI (mpMRI: T2W, DWI, DCE) (Report 2)
MRI Score 1, 2 (out of 5)*
MRI Score 3, 4, 5 (out of 5)*
PSA Density < 0.15 ng/ml/ml
No routine biopsy
PSA Surveillance
PSA Density ≥ 0.15 ng/ml/ml
VISIT 3: Systematic Biopsy
VISIT 3:
MRI-Targeted Biopsy & Systematic Biopsy
Biopsies from DCE-suspicious areas stored in separate containers
VISIT 4: Test results given & treatment decision (as per local standard of care)
Follow-up for further investigations from treatment decision

TRIAL IDENTIFIER: PARTICIPANT INITIALS:

Reporting Proforma (bpMRI):

Report 1 – Biparametric MRI (bpMRI) Report

This report should be completed **without looking at the contrast sequence.**

If applicable, complete the Target boxes & link these to your drawings of the Targets (e.g. with lines / colours)

Target 1	Likert:<br><br>PIRADS:

Target 2	Likert:<br><br>PIRADS:

Target 3	Likert:<br><br>PIRADS:

Target 4	Likert:<br><br>PIRADS:

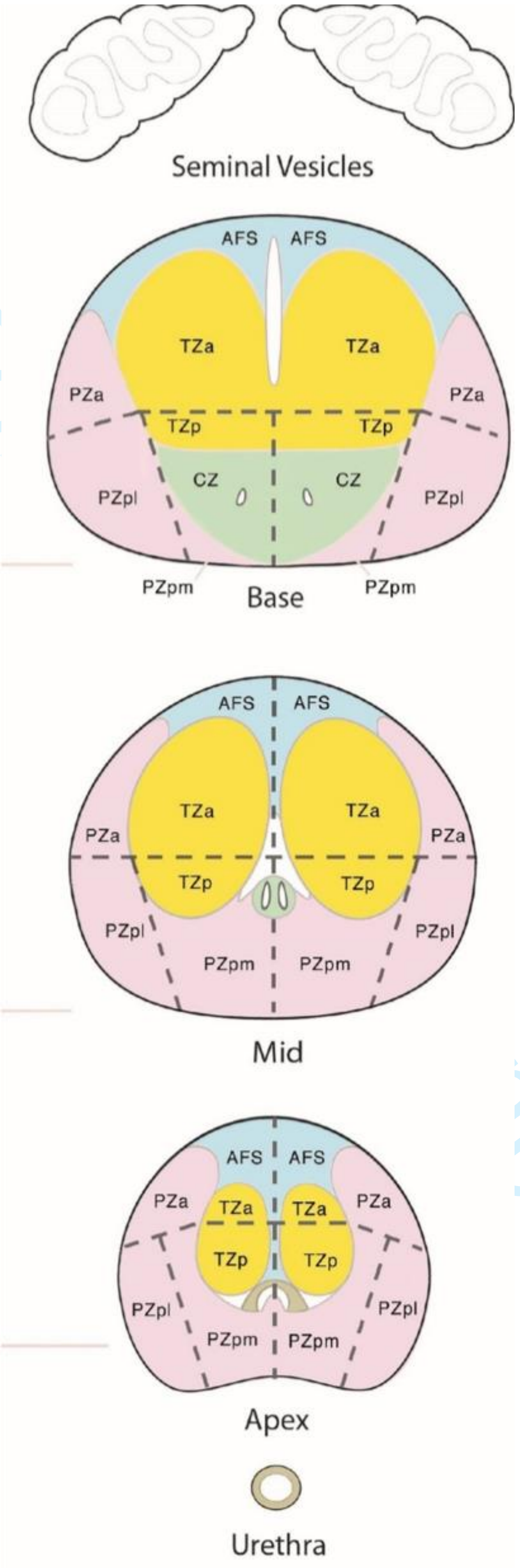

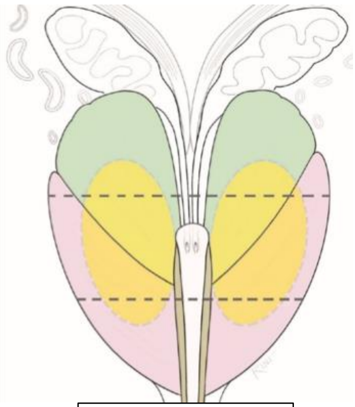

Coronal plane

Reminder of Likert Score: Likelihood of target containing significant cancer:

1 = Highly unlikely

2 = Unlikely

3 = Equivocal

4 = Likely

5 = Highly Likely

In the case of diffuse changes on **both sides** of the prostate scoring ≥3 (Likert), the diffuse changes on each side of the prostate can be arbitrarily treated as **separate targets.** **"**Diffuse change" is defined as an intermediate or low T2 signal that occupies the majority of at least one side of the peripheral zone, without a defined border

For peer review only - http://bmjopen.bmj.com/site/about/guidelines.xhtml

TRIAL IDENTIFIER: PARTICIPANT INITIALS:

1. Radiologists should **first** annotate, draw and label the diagram on the first page with **up to 3** suspicious areas scoring ≥ 3 on the Likert scale (L) of suspicion (1–5). Clinical information is permitted to be used to influence the score.
2. Radiologists should then score suspicious areas **strictly** using the PI-RADS v2.1 (P) criteria, **without** allowing clinical information to influence the score.
3. If an additional area of suspicion is identified when scoring with PI-RADS v2.1 that was **not** present on Likert, please draw on this 4th suspicious area.

A maximum of **4 targets** can be drawn on this report.

---

1. Every lesion **must have both** a Likert and PI-RADS v2.1 score marked on.
2. Mark the **most suspicious** area, "Target 1".
   a. Mark the **next most suspicious area**, "Target 2".
   b. Mark the **subsequent most suspicious area**, "Target 3" and so on.
3. **On the diagram above, every** lesion drawn must have the following marked and labelled:
   a. Target number
   b. Likert score
   c. PI-RADS v2.1 score
4. Please then insert these into **Table 1** and fill out the rest of the proforma.

e.g. Target 1. Likert 3. PI-RADS 1.

MRI Scanner and Clinical Information

Patient age (years):		PSA (ng/ml):		Which MRI scanner was used?<br>1. ☐ SCANNER ONE<br>2. ☐ SCANNER TWO<br>3. ☐ SCANNER THREE
MRI volume of prostate (ml):		PSA Density (ng/ml/ml):		
Field Strength of Magnet	☐ 1.5T ☐ 3T			

Confirmation of blinding

Confirmation by another individual / system that the radiologist is **blinded** to DCE images (**mandatory**)	☐ Yes	☐ No

---

For peer review only - http://bmjopen.bmj.com/site/about/guidelines.xhtml

TRIAL IDENTIFIER: PARTICIPANT INITIALS:

Table 1. Please only enter Targets below if the Likert or PI-RADS v2.1 score is ≥ 3.

**TARGET SPECIFIC INFORMATION**	TARGET 1	TARGET 2	TARGET 3	TARGET 4
Location of suspicious area(s) (select **one** option):	☐ Right<br>☐ Left<br>☐ Bilateral	☐ Right<br>☐ Left<br>☐ Bilateral	☐ Right<br>☐ Left<br>☐ Bilateral	☐ Right<br>☐ Left<br>☐ Bilateral
Location in prostate according to PI-RADS v2.1 41-sector diagram (select **the one main** location which contains the target):	☐ Base<br>☐ Mid<br>☐ Apex<br>☐ Seminal Vesicle	☐ Base<br>☐ Mid<br>☐ Apex<br>☐ Seminal Vesicle	☐ Base<br>☐ Mid<br>☐ Apex<br>☐ Seminal Vesicle	☐ Base<br>☐ Mid<br>☐ Apex<br>☐ Seminal Vesicle
Main sector which contains the lesion according to PI-RADS v2.1 41-sector diagram (write **one**, *e.g.* "PZpl"):				
Likert score of suspicion (1–5):				
PI-RADS v2.1 score of suspicion (1–5):				
Target appearance (select **one**):<br>The default is focal, unless there is diffuse change in the peripheral zone	☐ Focal<br>☐ Diffuse	☐ Focal<br>☐ Diffuse	☐ Focal<br>☐ Diffuse	☐ Focal<br>☐ Diffuse
Biaxial diameter on sequence where it was largest, in axial plane (mm x mm):				
Sequence used to measure biaxial diameter (select **one**):	☐ T2<br>☐ High b<br>☐ ADC	☐ T2<br>☐ High b<br>☐ ADC	☐ T2<br>☐ High b<br>☐ ADC	☐ T2<br>☐ High b<br>☐ ADC

Please complete the **overall scores** regardless of whether there are any Targets identified above:

**Overall patient Likert score**<br>Enter the highest Likert score		**Overall patient PIRADS v2.1 score**<br>Enter the highest PI-RADS v2.1 score	

If there are no Targets scoring ≥ 3 on either scoring system, then the overall Likert and PI-RADS v2.1 score will be 1 or 2.

For peer review only - http://bmjopen.bmj.com/site/about/guidelines.xhtml

TRIAL IDENTIFIER: PARTICIPANT INITIALS:

Table 2. Staging information. Complete **only if** a Target has been identified above:

Radiological stage:	☐ T2a	☐ T2b	☐ T2c	☐ T3a	☐ T3b	☐ T4
	Radiological T3a = unequivocal extracapsular disease					
Likelihood of **right**-sided extracapsular spread*:<br>1 = highly **unlikely**, 3 = equivocal, 5 = highly **likely**	☐ 1	☐ 2	☐ 3	☐ 4	☐ 5	
Likelihood of **left**-sided extracapsular spread*:	☐ 1	☐ 2	☐ 3	☐ 4	☐ 5	
Likelihood of **right** seminal vesicle involvement:	☐ 1	☐ 2	☐ 3	☐ 4	☐ 5	
Likelihood of **left** seminal vesicle involvement:	☐ 1	☐ 2	☐ 3	☐ 4	☐ 5	
Likelihood of urethral sphincter involvement:	☐ 1	☐ 2	☐ 3	☐ 4	☐ 5	
Likelihood of bladder neck involvement:	☐ 1	☐ 2	☐ 3	☐ 4	☐ 5	
Likelihood of rectal involvement:	☐ 1	☐ 2	☐ 3	☐ 4	☐ 5	

**See* PI-RADS v2.1 guidelines for examples of features suggestive of extracapsular spread.

MRI Quality: Please **complete** this for **all** MRIs regardless of whether a Target was identified:

Was there a problem with the quality of the T2W sequence?	☐ Yes			☐ No
Was there a problem with the quality of the DWI sequence?	☐ Yes			☐ No
If there were problems, please describe these (tick **all** that apply):				
For T2W:	☐ Rectal air	☐ Movement artefact	☐ Prosthesis	☐ Other
For DWI:	☐ Rectal air	☐ Movement artefact	☐ Prosthesis	☐ Other
If other, please describe:				
Was the quality of the scan sufficient for you to make a diagnostic assessment?	☐ Yes	☐ No		
Hypothetically, if this patient only had this biparametric MRI scan:				
• Would you typically have recommended a **repeat bpMRI?**	☐ Yes	☐ No		
• Would you typically have recommended a **contrast sequence** to be done?	☐ Yes	☐ No		

Radiologist (Forename, Surname):		Date of MRI:	
		Date of Report:	

For peer review only - http://bmjopen.bmj.com/site/about/guidelines.xhtml

TRIAL IDENTIFIER: PARTICIPANT INITIALS:

Reporting Proforma (mpMRI):

Report 2 – Multiparametric MRI (mpMRI) Report

The same radiologist should annotate the diagrams below after they are **unblinded** to the **DCE sequence**. This report will be used by the biopsy operator to perform **targeted biopsy**.

A total of **maximum 8 suspicious areas scoring ≥ 3 on either Likert or PI-RADS v2.1** can be annotated in this report.

PART ONE: TARGETS SEEN ON BPMRI

1. First, copy any targets drawn on **Report 1** (bpMRI) onto this report (**Report 2 – mpMRI**).
   a. Draw them on the diagram.
   b. Specify their biparametric MRI status (bpMRI +ve or bpMRI -ve) when you label each lesion.
   c. Add the information about each target to **Table 1** as indicated.
2. Upon viewing the **DCE findings**, for each of these lesions, please specify their multi-parametric MRI status (mpMRI +ve or mpMRI -ve) on the diagram then specify **updated** Likert (L) and PI-RADS v2.1 (P) scores on mpMRI.

Flow diagram: how to complete this proforma for lesions identified on bpMRI (Report 1)

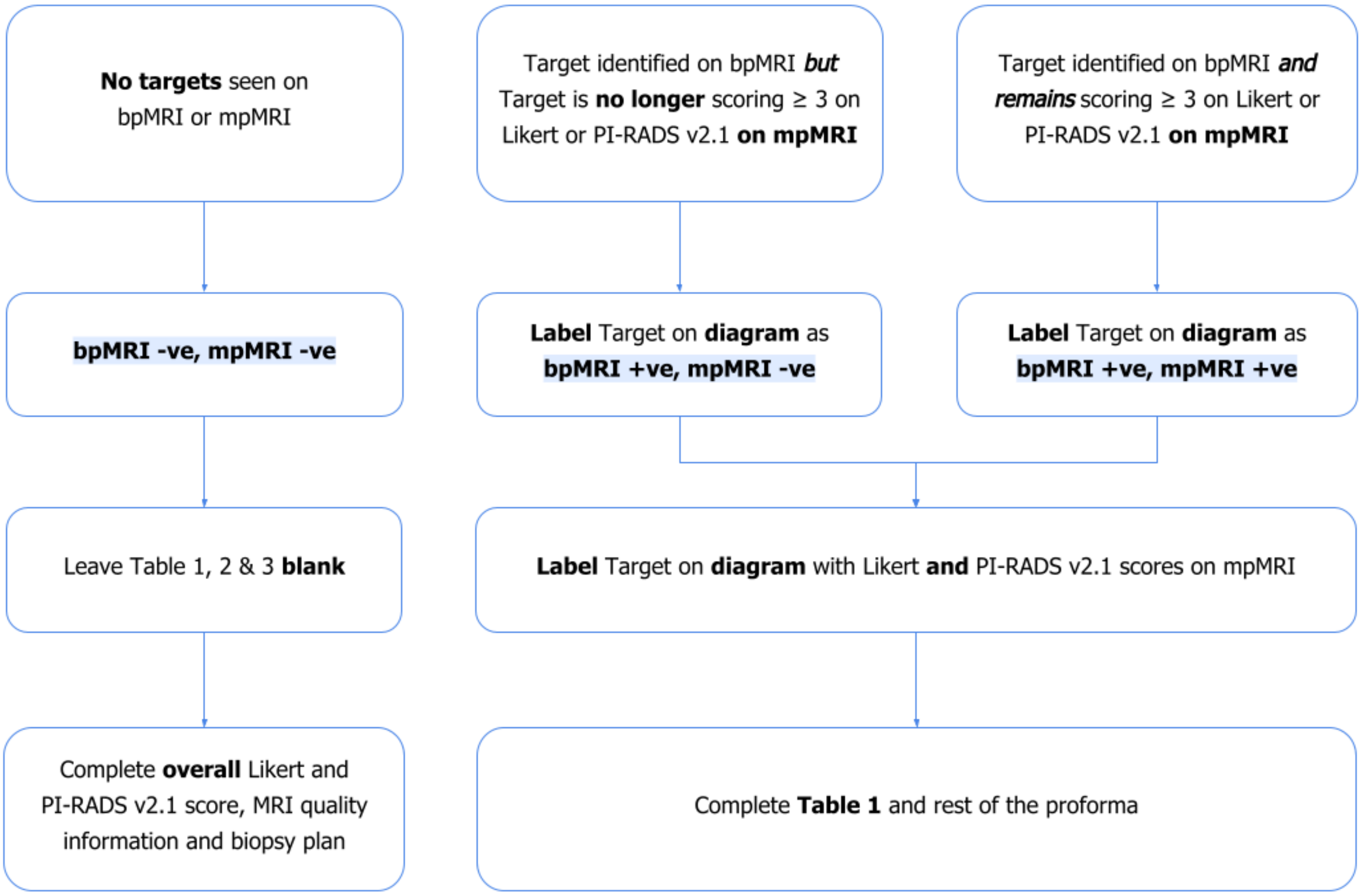

For peer review only - http://bmjopen.bmj.com/site/about/guidelines.xhtml

TRIAL IDENTIFIER: PARTICIPANT INITIALS:

Please draw any Targets on this diagram
and label them according to the flow diagram on Page 1

If applicable, complete the Target boxes & link these to your drawings of the Targets (e.g. with lines / colours)

Target 1	**bpMRI**<br>Likert:<br>PIRADS:	**mpMRI**<br>Likert:<br>PIRADS:

Target 2	**bpMRI**<br>Likert:<br>PIRADS:	**mpMRI**<br>Likert:<br>PIRADS:

Target 3	**bpMRI**<br>Likert:<br>PIRADS:	**mpMRI**<br>Likert:<br>PIRADS:

Target 4	**bpMRI**<br>Likert:<br>PIRADS:	**mpMRI**<br>Likert:<br>PIRADS:

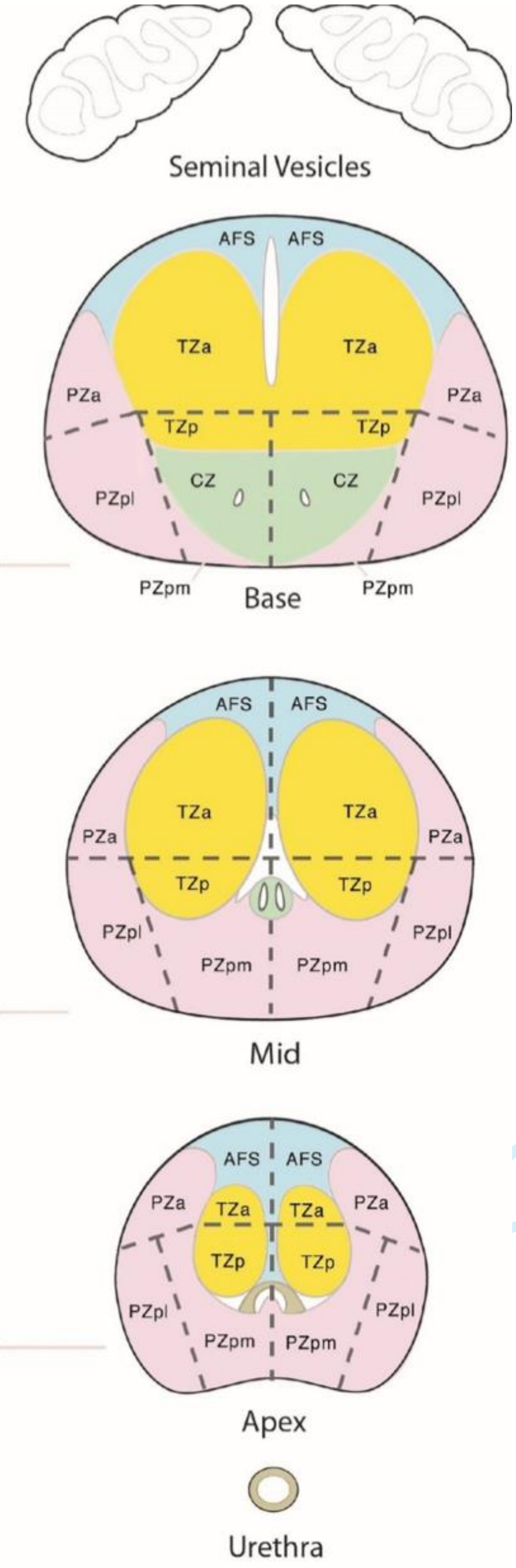

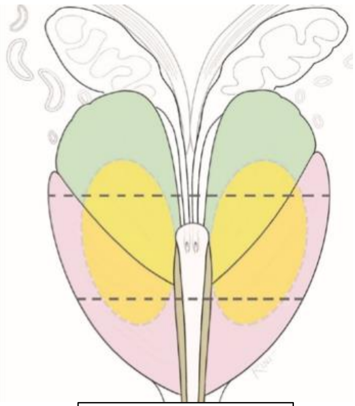

Coronal plane

**DCE-** Target 1	Likert:<br>PIRADS:

**DCE-** Target 2	Likert:<br>PIRADS:

**DCE-** Target 2	Likert:<br>PIRADS:

**DCE-** Target 4	Likert:<br>PIRADS:

In the case of diffuse changes on **both sides** of the prostate scoring ≥3 (Likert), the diffuse changes on each side of the prostate can be arbitrarily treated as **separate Targets.** **"**Diffuse change" is defined as an intermediate or low T2 signal that occupies the majority of at least one side of the peripheral zone, without a defined border.

Please note that every page is mandatory to complete. Version 2.0 [18/02/22]
For peer review only - http://bmjopen.bmj.com/site/about/guidelines.xhtml

TRIAL IDENTIFIER: PARTICIPANT INITIALS:

MRI Scanner and Clinical Information. Complete for all patients:

Patient age (years)		PSA (ng/ml):		
MRI volume of prostate (ml):		PSA Density (ng/ml/ml):		

Table 1. Information from Targets **originally** identified on the **biparametric MRI** (if applicable):

**TARGET SPECIFIC INFORMATION**	TARGET 1	TARGET 2	TARGET 3	TARGET 4
**COPY FROM REPORT 1 (BPMRI):**				
Location of suspicious area(s) (select **one** option):	☐ Right ☐ Left ☐ Bilateral	☐ Right ☐ Left ☐ Bilateral	☐ Right ☐ Left ☐ Bilateral	☐ Right ☐ Left ☐ Bilateral
Location in prostate according to PI-RADS v2.1 41-sector diagram (select **the one main** location which contains the target):	☐ Base ☐ Mid ☐ Apex ☐ Seminal Vesicle	☐ Base ☐ Mid ☐ Apex ☐ Seminal Vesicle	☐ Base ☐ Mid ☐ Apex ☐ Seminal Vesicle	☐ Base ☐ Mid ☐ Apex ☐ Seminal Vesicle
Main sector which contains the lesion according to PI-RADS v2.1 41-sector diagram (write **one** sector, *e.g.* "PZpl"):				
Biparametric MRI Likert score (1–5):				
Biparametric MRI PI-RADS v2.1 score (1–5):				
**RE-ASSESS, TAKING INTO ACCOUNT INFORMATION FROM DCE SEQUENCE (MPMRI):**				
Multiparametric MRI Likert score (1–5):				
Multiparametric MRI PI-RADS v2.1 score (1–5):				
Target appearance (select **one**):	☐ Focal ☐ Diffuse	☐ Focal ☐ Diffuse	☐ Focal ☐ Diffuse	☐ Focal ☐ Diffuse
Biaxial diameter on sequence where it was largest, in axial plane (mm x mm):				
Sequence used to measure biaxial diameter (select **one**):	☐ T2 ☐ High b ☐ ADC ☐ DCE	☐ T2 ☐ High b ☐ ADC ☐ DCE	☐ T2 ☐ High b ☐ ADC ☐ DCE	☐ T2 ☐ High b ☐ ADC ☐ DCE

For peer review only - http://bmjopen.bmj.com/site/about/guidelines.xhtml

TRIAL IDENTIFIER: PARTICIPANT INITIALS:

PART TWO: NEW DCE-TARGETS ON DYNAMIC CONTRAST ENHANCED SEQUENCE

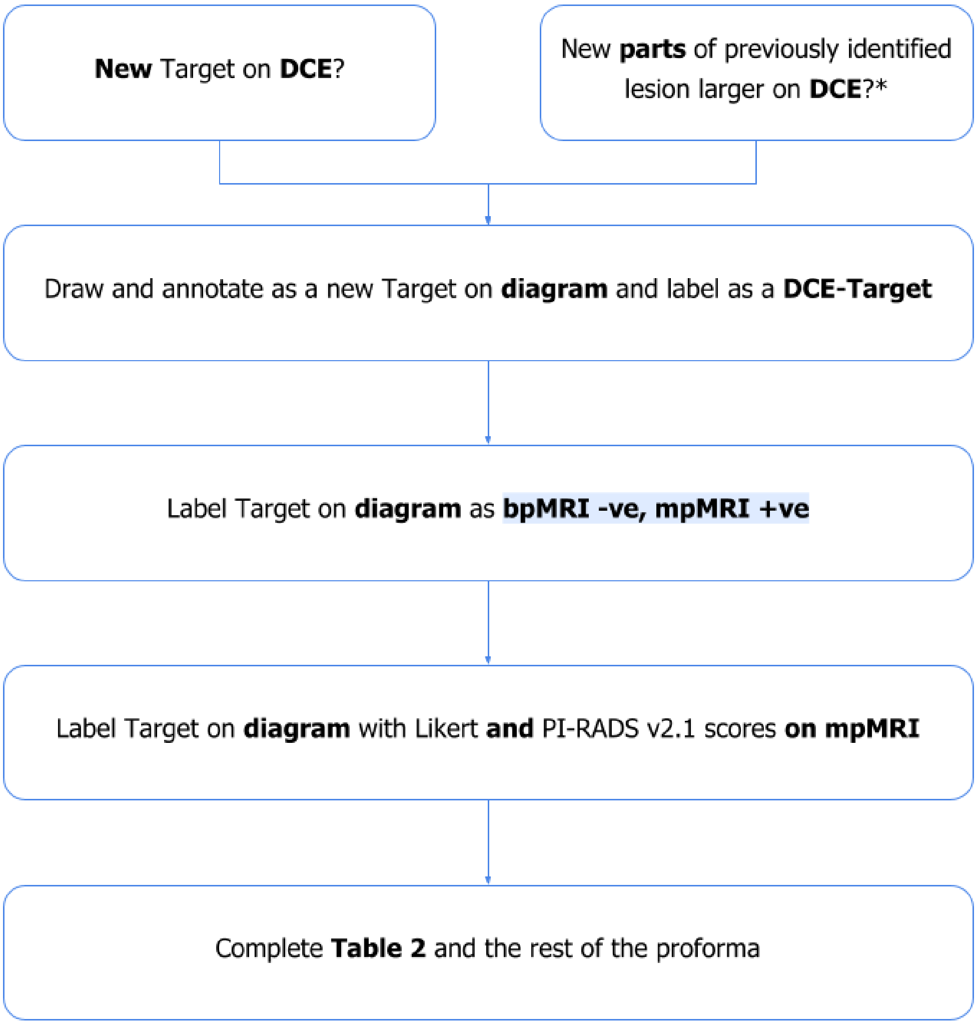

Please note: this is a **subjective decision** by the radiologist as to whether new parts of an existing lesion on bpMRI would need to be declared as **a new target** in order **not to be missed on biopsy**. A clear example of when to declare a new DCE-Target would be if the non-overlapping part of the lesion on DCE crosses into a new sector on the PI-RADSv2.1 sector diagram

---

5. Any new targets should be labelled **DCE-Target-x.**
   a. The first new, most suspicious, target should be **DCE-Target-1**. The second if applicable, **DCE-Target-2** and so on.
6. A maximum of **4 new targets** can be drawn on this report (**Report 2**).
   a. Thus, a maximum of **8 targets** can be drawn in total (4 carried over from **Report 1** and 4 new DCE targets).
7. **On the diagram on Page 2, every** lesion drawn must have the following marked and labelled:
   a. Target number
   b. bpMRI status (positive or negative)
   c. mpMRI status (positive or negative)
   d. Likert score for mpMRI
   e. PI-RADS v2.1 score for mpMRI

   e.g. DCE-Target-1. bpMRI negative. mpMRI positive. Likert 4. PI-RADS 2.

8. Then complete **Table 2** and the rest of the MRI proforma.

For peer review only - http://bmjopen.bmj.com/site/about/guidelines.xhtml

TRIAL IDENTIFIER: PARTICIPANT INITIALS:

Table 2. Information from Targets identified **ONLY** by DCE, which were **not** identified on the **biparametric MRI** (if applicable). If there are no DCE-Targets then leave Table 2 blank & move onto overall patient Likert & PI-RADs scores):

**TARGET SPECIFIC INFORMATION**	DCE-TARGET 1	DCE-TARGET 2	DCE-TARGET 3	DCE-TARGET 4
DCE-Target (select if **new** lesion or **part of existing lesion bigger on DCE**):	☐ New<br>☐ Existing	☐ New<br>☐ Existing	☐ New<br>☐ Existing	☐ New<br>☐ Existing
Location of suspicious area(s) (select **one**):	☐ Right<br>☐ Left<br>☐ Bilateral	☐ Right<br>☐ Left<br>☐ Bilateral	☐ Right<br>☐ Left<br>☐ Bilateral	☐ Right<br>☐ Left<br>☐ Bilateral
Location in prostate according to PI-RADS v2.1 41-sector diagram (select **the one main** location which contains the target):	☐ Base<br>☐ Mid<br>☐ Apex<br>☐ Seminal Vesicle	☐ Base<br>☐ Mid<br>☐ Apex<br>☐ Seminal Vesicle	☐ Base<br>☐ Mid<br>☐ Apex<br>☐ Seminal Vesicle	☐ Base<br>☐ Mid<br>☐ Apex<br>☐ Seminal Vesicle
Main sector which contains the lesion according to PI-RADS v2.1 41-sector diagram (write **one**, *e.g.* "PZpl"):				
Multiparametric MRI Likert score (1–5):				
Multiparametric MRI PI-RADS v2.1 score (1–5):				
Target appearance (select **one**):	☐ Focal<br>☐ Diffuse	☐ Focal<br>☐ Diffuse	☐ Focal<br>☐ Diffuse	☐ Focal<br>☐ Diffuse
Biaxial diameter on dominant sequence in axial plane (mm x mm):				
Looking back again at the T2W and DWI **only**, is the DCE-target identified here actually visible on the bpMRI?	☐ No<br>☐ Yes	☐ No<br>☐ Yes	☐ No<br>☐ Yes	☐ No<br>☐ Yes
If you answered **Yes**, please specify whether the lesion was missed on 1st look ***or*** whether it was seen but scored a 1 or 2 on PI-RADS v2.1 **and** Likert	☐ Missed on 1st look<br>☐ Seen on 1st look but scored a 1 or 2	☐ Missed on 1st look<br>☐ Seen on 1st look but scored a 1 or 2	☐ Missed on 1st look<br>☐ Seen on 1st look but scored a 1 or 2	☐ Missed on 1st look<br>☐ Seen on 1st look but scored a 1 or 2

For peer review only

For peer review only - http://bmjopen.bmj.com/site/about/guidelines.xhtml

TRIAL IDENTIFIER: PARTICIPANT INITIALS:

Please complete the **overall scores** regardless of whether there are any Targets identified above:

**Overall patient Likert score**<br>Enter the highest Likert score on **either** biparametric MRI or multiparametric MRI		**Overall patient PI-RADS v2.1 score**<br>Enter the highest PI-RADS v2.1 score on **either** biparametric MRI or multiparametric MRI	

Please note: if a lesion was suspicious on biparametric MRI but **not** suspicious on mpMRI (*i.e.* bpMRI +ve, mpMRI -ve), it should still be biopsied if either the Likert or PI-RADS v2.1 score on bpMRI is ≥ 3. This highest score on either bpMRI or mpMRI should be entered above.

Table 3. Staging information. Complete **only** if a Target has been identified above. Select **one option** each time:

Radiological stage:	☐ T2a ☐ T2b ☐ T2c ☐ T3a ☐ T3b ☐ T4<br>Radiological T3a = unequivocal extracapsular disease
Likelihood of **right**-sided extracapsular spread*:<br>1 = highly **unlikely**, 3 = equivocal, 5 = highly **likely**	☐ 1 ☐ 2 ☐ 3 ☐ 4 ☐ 5
Likelihood of **left**-sided extracapsular spread*:	☐ 1 ☐ 2 ☐ 3 ☐ 4 ☐ 5
Capsular involvement on **DCE**:	☐ No ☐Yes, on **right** ☐Yes, on **left** ☐Yes, on **both sides**
Likelihood of **right** seminal vesicle involvement:	☐ 1 ☐ 2 ☐ 3 ☐ 4 ☐ 5
Likelihood of **left** seminal vesicle involvement:	☐ 1 ☐ 2 ☐ 3 ☐ 4 ☐ 5
Seminal vesicle involvement on **DCE**:	☐ No ☐Yes, on **right** ☐Yes, on **left** ☐Yes, on **both sides**
Likelihood of urethral sphincter involvement:	☐ 1 ☐ 2 ☐ 3 ☐ 4 ☐ 5
Urethral sphincter involvement on **DCE**:	☐ No ☐Yes, on **right** ☐Yes, on **left** ☐Yes, on **both sides**
Likelihood of bladder neck involvement:	☐ 1 ☐ 2 ☐ 3 ☐ 4 ☐ 5
Bladder neck involvement on **DCE**:	☐ No ☐ Yes
Likelihood of rectal involvement:	☐ 1 ☐ 2 ☐ 3 ☐ 4 ☐ 5
Rectal wall involvement on **DCE**:	☐ No ☐ Yes

**See* PI-RADS v2.1 guidelines for examples of features suggestive of extracapsular spread.

For peer review only - http://bmjopen.bmj.com/site/about/guidelines.xhtml
For peer review only

TRIAL IDENTIFIER: PARTICIPANT INITIALS:

MRI Quality. Please **complete** this for all MRIs regardless of whether a Target was identified:

Was there a problem with the quality of the DCE sequence?	☐ Yes			☐ No
If problems with DCE, please specify: Tick **all** that apply	☐ Rectal air	☐ Movement artefact	☐ Prosthesis	☐ Other
If other, please describe:				
Was the quality of the scan sufficient for you to make a diagnostic assessment?	☐ Yes		☐ No	
Based on the quality of the mpMRI scan and your typical practice, would you recommend a repeat multiparametric MRI be performed?	☐ Yes		☐ No	

Biopsy protocol guidelines

It is **mandatory** to follow these recommendations below:

Number of MRI targets	Location of MRI targets in prostate	Number of MRI-targeted biopsy cores	Number of contralateral systematic cores	Total number of biopsy cores
0	If PSA Density is < 0.15ng/ml/ml			0
0	If PSA Density is ≥ 0.15ng/ml/ml, then 12 systematic biopsy cores are taken (6 from each side)			12
1	Unilateral	4	6	10
2	Unilateral	8	6	14
3	Unilateral	12	6	18
4–8	Unilateral	16–32	6	22–38
1	Bilateral (*e.g.* crossing midline)	4	0	4
2	Bilateral	8	0	8
3	Bilateral	12	0	12
4–8	Bilateral	16–32	0	16–32

Note: For 4–8 MRI targets, determine the number of MRI-targeted cores by using the principle of 4 cores per MRI target.

For peer review only - http://bmjopen.bmj.com/site/about/guidelines.xhtml

TRIAL IDENTIFIER: PARTICIPANT INITIALS:

Recommended Biopsy Plan for biopsy operator to follow

The radiologist should now complete this biopsy plan which should be passed directly to the person performing biopsy (if one is required) along with the labelled diagram on Page 2:

Even if radiologists do not typically write biopsy plans, we request they do this here following the protocol in the table above, in order to reduce errors between linking the MRI information to the protocol biopsy plan.

Number of MRI-targets to biopsy with MRI-targeted biopsy:<br>(*Note:* Targets which are only suspicious on bpMRI should still be biopsied. The number of MRI-targets for biopsy therefore includes MRI targets identified only on bpMRI, only on mpMRI or on both bpMRI and mpMRI and on either the Likert scoring system or the PIRADsv2.1 scoring system)	
Total number of MRI-targeted biopsy cores to be taken:<br>(*Note:* 4 biopsy cores should be taken per lesion)	
Total number of systematic biopsy cores to be taken:<br>(*Note:* Systematic cores should be peripheral zone-focused cores)	
Number of systematic cores to be taken from **right** side of prostate:<br>(*Note:* do not take systematic cores from the same side as an MRI target)	
Number of systematic cores to be taken from **left** side of prostate:<br>(*Note:* do not take systematic cores from the same side as an MRI target)	
Total number of systematic and targeted cores to be taken	

Radiologist<br>(Forename, Surname):		Date:	

For peer review only

For peer review only - http://bmjopen.bmj.com/site/about/guidelines.xhtml

Supplementary Appendix 2: Detailed PRIME Biopsy Plans

To be pragmatic and allow results to be generalisable to biopsy practice around the world, biopsies can be performed transperineally (**Figures 1** and **2**) or transrectally (**Figures 3 and 4**) as per local practice. We split this Appendix into these sections, respectively.

If there is an MRI lesion (scores 3, 4 or 5 on *either* Likert or PI-RADS v2.1 scoring systems), then MRI-targeted biopsy and some limited contralateral systematic biopsy should be performed. MRI-targeted biopsy should be performed **first,** with 4 cores per suspicious area. Then the systematic biopsy cores should be taken but avoid taking biopsies from the same side of the prostate that targeted biopsies were taken from.

Table of Contents

Systematic Transperineal Biopsy Schema 2

Non-suspicious MRI but a PSA Density of ≥ 0.15ng/mL/mL scenario 2

Suspicious MRI lesion scenarios 4

Single lesion scenario 4

Bilateral lesions scenario 4

Lesion crossing midline scenario 5

Bilateral diffuse change on Likert scoring scenario 5

New lesion is revealed on DCE sequence scenario 6

New part of existing lesion is revealed on DCE sequence scenario 6

Systematic Transrectal Biopsy Schema 7

Non-suspicious MRI but a PSA Density of ≥ 0.15ng/mL/mL scenario 7

Suspicious MRI lesion scenarios 9

Single lesion scenario 9

Bilateral lesions scenario 10

Lesion crossing midline scenario 11

Bilateral diffuse change on Likert scoring scenario 12

New lesion is revealed on DCE sequence scenario 13

New part of existing lesion is revealed on DCE sequence scenario 14

Summary Biopsy Guidelines 16

For peer review only

For peer review only - http://bmjopen.bmj.com/site/about/guidelines.xhtml

Systematic Transperineal Biopsy Schema

Figures 1 and **2A-F** depict examples of how to perform the systematic biopsy in the **absence** of an MRI lesion and in the **presence** of MRI lesions, respectively.

Non-suspicious MRI but a PSA Density of ≥ 0.15ng/mL/mL scenario

In patients with a **non-suspicious MRI but a PSA Density of ≥ 0.15ng/mL/mL**, 12-core systematic biopsy should be performed (**Figure 1**).

The number of systematic cores that should be taken per patient is **12.**

Systematic biopsy cores are taken from:

- Right anterior zone (2 cores)
- Right mid zone (2 cores)
- Right posterior zone (2 cores)
- Left anterior zone (2 cores)
- Left mid zone (2 cores)
- Left posterior zone (2 cores)

Systematic biopsy cores should be stored and labelled in a way that their **location** can be identified when the pathologist reports the result.

Figure 1. The transperineal biopsy schema for men with a **non-suspicious MRI** (scores 1 or 2 on both Likert and PI-RADS v2.1 scoring systems) *but* a PSA Density of ≥0.15ng/mL/mL, undergoing 12-core systematic biopsy.

For peer review only

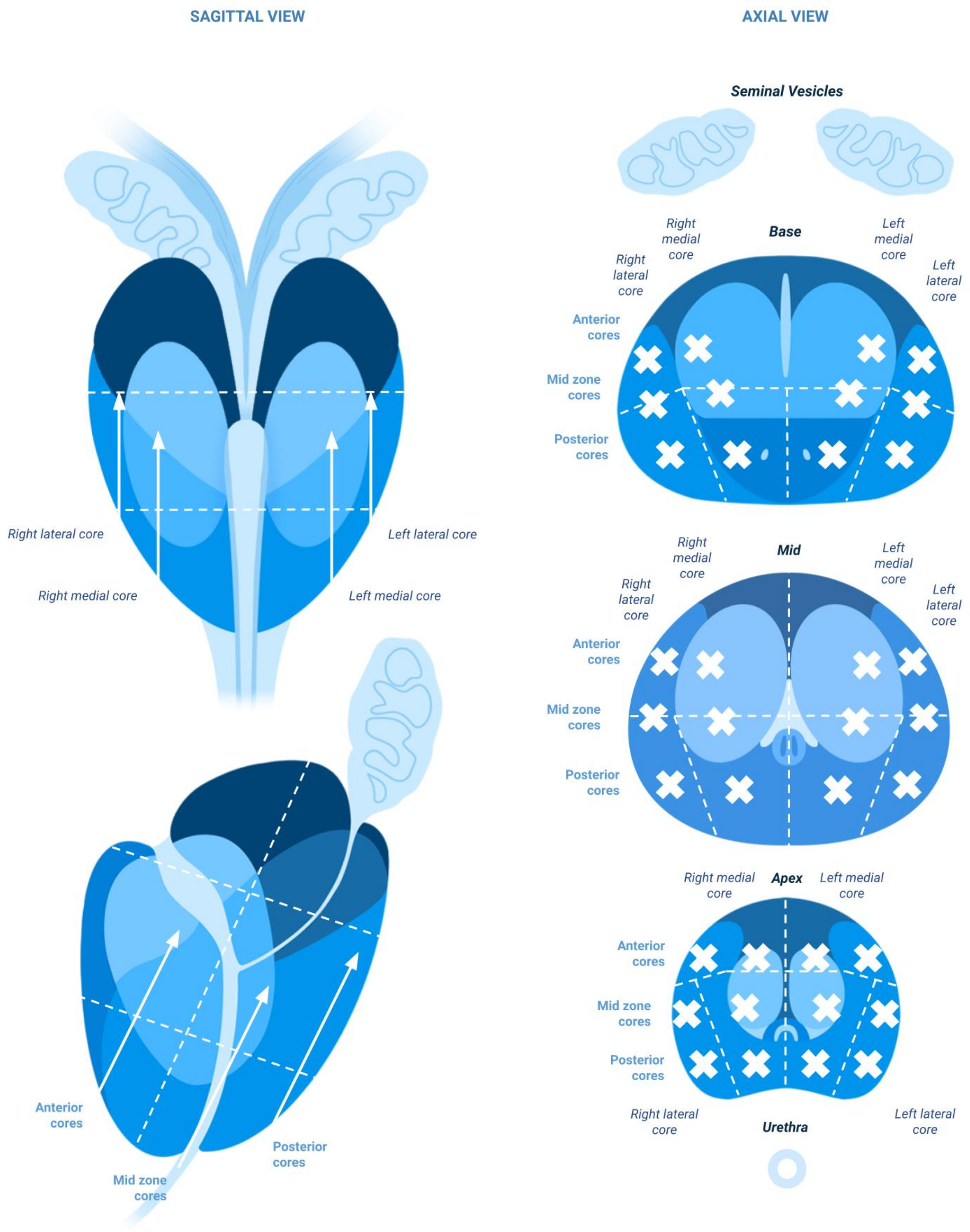

For each pair of biopsies – one core is more lateral, one core is more medial. From anterior—posterior, there are 3 planned rows of biopsies – anterior, mid zone, posterior. Avoid biopsy around the urethra.

Designed by Aqua Asif

Suspicious MRI lesion scenarios

Figure 2. Examples of how to perform transperineal biopsies in patients with an MRI Target (scores 3, 4 or 5 on *either* Likert or PI-RADS v2.1 scoring systems).

2A. Single lesion example.

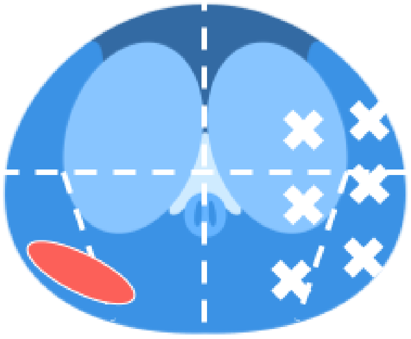

This is a single lesion in the right mid-gland peripheral zone posteromedially (PZ pm) and posterolaterally (PZ pl).

- Take **4 targeted biopsies** from the Target.
- Then take **6 peripheral zone focused biopsies** from the **contralateral** side.
- Do **not** resample the targeted biopsy side.

2B. Bilateral peripheral zone lesions example.

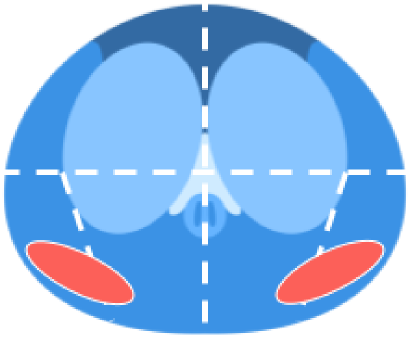

There are **two lesions**: one in right mid-gland, peripheral zone posteromedially and posterolaterally (PZ pm and PZ pl); one in left mid-gland, peripheral zone posteromedially and posterolaterally (PZ pm and PZ pl).

- Take **4 targeted biopsies** from ***each*** Target – *i.e.* **8 targeted biopsies** in **total**.
- **Do not take any systematic biopsies** as targeted biopsies are taken from both sides of the prostate.

Designed by Aqua Asif

For peer review only

2C. Lesion crossing midline example.

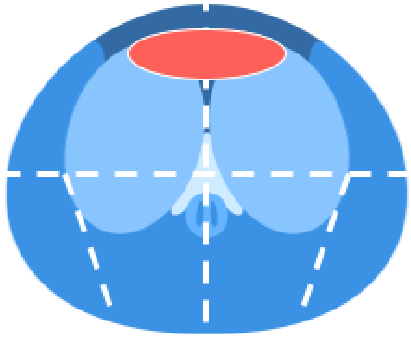

This is one lesion crossing the midline in the mid-gland, anterior fibromuscular stroma.

- Take **4 targeted biopsies** from the Target.
- **Do not take any systematic biopsies** as targeted biopsies are taken from both sides of the prostate.

2D. Bilateral diffuse change on Likert scoring example.

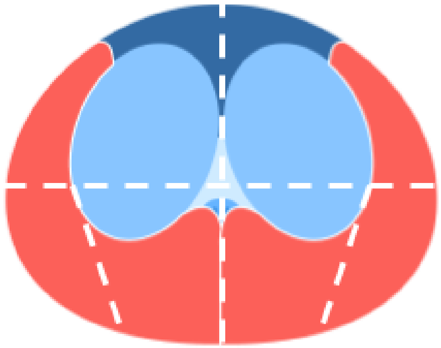

In the circumstance where on Likert scoring, the peripheral zone gives diffuse change, scoring 3 out of 5, arbitrarily **treat each peripheral zone** as a **different Target**.

- Take **4 targeted biopsies** from *each half* of the peripheral zone – *i.e.* **8 biopsies** in total.
- **Do not take any systematic biopsies** as targeted biopsies are taken from both sides of the prostate.

2E. A new lesion is revealed on DCE sequence example.

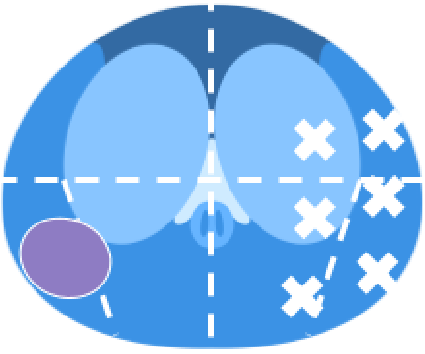

This is one lesion in the right mid-gland, peripheral zone posterolaterally. This **new Target** was specifically *not* suspicious (scored 1 or 2 on both Likert and PI-RADS v2.1) on bpMRI sequences (T2W and DWI). However, when the contrast sequence is revealed, the lesion appears to be suspicious (scored 3, 4 or 5 on Likert) on the dynamic contrast-enhanced (DCE) sequence than on the bpMRI.

- Thus, label the **new lesion** as a **DCE-Target**.
- Take **4 targeted biopsies** from **DCE-Target-1**.
- Then take **6 peripheral zone focused biopsies** from the **contralateral** side of the prostate.
- Do **not** resample the targeted biopsy side.

2F. A new **part** of an *existing* lesion is revealed on DCE sequence example.

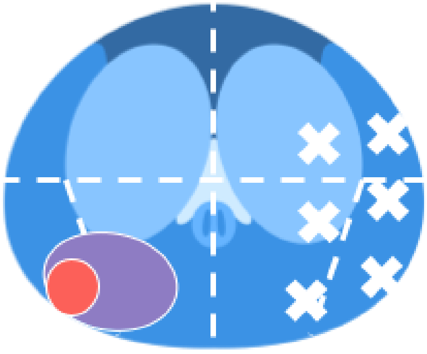

There are two lesions in this example. **Target 1** (**red**) was suspicious on **both** bpMRI and mpMRI. It is in the right mid-gland, peripheral zone, posterolaterally (PZ pl). It scores Likert 4 and PI-RADS v2.1 4.

However, when the contrast sequence is revealed, this lesion appears to be larger on the DCE sequence than on bpMRI. The part of the lesion that is **non-overlapping** would **not** have been

Designed by Aqua Asif
For peer review only - http://bmjopen.bmj.com/site/about/guidelines.xhtml

target biopsied if bpMRI alone was used. Thus, the second lesion (the non-overlapping part, **purple**) is called **DCE Target 1**. It is in the right mid-gland, peripheral zone, posteromedially (PZ pm).

Thus, the instructions are as follows in this instance:

- Take **4 targeted biopsies** from **Target 1**.
- Take **4 targeted biopsies** from **DCE Target 1**.
- Take **6 peripheral zone focused biopsies** from the **contralateral** side of the prostate.
- Do **not** resample the targeted biopsy side.

Systematic Transrectal Biopsy Schema

Figures 3 and **4** depict examples of how to perform the systematic biopsy in the **absence** of an MRI lesion and in the **presence** of MRI lesions, respectively.

Non-suspicious MRI but a PSA Density of ≥ 0.15ng/mL/mL scenario

In patients with a **non-suspicious MRI but a PSA Density of ≥ 0.15ng/mL/mL**, 12-core systematic biopsy should be performed (**Figure 3**).

If performing biopsies transrectally, systematic biopsy cores should be taken from:

- Right base (2 cores)
- Right mid gland (2 cores)
- Right apex (2 cores)
- Left base (2 cores)
- Left mid gland (2 cores)
- Left apex (2 cores)

Systematic biopsy cores should be stored and labelled in a way that their location can be identified when the pathologist reports the result.

The 12 systematic biopsies **should be focused on the peripheral zone.** The urethra should be avoided.

For peer review only

Figure 3. The transrectal biopsy schema for men with a **non-suspicious MRI** (scores 1 or 2 on both Likert and PI-RADS v2.1 scoring systems) *but* a PSA Density of ≥0.15ng/mL/mL, undergoing 12-core systematic biopsy.

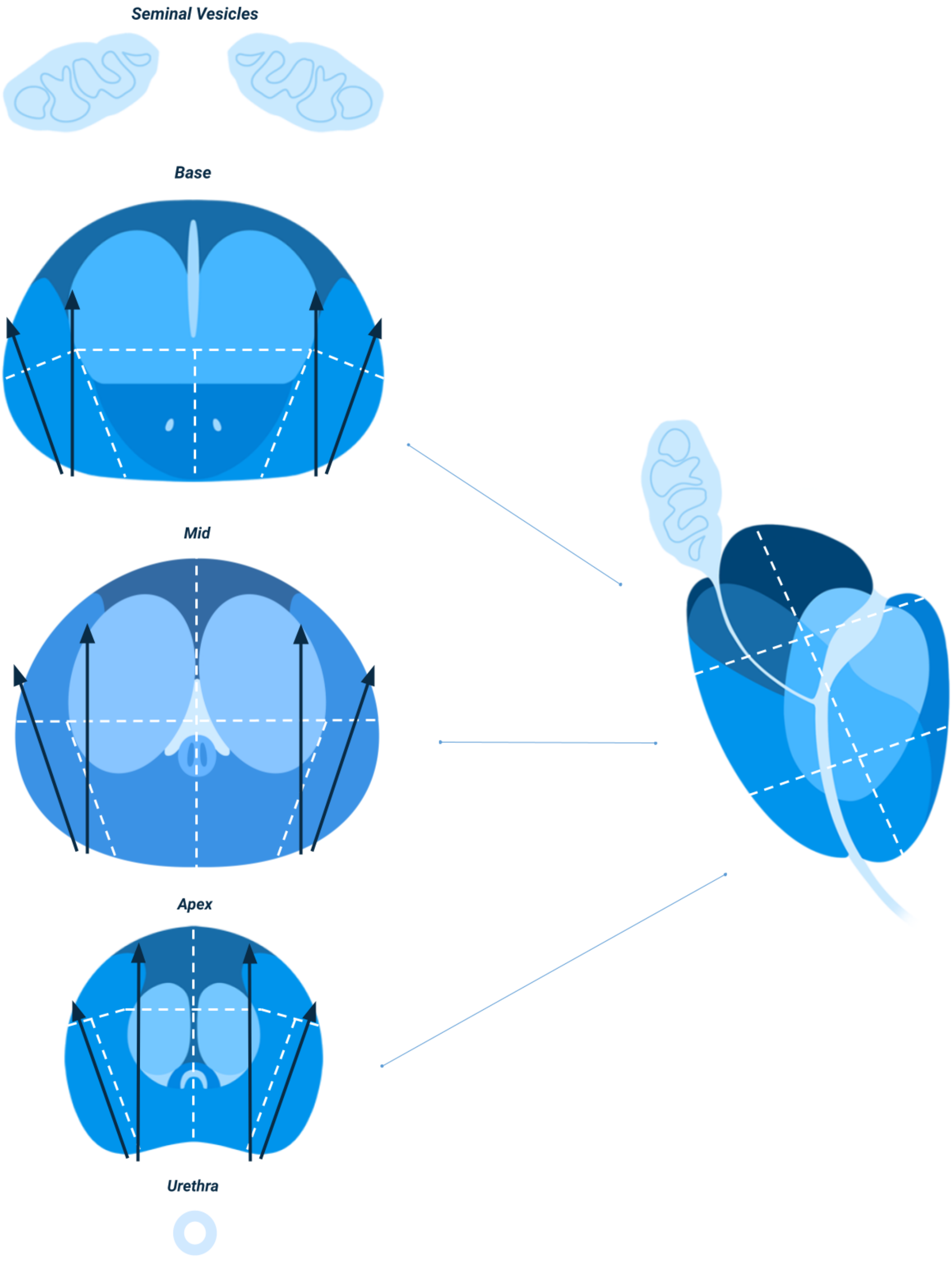

Designed by Aqua Asif

Suspicious MRI lesion scenarios

Figure 4. Examples of how to perform transrectal biopsies in patients with an MRI Target (scores 3, 4 or 5 on *either* Likert or PI-RADS v2.1 scoring systems).

4A. Single lesion example.

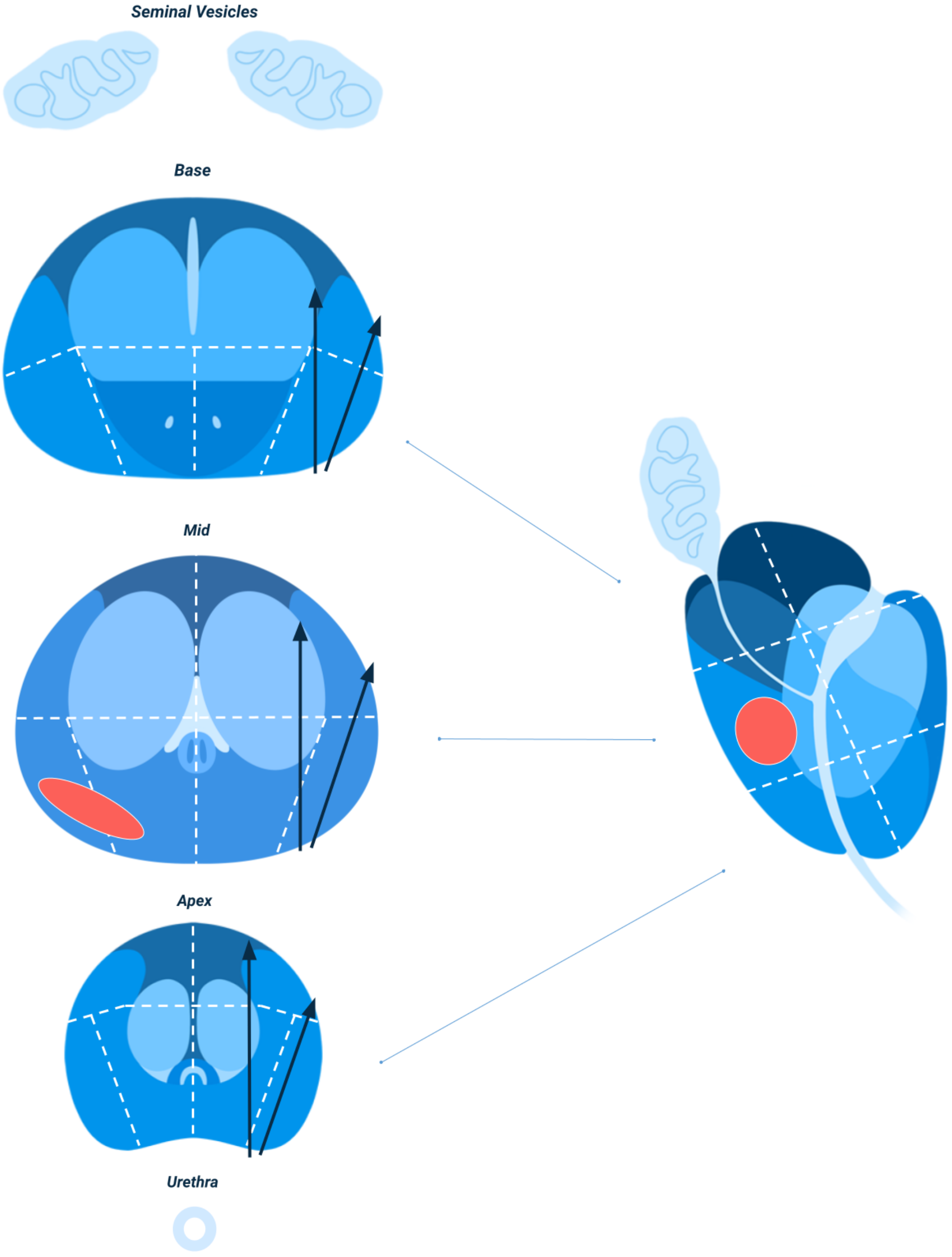

This is a single lesion in the right mid-gland peripheral zone posteromedially (PZ pm) and posterolaterally (PZ pl).

- Take **4 targeted biopsies** from the Target.
- Then take **6 peripheral zone focused biopsies** from the **contralateral** side.

- Do **not** resample the targeted biopsy side.

4B. Bilateral peripheral zone lesions example.

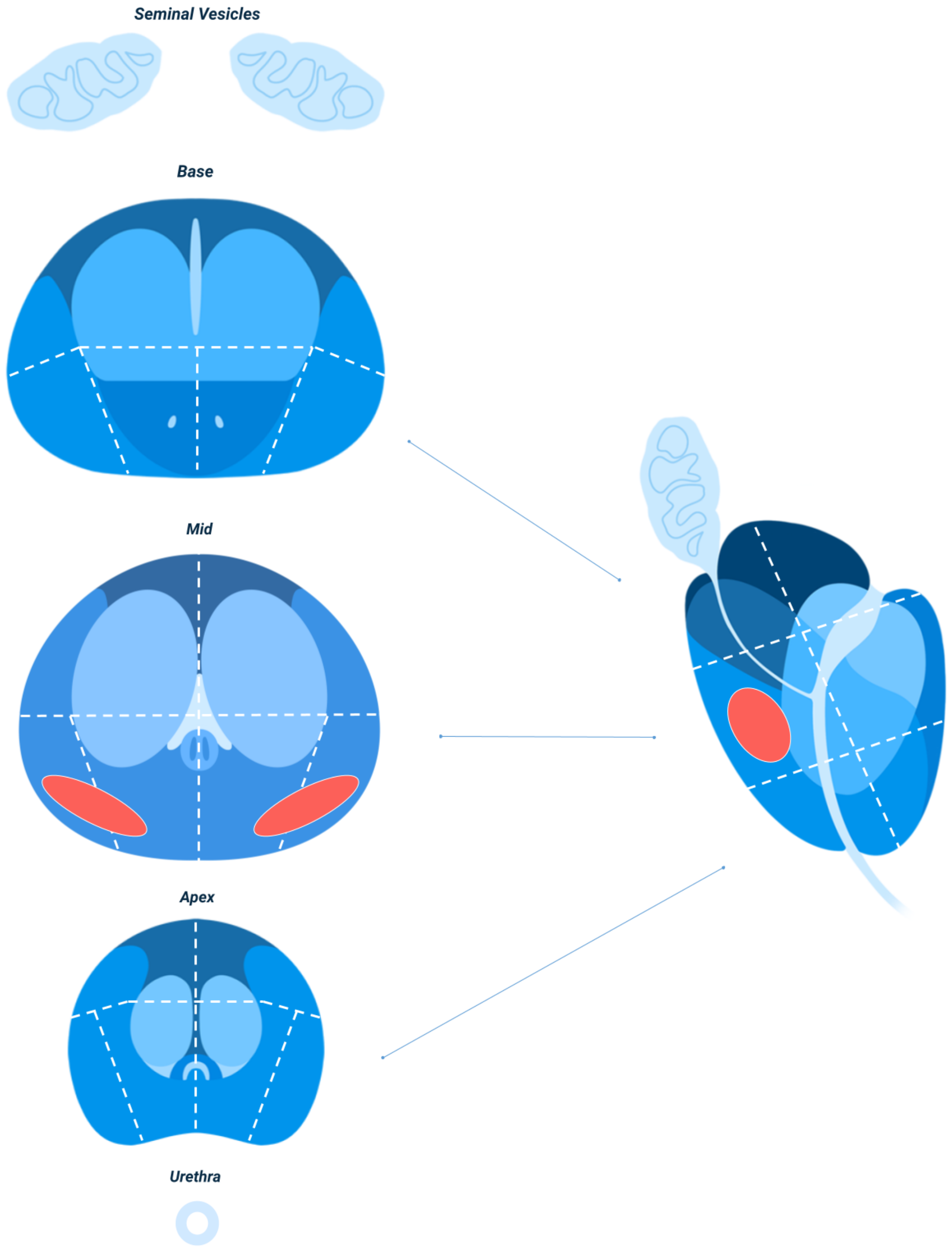

There are **two lesions**: one in right mid-gland, peripheral zone posteromedially and posterolaterally (PZ pm and PZ pl); one in left mid-gland, peripheral zone posteromedially and posterolaterally (PZ pm and PZ pl).

- Take **4 targeted biopsies** from ***each*** Target – *i.e.* **8 targeted biopsies** in **total**.

- **Do not take any systematic biopsies** as targeted biopsies are taken from both sides of the prostate.

4C. Lesion crossing midline example.

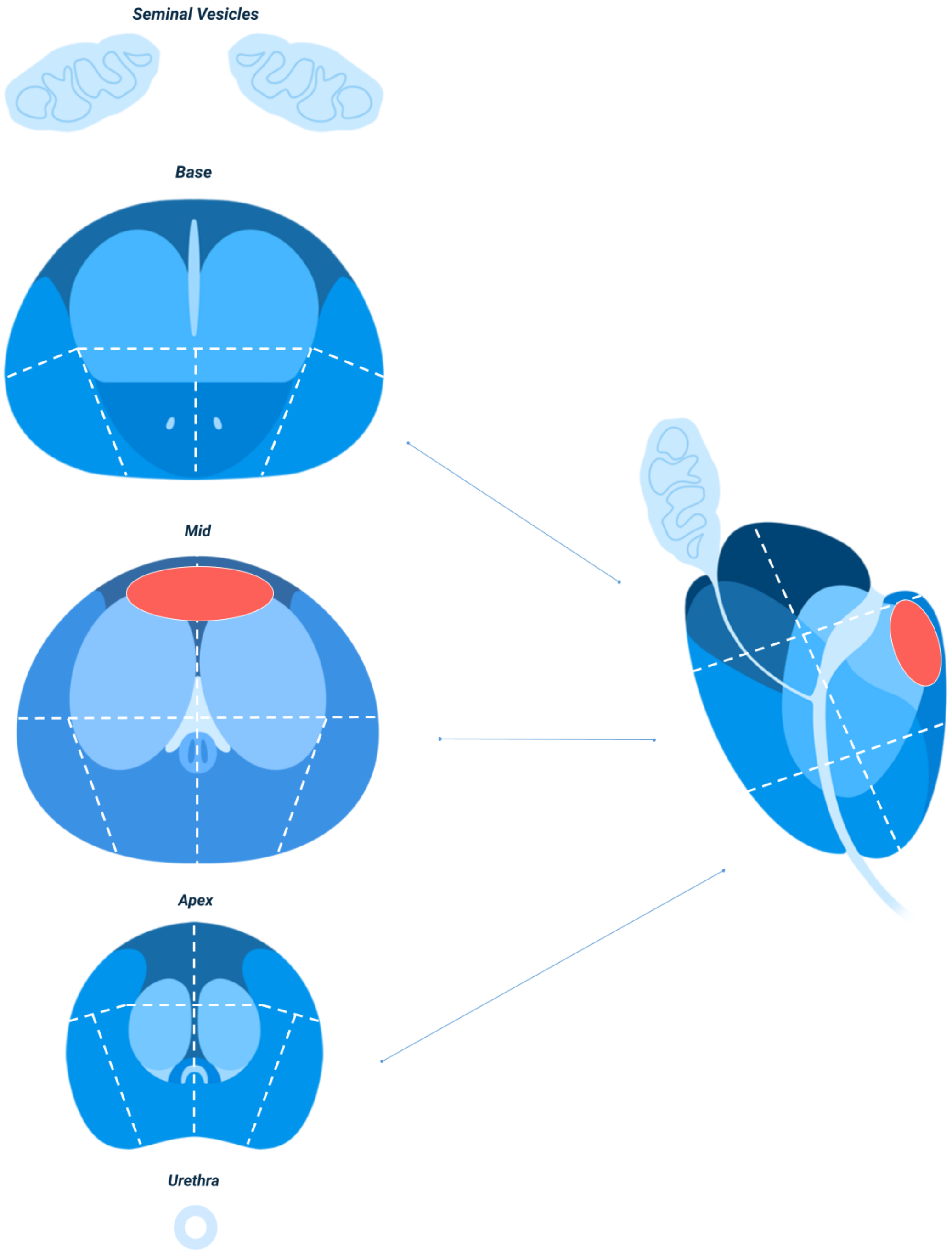

This is one lesion crossing the midline in the mid-gland, anterior fibromuscular stroma.

- Take **4 targeted biopsies** from the Target.

- **Do not take any systematic biopsies** as targeted biopsies are taken from both sides of the prostate.

4D. Bilateral diffuse change on Likert scoring example.

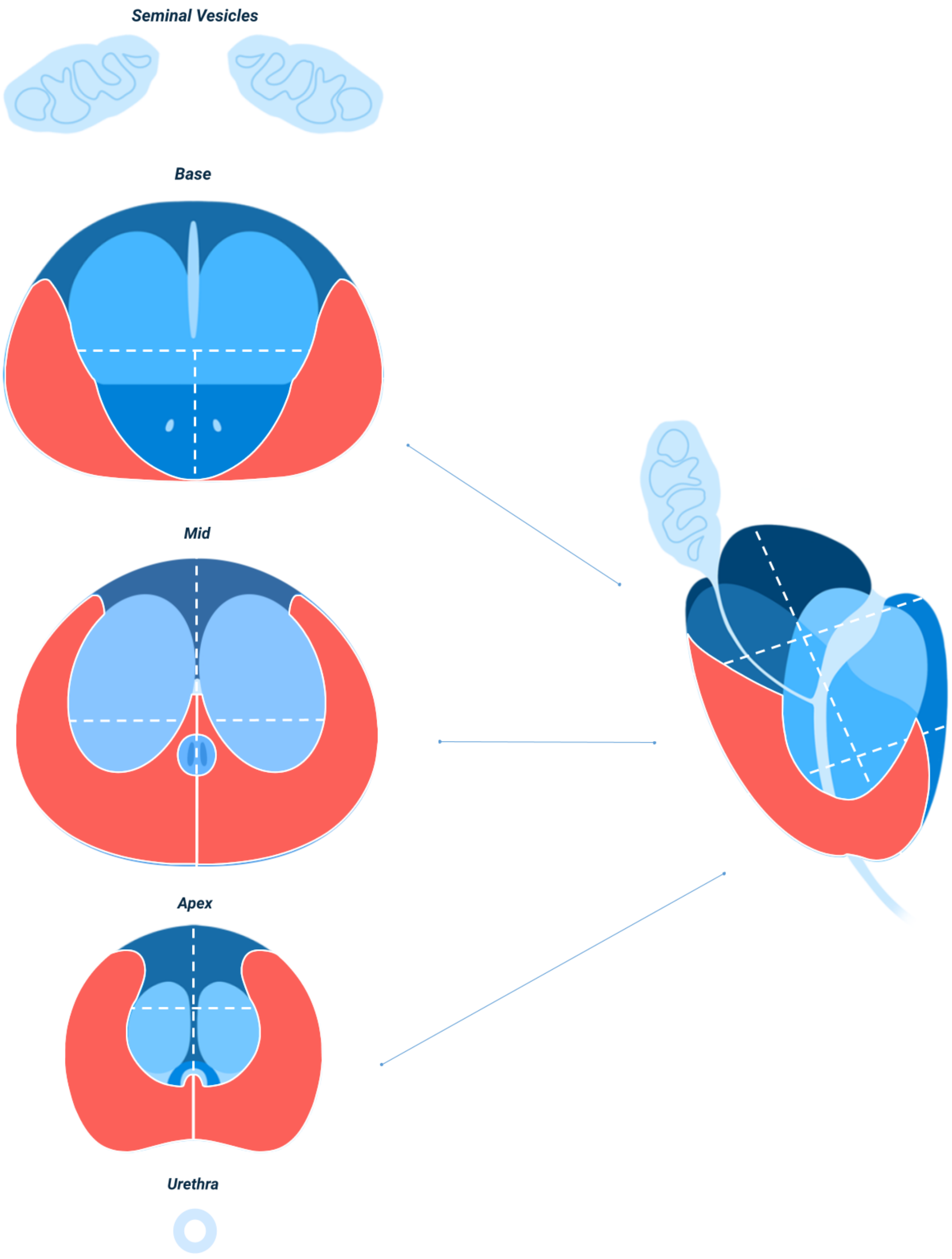

In the circumstance where on Likert scoring, the peripheral zone gives diffuse change, scoring 3 out of 5, arbitrarily **treat each peripheral zone** as a **different Target**.

Designed by Aqua Asif

- Take **4 targeted biopsies** from *each half* of the peripheral zone – *i.e.* **8 biopsies** in total.
- **Do not take any systematic biopsies** as targeted biopsies are taken from both sides of the prostate.

4E. A new lesion is revealed on DCE sequence example.

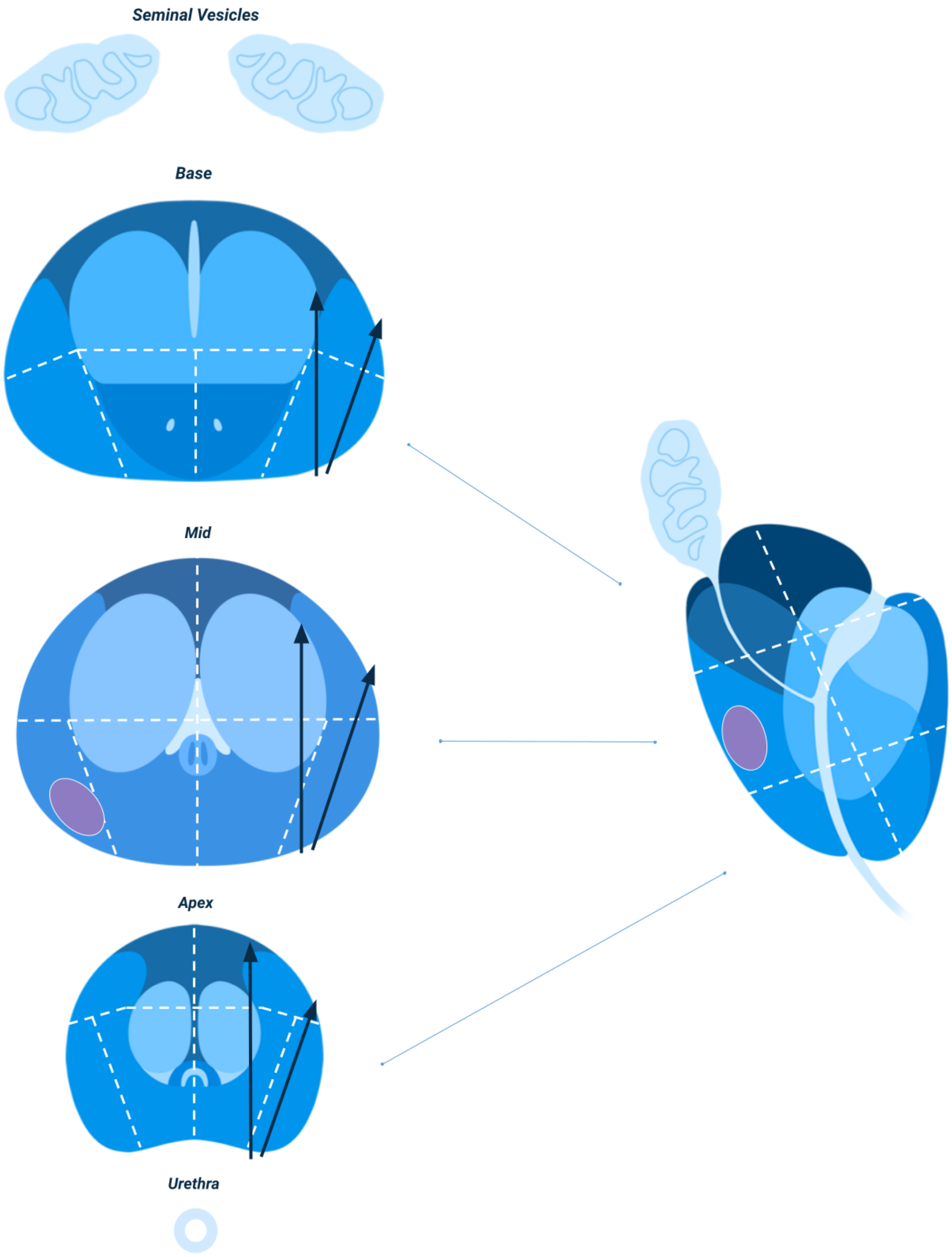

This is one lesion in the right mid-gland, peripheral zone posterolaterally. This **new Target** was specifically *not* suspicious (scored 1 or 2 on both Likert and PI-RADS v2.1) on bpMRI sequences (T2W and DWI). However, when the contrast sequence is revealed, the lesion

appears to be suspicious (scored 3, 4 or 5 on Likert) on the dynamic contrast-enhanced (DCE) sequence than on the bpMRI.

- Thus, label the **new lesion** as a **DCE-Target**.
- Take **4 targeted biopsies** from **DCE-Target-1**.
- Then take **6 peripheral zone focused biopsies** from the **contralateral** side of the prostate.
- Do **not** resample the targeted biopsy side.

4F. A new **part** of an *existing* lesion is revealed on DCE sequence example.

There are two lesions in this example. **Target 1** (**red**) was suspicious on **both** bpMRI and mpMRI. It is in the right mid-gland, peripheral zone, posterolaterally (PZ pl). It scores Likert 4 and PI-RADS v2.1 4.

However, when the contrast sequence is revealed, this lesion appears to be larger on the DCE sequence than on bpMRI. The part of the lesion that is **non-overlapping** would **not** have been target biopsied if bpMRI alone was used. Thus, the second lesion (the non-overlapping part, **purple**) is called **DCE Target 1**. It is in the right mid-gland, peripheral zone, posteromedially (PZ pm).

Thus, the instructions are as follows in this instance:

- Take **4 targeted biopsies** from **Target 1**.
- Take **4 targeted biopsies** from **DCE Target 1**.
- Take **6 peripheral zone focused biopsies** from the **contralateral** side of the prostate.
- Do **not** resample the targeted biopsy side.

For peer review only

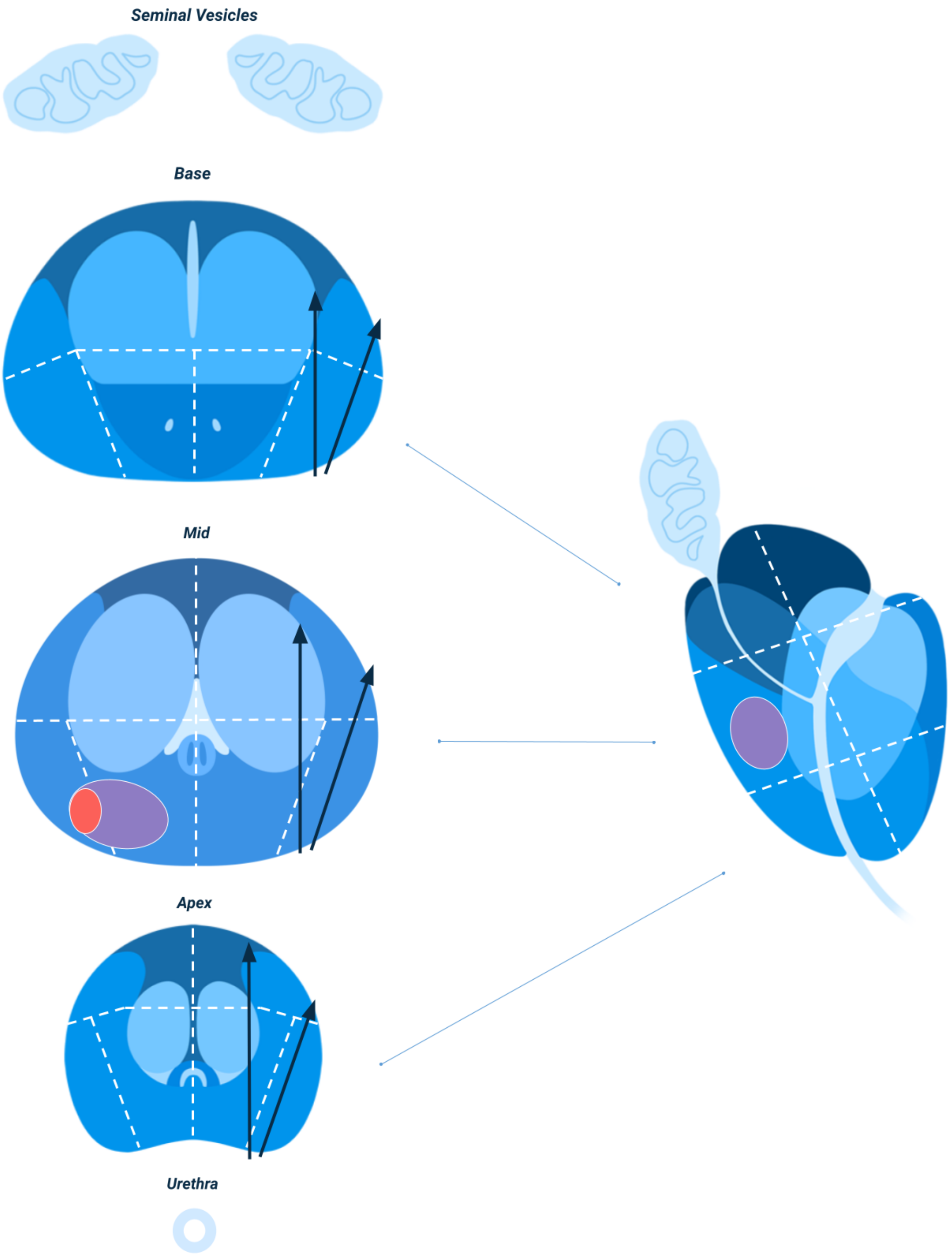

Designed by Aqua Asif

Summary Biopsy Guidelines

Number of MRI targets	Location of MRI targets in prostate	Number of MRI-targeted biopsy cores	Number of contralateral systematic cores	Total number of biopsy cores
**0**	If PSA Density is < 0.15ng/ml/ml			0
**0**	If PSA Density is ≥ 0.15ng/ml/ml, then 12 systematic biopsy cores are taken (6 from each side)			12
**1**	Unilateral	4	6	10
**2**	Unilateral	8	6	14
**3**	Unilateral	12	6	18
**4–8**	Unilateral	16–32	6	22–38
**1**	Bilateral (*e.g.* crossing midline)	4	0	4
**2**	Bilateral	8	0	8
**3**	Bilateral	12	0	12
**4–8**	Bilateral	16–32	0	16–32

For peer review only

Designed by Aqua Asif

Please present on local headed paper

REC Number:
IRAS Number: 282789

Subject Identification: ____________________
Study Number ;__________________________

CONSENT FORM

Title of Project: PRostate **I**maging using **M**RI +/- contrast **E**nhancement (PRIME)

Name of Researcher:

Please initial box

1. I confirm that I have read and understand the information sheet dated................... (version...........) for the above study. I have had the opportunity to consider the information, ask questions and have had these answered satisfactorily. ☐

2. I understand that my participation is voluntary and that I am free to withdraw at any time without giving any reason, without my medical care or legal rights being affected. ☐

3. I understand that relevant sections of my medical notes and data collected during the study, may be looked at by individuals from the sponsor of the trial (University College London), responsible persons authorised by the sponsor, from regulatory authorities, from the NHS Trust and from PRIME study researchers who may be outside of my local centre, where it is relevant to my taking part in this research. I give permission for these individuals to have access to my records. ☐

4. I agree to my GP being informed of my participation in the study. ☐

5. I give my permission for the PRIME research team at my local centre to hold identifiable information such as my name, address, date of birth, email address, mobile phone number, NHS number or other applicable hospital identifier. I understand this may be used to collect longer term healthcare information on me from national records, such as the Office for National Statistics, NHS Digital, Public Health England, and other applicable NHS information systems, or other relevant national databases. This data may be linked to my data from the PRIME study in future research. ☐

For peer review only - http://bmjopen.bmj.com/site/about/guidelines.xhtml

6. I give permission to be contacted for further information in the future. This may include requests to complete quality of life questionnaires or for ascertaining future health status, if required. ☐

7. I give permission for my samples to be sent to UCL by courier for quality control assessments. ☐

8. I give permission for my anonymized data to be used for teaching and educational purposes for healthcare professionals. ☐

9. I give my permission for my anonymized data to be shared with affiliated researchers and commercial partners who are approved by the PRIME study team for future research if deemed suitable by the PRIME Chief Investigator ☐

10. I give my permission to be approached for other studies in the future that may be relevant to me, and for my study data collected in PRIME to be used for this purpose. ☐

11. I agree to take part in the above study and to complete study procedures outlined in the patient information sheet provided. ☐

All boxes above must be initialed for consent to be valid

______________________	___________	________________
Name of Participant	Date	Signature

______________________	___________	________________
Name of Person taking consent	Date	Signature

When completed: 1 for participant; 1 (original) for researcher site file; 1 to be kept in medical notes.

For peer review only - http://bmjopen.bmj.com/site/about/guidelines.xhtml

PLACE HOSPITAL LETTER HEAD ON FIRST PAGE ONLY.

Affix patient sticker / details here

Version 3.0 8 June 2021

This is the Patient Information Sheet for a Health Research Study called PRIME

Study Short Title: Prostate Imaging using MRI +/- contrast Enhancement

Study acronym: **PRIME**

Chief Investigator: Mr Veeru Kasivisvanathan

UCL Reference number: 135819

REC Reference number: 21/WM/0091

IRAS Number: 282789

We would like to invite you to take part in our research study. Before you decide we would like you to understand why you are being invited, why the research is being done and what it would involve for you. One of our team will go through the information sheet with you and answer any questions you have. Talk to others about the study if you wish.

Part 1 tells you the purpose of this study and what will happen to you if you take part. Part 2 gives you more detailed information about the conduct of the study.

Ask us if there is anything that is not clear. Please take as much time as you need to consider the study.

For peer review only - http://bmjopen.bmj.com/site/about/guidelines.xhtml

Part 1

1. Why have I been invited?

You are being invited because you may require further investigation of your prostate with an MRI scan and / or a prostate biopsy. You have not been diagnosed with cancer but an MRI and / or a biopsy may be required to establish whether you do or do not have cancer. The clinical Urology team that you have been referred to has informed us that you may be eligible for this study.

2. What is the purpose of the study?

The standard way of diagnosing prostate cancer is to carry out a multiparametric prostate MRI scan and prostate biopsy. This type of MRI scan normally involves an injection of contrast into one of your veins.

Another type of MRI scan (biparametric) can be performed that does not require contrast, and therefore does not require the insertion of a cannula. We currently do not know for certain whether using this type of MRI will allow us to detect the same, more or less prostate cancer than if we use the standard (multiparametric) type of MRI. Current evidence supports the idea that using biparametric MRI may detect a similar amount of cancer to when it is not used but one advantage is it may allow a man to have a scan without contrast.

The main purpose of this study is to assess if biparametric MRI can provide similar information to multiparametric MRI. You will undergo a multiparametric MRI with a contrast injection, which is the typical method used for investigating the prostate for the presence of cancer. The doctor reviewing your scan will be asked to review the MRI scan in a particular order so that they can tell whether the additional information given by the contrast injection helps identifies prostate cancer.

If there is a suspicious area in the prostate on the MRI, a few biopsies can be directed at where the suspicious area is thought to be, also using an ultrasound probe in the back passage. If there is no suspicious area on the MRI and if you at low risk of harbouring cancer, which occurs in about 30% of men, then no biopsy will be taken at all.

3. Do I have to take part?

It is up to you to decide to join the study. We will describe the study and go through this information sheet. If you agree to take part, we will then ask you to sign a consent form. You are free to withdraw at any time without giving a reason. This will not affect the standard of care you receive.

4. What are the benefits to me of taking part in this study?

The healthcare team carrying out the tests in the study are experienced in carrying out and interpreting these tests. The research team will ensure your tests are carried out as quickly as possible and will be a point of contact for you should you have any concerns or questions.

The information we get from this study will help improve the diagnosis of prostate cancer for men in the future.

5. What type of study is this?

This is a study evaluating the accuracy of diagnostic tests. In this trial, you will have the same investigation (multiparametric MRI) as your hospital normally does to investigate the prostate, but the doctor interpreting your scan will be asked to report this in a particular order. The full information will be available to the doctors as it would normally be available if you were not taking part in the study.

You will be required to attend a screening visit with a member of the research team who will spend around 40 minutes explaining what is involved in the study and making sure you are

For peer review only - http://bmjopen.bmj.com/site/about/guidelines.xhtml

eligible for the study. Where possible, all study visits that do not require a journey to the hospital will be performed remotely (e.g. over the phone or video call).

6. What will happen to me if I take part?

After you have attended the screening visit, if you are eligible to take part in the study, you will asked to visit the hospital 2-3 times in total, which is the same as if you were not taking part in the study. After you consent to participating in the study, you will be asked to complete two short questionnaires which will ask about any symptoms related to your prostate that you may be having. These are questionnaires that are typically used as part of routine care. You would only undergo tests that you would normally have as part of routine care if you were not taking part in the study.

If you have not already had a prostate MRI, you will have one within a few weeks after the screening visit. The MRI takes about 40 minutes. Alternatively, it is possible that you are approached for the study after you have had your prostate MRI.

If you have an MRI with a high enough suspicion (MRI Score 3, 4 or 5) you will be booked for a biopsy following the MRI. If the MRI is non-suspicious but you are at high risk of having cancer because of a blood test result, (called your prostate specific antigen density) you will also undergo a prostate biopsy. If you do not need a biopsy (if your MRI is non-suspicious and your prostate specific antigen density is low) then you do not need to undergo a biopsy and we will explain this to you once your MRI results is available.

The biopsy procedure itself takes about 40 minutes and is typically carried out under local or general anaesthetic. Prostate biopsies, which take very small samples of prostate tissue, are taken from the prostate gland and sent to the lab to determine whether there is cancer there or not. If there is a suspicious area on the MRI scan, the MRI information will be used to influence where the biopsies are taken from. Software may be used to transfer additional information from the original MRI onto the screen when the biopsies are taken. In some centres, this would be exactly what you would normally get, and there would be no difference to standard of care. In other centres, their usual practice may be slightly different to this, and you may be required to have a few extra or fewer biopsies than what is typical in your usual centre. After the procedure, we then wait for the results and discuss treatment options with you in clinic at approximately 2-3 weeks after the biopsies.

Please note that the above time frames are suggested time frames and depending on clinical workload within the hospital, the time frame may be shorter or longer. This would be no different than if you were not part of the study.

Being involved in the study does not limit subsequent tests or treatment you may receive. If you do undergo further tests or treatment after the study is complete we may check the results of these on your records. We use the research data we have gathered from your involvement in the study to help us determine how good the diagnostic tests you have had are. We will work with other research teams to do this. We also ask your permission to use research data for teaching and education of other healthcare professionals. After completing the study, we also ask your permission to check your health through national databases. We may also contact you for further information in the future. This may include requests to complete quality of life questionnaires or for ascertaining future health status. All information which is collected about you during the course of the research will be kept strictly confidential, and any information about you which leaves the hospital will have your name and address removed so that you cannot be recognised. Please see Part 2 for further information on this.

7. What data will be collected and use of data

We will need to use information from your medical records for this research project. Your hospital will hold personal identifiable data on you. This information will include information such as age, PSA level, family history of medical conditions such as prostate cancer and examination findings. We allow the PRIME research team at your local site to hold

For peer review only - http://bmjopen.bmj.com/site/about/guidelines.xhtml

identifiable data on you, which will be for 10 years. Longer term data that may be requested from you include information on whether or not you have had further investigations or treatment for prostate problems and what the outcomes of those were as well as quality of life assessments. Non-identifiable data will be stored in the MARVIN database and the database will be transferred and stored at UCL within UCL's data safe haven. You will be given a subject number and a subject identifier, and this will be used on all your study records. The code for this number will be known to the investigators at your site so that the link between your name and the data we hold on the study database is not completely broken. Any paperwork for the study will be kept in locked cupboards, staff access to these cupboards is strictly controlled.

In general, UCL, as a university and a study sponsor, uses personally-identifiable information to conduct research to improve health, care and services. As a publicly-funded organisation, we have to ensure that it is in the public interest when we use personally-identifiable information from people who have agreed to take part in research. This means that when you agree to take part in a research study, we will use your data in the ways needed to conduct and analyse the research study. Your rights to access, change or move your information are limited, as we need to manage your information in specific ways in order for the research to be reliable and accurate. If you withdraw from the study, we will keep the information about you that we have already obtained. To safeguard your rights, we will use the minimum personally-identifiable information possible.

Health and care research should serve the public interest, which means that we have to demonstrate that our research serves the interests of society as a whole. We do this by following the UK Policy Framework for Health and Social Care Research.

All data is managed in line with the Data Protection Act (2018) & General Data Protection Regulations (GDPR).

If you wish to raise a complaint on how we have handled your personal data, you can contact our Data Protection Officer who will investigate the matter. If you are not satisfied with our response or believe we are processing your personal data in a way that is not lawful you can complain to the Information Commissioner's Office (ICO).

UCL Data Protection Officer can be contacted on data-protection@ucl.ac.uk

8. What will I have to do?

You should attend your screening visit and if eligible for the study, await contact from the hospital for further dates of investigations. Unless otherwise advised by a doctor you should carry on with your normal activities and medication. Sometimes before a biopsy your doctor will prescribe you antibiotics and may ask you to stop blood-thinning medications.

You should undergo the necessary tests and biopsy procedures that you are advised to have by your doctor.

You should attend your follow up clinic appointment where we discuss your results. Treatment options will be discussed with you at the results clinic. In total you will typically be required to attend the hospital 2-3 times.

9. What are the alternatives for diagnosis?

An MRI scan and biopsies of the prostate if required are the standard ways in which prostate cancer is diagnosed.

10. What are the possible disadvantages and risks of taking part?

For peer review only - http://bmjopen.bmj.com/site/about/guidelines.xhtml

Being involved in the study is unlikely to expose you to additional risk than if you were not involved in the study but underwent the normal procedures for men referred for further investigation of prostate disease.

Risks of prostate biopsy include:

- Temporary discomfort in the back passage (most men)
- Blood in the urine – up to 2 weeks (most men)
- Blood in the semen – up to 3 months (most men)
- Blood in the back passage – up to 1 week (most men)
- Infection in the blood stream – 1-4 out of 100 men
- Urinary tract infection – 4 out of 100 men
- Urinary retention – 1 out of 100 men
- Adverse reaction to antibiotics – less than 1 in 100 men

Risks of MRI include:

- Discomfort from cannulation
- Allergic reaction:
  - Mild reaction e.g. rash, itching – less than 1 in 250 men
  - Moderate reaction e.g. nausea, omitting – less than 1 in 2000 men
  - Severe reaction e.g. breathing problems – less than 1 in 10000 men

In some centres, you would receive exactly what you would normally get outside of the study. In other centres, their usual practice may be slightly different to this, and you may be required to have a few extra or fewer biopsies than what is typical in your usual centre. However, there is no evidence that a few extra or fewer biopsies within the proposed study would result in additional adverse effects for you.

Before participating you should consider if this will affect any insurance you have and seek advice if necessary.

11. What should you do if you experience any problems during the study?

Though the risk is very low, if you do experience any possible signs of infection after biopsies (fevers and feeling generally unwell) then you should urgently go to your nearest accident and emergency department which is open 24 hours a day. If you are not able to pass urine you should urgently go to your nearest accident and emergency. If you are unsure about what to do or have any questions please call 0207 679 9092 between 9am and 5pm and a member of our team may be able to offer you advice or direct you to someone who can offer you advice.

If you experience any other untoward complication or need to see a doctor we would like to know about this so please let us know on the above number as soon as possible after the complication. For any emergencies at any time or if you are unable to contact a member of the research team, please attend your local accident and emergency for an assessment.

12. What happens when the research study stops?

Once the results of the MRI and, if required, biopsy are available you will be called to clinic to discuss them. Once a treatment decision is made, most men in the study will complete the study and your normal clinical team will continue to look after your care. Being part of the study does not prevent you from undergoing any further diagnostic test or treatment that your clinician would normally recommend.

13. What if there is a problem?

Any complaint about the way you have been dealt with during the clinical study or any possible harm you might suffer will be addressed. The detailed information concerning this is given in Part 2 of this information sheet. If you have any concerns or complaints you should contact a member of the research team in the first instance.

For peer review only - http://bmjopen.bmj.com/site/about/guidelines.xhtml
For peer review only

14. Will my taking part in the study be kept confidential?
Yes. We will follow ethical and legal practice and all information about you will be handled in confidence. The details are included in Part 2.

15. Will any costs I incur in travelling to study visits be reimbursed to me?
Reasonable transport costs that you incur to get to additional study visits (if any further visits are necessary) that are above what you would normally need if you were not part of the study may be reimbursed. Please contact your local study nurse or doctor or the Study Coordinator (details below) for further information on claiming.

16. Contact Details
If you have any further questions or need any further information please do no hesitate to contact the research team.

or the **Chief Investigator:**
Mr Veeru Kasivisvanathan MBBS BSc FRCS MSc PGCert PhD
Division of Surgery and Interventional Science, University College London
3rd floor Charles Bell House, 43-45 Foley Street
London W1W 7TS
T: 0207 679 9092 F: 0207 679 9511 E: veeru.kasi@ucl.ac.uk

This completes Part 1 of the Information Sheet.

If the information in Part 1 has interested you and you are considering participation, please read the additional information in Part 2 before making any decision.

For peer review only

For peer review only - http://bmjopen.bmj.com/site/about/guidelines.xhtml

Part 2

17. What if relevant new information becomes available?
Sometimes we get new information about the procedures being studied. If this happens and we feel it is important to your participation in the study, we will tell you about it and discuss whether you want to or should continue in the study. If you decide not to carry on, we will make arrangements for your care to continue. If you decide to continue in the study, we may ask you to sign an updated consent form. You can also find out if there is any new relevant information by visiting www.ncita.org.uk.

18. What will happen if I don't want to carry on with the study?
You can withdraw from the study at any point and it will not affect the care that you are given. We will use information collected about you up until your withdrawal. Kindly keep in contact with us to let us know your progress.

19. What if there is a problem?
If you have a concern about any aspect of this study, you should ask to speak to your research team who will do their best to answer your questions, please see point number 24. You can also contact the Chief Investigators on the number or address given earlier in this document. If you wish to complain by other means or have any concerns about any aspect of the way you have been approached or treated by members of staff or about any side effects (adverse events) you may have experienced due to your participation in the clinical study, the normal National Health Service complaints mechanisms are available to you. You can contact the hospital Patient Advice and Liaison Service (PALS). Your local PALS team can be contacted at the following number:

Local team to insert contact details of local PALS office here:

You can also contact NHS helpline at 111 which will be able to give you the number of your local PALS office if you are concerned.

Every care will be taken in the course of this clinical study. However in the unlikely event that you are injured by taking part, compensation may be available.

If you suspect that the injury is the result of the Sponsor's (University College London) or the hospital's negligence, then you may be able to claim compensation. After discussing with your study doctor, please make the claim in writing to Mr Veeru Kasivisvanathan who is the Chief Investigator for the clinical study and is based at University College London. The Chief Investigator will then pass the claim to the Sponsor's Insurers, via the Sponsor's office. You may have to bear the costs of the legal action initially, and you should consult a lawyer about this.

20. Will my taking part in this study be kept confidential?
If you consent to take part in this study, the records obtained while you are in this study as well as related health records will remain strictly confidential at all times. The information will be held securely on paper and electronically at your treating hospital under the provisions of the Data Protection Act 2018 and the General Data Protection Regulations 2018. The information will be made available to persons in the clinical and research teams treating you. Your name and personal details will not be passed to anyone else outside the clinical team, research team or the Sponsor, who is not involved in the study. No additional samples will be taken specially for research in this study. All The research team may verify results of tests carried out at your local hospital (for example MRI results or prostate biopsy results) by transferring and analysing a small number of samples collected to UCL. samples and information collected will be de-identified to you prior to transfer to UCL, so only non-identifiable data will be transferred to UCL. This includes some pathology glass slides, which will be reviewed at Dr Alex Freeman's laboratory at University College London (UCL), for quality control. Slides sent to UCL will be

For peer review only - http://bmjopen.bmj.com/site/about/guidelines.xhtml

not have your name assigned. Samples will be sent using one of UCL's preferred couriers, for both pick up and return.

Any data stored by the research team outside of your treating hospital will be kept at a secure location and will not contain information that can directly identify you. You will be allocated a study number, which will be used as a code to identify you on all study forms and data. The information will be linked to you so that if we did need to identify you for your safety or to clarify some information we would be able to by using a unique key, which will be known only to your local hospital team.

Your records will be available to people authorised to work on the study but may also need to be made available to people authorised by the Sponsor, which is the organisation responsible for ensuring that the study is carried out correctly. By signing the consent form, you agree to this access for the current study and any further research that may be conducted in relation to it, even if you withdraw from the current study. All will have a duty of confidentiality to you as a research participant.

If you withdraw consent from further study treatment, your data and samples will remain on file and will be included in the final study analysis.

In line with the regulations, at the end of the study your data will be securely archived for 20 years. Arrangements for confidential destruction will then be made.

Anonymised data collected during the study may be transferred for the purpose of processing or analysis to approved associated researchers and commercial partners within/outside the European Economic Area. The Sponsor of the study will take all reasonable steps to protect your privacy.

In the future we may publish our findings from the study in scientific journals, but you will not be identifiable in any publications.

21. Will my GP be informed of my involvement?
Because this study is not being carried out by your GP, we would like to inform them of your participation. If you agree to take part and agree to us contacting your GP, we will give him or her details of the study and inform them that you have chosen to participate in it. You will not be able to participate in this study if you do not give us this permission to inform your GP.

22. What will happen to the results of the research study?
The results of the study will be available after it finishes and will usually be published online in a medical journal and presented at a scientific conference, they will also be posted to. The data will be anonymous and it will not be possible to identify you in any report or publication. Sometimes the data may be used to teach other healthcare professionals how to treat patients in a similar position to you.

Should you wish to see the results, or the publication, please ask your study doctor or see the trial website on https://www.ucl.ac.uk/surgery/research/research-department-targeted-intervention/prime-trial-information, or the clinical trials units website www.ncita.org.uk.

23. Who is organising and funding the research?
The governance sponsor is University College London. The study is funded by Prostate Cancer UK, the European Association of Urology Research Foundation, the UK National Institute for Health Research via an Academic Clinical Lectureship to Dr Veeru Kasivisvanathan and the UK National Cancer Imaging Translational Accelerator.

24. Who has reviewed the study?

For peer review only - http://bmjopen.bmj.com/site/about/guidelines.xhtml

All research in the NHS is looked at by an independent group of people, called a Research Ethics Committee, to protect your interests. This study has been reviewed and given favorable opinion by National Research Ethics Service Committee _West Midlands - Black Country Research Ethics Committee. Patients and members of the public have also reviewed the study documents to ensure they are appropriate and well written.

25. Further information

You are encouraged to ask any questions you wish, before, during or after your investigations. If you have any questions about the study, please speak to your study nurse or doctor on the numbers specified below, who will be able to provide you with up to date information about the procedures involved. If you wish to read the research on which this study is based, please ask your study nurse or doctor.

Site Study staff contact details:

Principal Investigator (site) details:

Alternatively, if you or your relatives have any questions about this study you may wish to contact one of the following organisations that are independent of the hospital at which you are being treated:

Prostate Cancer UK – 0800 082 1616 - http://prostatecanceruk.org

Macmillan Cancer Support - 0808 808 0000 – http://www.macmillan.org.uk

If you decide you would like to take part then please read and sign the consent form. You will be given a copy of this information sheet and the consent form to keep. A copy of the consent form will be filed in your patient notes, one will be filed with the study records and one may be sent to the Research Sponsor.

You can have more time to think this over if you are at all unsure.

Thank you for taking the time to read this information sheet and to consider this study.

For peer review only

For peer review only - http://bmjopen.bmj.com/site/about/guidelines.xhtml

Reporting checklist for protocol of a clinical trial.

Based on the SPIRIT guidelines.

Instructions to authors

Complete this checklist by entering the page numbers from your manuscript where readers will find each of the items listed below.

Your article may not currently address all the items on the checklist. Please modify your text to include the missing information. If you are certain that an item does not apply, please write "n/a" and provide a short explanation.

Upload your completed checklist as an extra file when you submit to a journal.

In your methods section, say that you used the SPIRITreporting guidelines, and cite them as:

Chan A-W, Tetzlaff JM, Gøtzsche PC, Altman DG, Mann H, Berlin J, Dickersin K, Hróbjartsson A, Schulz KF, Parulekar WR, Krleža-Jerić K, Laupacis A, Moher D. SPIRIT 2013 Explanation and Elaboration: Guidance for protocols of clinical trials. BMJ. 2013;346:e7586

		Reporting Item	Page Number
**Administrative information**			
Title	#1	Descriptive title identifying the study design, population, interventions, and, if applicable, trial acronym	#1
Trial registration	#2a	Trial identifier and registry name. If not yet	#3

For peer review only

		registered, name of intended registry	
Trial registration: data set	#2b	All items from the World Health Organization Trial Registration Data Set	Table 3
Protocol version	#3	Date and version identifier	Table 4
Funding	#4	Sources and types of financial, material, and other support	#13
Roles and responsibilities: contributorship	#5a	Names, affiliations, and roles of protocol contributors	#13
Roles and responsibilities: sponsor contact information	#5b	Name and contact information for the trial sponsor	Table 5
Roles and responsibilities: sponsor and funder	#5c	Role of study sponsor and funders, if any, in study design; collection, management, analysis, and interpretation of data; writing of the report; and the decision to submit the report for publication, including whether they will have ultimate authority over any of these activities	Table 5
Roles and responsibilities: committees	#5d	Composition, roles, and responsibilities of the coordinating centre, steering committee, endpoint adjudication committee, data management team, and other individuals or groups overseeing the trial,	Table 5

For peer review only

		if applicable (see Item 21a for data monitoring committee)	
**Introduction**			
Background and rationale	#6a	Description of research question and justification for undertaking the trial, including summary of relevant studies (published and unpublished) examining benefits and harms for each intervention	#4
Background and rationale: choice of comparators	#6b	Explanation for choice of comparators	#5
Objectives	#7	Specific objectives or hypotheses	#4
Trial design	#8	Description of trial design including type of trial (eg, parallel group, crossover, factorial, single group), allocation ratio, and framework (eg, superiority, equivalence, non-inferiority, exploratory)	#5
**Methods: Participants, interventions, and outcomes**			
Study setting	#9	Description of study settings (eg, community clinic, academic hospital) and list of countries where data will be collected. Reference to where list of study sites can be obtained	#5

For peer review only

Eligibility criteria	#10	Inclusion and exclusion criteria for participants. If applicable, eligibility criteria for study centres and individuals who will perform the interventions (eg, surgeons, psychotherapists)	#5
Interventions: description	#11a	Interventions for each group with sufficient detail to allow replication, including how and when they will be administered	#5
Interventions: modifications	#11b	Criteria for discontinuing or modifying allocated interventions for a given trial participant (eg, drug dose change in response to harms, participant request, or improving / worsening disease)	#11
Interventions: adherance	#11c	Strategies to improve adherence to intervention protocols, and any procedures for monitoring adherence (eg, drug tablet return; laboratory tests)	#9
Interventions: concomitant care	#11d	Relevant concomitant care and interventions that are permitted or prohibited during the trial	#9
Outcomes	#12	Primary, secondary, and other outcomes, including the specific measurement variable (eg, systolic blood pressure), analysis metric (eg, change from baseline, final value, time to event), method of aggregation (eg, median, proportion), and time point for each outcome. Explanation of the clinical relevance of chosen efficacy and harm outcomes is strongly recommended	#9

For peer review only

Participant timeline	#13	Time schedule of enrolment, interventions (including any run-ins and washouts), assessments, and visits for participants. A schematic diagram is highly recommended (see Figure)	Table 2 and Figure 1
Sample size	#14	Estimated number of participants needed to achieve study objectives and how it was determined, including clinical and statistical assumptions supporting any sample size calculations	#9
Recruitment	#15	Strategies for achieving adequate participant enrolment to reach target sample size	#9
**Methods: Assignment of interventions (for controlled trials)**			
Allocation: sequence generation	#16a	Method of generating the allocation sequence (eg, computer-generated random numbers), and list of any factors for stratification. To reduce predictability of a random sequence, details of any planned restriction (eg, blocking) should be provided in a separate document that is unavailable to those who enrol participants or assign interventions	N/A
Allocation concealment mechanism	#16b	Mechanism of implementing the allocation sequence (eg, central telephone; sequentially numbered, opaque, sealed envelopes), describing	N/A

For peer review only

		any steps to conceal the sequence until interventions are assigned	
Allocation: implementation	#16c	Who will generate the allocation sequence, who will enrol participants, and who will assign participants to interventions	N/A
Blinding (masking)	#17a	Who will be blinded after assignment to interventions (eg, trial participants, care providers, outcome assessors, data analysts), and how	N/A
Blinding (masking): emergency unblinding	#17b	If blinded, circumstances under which unblinding is permissible, and procedure for revealing a participant's allocated intervention during the trial	N/A
**Methods: Data collection, management, and analysis**			
Data collection plan	#18a	Plans for assessment and collection of outcome, baseline, and other trial data, including any related processes to promote data quality (eg, duplicate measurements, training of assessors) and a description of study instruments (eg, questionnaires, laboratory tests) along with their reliability and validity, if known. Reference to where data collection forms can be found, if not in the protocol	#9 and Supplementary Appendix 1

For peer review only

Data collection plan: retention	#18b	Plans to promote participant retention and complete follow-up, including list of any outcome data to be collected for participants who discontinue or deviate from intervention protocols	#9
Data management	#19	Plans for data entry, coding, security, and storage, including any related processes to promote data quality (eg, double data entry; range checks for data values). Reference to where details of data management procedures can be found, if not in the protocol	#9
Statistics: outcomes	#20a	Statistical methods for analysing primary and secondary outcomes. Reference to where other details of the statistical analysis plan can be found, if not in the protocol	#10
Statistics: additional analyses	#20b	Methods for any additional analyses (eg, subgroup and adjusted analyses)	#10
Statistics: analysis population and missing data	#20c	Definition of analysis population relating to protocol non-adherence (eg, as randomised analysis), and any statistical methods to handle missing data (eg, multiple imputation)	#10

Methods: Monitoring

Data monitoring: formal committee	#21a	Composition of data monitoring committee (DMC); summary of its role and reporting structure;	#11

For peer review only

		statement of whether it is independent from the sponsor and competing interests; and reference to where further details about its charter can be found, if not in the protocol. Alternatively, an explanation of why a DMC is not needed	
Data monitoring: interim analysis	#21b	Description of any interim analyses and stopping guidelines, including who will have access to these interim results and make the final decision to terminate the trial	#11
Harms	#22	Plans for collecting, assessing, reporting, and managing solicited and spontaneously reported adverse events and other unintended effects of trial interventions or trial conduct	#11
Auditing	#23	Frequency and procedures for auditing trial conduct, if any, and whether the process will be independent from investigators and the sponsor	Table 5
**Ethics and dissemination**			
Research ethics approval	#24	Plans for seeking research ethics committee / institutional review board (REC / IRB) approval	#11
Protocol amendments	#25	Plans for communicating important protocol modifications (eg, changes to eligibility criteria, outcomes, analyses) to relevant parties (eg, investigators, REC / IRBs, trial participants, trial	Table 4

For peer review only

		registries, journals, regulators)	
Consent or assent	#26a	Who will obtain informed consent or assent from potential trial participants or authorised surrogates, and how (see Item 32)	#12
Consent or assent: ancillary studies	#26b	Additional consent provisions for collection and use of participant data and biological specimens in ancillary studies, if applicable	Supplementary Appendix 3
Confidentiality	#27	How personal information about potential and enrolled participants will be collected, shared, and maintained in order to protect confidentiality before, during, and after the trial	#12
Declaration of interests	#28	Financial and other competing interests for principal investigators for the overall trial and each study site	#12
Data access	#29	Statement of who will have access to the final trial dataset, and disclosure of contractual agreements that limit such access for investigators	#12
Ancillary and post trial care	#30	Provisions, if any, for ancillary and post-trial care, and for compensation to those who suffer harm from trial participation	#11
Dissemination policy: trial results	#31a	Plans for investigators and sponsor to communicate trial results to participants, healthcare professionals, the public, and other relevant groups (eg, via publication, reporting in results databases, or other	#12

For peer review only

		data sharing arrangements), including any publication restrictions	
Dissemination policy: authorship	#31b	Authorship eligibility guidelines and any intended use of professional writers	#12
Dissemination policy: reproducible research	#31c	Plans, if any, for granting public access to the full protocol, participant-level dataset, and statistical code	#12

Appendices

Informed consent materials	#32	Model consent form and other related documentation given to participants and authorised surrogates	Supplementary Appendix 3
Biological specimens	#33	Plans for collection, laboratory evaluation, and storage of biological specimens for genetic or molecular analysis in the current trial and for future use in ancillary studies, if applicable	Supplementary Appendix 1 and 2

Notes:

- 18a: #11 and Supplementary Appendix 1
- 26b: Supplementary Appendix 3
- 32: Supplementary Appendix 3
- 33: Supplementary Appendix 1 and 2
- The SPIRIT Explanation and Elaboration paper is distributed under the terms of the Creative Commons Attribution License CC-BY-NC. This checklist was completed on 16. November 2022

For peer review only

using https://www.goodreports.org/, a tool made by the EQUATOR Network in collaboration with Penelope.ai

For peer review only

BMJ Open

Comparing biparametric to multiparametric MRI in the diagnosis of clinically significant prostate cancer in biopsy-naive men (PRIME): a prospective, international, multicentre, non-inferiority, within-patient, diagnostic yield trial protocol

Journal:	*BMJ Open*
Manuscript ID	bmjopen-2022-070280.R1
Article Type:	Protocol
Date Submitted by the Author:	08-Feb-2023
Complete List of Authors:	Asif, Aqua; University College London, Division of Surgery and Interventional Science<br>Nathan, Arjun; University College London, Division of Surgery and Interventional Science; Royal College of Surgeons of England, Clinical Effectiveness Unit<br>Ng, Alexander; University College London, Division of Surgery and Interventional Science<br>Khetrapal, Pramit; University College London, Division of Surgery and Interventional Science; Whipps Cross University Hospital, Department of Urology<br>Chan, Vinson Wai-Shun; University College London, Division of Surgery and Interventional Science<br>Giganti, Francesco; University College London, Division of Surgery and Interventional Science; University College London Hospitals NHS Foundation Trust, Department of Radiology<br>Allen, Clare; University College London, Division of Surgery and Interventional Science; University College London Hospitals NHS Foundation Trust, Department of Radiology<br>Freeman, Alex; University College London Hospitals NHS Foundation Trust, Department of Histopathology<br>Punwani, Shonit; University College London Hospitals NHS Foundation Trust, Department of Radiology; University College London, Centre for Medical Imaging<br>Lorgelly, Paula; University College London, Institute of Epidemiology and Health Care; The University of Auckland, School of Population Health<br>Clarke, Caroline; University College London, Research Department of Primary Care and Population Health<br>Brew-Graves, Chris; University College London, National Cancer Imaging Translational Accelerator<br>Muirhead, Nicola; University College London, National Cancer Imaging Translational Accelerator<br>Emberton, Mark; University College London, Division of Surgery and Interventional Science; University College London Hospitals NHS Foundation Trust, Department of Urology<br>Agarwal, Ridhi; University of Birmingham, Test Evaluation Research Group, Institute of Applied Health Research; University Hospitals Birmingham NHS Foundation Trust, NIHR Birmingham Biomedical

	Research Centre<br>Takwoingi, Yemisi; University of Birmingham, Test Evaluation Research Group, Institute of Applied Health Research; University Hospitals Birmingham NHS Foundation Trust, NIHR Birmingham Biomedical Research Centre<br>Deeks, Jonathan; University of Birmingham, Test Evaluation Research Group, Institute of Applied Health Research; University Hospitals Birmingham NHS Foundation Trust, NIHR Birmingham Biomedical Research Centre<br>Moore, Caroline; University College London, Division of Surgery and Interventional Science; University College London Hospitals NHS Foundation Trust, Department of Urology<br>Kasivisvanathan, Veeru; University College London, Division of Surgery and Interventional Science; University College London Hospitals NHS Foundation Trust, Department of Urology<br>Trial Group, PRIME; University College London
**Primary Subject Heading**:	Urology
Secondary Subject Heading:	Radiology and imaging, Surgery
Keywords:	Prostate disease < UROLOGY, Magnetic resonance imaging < RADIOLOGY & IMAGING, RADIOLOGY & IMAGING, Protocols & guidelines < HEALTH SERVICES ADMINISTRATION & MANAGEMENT, Urological tumours < ONCOLOGY

SCHOLARONE™
Manuscripts

Comparing biparametric to multiparametric MRI in the diagnosis of clinically significant prostate cancer in biopsy-naive men (PRIME): a prospective, international, multicentre, non-inferiority within-patient, diagnostic yield trial protocol

Authors:
Aqua Asif[1†]
Arjun Nathan[1,2†]
Alexander Ng[1]
Pramit Khetrapal[1,3]
Vinson Wai-Shun Chan[1]
Francesco Giganti[1,4]
Clare Allen[1,4]
Alex Freeman[5]
Shonit Punwani[4,6]
Paula Lorgelly[7,8]
Caroline S Clarke[9]
Chris Brew-Graves[10]
Nicola Muirhead[10]
Mark Emberton[1,11]
Ridhi Agarwal[12,13]
Yemisi Takwoingi[12,13]
Jon Deeks[12,13]
Caroline M Moore[1,11]
Veeru Kasivisvanathan[1,11]
On behalf of the PRIME Trial Group*

†Joint-first authors have made equal contribution to this study

*PubMed Indexed Collaborators (PRIME Trial Group)
John Wilkinson, Marimo Rossiter, Réka Novotta, Philip Ryan, Alex Kirkham, Louise Dickinson, Tushar Narain, Hazel McBain, Antonette Andrews, Daniel Margolis, Valeria Panebianco, Antti Ranniko, Peter Albertsen, Lance Mynderse, Adam Froemming, Naoki Takahasi, Tristan Barrett, Raphaele Renard Penna, Vibeke Løgager, Lars Boesen, Sangeet Ghai, Lars Budäus, Valeria Panebianco, Enrique Gómez Gómez, Jeremy Grummet, Tho Pham, Aiman Haider, Jing Yi Jessica Weng, Anders Bjartell, Wim Witjes, Christien Caris, Joke Van Egmond.

Affiliations:
1. Division of Surgery and Interventional Science, University College London, London, United Kingdom
2. Clinical Effectiveness Unit, The Royal College of Surgeons of England, United Kingdom
3. Department of Urology, Whipps Cross Hospital, Barts Health NHS Trust, London, United Kingdom
4. Department of Radiology, University College London Hospital NHS Foundation Trust, London, United Kingdom
5. Department of Pathology, University College London Hospitals NHS Foundations Trust, London, United Kingdom
6. Centre for Medical Imaging, University College London, London, United Kingdom
7. Institute of Epidemiology and Health Care, University College London, London, United Kingdom
8. School of Population Health, The University of Auckland, Auckland, 1023, New Zealand
9. Research Department of Primary Care and Population Health, University College London, London, United Kingdom

For peer review only - http://bmjopen.bmj.com/site/about/guidelines.xhtml

10. National Cancer Imaging Translational Accelerator, Division of Medicine, University College London, London, United Kingdom
11. Department of Urology, University College London Hospital NHS Foundation Trust, London, United Kingdom
12. Test Evaluation Research Group, Institute of Applied Health Research, University of Birmingham, Birmingham, United Kingdom
13. NIHR Birmingham Biomedical Research Centre, University Hospitals Birmingham NHS Foundation Trust, University of Birmingham, Birmingham, United Kingdom

Corresponding Author:
Veeru Kasivisvanathan (veeru.kasi@ucl.ac.uk)
@veerukasi
Division of Surgery and Interventional Science, University College London, London, United Kingdom

Keywords:
prostate cancer, diagnostic, MRI, prostate, biopsy

Current word count: 3699
Figure limit: No limits on figures or tables
Figure count: 1
Box count: 2
Table count: 5
Supplementary appendix count: 3

ARTICLE SUMMARY

STRENGTHS AND LIMITATIONS

- *Strength:* PRIME is a pragmatic, prospective, international, multicentre trial being carried out in a range of different healthcare settings
- *Strength:* Its within-patient design allows patients to act as their own control, improving the efficiency and power of the trial compared to a randomised study
- *Strength:* Its within-patient design allows the impact of the dynamic contrast enhanced sequences on staging decisions and treatment eligibility to be made at an individual patient level.
- *Strength:* PRIME will be one of the first trials to quality control the performance of sites' dynamic contrast enhanced sequences prior to their involvement in the trial
- *Limitation:* as both biparametric and multiparametric targeted biopsies are carried out in the same patient it is possible for the performance of one technique to influence the other

For peer review only - http://bmjopen.bmj.com/site/about/guidelines.xhtml

ABSTRACT

Introduction

Prostate MRI is a well-established tool for the diagnostic work-up for men with suspected prostate cancer. Current recommendations advocate the use of multiparametric MRI (mpMRI), which is comprised of three sequences: T2-weighted (T2W), diffusion-weighted (DWI), and dynamic contrast enhanced (DCE). Prior studies suggest that a biparametric MRI approach (bpMRI), omitting the DCE sequences, may not compromise clinically significant cancer detection, though there are limitations to these studies, and it is not known how this may affect treatment eligibility. A bpMRI approach will reduce scanning time, may be more cost effective and at a population level, allow more men to gain access to an MRI than a mpMRI approach.

Methods

PRIME is a prospective, international, multicentre within-patient diagnostic yield trial, assessing whether bpMRI is non-inferior to mpMRI in the diagnosis of clinically significant prostate cancer. Patients will undergo the full mpMRI scan. Radiologists will be blinded to the dynamic contrast enhanced sequence (DCE) and will initially report the MRI using only the bpMRI (T2W and DWI) sequences. They will then be unblinded to the DCE sequence and will then re-report the MRI using the mpMRI sequences (T2W, DWI and DCE). Men with suspicious lesion(s) on either bpMRI or mpMRI will undergo prostate biopsy. The main inclusion criteria are men with suspected prostate cancer, with a serum PSA of ≤ 20 ng/mL and no prior prostate biopsy. The primary outcome is the proportion of men with clinically significant prostate cancer detected (Gleason ≥ 3+4 or Gleason Grade Group ≥ 2). A sample size of at least 500 patients is required. Key secondary outcomes include the proportion of clinically insignificant prostate cancer detected and treatment decision.

Ethics and Dissemination Ethical approval was obtained from the National Research Ethics Committee West Midlands, Nottingham 21/WM/0091. Results of this trial will be disseminated through peer-reviewed publications. Participants and relevant patient support groups will be informed about the results of the trial.

Registration details NCT04571840

STUDY TITLE

Long Title: A trial assessing whether biparametric MRI is non-inferior to multiparametric MRI in the diagnosis of clinically significant prostate cancer

Short Title: Prostate Imaging Using MRI +/- Contrast Enhancement

Trial Acronym: PRIME

For peer review only

For peer review only - http://bmjopen.bmj.com/site/about/guidelines.xhtml

INTRODUCTION

This protocol was written according to SPIRIT guidelines [1]. Magnetic Resonance Imaging (MRI) is widely established as the gold standard diagnostic imaging modality for detecting clinically significant prostate cancer (PCa) [2]. The landmark PRECISION study established the benefit of detecting clinically significant prostate cancer using MRI and targeting biopsies based on MRI findings [3]. The National Prostate Cancer Audit data from England showed that only 62% of patients receive prostate MRI before biopsy, despite the level 1 evidence to support the use of MRI [2] [3] [4].

Current recommendations for the use of MRI for detection of PCa focus on the use of multiparametric MRI (mpMRI) [2] [3]. mpMRI consists of three sequences: T2-weighted (T2W), diffusion-weighted (DWI) and dynamic contrast enhanced (DCE) sequences. On the DCE sequences, cancer-suspicious areas can demonstrate early wash-in, enhancement and rapid wash-out of contrast [5] [6] [7] [8]. The DCE sequences involve administering gadolinium contrast via an intravenous cannula. Therefore, it increases scanning time and healthcare costs compared to a bpMRI approach where only T2W and DWI are used. Whilst gadolinium is in widespread use, literature suggests it may accumulate in the basal ganglia, though its clinical relevance is not fully understood [9] [10]. In patients who are likely to get repeated scans over their lifetime – there may be no advantage of using the additional contrast if the bpMRI option is as good as the mpMRI option.

Removing the DCE sequences from the MRI protocol has been suggested as a potential avenue to improve the cost-effectiveness of using MRI in the diagnostic pathway for PCa [11] [12] and the reduced scanning time required may improve the number of men with suspected prostate cancer accessing an MRI scan. Using bpMRI has demonstrated similar detection rates of PCa as mpMRI but current evidence is limited primarily to retrospective, single-centre studies [12] [13]. The few prospective studies have not been typically robustly designed to evaluate the role of DCE in prostate cancer detection [13] [14].

The PRIME trial aims to assess whether bp-MRI is non-inferior to mpMRI in the detection of clinically significant prostate cancer. PRIME may redefine the standard of care diagnostic test for men with suspicion of PCa and allow many more patients who need access to an MRI to get one.

Objectives

The primary objective is to compare the detection of clinically significant PCa (Gleason ≥ 3+4 or Gleason Grade Group ≥ 2) using bpMRI ± targeted biopsy with mpMRI ± targeted biopsy.

Key secondary objectives include:

- To compare the proportion of men who have clinically insignificant PCa (Gleason 3+3 or Gleason Grade Group 1) detected for bpMRI versus mpMRI
- To compare the proportion of men with non-suspicious MRIs for bpMRI versus mpMRI
- To compare the proportion of men with indeterminately-scored MRI as reported by bpMRI and mpMRI
- To compare the proportion of men with MRIs of adequate standard for reporting for bpMRI versus mpMRI
- To compare the diagnostic test performance for bpMRI versus mpMRI
- To compare radiological staging for bpMRI versus mpMRI
- To compare treatment eligibility decisions for bpMRI when compared with mpMRI
- To compare diagnostic performance of bpMRI and mpMRI when using the Likert scoring system in comparison to the PI-RADS v2.1 scoring system
- To compare the cost effectiveness of bpMRI when compared to mpMRI

For peer review only - http://bmjopen.bmj.com/site/about/guidelines.xhtml

Trial Design

The PRIME trial is designed as a prospective, multicentre, within-patient, diagnostic yield trial, assessing whether bpMRI is non-inferior to mpMRI for the diagnosis of clinically significant PCa in biopsy-naive men. A paired cohort design was chosen rather than a randomised trial design for the following reasons:

- More efficient design (sevenfold lower sample size required) with equivalent quality of evidence in the setting of a diagnostic study
- Patients act as their own control due to the within-patient design, thus allowing us to draw conclusions regarding the value of DCE sequences on a per patient level
- Allows for the evaluation of the impact of contrast on staging decisions and treatment eligibility decisions at an individual patient level
- Patients get the benefit of having targeted biopsies based on the information from both bpMRI and mpMRI information, whereas with a randomised study, patients randomised to one technique will be denied of potential benefit of the other

METHODS AND ANALYSIS

Trial Setting

We expect centres who perform prostate cancer diagnostics and management from the following countries to take part: Argentina, Australia, Belgium, Brazil, Canada, Denmark, France, Finland, Germany, Italy, Netherlands, Singapore, Spain, UK and USA. Sites will be required to undergo a period of quality control prior to including patients to ensure minimum acceptable standards for the conduct of mpMRI, reporting and targeted biopsy.

Eligibility Criteria

Patients will be considered eligible for registration into this trial if they fulfil all of the inclusion criteria and none of the exclusion criteria (**Box 1**).

Box 1 Eligibility criteria

Inclusion criteria

1. Men at least 18 years of age referred with clinical suspicion of prostate cancer
2. Serum PSA ≤ 20 ng/mL
3. Fit to undergo all procedures listed in protocol
4. Able to provide written informed consent

Exclusion criteria

1. Prior prostate biopsy
2. Prior treatment for prostate cancer
3. Prior prostate MRI on a previous encounter
4. Contraindication to MRI (*e.g.* claustrophobia, some pacemakers)
5. Contraindication to prostate biopsy
6. Unfit to undergo any procedures listed in protocol

Interventions

MRI Conduct

MRI will be conducted with 1.5T or 3.0T with pelvic-phased array coils, with or without endorectal coils. The PRECISION study quality control highlighted that the image quality of the DCE sequences was the most variable sequence across sites [3]. Therefore, to give DCE a reasonable chance of demonstrating whether it has value, MRI scanner approval for use in the study will be made on the basis of central review of MRI images, utilising the Prostate Imaging Quality (PI-QUAL) scoring system [15]. In brief, PI-QUAL is a 5-point Likert scoring system, where 1 indicates no sequences are of diagnostic quality and 5 implies that each sequence individually is of optimal diagnostic quality. The objective criteria used to determine PI-QUAL scores are derived from internationally published

For peer review only - http://bmjopen.bmj.com/site/about/guidelines.xhtml

minimum standards for MRI conduct [16]. If necessary, sites will be given recommendations to improve image quality and will be re-evaluated after optimisation for participation in the study.

Reporting of MRI

Patients will undergo (or will have undergone) standard of care mpMRI as per their local protocol. The radiologists participating in this trial will be blinded to the DCE sequences and will report the MRI using only the biparametric (T2W and DWI) sequences in Report 1. After reporting the bpMRI, the same radiologist will be unblinded to the DCE sequences and will re-report the MRI using the mpMRI sequences (T2W, DWI and DCE) in Report 2 (**Figure 1**).

The MRIs and lesions are scored on a 1–5 scale of suspicion for the likelihood that clinically significant PCa is present, with 5 representing the greatest score of suspicion. Both the traditional Likert and PI-RADS v2.1 scoring systems will be used to identify any suspicious lesions in the prostate. Suspicious areas (Likert or PI-RADS v2.1 ≥3) on either bpMRI or mpMRI will undergo targeted biopsy of the prostate, with cores from contrast-enhanced suspicious areas stored separately.

A summary of the rules for reporting MRI scans in the PRIME trial is in **Box 2.** Please see **Supplementary Appendix 1** for our model reporting proformas, which radiologists participating in the PRIME trial will use to label lesions.

Non-suspicious bpMRI and mpMRI

Men whose MRIs do not show suspicious areas on bpMRI and mpMRI (i.e. scored 1 or 2 on Likert and PI-RADS v2.1) will be stratified by PSA density. Men with PSA density <0.15ng/mL/mL will not undergo biopsy and men with PSA density ≥0.15ng/mL/mL will undergo systematic biopsy.

Box 2 Summary of MRI reporting rules

Report 1 (biparametric MRI: T2W and DWI)

1. The radiologist reporting this will be blinded to DCE, with verification of this via an independent person or an automated system (MIM by MIM Software Inc)
2. The radiologist should then interpret the bpMRI sequences **blinded** to DCE
3. Up to 4 suspicious areas (score ≥ 3 out of 5 on the Likert or PI-RADS v2.1 scoring system) can be marked on Report 1 – if there are more, the four **most suspicious** should only be marked on
4. The location of the suspicious areas should be labelled according to the PI-RADS v2.1 41-sector diagram
5. Once Report 1 (biparametric MRI: T2W and DWI) has been done, this **cannot be altered** after looking at the DCE

Report 2 (multiparametric MRI: T2W, DWI and DCE)

1. The same radiologist must report both Report 1 and Report 2
2. They will then be **unblinded** to the DCE sequence
3. The radiologist should now complete Report 2
4. The location of the suspicious areas should be similarly labelled according to the PI-RADS v2.1 41-sector diagram as above
5. On Report 2, each of the existing lesions are additionally labelled as one of:

bpMRI positive, mpMRI positive

This occurs when a lesion scores 3, 4 or 5 on both bpMRI and mpMRI based on ***either*** Likert or PI-RADS v2.1 scoring systems

bpMRI positive, mpMRI negative

For peer review only - http://bmjopen.bmj.com/site/about/guidelines.xhtml

This occurs when a lesion scores 3, 4 or 5 on bpMRI on ***either*** Likert or PI-RADS v2.1 scoring systems, but also scores a 1 or 2 on mpMRI on both Likert and PI-RADS v2.1 scoring systems

bpMRI negative, mpMRI positive

There are two instances in which **new targets** may be labelled and drawn onto Report 2:

1. **New** lesions scoring 3, 4 or 5, identified by DCE not previously identified on bpMRI should be marked on as new lesions as **DCE Targets** and **bpMRI negative, mpMRI positive**

2. Lesions that appear **larger** on DCE should be treated as 2 separate targets
   - One target depicts the completely overlapping segment from Report 1 (bpMRI positive, mpMRI positive)
   - The non-overlapping part which would otherwise not be sampled should be labelled as a **new target** (bpMRI **negative**, mpMRI positive). This is a subjective decision by the radiologist. A typical example of when to declare this as a separate target is if the non-overlapping part enters an adjacent sector on the PI-RADS v2.1 sector diagram

A biopsy plan is recommended by the radiologist thereafter for the biopsy operator to follow.

Prostate Biopsy Procedures

MRI-Targeted Biopsy

Men will undergo MRI-targeted biopsy if either their bpMRI or mpMRI identifies a suspicious lesion which scores ≥3 on either Likert or PI-RADS v2.1. Four targeted cores will be taken per suspicious lesion, and these should be stored and labelled in separate containers to ensure cancer detection from separate suspicious areas are ascertained.

Systematic Biopsy

Systematic biopsies should be performed after targeted biopsies, with 6 cores taken from the contralateral side of the MRI lesion, focused on sampling the peripheral zone of the prostate. If there are bilateral MRI lesions or midline lesions, then no systematic biopsies are necessary.

Please see **Supplementary Appendix 2** for a detailed overview of how our biopsies will be conducted.

Prostate Histopathology

Both the Gleason score and the Gleason Grade Group will be reported for the overall biopsy and for each individual target lesion.

Pre-Trial Assessments

For all patients, patient referral would follow clinical suspicion of PCa (*e.g.* raised PSA or abnormal digital rectal examination). To confirm a patient's eligibility, screening will be undertaken. Patients can enter the trial either before or after they have had their mpMRI scan. If patients are recruited after an mpMRI scan has been carried out, this will only be permitted if the MRI has not been seen by any clinician.

Registration Procedures

Following consent and confirmation of eligibility, trial processes can commence. The patient will be registered and assigned a trial ID using a central online database (Marvin by XClinical).

Intervention Procedures

For peer review only - http://bmjopen.bmj.com/site/about/guidelines.xhtml

For peer review only

All patients will undergo a full mpMRI scan. This includes T2W, DWI and DCE sequences.

Follow-Up for Results

If bpMRI and mpMRI is non-suspicious and PSA density is <0.15 ng/mL/mL, the patient will be counselled for standard of care follow-up – typically consisting of PSA surveillance. If a decision for prostate biopsy or other tests is made, these results will be recorded after which the participant completes the trial.

Multidisciplinary Team Decision-Making for Treatment Eligibility

Treatment decisions will be per local standard of care, based on pathology results and will be recorded. Subsequently, a virtual multidisciplinary team meeting will be conducted and treatment eligibility decisions blinded to the DCE will be recorded. Once a decision has been recorded, the clinicians will be unblinded to the DCE sequence and the impact that this information makes on treatment eligibility will be evaluated.

MRI and Pathology Quality Control

Quality control will be carried out at the end of the study by the PRIME chief radiologists reviewing the original MRIs, who will assess the MRI quality and re-report the MRI blinded to the study reports. Anonymised pathology slides from a proportion of patients may also be reviewed by central pathologists. Any slides assessed outside of the originating site will be returned to the original site after quality control. Quality control results will be reported but will not influence patient management or outcomes.

Cost-Effectiveness

A within-trial incremental cost-effectiveness analysis will be conducted to calculate the difference in mean cost per diagnosis of clinically significant prostate cancer if a strategy of bpMRI were adopted instead of the current mpMRI standard of care, over a time horizon of 30 days. The difference in cost of avoiding each additional case of clinically insignificant prostate cancer diagnosed may also be calculated.

Costs of procedures will be estimated by applying standard unit costs to resource use data captured within the trial plus other procedures that would be offered to patients in either pathway. Estimates of the resources used (procedures, tests, radiotherapy, chemotherapy, other therapies, surveillance visits, and other care events) on the two treatment pathways will be obtained for the theoretical bpMRI cohort using decisions made initially by the MDT with information from the bpMRI scan and any biopsies as a result of that scan; and estimates of the treatment pathway resources used in the theoretical mpMRI cohort will be made subsequently by the MDT on viewing additional information from the mpMRI scan and any further biopsies performed as a result of that scan. This thought experiment is required due to the ethical requirement to use all available information, *i.e.* not just bpMRI and biopsies or just mpMRI and biopsies, when making the actual treatment decision with the patient.

The analysis perspective will be that of the NHS and personal social services. Standard unit costs (*e.g.* NHS Reference Costs) will be supplemented by unit cost data from the participating trial sites. A microcosting study to provide this information will be undertaken in a small number of sites as part of the trial to investigate the resources employed to deliver bpMRI and mpMRI scans. This information will allow us to understand the MRI booking system, consumption of consumables, and staff time as related to delivering bpMRI and mpMRI scans.

Depending on the within-trial cost-effectiveness findings, consideration will be given to extending this analysis using decision analytic modelling to estimate quality-adjusted life-years gained (QALYs) over

For peer review only - http://bmjopen.bmj.com/site/about/guidelines.xhtml

a lifetime horizon. Quality of life information will be estimated from anonymised patient-level data by the same group from an earlier study in this instance.

Outcomes

Primary Outcome

The primary outcome will be the proportion of men with clinically significant PCa detected – any pattern 4 disease on any core (*i.e.* Gleason ≥3+4 or Gleason Grade Group ≥2). The time frame for assessment: when biopsy results are available, at an expected average of 30 days post-biopsy.

Secondary Outcomes

Table 1 lists our secondary outcomes.

Sample Size

The margin of clinical unimportance to allow a conclusion of non-inferiority of bpMRI to mpMRI to be made was set at 5 percentage points – i.e. if the lower bound of the 95% confidence intervals (CIs) for the difference in detection rates of bpMRI-targeted biopsy compared to mpMRI-targeted biopsy is above -5 percentage points, then bpMRI will be deemed as non-inferior.

Using simulation, an mpMRI underlying probability of detecting clinically significant cancer of 38% (3) and the following, two key probabilities were used to determine the sample size:

A. The probability that a patient found to have no suspicious lesions on bpMRI or have no clinically significant PCa on bpMRI-targeted biopsy will have clinically significant PCa on mpMRI-targeted biopsy

B. The probability that a patient found to have no suspicious lesions on mpMRI or have no clinically significant PCa on mpMRI-targeted biopsy will have clinically significant PCa on bpMRI-targeted biopsy

Assuming the probability of A is greater than the probability of B, and applying McNemar's test in each of 1,000 simulation runs for each combination of probabilities A and B ranging from 0 to 0.05, a sample size of 400 patients gives more than 90% power across these probabilities of A and B. Accounting for 20% dropout or exclusion after enrolment, at least 500 patients will be required.

Recruitment

At each participating site, enrolment will occur at outpatient clinics. With at least 25 sites, it is estimated that the trial will complete within 24 months of commencement. The trial opened for recruitment in April 2022 and the estimated completion date is April 2024.

Data Collection Methods

The electronic case report form (eCRF) system Marvin by XClinical will be used to collect data.

Patient-Reported Outcome Measures

The International Index of Erectile Function (IIEF-5) and the International Prostate Symptom Score (IPSS) will be utilised to assess baseline erectile function and lower urinary tract symptoms, respectively. These questionnaires will aid the multidisciplinary team decision-making for treatment eligibility.

Patient Retention

It is estimated that loss to follow-up will be no more than 20% due to the expected short time interval between enrolment and end of study. It is expected that the majority of patients will complete the trial within 4 to 6 weeks (**Table 2A, Table 2B**).

For peer review only

For peer review only - http://bmjopen.bmj.com/site/about/guidelines.xhtml

Statistical Methods

A statistical analysis plan will be finalised before our database lock and before any statistical analysis occurs. A consort diagram will be presented. All continuous variables will be described using the mean and standard deviation, or median and interquartile range, as appropriate. Categorical variables will be described using frequencies and percentages. Baseline characteristics will be examined and presented for those with and those without clinically significant PCa. The assumptions underpinning the statistical methods used will be assessed. The use of transformations will be considered to satisfy statistical assumptions.

Primary Outcome Analysis

The primary outcome is the difference in the proportion of men with clinically significant PCa, as detected by bpMRI-targeted biopsy compared to mpMRI-targeted biopsy. The proportion of men with clinically significant PCa, Gleason ≥ 3+4 or Gleason Grade Group ≥ 2, detected by bpMRI-targeted biopsy is defined as the number of men with clinically significant PCa identified on bpMRI-targeted biopsy divided by the number of men undergoing bpMRI. Similarly, the proportion of men with clinically significant PCa detected by mpMRI-targeted biopsy is defined as the number of men with clinically significant PCa identified on mpMRI-targeted biopsy divided by the number of men undergoing mpMRI. Methods that account for the paired nature of the data such as McNemar's test will be used to compare bpMRI and mpMRI.

Secondary Outcome Analysis

The proportion of men with clinically insignificant cancer (any cancer core with Gleason 3+3 or Gleason grade group 1 detected by bpMRI-targeted biopsy will be compared to that of mpMRI-targeted biopsy. The proportion of men with clinically insignificant cancer detected by bpMRI-targeted biopsy is defined as the number of men with clinically insignificant prostate cancer identified on bpMRI-targeted biopsy divided by the number of men undergoing bpMRI. Similarly, the proportion of men with clinically insignificant cancer detected by mpMRI-targeted biopsy is defined as the number of men with clinically insignificant prostate cancer identified on mpMRI-targeted biopsy divided by the number of men undergoing mpMRI. The same analytical approach described for clinically significant PCa will be applied.

The number and proportion of men scoring 1 or 2 (non-suspicious) or 3 (indeterminate) on bpMRI and mpMRI will be reported. A two-way table will be produced to show the agreement between the two MRI results using the Likert scoring system on a scale of 1-5.

The number and proportion of men with adequate standard of reporting on bpMRI and mpMRI will be reported.

A two-way table will be produced to show the number and proportion of patients with each radiological stage of bpMRI and mpMRI. Similarly, we will report the number and proportion of patients eligible for different treatment options following discussion of the bpMRI and mpMRI results in the Multidisciplinary Team meeting.

Using histopathology as the reference standard, sensitivity, specificity, positive predictive value and negative predictive value with 95% CI of bpMRI and mpMRI will be reported. The following assumptions will be made, where non-suspicious MRI refers to a score of 1 or 2 and suspicious MRI refers to a score of 3, 4 or 5 on the Likert and PI-RADS v2.1 scoring systems and absence of clinically significant cancer refers to a combination of clinical insignificant and no cancer.

The number and proportion of men with clinically significant cancer detected by systematic biopsy and not detected by bpMRI and mpMRI with targeted biopsy will be reported. A two-way table will be

For peer review only - http://bmjopen.bmj.com/site/about/guidelines.xhtml

produced to show a comparison between systematic biopsy (no biopsy, clinically significant cancer, clinically insignificant cancer and no cancer) and the two MRI results with targeted biopsy (no biopsy, clinically significant cancer, clinically insignificant cancer and no cancer).

Sensitivity and Other Planned Analyses

The primary outcome analysis will be repeated with a definition of clinically significant PCa being any primary pattern 4 disease – Gleason 4+3 or Gleason Grade Group 3.

Monitoring

The NCITA Global Prostate Trial Steering Committee (TSC) is responsible for the governance of the PRIME Study. A sub-group of independent TSC members form the Data Monitoring Sub-Committee (DMSC).

Roles and responsibilities of the TSC

To act as the oversight body for up to five prostate cancer studies on behalf of the Sponsor and Funders. In addition, the independent members will form a DMSC to review safety. The role of the TSC is to provide oversight for the studies and provide advice through its Chair to the Chief Investigators (CI), whilst working in tandem with the DMSC, Sponsor, Funders and host institution on all aspects of the studies. The rights, safety and well-being of the study participants are the most important consideration and should prevail over the interests of science and society.

Harms

Adverse events (AEs) will be defined as 'any untoward medical occurrence in a clinical trial subject undergoing any intervention in the trial, which does not necessarily have a causal relationship with this treatment'.

Serious adverse events (SAEs) will be defined as 'any untoward medical occurrence as a result of any intervention in the trial that:

- Results in death,
- Is life-threatening
- Requires hospitalisation or prolongation of existing inpatients' hospitalisation, results in persistent or significant disability or incapacity'

AEs and SAEs will be recorded until 30 days post biopsy. In the event that the patient does not undergo biopsy, AEs and SAEs should be recorded until 30 days post-MRI.

Unexpected AEs will be recorded by a member of the research team or clinical team on an AE report form eCRF. All SAEs must be recorded on an SAE report form eCRF which must be sent to the coordinating trials unit within 24 hours of knowledge of the SAE. Both AEs and SAEs should be recorded in the medical notes.

Ethics and Approval

The UK National REC (West Midlands – Black Country Research Ethics Committee, Nottingham) gave favourable approval for PRIME protocol version 2.0 on 28 June 2021 (Ref:21/WM/0091). All participating centres have gained local and ethical approvals prior to receiving a site initiation visit and approval by the sponsor to open for recruitment.

Patient and Public Involvement

Patients and public members were involved in defining the research question, evaluation of the research proposal, suggesting modifications to the trial, reviewing the patient information sheet,

For peer review only - http://bmjopen.bmj.com/site/about/guidelines.xhtml

consent form and GP letter. Patient groups and charities will also be involved in the dissemination of results.

Consent

The clinical teams managing patients with suspected PCa who are referred to their centre will identify potential trial participants. Patient information sheets will be provided to patients. Members of staff who are trained to take informed consent, as indicated by the PI on the delegation log for that site, will take informed consent. A model patient information sheet is shown in **Supplementary Appendix 3**.

Confidentiality

The data of the participants will be recorded into the eCRF system and analysed without any personal identifiers, by pseudoanonymised coded information. A site's source documents and identification lists will be archived in a secured facility at that centre.

Dissemination

Results of this trial will be disseminated through national and international conferences and papers. Authorship criteria will be based on recommendations of the International Committee of Medical Journal Editors. The participants and relevant patient support groups will be informed about the results of the trial.

Access to Data

Only authorised individuals within the PRIME Clinical Operations Group have access to the final data set. Individual PIs have access to their own data but not that of other sites.

WHO Trial Registration Dataset

Please see **Table 3** for the WHO trial registration dataset.

Current Protocol Version

The current protocol is V.2.0, issued 27 April 2021. The current protocol amendment number is 01. For full amendment history, please see **Table 4**.

Roles and Responsibilities

Please see **Table 5** for roles and responsibilities of the trial sponsor and involved committees.

Acknowledgements

We would like to thank our patients and funders, without whom we wouldn't be able to carry out this important study: Prostate Cancer UK & The John Black Charitable Trust, European Association of Urology Research Foundation and the Dieckmann Foundation. We thank all the international centres taking part in PRIME. We are grateful to EAU Research Foundation and the XClinical team for their support with the MARVIN database; and Sydney Lindner, Steven Lelie, Jessica Sternisa, Tyler Edwards, Adam Kulp, Jon Piper from the MIM Software Inc team. We are thankful for the trial oversight provided by our sponsor, University College London and the National Cancer Imaging Translational Accelerator trials unit.

Author Contributions

Study concept and design: ANg, AA, AN, VC, PK, FG, CA, AF, SP, PL, CSC, CBG, NM, ME, RA, YT, JD, CMM, VK. Drafting of manuscript: AA, AN, CSC, CBG, RA, YT, VK. Critical revision of the manuscript for important intellectual content: all authors. Supervision: CA, VK. All authors read and approved the final manuscript.

For peer review only

Funding

The PRIME is primarily funded by Prostate Cancer UK (grant number: TLD-PF19-004) and The John Black Charitable Trust Travelling Prize Grant (grant number: TLD-PF19-004). The EAU Research Foundation (EAU RF) and The Dieckmann Foundation also supported costs for international sites.

Declaration of Interests

AN is an academic clinical fellow funded by the National Institute for Health and Care Research. PK is an academic clinical fellow funded by the National Institute for Health and Care Research and The Urology Foundation. FG is a recipient of the 2020 Young Investigator Award (20YOUN15) funded by the Prostate Cancer Foundation / CRIS Cancer Foundation. SP is supported by the National Institute of Health and Care Research (NIHR), UCLH and UCL Biomedical Research Centre. ME receives research support from the National Institute of Health and Care Research (NIHR), UCLH and UCL Biomedical Research Centre. YT is funded by a UK NIHR Postdoctoral Fellowship and supported by the NIHR Birmingham Biomedical Research Centre. CMM is an NIHR Research Professor, and receives grants from MRC, CRUK, Movember, and Prostate Cancer UK. VK is funded by Prostate Cancer UK and The John Black Charitable Foundation. He receives speaker fees from the European Association of Urology, Singapore Urology Association, The Clinical Comms Group and Got IT consulting SL. All authors declare that there are no conflicts of interest. The views expressed in this publication are those of the authors and not necessarily those of the National Health Service, NIHR, or the Department of Health and Social Care.

REFERENCES

1. A-W. Chan, JM. Tetzlaff, PC. Gøtzsche, et al. SPIRIT 2013 explanation and elaboration: Guidance for protocols of clinical trials. *BMJ*, 346 (2013), e7586, 10.1136/bmj.e7586.
2. N. Mottet, P. Cornford, R. Van Den Bergh, et al. EAU guidelines: prostate cancer. European Association of Urology, Arnhem, The Netherlands (2021). https://uroweb.org/guideline/prostate-cancer/
3. V. Kasivisvanathan, A.S. Rannikko, M. Borghi, et al. MRI-targeted or standard biopsy for prostate-cancer diagnosis. *N Engl J Med,* 378 (2018), pp. 1767-1777, 10.1056/NEJMoa1801993.
4. National Prostate Cancer Audit - Annual Report 2019. https://www.npca.org.uk/reports/npca-annual-report-2019/
5. S. Verma, B. Turkbey, N. Muradyan, A. Rajesh, et al. Overview of dynamic contrast-enhanced MRI in prostate cancer diagnosis and management. *AJR Am J Roentgenol,* 198 (2012), pp. 1277–1288. 10.2214/AJR.12.8510
6. R. Berman, A. Brown, S. Chang, et al. DCE MRI of prostate cancer. *Abdom Radiol (NY),* 41 (2016), pp. 844-853. 10.1007/s00261-015-0589-3
7. M. Engelbrecht, H. Huisman, R. Laheij, et al. Discrimination of prostate cancer from normal peripheral zone and central gland tissue by using dynamic contrast-enhanced MR imaging. *Radiology*. 229 (2003), pp. 248-254. 10.1148/radiol.2291020200
8. M. van der Leest, E. Cornel, B. Israël, et al. Head-to-head comparison of transrectal ultrasound-guided prostate biopsy versus multiparametric prostate resonance imaging with subsequent magnetic resonance-guided biopsy in biopsy-naïve men with elevated prostate-specific antigen: a large prospective multicenter clinical study. *Eur Urol,* 75 (2019), pp. 570-578, 10.1016/j.eururo.2018.11.023
9. T. Fraum, D. Ludwig, M. Bashir, et al. Gadolinium-based contrast agents: A comprehensive risk assessment. *J Magn Reson Imaging,* 46 (2017), pp. 338-353, 10.1002/jmri.25625.
10. M. Ramalho, J. Ramalho, L. Burke, et al. Gadolinium Retention and Toxicity-An Update. *Adv Chronic Kidney Dis.* 24 (2017), pp. 138-146. 10.1053/j.ackd.2017.03.004.
11. L. Boesen, N. Nørgaard, V. Løgager, et al. Assessment of the diagnostic accuracy of biparametric magnetic resonance imaging for prostate cancer in biopsy-naive men: the

For peer review only - http://bmjopen.bmj.com/site/about/guidelines.xhtml

Biparametric MRI for Detection of Prostate Cancer (BIDOC) study. *JAMA Netw Open*, 1 (2018), p. e180219, 10.1001/jamanetworkopen.2018.0219
12. Z. Kang, X. Min, J. Weinreb, Q. Li, Z. Feng, L. Wang. Abbreviated biparametric versus standard multiparametric MRI for diagnosis of prostate cancer: a systematic review and meta-analysis. *Am J Roengenol*, 212 (2018), pp. 357-365, 10.2214/ajr.18.20103
13. M. Alabousi, J.P. Salameh, K. Gusenbauer, et al. Biparametric vs multiparametric prostate magnetic resonance imaging for the detection of prostate cancer in treatment-naïve patients: a diagnostic test accuracy systematic review and meta-analysis. *BJU Int*, 124 (2019), pp. 209-220, 10.1111/bju.14759
14. A. Ng, P. Khetrapal, V. Kasivisvanathan. Is It PRIME Time for Biparametric Magnetic Resonance Imaging in Prostate Cancer Diagnosis? *Eur Urol*, S0302-2838 (2022), pp. 01663-3, 10.1016/j.eururo.2022.02.021.
15. F. Giganti, C. Allen, M. Emberton, C.M. Moore, V. Kasivisvanathan. Prostate Imaging Quality (PI-QUAL): a new quality control scoring system for multiparametric magnetic resonance imaging of the prostate from the PRECISION trial. *Eur Urol Oncol,* 3 (2020), pp. 615-619, 10.1016/j.euo.2020.06.007.
16. J.C. Weinreb, J.O. Barentsz, P.L. Choyke, et al. PI-RADS Prostate Imaging - Reporting and Data System: 2015, Version 2. *Eur Urol*, 69 (2016), pp. 16-40, 10.1016/j.eururo.2015.08.052.

FIGURE LEGENDS

Figure 1 The PRIME trial schema – the approach prior to MRI.
(bpMRI = biparametric MRI, DCE = dynamic contrast enhanced sequence, DRE = digital rectal examination, DWI = diffusion-weighted sequence, mpMRI = multiparametric MRI, PSA = prostate specific antigen, T2W = T2-weighted sequence.)

TABLES

Table 1 Secondary outcomes in PRIME

Outcome	Time frame for assessment
Proportion of men with clinically insignificant cancer (Gleason grade 3+3 / Gleason grade group 1)	When biopsy results available, at an expected average of 30 days post-biopsy
Agreement between bpMRI and mpMRI for score of suspicion	When MRI results available, at an expected average of 30 days post-MRI
Proportion of bp-MRI scans and mpMRI whose quality was deemed adequate for reporting	When MRI results available, at an expected average of 30 days post-MRI
Agreement between bpMRI and mpMRI for radiological staging decision	When MRI results available, at an expected average of 30 days post-MRI
Agreement between bpMRI and mpMRI for treatment eligibility	When treatment eligibility is discussed in a multidisciplinary meeting, at an expected average of 30 days post biopsy.
Test performance characteristics for bpMRI and mpMRI when using the Likert scoring system in comparison to the PI-RADS scoring system	When biopsy results available, at an expected average of 30 days post-MRI
Proportion of men with clinically significant cancer missed by bpMRI and mpMRI-targeted biopsies and detected by systematic biopsy	When biopsy results available, at an expected average of 30 days post-biopsy
Cost-effectiveness of bpMRI compared to mpMRI (cost per diagnosis of prostate cancer)	At an expected average of 30 days post-intervention

(bpMRI = biparametric MRI; mpMRI = multiparametric MRI.)

For peer review only - http://bmjopen.bmj.com/site/about/guidelines.xhtml

Table 2A Participant timeline in the trial: the timeline for men enrolled to the trial prior to undergoing MRI

	**Contact with patient**				
	**Visit 0***	**Visit 1**	**Visit 2**	**Visit 3**	**Visit 4**
Screening	X	X			
PIS given	X	X			
Consent	X	X			
IIEF-5 and IPSS questionnaires	X	X			
Multiparametric MRI			X		
Radiologists reports bpMRI (T2W and DWI only)			X		
Radiologists reports mpMRI (T2W, DWI and DCE)			X		
MRI-targeted biopsy and systematic biopsy				X	
Test results given and treatment decision					X
Follow-up for further investigations from treatment decision					X
Serious adverse event	Complete as required at any time following registration				
Withdrawal form	Complete as required at any time following registration				

*(*Visit 0 is an optional teleconsult, depending on local practice. Note: where applicable, more than one visit can take place on the same day, depending on local practice (e.g. in centres where an MRI is performed on the same day as subsequent biopsies).*

IIEF-5 = The International Index of Erectile Function, IPSS = International Prostate Symptom Score, PIS = patient information sheet, bpMRI = biparametric MRI, mpMRI = multiparametric MRI, T2W = T2-weighted sequence, DWI = diffusion-weighted sequence, DCE = dynamic contrast-enhanced sequence.)

Table 2B Participant timeline in the trial: the timeline for men enrolled after undergoing multiparametric MRI as part of routine care

	**Contact with patient**				
	**Visit 0**	**Visit 1**	**Visit 2**	**Visit 3**	**Visit 4**
Screening		X			
PIS given		X			
Consent		X			
IIEF-5 and IPSS questionnaires	X				
Multiparametric MRI		X			
Radiologists reports bpMRI (T2W and DWI only)		X			
Radiologists reports mpMRI (T2W, DWI and DCE)		X			
MRI-targeted biopsy and systematic biopsy			X		
Test results given and treatment decision					X
Follow-up for further investigations from treatment decision					X
Serious adverse event	Complete as required at any time following registration				
Withdrawal form	Complete as required at any time following registration				

For peer review only

For peer review only - http://bmjopen.bmj.com/site/about/guidelines.xhtml

(IIEF-5 = The International Index of Erectile Function, IPSS = International Prostate Symptom Score, PIS = patient information sheet, bpMRI = biparametric MRI, mpMRI = multiparametric MRI, T2W = T2-weighted sequence, DWI = diffusion-weighted sequence, DCE = dynamic contrast-enhanced sequence.)

Table 3 WHO trial registration dataset

**Data category**	**Information**
Primary registry and trial identifying number	ClinicalTrials.gov: NCT04571840
Date of registration in the primary registry	October 1, 2020
Sources of monetary or material support	• Prostate Cancer UK<br>• The John Black Charitable Foundation<br>• European Association of Urology Research Foundation<br>• The Dieckmann Foundation
Primary sponsor	University College London
Secondary sponsor(s)	N/A
Contact for public queries	Mr Veeru Kasivisvanathan veeru.kasi@ucl.ac.uk<br>Div of Surgery & Interventional Sci,<br>University College London,<br>3rd Floor, Charles Bell House,<br>43-45 Foley Street, London, W1W 7TS
Contact for scientific queries	Mr Veeru Kasivisvanathan veeru.kasi@ucl.ac.uk<br>Div of Surgery & Interventional Sci,<br>University College London,<br>3rd Floor, Charles Bell House,<br>43-45 Foley Street, London, W1W 7TS
Public title / short title	Prostate Imaging Using MRI +/- Contrast Enhancement (PRIME)
Acronym	PRIME
Scientific title	A trial assessing whether biparametric MRI is non-inferior to multiparametric MRI in the diagnosis of clinically significant prostate cancer
Countries of recruitment	Argentina<br>Australia<br>Belgium<br>Brazil<br>Canada<br>Denmark<br>France<br>Finland<br>Germany<br>Italy<br>The Netherlands<br>Singapore<br>Spain<br>UK<br>USA

For peer review only

For peer review only - http://bmjopen.bmj.com/site/about/guidelines.xhtml

Health condition(s) or problem(s) studied	Prostate neoplasm
Intervention(s)	Device: MRI<br>Diagnostic Test: Multiparametric MRI +/- prostate biopsy<br>Diagnostic Test: Biparametric MRI +/- prostate biopsy
Intervention description	1. Active comparator: Multiparametric MRI (mpMRI)<br>MRI with T2-weighted, diffusion weighted and dynamic contrast enhanced sequences followed by prostate biopsy if indicated on MRI and clinical findings<br>Diagnostic Test: Multiparametric MRI +/- prostate biopsy<br><br>1. Experimental: Biparametric MRI (bpMRI)<br>MRI with T2-weighted and diffusion weighted sequences followed by prostate biopsy if indicated on MRI and clinical findings<br>Diagnostic Test: Biparametric MRI +/- prostate biopsy
Key inclusion and exclusion criteria	Inclusion Criteria:<br>1. Men at least 18 years of age referred with clinical suspicion of prostate cancer<br>2. Serum PSA ≤ 20ng/mL<br>3. Fit to undergo all procedures listed in protocol<br>4. Able to provide written informed consent<br><br>Exclusion Criteria:<br>1. Prior prostate biopsy<br>2. Prior treatment for prostate cancer<br>3. Prior prostate MRI on a previous encounter<br>4. Contraindication to MRI<br>5. Contraindication to prostate biopsy<br>6. Unfit to undergo any procedures listed in protocol
Study type	Interventional<br>Allocation: Non-Randomized<br>Intervention Model: Single Group Assignment<br>Intervention Model Description: Within-person controlled, paired cohort, diagnostic evaluation study. Participants undergo two index tests and a reference test.<br>Masking: Single (Care Provider)<br>Masking Description: Radiologist assessing MRI for suspicion of prostate cancer is blinded to the contrast sequence when reporting the biparametric MRI. After this report, they are unblinded to the contrast sequence and report the multiparametric MRI. All biopsies conducted as a result of MRI findings will be labelled as bpMRI and mpMRI, and diagnostic accuracy will be assessed against histology findings.
Date of first enrolment	05 April 2022
Target sample size	500
Recruitment status	Recruiting
Primary outcome(s)	Proportion of men with clinically significant cancer

For peer review only - http://bmjopen.bmj.com/site/about/guidelines.xhtml

For peer review only

Key secondary outcomes	• Proportion of men with clinically insignificant cancer (Gleason grade 3+3 / Gleason grade group 1)<br>• Agreement between bpMRI and mpMRI for score of suspicion<br>• Proportion of bp-MRI scans and mpMRI whose quality was deemed adequate for reporting<br>• Agreement between bpMRI and mpMRI for radiological staging decision<br>• Agreement between bpMRI and mpMRI for treatment eligibility<br>• Test performance characteristics for bpMRI and mpMRI when using the Likert scoring system in comparison to the PI-RADS scoring system<br>• Proportion of men with clinically significant cancer missed by bpMRI and mpMRI-targeted biopsies and detected by systematic biopsy<br>• Cost-effectiveness of bpMRI compared to mpMRI (cost per diagnosis of prostate cancer)

Table 4 Revision chronology for amendments to protocol

**Protocol version to date**	**Reasons for amendments**
V.1.0, issued 24 August 2020	Original protocol
V.2.0, issued 27 April 2021	Main reasons for amendment: minor changes to make existing trial documents clearer. Main changes:<br>1. Updated Section 18 Record Keeping and Archiving. Added the sentence, "Identifiable data will be kept by the site for 10 years, and non-identifiable data will be kept for a minimum of 20 years."<br>2. Version number and date added to all pages.

Table 5 Roles and responsibilities in the PRIME Trial

**Role**	**Details and responsibilities**
Trial sponsor	University College London (UCL)<br>Sponsor's Edge reference: 135819<br>Email: Rand.D@uclh.nhs.uk<br>The trial sponsor did not provide any funding for the study. University College London has the role of research governance sponsor of PRIME. UCL adopted the study as sponsor after the UCL CCTU carried out a trial adoption process which involved the UCL CCTU reviewing the protocol to ensure it conformed to high standards of trial conduct and met the governance requirements of UCL. The UCL CCTU is responsible for oversight of the trial. The sponsor plays no role in data collection, management, analysis and interpretation of data, writing of the report or the decision to submit the report for publication.
PRIME Operations Group	The PRIME Operations Group consists of the chief investigator, the Clinical Operations Group, National Cancer Imaging Translational Accelerator, the UCL Surgical and Interventional Trials Unit and the eCRF database managers. This group is responsible for:<br>• Study planning<br>• Preparation of protocol and revisions<br>• Assistance with international review board/independent ethics committee applications

For peer review only

	• Preparation of investigators brochure and CRFs<br>• Organisation of steering committee meetings<br>• Provide annual progress reports to the ethics committee<br>• Reporting serious adverse events to the sponsor and ethics committee when necessary<br>• Responsible for trial master file<br>• Budget administration and contractual issues with individual centres<br>• Advice for PIs<br>• Site initiation visits<br>• Data verification and management<br>• Central monitoring and resolving data queries with clinicians and nurses at the trial sites<br>• Maintenance of the trial Information Technology (IT) system<br>• Publication of study reports
Principal Investigator	At each participating site, the PI is responsible for the conduct of the clinical trial to ensure the safety of participants and the reliability and robustness of the data generated. They will be responsible for identification, recruitment, data collection and completion of CRFs, along with follow-up of trial patients and adherence to trial protocol. The PI as leader of the research team may delegate their duties to members of their team.
Global Trial Steering Committee	The NCITA global prostate trial steering committee (TSC) is responsible for the governance of the PRIME Study, and they have delegated safety to a data monitoring subcommittee (DMSC).<br><br>Roles and responsibilities:<br>To act as the oversight body for up to five prostate cancer studies on behalf of the Sponsor and Funders. In addition, the independent members will form a sub-committee to review safety. The role of the TSC is to provide oversight for the studies and provide advice through its Chair to the Chief Investigators (CI), work in tandem with the Data Monitoring Sub-Committee (DMSC), Sponsor, Funders and host institution on all aspects of the studies. The rights, safety and well-being of the study participants are the most important consideration and should prevail over the interests of science and society.

For peer review only

For peer review only - http://bmjopen.bmj.com/site/about/guidelines.xhtml

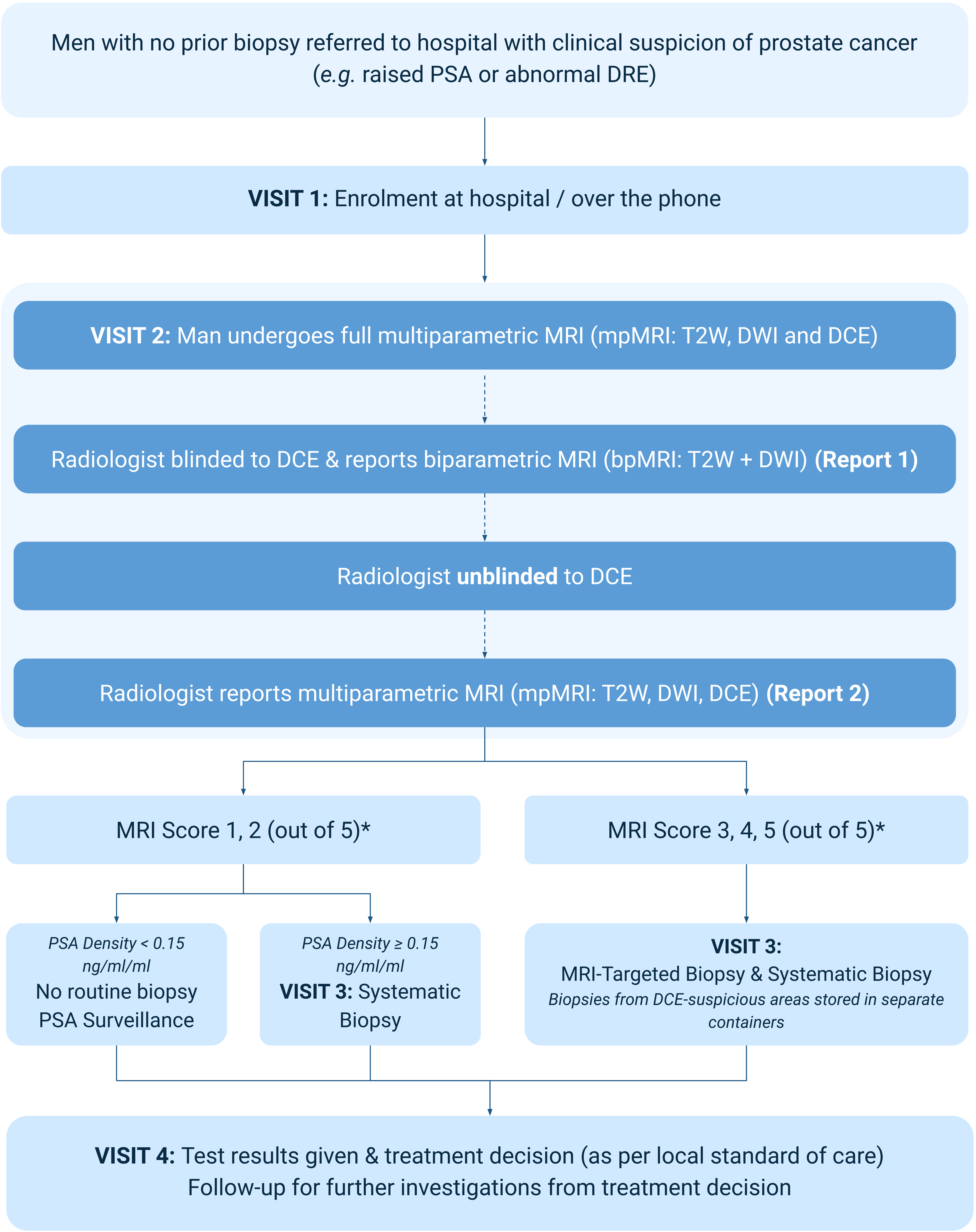
Men with no prior biopsy referred to hospital with clinical suspicion of prostate cancer (e.g. raised PSA or abnormal DRE)
VISIT 1: Enrolment at hospital / over the phone
VISIT 2: Man undergoes full multiparametric MRI (mpMRI: T2W, DWI and DCE)
Radiologist blinded to DCE & reports biparametric MRI (bpMRI: T2W + DWI) (Report 1)
Radiologist unblinded to DCE
Radiologist reports multiparametric MRI (mpMRI: T2W, DWI, DCE) (Report 2)
MRI Score 1, 2 (out of 5)*
MRI Score 3, 4, 5 (out of 5)*
PSA Density < 0.15 ng/ml/ml
No routine biopsy
PSA Surveillance
PSA Density ≥ 0.15 ng/ml/ml
VISIT 3: Systematic Biopsy
VISIT 3:
MRI-Targeted Biopsy & Systematic Biopsy
Biopsies from DCE-suspicious areas stored in separate containers
VISIT 4: Test results given & treatment decision (as per local standard of care)
Follow-up for further investigations from treatment decision

TRIAL IDENTIFIER: PARTICIPANT INITIALS:

Reporting Proforma (bpMRI):

Report 1 – Biparametric MRI (bpMRI) Report

This report should be completed **without looking at the contrast sequence.**

If applicable, complete the Target boxes & link these to your drawings of the Targets (e.g. with lines / colours)

Target 1	Likert:	PIRADS:
Target 2	Likert:	PIRADS:
Target 3	Likert:	PIRADS:
Target 4	Likert:	PIRADS:

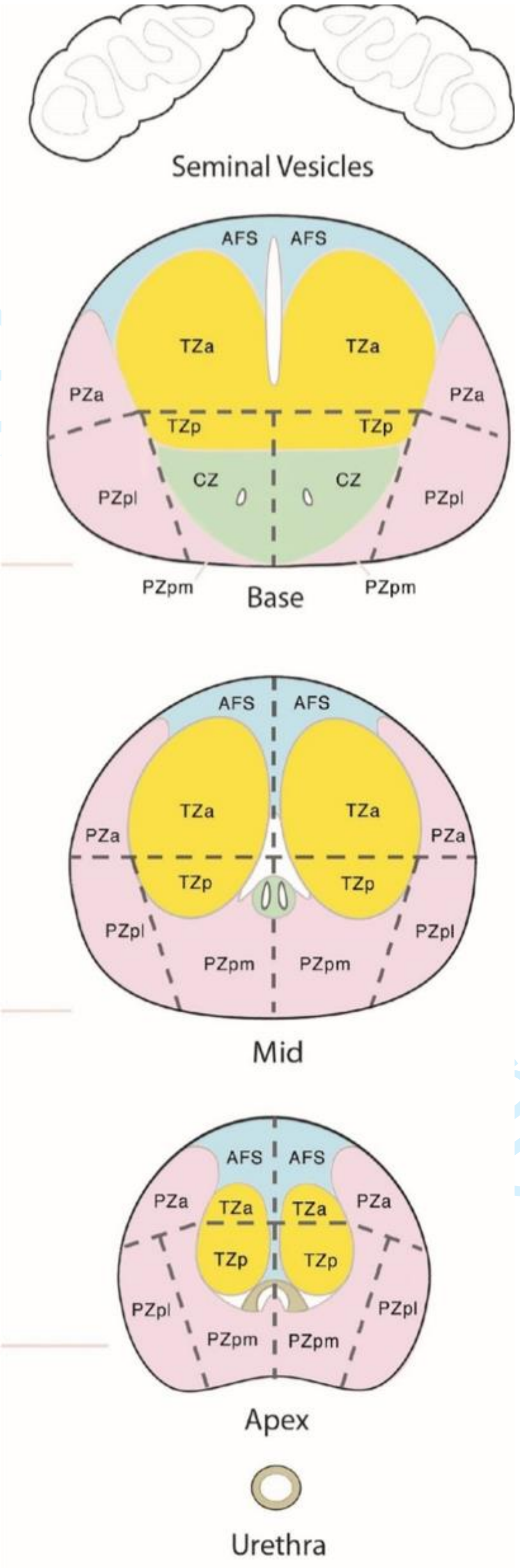

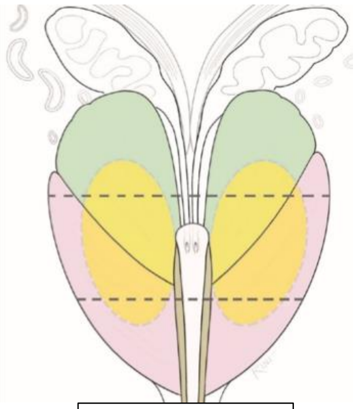

Coronal plane

Reminder of Likert Score: Likelihood of target containing significant cancer:

1 = Highly unlikely

2 = Unlikely

3 = Equivocal

4 = Likely

5 = Highly Likely

In the case of diffuse changes on **both sides** of the prostate scoring ≥3 (Likert), the diffuse changes on each side of the prostate can be arbitrarily treated as **separate targets.** "Diffuse change" is defined as an intermediate or low T2 signal that occupies the majority of at least one side of the peripheral zone, without a defined border

For peer review only

For peer review only - http://bmjopen.bmj.com/site/about/guidelines.xhtml

TRIAL IDENTIFIER: PARTICIPANT INITIALS:

1. Radiologists should **first** annotate, draw and label the diagram on the first page with **up to 3** suspicious areas scoring ≥ 3 on the Likert scale (L) of suspicion (1–5). Clinical information is permitted to be used to influence the score.
2. Radiologists should then score suspicious areas **strictly** using the PI-RADS v2.1 (P) criteria, **without** allowing clinical information to influence the score.
3. If an additional area of suspicion is identified when scoring with PI-RADS v2.1 that was **not** present on Likert, please draw on this 4th suspicious area.

A maximum of **4 targets** can be drawn on this report.

---

1. Every lesion **must have both** a Likert and PI-RADS v2.1 score marked on.
2. Mark the **most suspicious** area, "Target 1".
    a. Mark the **next most suspicious area**, "Target 2".
    b. Mark the **subsequent most suspicious area**, "Target 3" and so on.
3. **On the diagram above, every** lesion drawn must have the following marked and labelled:
    a. Target number
    b. Likert score
    c. PI-RADS v2.1 score
4. Please then insert these into **Table 1** and fill out the rest of the proforma.

e.g. Target 1. Likert 3. PI-RADS 1.

MRI Scanner and Clinical Information

<table>
<tr><td>Patient age (years):</td><td></td><td>PSA (ng/ml):</td><td></td><td rowspan="3">Which MRI scanner was used?
1. ☐ SCANNER ONE
2. ☐ SCANNER TWO
3. ☐ SCANNER THREE</td></tr>
<tr><td>MRI volume of prostate (ml):</td><td></td><td>PSA Density (ng/ml/ml):</td><td></td></tr>
<tr><td>Field Strength of Magnet</td><td>☐ 1.5T ☐ 3T</td><td></td><td></td></tr>
</table>

Confirmation of blinding

Confirmation by another individual / system that the radiologist is **blinded** to DCE images (**mandatory**)	☐ Yes	☐ No

---

For peer review only - http://bmjopen.bmj.com/site/about/guidelines.xhtml

TRIAL IDENTIFIER: PARTICIPANT INITIALS:

Table 1. Please only enter Targets below if the Likert or PI-RADS v2.1 score is ≥ 3.

**TARGET SPECIFIC INFORMATION**	TARGET 1	TARGET 2	TARGET 3	TARGET 4
Location of suspicious area(s) (select **one** option):	☐ Right<br>☐ Left<br>☐ Bilateral	☐ Right<br>☐ Left<br>☐ Bilateral	☐ Right<br>☐ Left<br>☐ Bilateral	☐ Right<br>☐ Left<br>☐ Bilateral
Location in prostate according to PI-RADS v2.1 41-sector diagram (select **the one main** location which contains the target):	☐ Base<br>☐ Mid<br>☐ Apex<br>☐ Seminal Vesicle	☐ Base<br>☐ Mid<br>☐ Apex<br>☐ Seminal Vesicle	☐ Base<br>☐ Mid<br>☐ Apex<br>☐ Seminal Vesicle	☐ Base<br>☐ Mid<br>☐ Apex<br>☐ Seminal Vesicle
Main sector which contains the lesion according to PI-RADS v2.1 41-sector diagram (write **one**, *e.g.* "PZpl"):				
Likert score of suspicion (1–5):				
PI-RADS v2.1 score of suspicion (1–5):				
Target appearance (select **one**):<br>The default is focal, unless there is diffuse change in the peripheral zone	☐ Focal<br>☐ Diffuse	☐ Focal<br>☐ Diffuse	☐ Focal<br>☐ Diffuse	☐ Focal<br>☐ Diffuse
Biaxial diameter on sequence where it was largest, in axial plane (mm x mm):				
Sequence used to measure biaxial diameter (select **one**):	☐ T2<br>☐ High b<br>☐ ADC	☐ T2<br>☐ High b<br>☐ ADC	☐ T2<br>☐ High b<br>☐ ADC	☐ T2<br>☐ High b<br>☐ ADC

Please complete the **overall scores** regardless of whether there are any Targets identified above:

**Overall patient Likert score**<br>Enter the highest Likert score		**Overall patient PIRADS v2.1 score**<br>Enter the highest PI-RADS v2.1 score	

If there are no Targets scoring ≥ 3 on either scoring system, then the overall Likert and PI-RADS v2.1 score will be 1 or 2.

For peer review only - http://bmjopen.bmj.com/site/about/guidelines.xhtml

For peer review only

TRIAL IDENTIFIER: PARTICIPANT INITIALS:

Table 2. Staging information. Complete **only if** a Target has been identified above:

Radiological stage:	☐ T2a	☐ T2b	☐ T2c	☐ T3a	☐ T3b	☐ T4
	Radiological T3a = unequivocal extracapsular disease					
Likelihood of **right**-sided extracapsular spread*:<br>1 = highly **unlikely**, 3 = equivocal, 5 = highly **likely**	☐ 1	☐ 2	☐ 3	☐ 4	☐ 5	
Likelihood of **left**-sided extracapsular spread*:	☐ 1	☐ 2	☐ 3	☐ 4	☐ 5	
Likelihood of **right** seminal vesicle involvement:	☐ 1	☐ 2	☐ 3	☐ 4	☐ 5	
Likelihood of **left** seminal vesicle involvement:	☐ 1	☐ 2	☐ 3	☐ 4	☐ 5	
Likelihood of urethral sphincter involvement:	☐ 1	☐ 2	☐ 3	☐ 4	☐ 5	
Likelihood of bladder neck involvement:	☐ 1	☐ 2	☐ 3	☐ 4	☐ 5	
Likelihood of rectal involvement:	☐ 1	☐ 2	☐ 3	☐ 4	☐ 5	

**See* PI-RADS v2.1 guidelines for examples of features suggestive of extracapsular spread.

MRI Quality: Please **complete** this for **all** MRIs regardless of whether a Target was identified:

Was there a problem with the quality of the T2W sequence?	☐ Yes			☐ No
Was there a problem with the quality of the DWI sequence?	☐ Yes			☐ No
If there were problems, please describe these (tick **all** that apply):<br>For T2W:	☐ Rectal air	☐ Movement artefact	☐ Prosthesis	☐ Other
For DWI:	☐ Rectal air	☐ Movement artefact	☐ Prosthesis	☐ Other
If other, please describe:				
Was the quality of the scan sufficient for you to make a diagnostic assessment?	☐ Yes	☐ No		
Hypothetically, if this patient only had this biparametric MRI scan:<br>• Would you typically have recommended a **repeat bpMRI?**	☐ Yes	☐ No		
• Would you typically have recommended a **contrast sequence** to be done?	☐ Yes	☐ No		

Radiologist (Forename, Surname):		Date of MRI:	
		Date of Report:	

For peer review only - http://bmjopen.bmj.com/site/about/guidelines.xhtml

TRIAL IDENTIFIER: PARTICIPANT INITIALS:

Reporting Proforma (mpMRI):

Report 2 – Multiparametric MRI (mpMRI) Report

The same radiologist should annotate the diagrams below after they are **unblinded** to the **DCE sequence**. This report will be used by the biopsy operator to perform **targeted biopsy**.

A total of **maximum 8 suspicious areas scoring ≥ 3 on either Likert or PI-RADS v2.1** can be annotated in this report.

PART ONE: TARGETS SEEN ON BPMRI

1. First, copy any targets drawn on **Report 1** (bpMRI) onto this report (**Report 2 – mpMRI**).
   a. Draw them on the diagram.
   b. Specify their biparametric MRI status (bpMRI +ve or bpMRI -ve) when you label each lesion.
   c. Add the information about each target to **Table 1** as indicated.
2. Upon viewing the **DCE findings**, for each of these lesions, please specify their multi-parametric MRI status (mpMRI +ve or mpMRI -ve) on the diagram then specify **updated** Likert (L) and PI-RADS v2.1 (P) scores on mpMRI.

For peer re

Flow diagram: how to complete this proforma for lesions identified on bpMRI (Report 1)

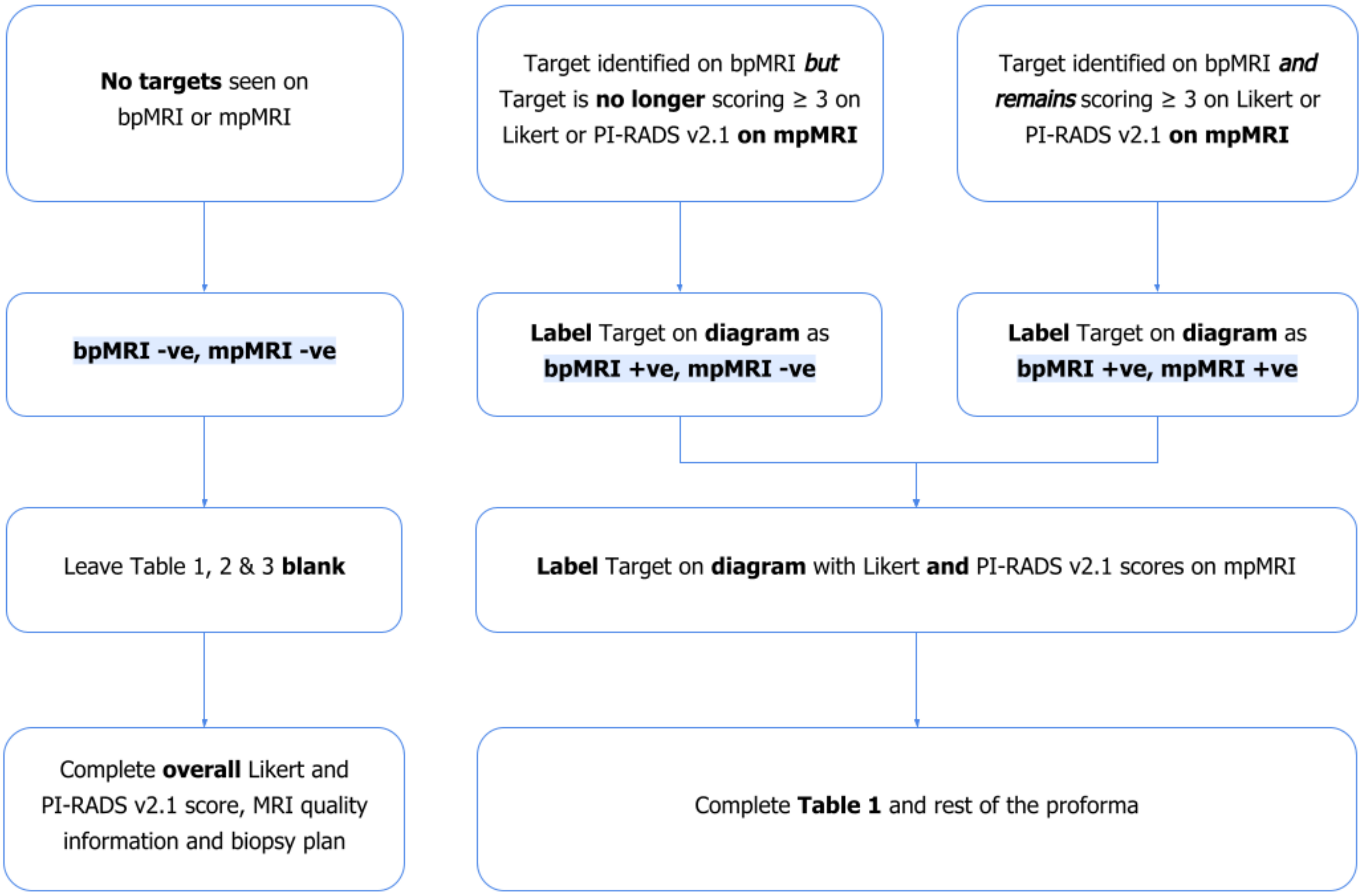

For peer review only - http://bmjopen.bmj.com/site/about/guidelines.xhtml

TRIAL IDENTIFIER: PARTICIPANT INITIALS:

Please draw any Targets on this diagram and label them according to the flow diagram on Page 1

If applicable, complete the Target boxes & link these to your drawings of the Targets (e.g. with lines / colours)

Target 1	**bpMRI**<br>Likert:<br>PIRADS:	**mpMRI**<br>Likert:<br>PIRADS:

Target 2	**bpMRI**<br>Likert:<br>PIRADS:	**mpMRI**<br>Likert:<br>PIRADS:

Target 3	**bpMRI**<br>Likert:<br>PIRADS:	**mpMRI**<br>Likert:<br>PIRADS:

Target 4	**bpMRI**<br>Likert:<br>PIRADS:	**mpMRI**<br>Likert:<br>PIRADS:

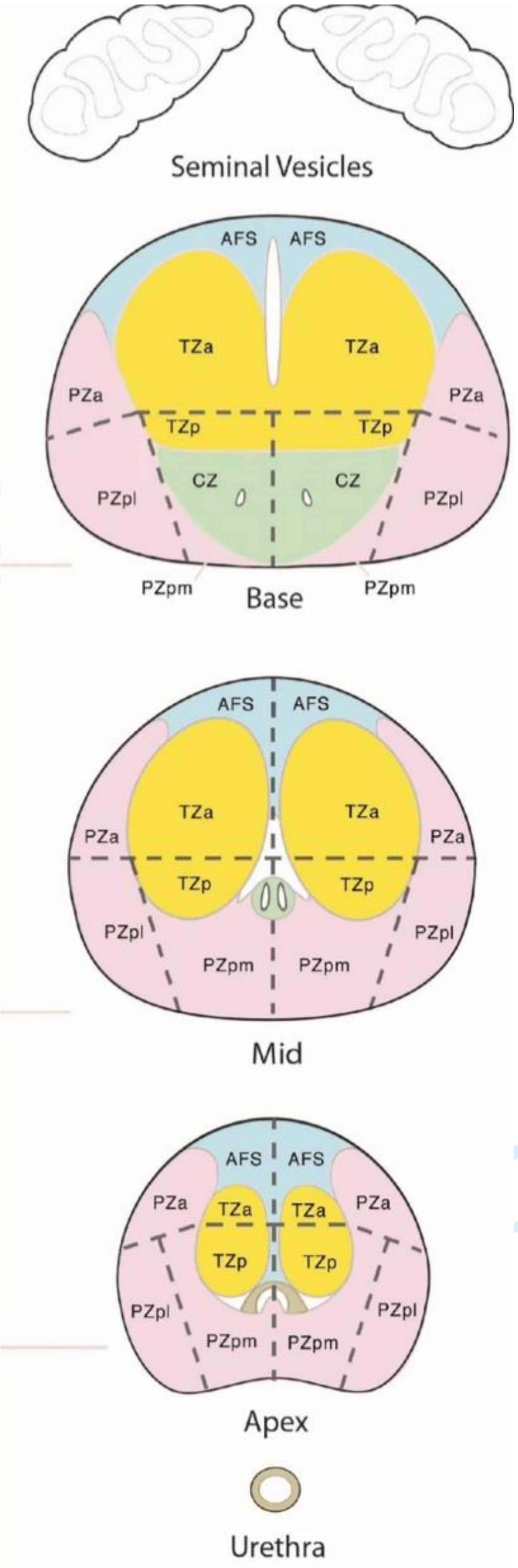

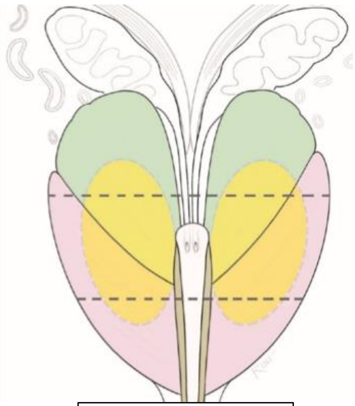

Coronal plane

**DCE-** Target 1	Likert:<br>PIRADS:

**DCE-** Target 2	Likert:<br>PIRADS:

**DCE-** Target 2	Likert:<br>PIRADS:

**DCE-** Target 4	Likert:<br>PIRADS:

In the case of diffuse changes on **both sides** of the prostate scoring ≥3 (Likert), the diffuse changes on each side of the prostate can be arbitrarily treated as **separate Targets.** **"**Diffuse change" is defined as an intermediate or low T2 signal that occupies the majority of at least one side of the peripheral zone, without a defined border.

For peer review only - http://bmjopen.bmj.com/site/about/guidelines.xhtml

TRIAL IDENTIFIER: PARTICIPANT INITIALS:

MRI Scanner and Clinical Information. Complete for all patients:

Patient age (years)		PSA (ng/ml):		
MRI volume of prostate (ml):		PSA Density (ng/ml/ml):		

Table 1. Information from Targets **originally** identified on the **biparametric MRI** (if applicable):

**TARGET SPECIFIC INFORMATION**	TARGET 1	TARGET 2	TARGET 3	TARGET 4
**COPY FROM REPORT 1 (BPMRI):**				
Location of suspicious area(s) (select **one** option):	☐ Right<br>☐ Left<br>☐ Bilateral	☐ Right<br>☐ Left<br>☐ Bilateral	☐ Right<br>☐ Left<br>☐ Bilateral	☐ Right<br>☐ Left<br>☐ Bilateral
Location in prostate according to PI-RADS v2.1 41-sector diagram (select **the one main** location which contains the target):	☐ Base<br>☐ Mid<br>☐ Apex<br>☐ Seminal Vesicle	☐ Base<br>☐ Mid<br>☐ Apex<br>☐ Seminal Vesicle	☐ Base<br>☐ Mid<br>☐ Apex<br>☐ Seminal Vesicle	☐ Base<br>☐ Mid<br>☐ Apex<br>☐ Seminal Vesicle
Main sector which contains the lesion according to PI-RADS v2.1 41-sector diagram (write **one** sector, *e.g.* “PZpl”):				
Biparametric MRI Likert score (1–5):				
Biparametric MRI PI-RADS v2.1 score (1–5):				
**RE-ASSESS, TAKING INTO ACCOUNT INFORMATION FROM DCE SEQUENCE (MPMRI):**				
Multiparametric MRI Likert score (1–5):				
Multiparametric MRI PI-RADS v2.1 score (1–5):				
Target appearance (select **one**):	☐ Focal<br>☐ Diffuse	☐ Focal<br>☐ Diffuse	☐ Focal<br>☐ Diffuse	☐ Focal<br>☐ Diffuse
Biaxial diameter on sequence where it was largest, in axial plane (mm x mm):				
Sequence used to measure biaxial diameter (select **one**):	☐ T2 ☐ High b<br>☐ ADC ☐ DCE	☐ T2 ☐ High b<br>☐ ADC ☐ DCE	☐ T2 ☐ High b<br>☐ ADC ☐ DCE	☐ T2 ☐ High b<br>☐ ADC ☐ DCE

For peer review only - http://bmjopen.bmj.com/site/about/guidelines.xhtml

TRIAL IDENTIFIER: PARTICIPANT INITIALS:

PART TWO: NEW DCE-TARGETS ON DYNAMIC CONTRAST ENHANCED SEQUENCE

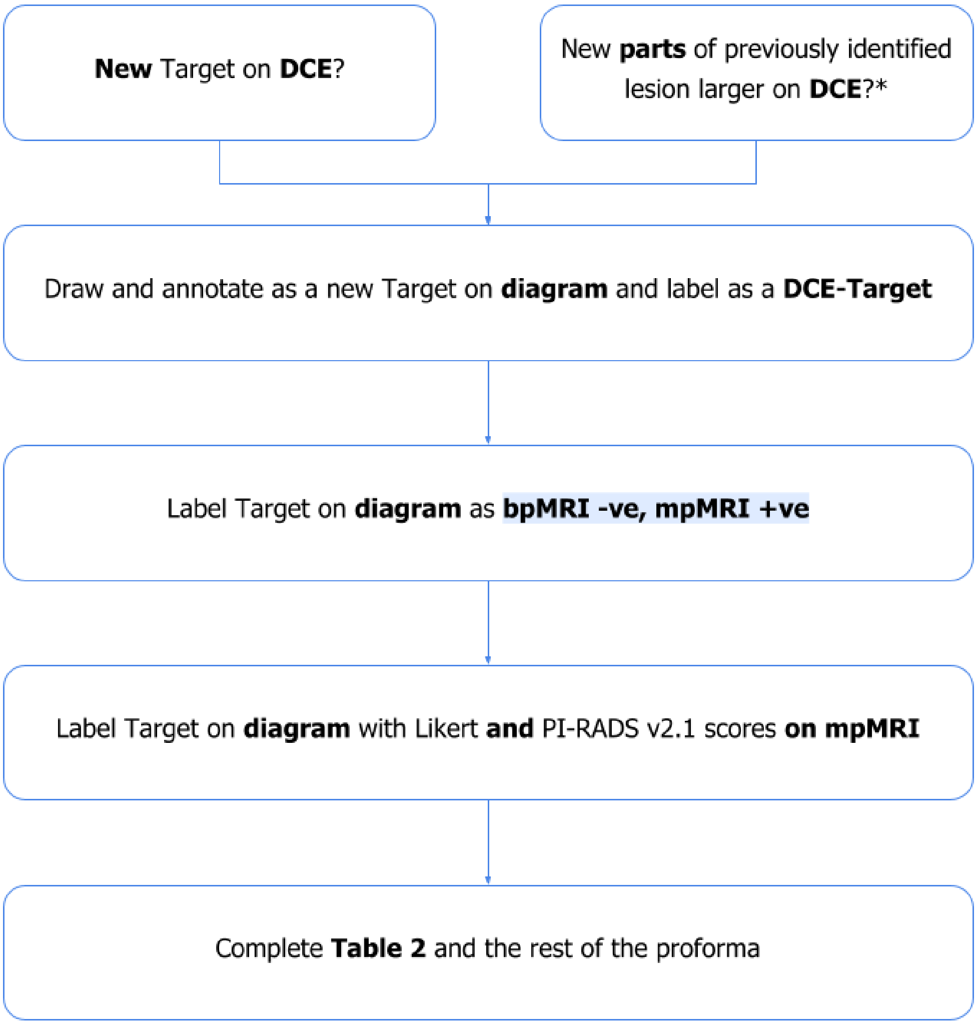

Please note: this is a **subjective decision** by the radiologist as to whether new parts of an existing lesion on bpMRI would need to be declared as **a new target** in order **not to be missed on biopsy**. A clear example of when to declare a new DCE-Target would be if the non-overlapping part of the lesion on DCE crosses into a new sector on the PI-RADSv2.1 sector diagram

---

5. Any new targets should be labelled **DCE-Target-x.**
   a. The first new, most suspicious, target should be **DCE-Target-1**. The second if applicable, **DCE-Target-2** and so on.
6. A maximum of **4 new targets** can be drawn on this report (**Report 2**).
   a. Thus, a maximum of **8 targets** can be drawn in total (4 carried over from **Report 1** and 4 new DCE targets).
7. **On the diagram on Page 2, every** lesion drawn must have the following marked and labelled:
   a. Target number
   b. bpMRI status (positive or negative)
   c. mpMRI status (positive or negative)
   d. Likert score for mpMRI
   e. PI-RADS v2.1 score for mpMRI

   e.g. DCE-Target-1. bpMRI negative. mpMRI positive. Likert 4. PI-RADS 2.

8. Then complete **Table 2** and the rest of the MRI proforma.

For peer review only - http://bmjopen.bmj.com/site/about/guidelines.xhtml

TRIAL IDENTIFIER: PARTICIPANT INITIALS:

Table 2. Information from Targets identified **ONLY** by DCE, which were **not** identified on the **biparametric MRI** (if applicable). If there are no DCE-Targets then leave Table 2 blank & move onto overall patient Likert & PI-RADs scores):

**TARGET SPECIFIC INFORMATION**	DCE-TARGET 1	DCE-TARGET 2	DCE-TARGET 3	DCE-TARGET 4
DCE-Target (select if **new** lesion or **part of existing lesion bigger on DCE**):	☐ New<br>☐ Existing	☐ New<br>☐ Existing	☐ New<br>☐ Existing	☐ New<br>☐ Existing
Location of suspicious area(s) (select **one**):	☐ Right<br>☐ Left<br>☐ Bilateral	☐ Right<br>☐ Left<br>☐ Bilateral	☐ Right<br>☐ Left<br>☐ Bilateral	☐ Right<br>☐ Left<br>☐ Bilateral
Location in prostate according to PI-RADS v2.1 41-sector diagram (select **the one main** location which contains the target):	☐ Base<br>☐ Mid<br>☐ Apex<br>☐ Seminal Vesicle	☐ Base<br>☐ Mid<br>☐ Apex<br>☐ Seminal Vesicle	☐ Base<br>☐ Mid<br>☐ Apex<br>☐ Seminal Vesicle	☐ Base<br>☐ Mid<br>☐ Apex<br>☐ Seminal Vesicle
Main sector which contains the lesion according to PI-RADS v2.1 41-sector diagram (write **one**, *e.g.* "PZpl"):				
Multiparametric MRI Likert score (1–5):				
Multiparametric MRI PI-RADS v2.1 score (1–5):				
Target appearance (select **one**):	☐ Focal<br>☐ Diffuse	☐ Focal<br>☐ Diffuse	☐ Focal<br>☐ Diffuse	☐ Focal<br>☐ Diffuse
Biaxial diameter on dominant sequence in axial plane (mm x mm):				
Looking back again at the T2W and DWI **only**, is the DCE-target identified here actually visible on the bpMRI?	☐ No<br>☐ Yes	☐ No<br>☐ Yes	☐ No<br>☐ Yes	☐ No<br>☐ Yes
If you answered **Yes**, please specify whether the lesion was missed on 1st look ***or*** whether it was seen but scored a 1 or 2 on PI-RADS v2.1 **and** Likert	☐ Missed on 1st look<br>☐ Seen on 1st look but scored a 1 or 2	☐ Missed on 1st look<br>☐ Seen on 1st look but scored a 1 or 2	☐ Missed on 1st look<br>☐ Seen on 1st look but scored a 1 or 2	☐ Missed on 1st look<br>☐ Seen on 1st look but scored a 1 or 2

For peer review only - http://bmjopen.bmj.com/site/about/guidelines.xhtml

TRIAL IDENTIFIER: PARTICIPANT INITIALS:

Please complete the **overall scores** regardless of whether there are any Targets identified above:

**Overall patient Likert score**<br>Enter the highest Likert score on **either** biparametric MRI or multiparametric MRI		**Overall patient PI-RADS v2.1 score**<br>Enter the highest PI-RADS v2.1 score on **either** biparametric MRI or multiparametric MRI	

Please note: if a lesion was suspicious on biparametric MRI but **not** suspicious on mpMRI (*i.e.* bpMRI +ve, mpMRI -ve), it should still be biopsied if either the Likert or PI-RADS v2.1 score on bpMRI is ≥ 3. This highest score on either bpMRI or mpMRI should be entered above.

Table 3. Staging information. Complete **only** if a Target has been identified above. Select **one option** each time:

Radiological stage:	☐ T2a	☐ T2b	☐ T2c	☐ T3a	☐ T3b	☐ T4
	Radiological T3a = unequivocal extracapsular disease					
Likelihood of **right**-sided extracapsular spread*:<br>1 = highly **unlikely**, 3 = equivocal, 5 = highly **likely**	☐ 1	☐ 2	☐ 3	☐ 4	☐ 5	
Likelihood of **left**-sided extracapsular spread*:	☐ 1	☐ 2	☐ 3	☐ 4	☐ 5	
Capsular involvement on **DCE**:	☐ No	☐Yes, on **right**	☐Yes, on **left**	☐Yes, on **both sides**		
Likelihood of **right** seminal vesicle involvement:	☐ 1	☐ 2	☐ 3	☐ 4	☐ 5	
Likelihood of **left** seminal vesicle involvement:	☐ 1	☐ 2	☐ 3	☐ 4	☐ 5	
Seminal vesicle involvement on **DCE**:	☐ No	☐Yes, on **right**	☐Yes, on **left**	☐Yes, on **both sides**		
Likelihood of urethral sphincter involvement:	☐ 1	☐ 2	☐ 3	☐ 4	☐ 5	
Urethral sphincter involvement on **DCE**:	☐ No	☐Yes, on **right**	☐Yes, on **left**	☐Yes, on **both sides**		
Likelihood of bladder neck involvement:	☐ 1	☐ 2	☐ 3	☐ 4	☐ 5	
Bladder neck involvement on **DCE**:	☐ No	☐ Yes				
Likelihood of rectal involvement:	☐ 1	☐ 2	☐ 3	☐ 4	☐ 5	
Rectal wall involvement on **DCE**:	☐ No	☐ Yes				

**See* PI-RADS v2.1 guidelines for examples of features suggestive of extracapsular spread.

For peer review only - http://bmjopen.bmj.com/site/about/guidelines.xhtml

TRIAL IDENTIFIER: PARTICIPANT INITIALS:

MRI Quality. Please **complete** this for all MRIs regardless of whether a Target was identified:

Was there a problem with the quality of the DCE sequence?	☐ Yes			☐ No
If problems with DCE, please specify: Tick **all** that apply	☐ Rectal air	☐ Movement artefact	☐ Prosthesis	☐ Other
If other, please describe:				
Was the quality of the scan sufficient for you to make a diagnostic assessment?	☐ Yes		☐ No	
Based on the quality of the mpMRI scan and your typical practice, would you recommend a repeat multiparametric MRI be performed?	☐ Yes		☐ No	

Biopsy protocol guidelines

It is **mandatory** to follow these recommendations below:

Number of MRI targets	Location of MRI targets in prostate	Number of MRI-targeted biopsy cores	Number of contralateral systematic cores	Total number of biopsy cores
0	If PSA Density is < 0.15ng/ml/ml			0
0	If PSA Density is ≥ 0.15ng/ml/ml, then 12 systematic biopsy cores are taken (6 from each side)			12
1	Unilateral	4	6	10
2	Unilateral	8	6	14
3	Unilateral	12	6	18
4–8	Unilateral	16–32	6	22–38
1	Bilateral (*e.g.* crossing midline)	4	0	4
2	Bilateral	8	0	8
3	Bilateral	12	0	12
4–8	Bilateral	16–32	0	16–32

Note: For 4–8 MRI targets, determine the number of MRI-targeted cores by using the principle of 4 cores per MRI target.

For peer review only - http://bmjopen.bmj.com/site/about/guidelines.xhtml

TRIAL IDENTIFIER: PARTICIPANT INITIALS:

Recommended Biopsy Plan for biopsy operator to follow

The radiologist should now complete this biopsy plan which should be passed directly to the person performing biopsy (if one is required) along with the labelled diagram on Page 2:

Even if radiologists do not typically write biopsy plans, we request they do this here following the protocol in the table above, in order to reduce errors between linking the MRI information to the protocol biopsy plan.

Number of MRI-targets to biopsy with MRI-targeted biopsy:<br>(*Note:* Targets which are only suspicious on bpMRI should still be biopsied. The number of MRI-targets for biopsy therefore includes MRI targets identified only on bpMRI, only on mpMRI or on both bpMRI and mpMRI and on either the Likert scoring system or the PIRADsv2.1 scoring system)	
Total number of MRI-targeted biopsy cores to be taken:<br>(*Note:* 4 biopsy cores should be taken per lesion)	
Total number of systematic biopsy cores to be taken:<br>(*Note:* Systematic cores should be peripheral zone-focused cores)	
Number of systematic cores to be taken from **right** side of prostate:<br>(*Note:* do not take systematic cores from the same side as an MRI target)	
Number of systematic cores to be taken from **left** side of prostate:<br>(*Note:* do not take systematic cores from the same side as an MRI target)	
Total number of systematic and targeted cores to be taken	

Radiologist<br>(Forename, Surname):		Date:	

For peer review only - http://bmjopen.bmj.com/site/about/guidelines.xhtml

Supplementary Appendix 2: Detailed PRIME Biopsy Plans

To be pragmatic and allow results to be generalisable to biopsy practice around the world, biopsies can be performed transperineally (**Figures 1** and **2**) or transrectally (**Figures 3 and 4**) as per local practice. We split this Appendix into these sections, respectively.

If there is an MRI lesion (scores 3, 4 or 5 on *either* Likert or PI-RADS v2.1 scoring systems), then MRI-targeted biopsy and some limited contralateral systematic biopsy should be performed. MRI-targeted biopsy should be performed **first,** with 4 cores per suspicious area. Then the systematic biopsy cores should be taken but avoid taking biopsies from the same side of the prostate that targeted biopsies were taken from.

Table of Contents

Systematic Transperineal Biopsy Schema 2
- Non-suspicious MRI but a PSA Density of ≥ 0.15ng/mL/mL scenario 2
- Suspicious MRI lesion scenarios 4
  - *Single lesion scenario* 4
  - *Bilateral lesions scenario* 4
  - *Lesion crossing midline scenario* 5
  - *Bilateral diffuse change on Likert scoring scenario* 5
  - *New lesion is revealed on DCE sequence scenario* 6
  - *New part of existing lesion is revealed on DCE sequence scenario* 6

Systematic Transrectal Biopsy Schema 7
- Non-suspicious MRI but a PSA Density of ≥ 0.15ng/mL/mL scenario 7
- Suspicious MRI lesion scenarios 9
  - *Single lesion scenario* 9
  - *Bilateral lesions scenario* 10
  - *Lesion crossing midline scenario* 11
  - Bilateral diffuse change on Likert scoring scenario 12
  - *New lesion is revealed on DCE sequence scenario* 13
  - *New part of existing lesion is revealed on DCE sequence scenario* 14

Summary Biopsy Guidelines 16

For peer review only - http://bmjopen.bmj.com/site/about/guidelines.xhtml

Systematic Transperineal Biopsy Schema

Figures 1 and **2A-F** depict examples of how to perform the systematic biopsy in the **absence** of an MRI lesion and in the **presence** of MRI lesions, respectively.

Non-suspicious MRI but a PSA Density of ≥ 0.15ng/mL/mL scenario

In patients with a **non-suspicious MRI but a PSA Density of ≥ 0.15ng/mL/mL**, 12-core systematic biopsy should be performed (**Figure 1**).

The number of systematic cores that should be taken per patient is **12.**

Systematic biopsy cores are taken from:

- Right anterior zone (2 cores)
- Right mid zone (2 cores)
- Right posterior zone (2 cores)
- Left anterior zone (2 cores)
- Left mid zone (2 cores)
- Left posterior zone (2 cores)

Systematic biopsy cores should be stored and labelled in a way that their **location** can be identified when the pathologist reports the result.

Figure 1. The transperineal biopsy schema for men with a **non-suspicious MRI** (scores 1 or 2 on both Likert and PI-RADS v2.1 scoring systems) *but* a PSA Density of ≥0.15ng/mL/mL, undergoing 12-core systematic biopsy.

For peer review only

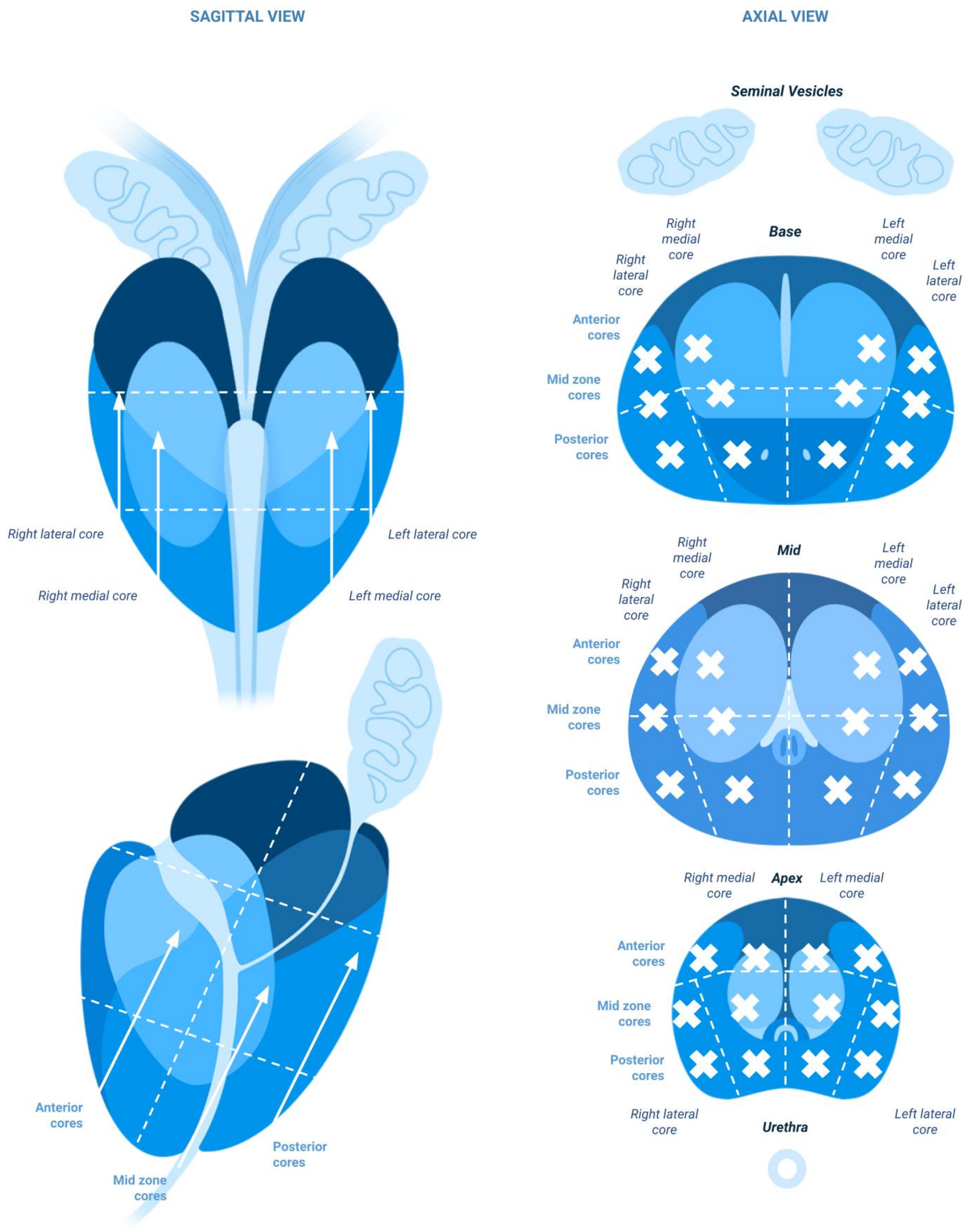

For each pair of biopsies – one core is more lateral, one core is more medial. From anterior—posterior, there are 3 planned rows of biopsies – anterior, mid zone, posterior. Avoid biopsy around the urethra.

Designed by Aqua Asif

Suspicious MRI lesion scenarios

Figure 2. Examples of how to perform transperineal biopsies in patients with an MRI Target (scores 3, 4 or 5 on *either* Likert or PI-RADS v2.1 scoring systems).

2A. Single lesion example.

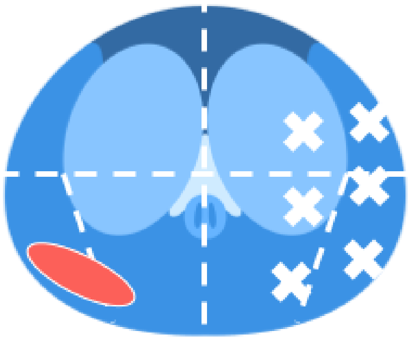

This is a single lesion in the right mid-gland peripheral zone posteromedially (PZ pm) and posterolaterally (PZ pl).

- Take **4 targeted biopsies** from the Target.
- Then take **6 peripheral zone focused biopsies** from the **contralateral** side.
- Do **not** resample the targeted biopsy side.

2B. Bilateral peripheral zone lesions example.

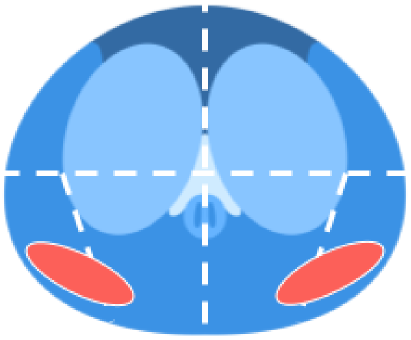

There are **two lesions**: one in right mid-gland, peripheral zone posteromedially and posterolaterally (PZ pm and PZ pl); one in left mid-gland, peripheral zone posteromedially and posterolaterally (PZ pm and PZ pl).

- Take **4 targeted biopsies** from ***each*** Target – *i.e.* **8 targeted biopsies** in **total**.
- **Do not take any systematic biopsies** as targeted biopsies are taken from both sides of the prostate.

For peer review only

2C. Lesion crossing midline example.

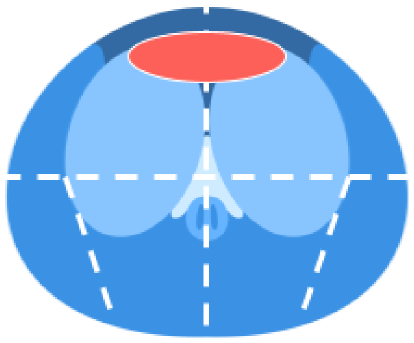

This is one lesion crossing the midline in the mid-gland, anterior fibromuscular stroma.

- Take **4 targeted biopsies** from the Target.
- **Do not take any systematic biopsies** as targeted biopsies are taken from both sides of the prostate.

2D. Bilateral diffuse change on Likert scoring example.

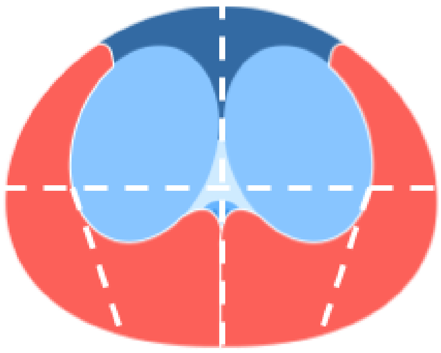

In the circumstance where on Likert scoring, the peripheral zone gives diffuse change, scoring 3 out of 5, arbitrarily **treat each peripheral zone** as a **different Target**.

- Take **4 targeted biopsies** from *each half* of the peripheral zone – *i.e.* **8 biopsies** in total.
- **Do not take any systematic biopsies** as targeted biopsies are taken from both sides of the prostate.

Designed by Aqua Asif

2E. A new lesion is revealed on DCE sequence example.

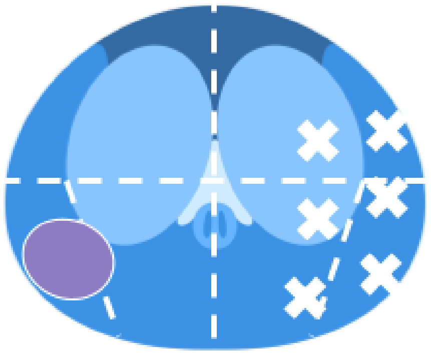

This is one lesion in the right mid-gland, peripheral zone posterolaterally. This **new Target** was specifically *not* suspicious (scored 1 or 2 on both Likert and PI-RADS v2.1) on bpMRI sequences (T2W and DWI). However, when the contrast sequence is revealed, the lesion appears to be suspicious (scored 3, 4 or 5 on Likert) on the dynamic contrast-enhanced (DCE) sequence than on the bpMRI.

- Thus, label the **new lesion** as a **DCE-Target**.
- Take **4 targeted biopsies** from **DCE-Target-1**.
- Then take **6 peripheral zone focused biopsies** from the **contralateral** side of the prostate.
- Do **not** resample the targeted biopsy side.

2F. A new **part** of an *existing* lesion is revealed on DCE sequence example.

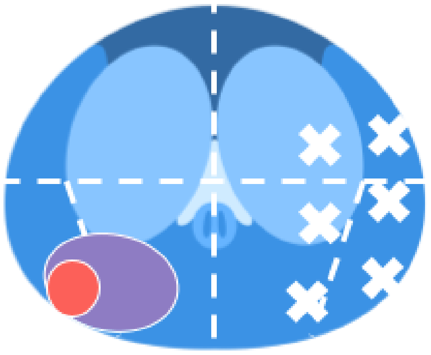

There are two lesions in this example. **Target 1** (**red**) was suspicious on **both** bpMRI and mpMRI. It is in the right mid-gland, peripheral zone, posterolaterally (PZ pl). It scores Likert 4 and PI-RADS v2.1 4.

However, when the contrast sequence is revealed, this lesion appears to be larger on the DCE sequence than on bpMRI. The part of the lesion that is **non-overlapping** would **not** have been

Designed by Aqua Asif

target biopsied if bpMRI alone was used. Thus, the second lesion (the non-overlapping part, **purple**) is called **DCE Target 1**. It is in the right mid-gland, peripheral zone, posteromedially (PZ pm).

Thus, the instructions are as follows in this instance:

- Take **4 targeted biopsies** from **Target 1**.
- Take **4 targeted biopsies** from **DCE Target 1**.
- Take **6 peripheral zone focused biopsies** from the **contralateral** side of the prostate.
- Do **not** resample the targeted biopsy side.

Systematic Transrectal Biopsy Schema

Figures 3 and **4** depict examples of how to perform the systematic biopsy in the **absence** of an MRI lesion and in the **presence** of MRI lesions, respectively.

Non-suspicious MRI but a PSA Density of ≥ 0.15ng/mL/mL scenario

In patients with a **non-suspicious MRI but a PSA Density of ≥ 0.15ng/mL/mL**, 12-core systematic biopsy should be performed (**Figure 3**).

If performing biopsies transrectally, systematic biopsy cores should be taken from:

- Right base (2 cores)
- Right mid gland (2 cores)
- Right apex (2 cores)
- Left base (2 cores)
- Left mid gland (2 cores)
- Left apex (2 cores)

Systematic biopsy cores should be stored and labelled in a way that their location can be identified when the pathologist reports the result.

The 12 systematic biopsies **should be focused on the peripheral zone.** The urethra should be avoided.

For peer review only - http://bmjopen.bmj.com/site/about/guidelines.xhtml

Figure 3. The transrectal biopsy schema for men with a **non-suspicious MRI** (scores 1 or 2 on both Likert and PI-RADS v2.1 scoring systems) *but* a PSA Density of ≥0.15ng/mL/mL, undergoing 12-core systematic biopsy.

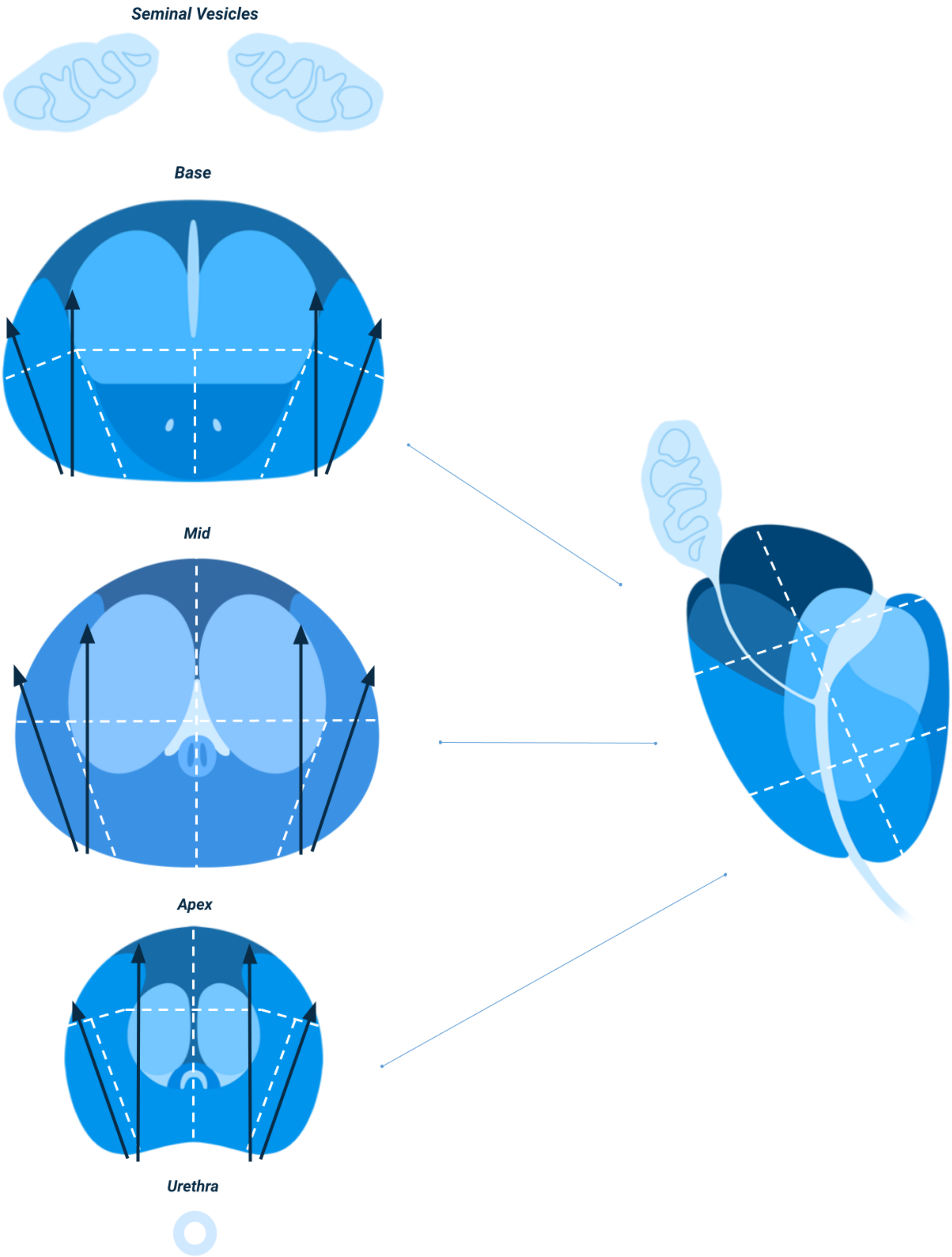

Designed by Aqua Asif

Suspicious MRI lesion scenarios

Figure 4. Examples of how to perform transrectal biopsies in patients with an MRI Target (scores 3, 4 or 5 on *either* Likert or PI-RADS v2.1 scoring systems).

4A. Single lesion example.

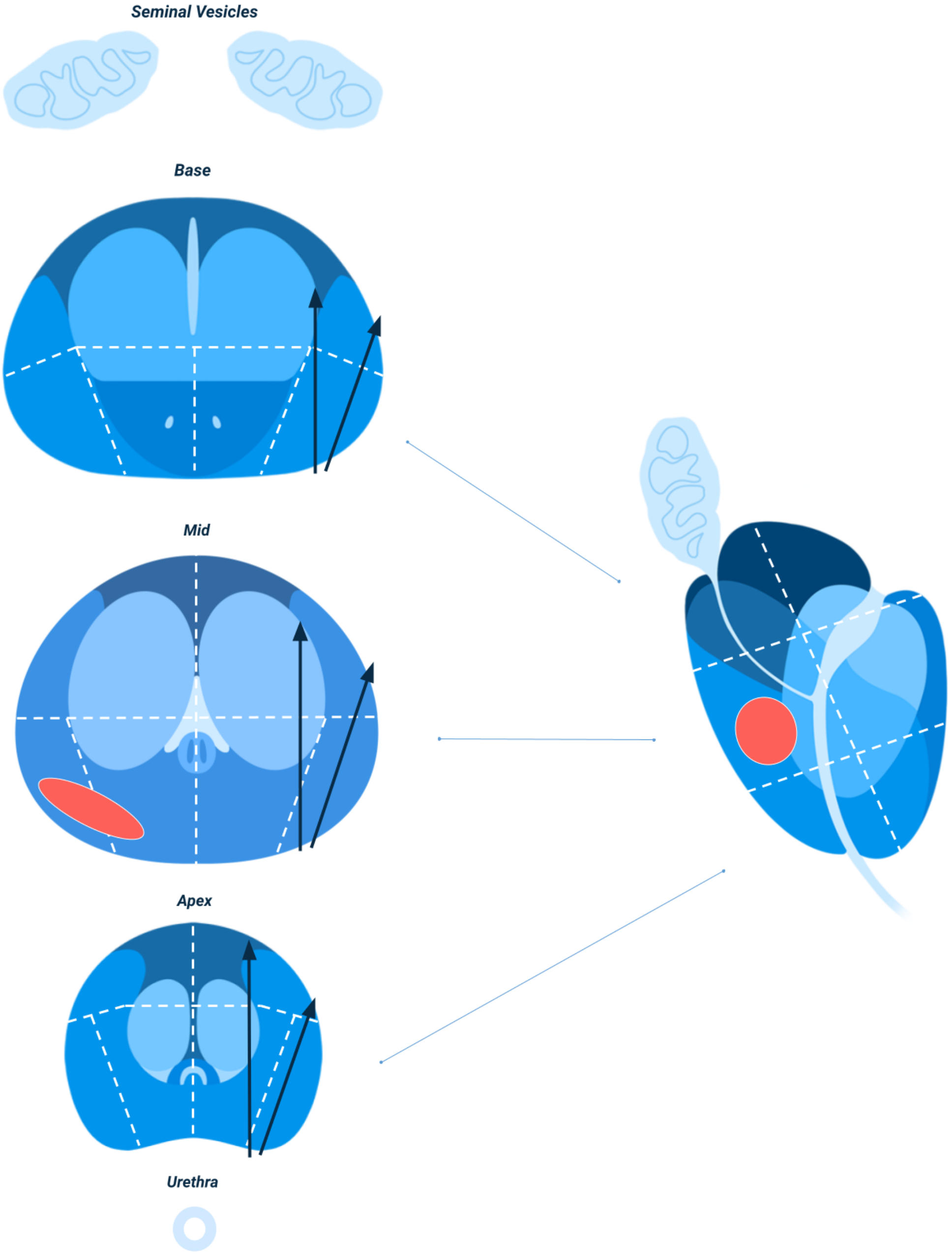

This is a single lesion in the right mid-gland peripheral zone posteromedially (PZ pm) and posterolaterally (PZ pl).

- Take **4 targeted biopsies** from the Target.
- Then take **6 peripheral zone focused biopsies** from the **contralateral** side.

- Do **not** resample the targeted biopsy side.

4B. Bilateral peripheral zone lesions example.

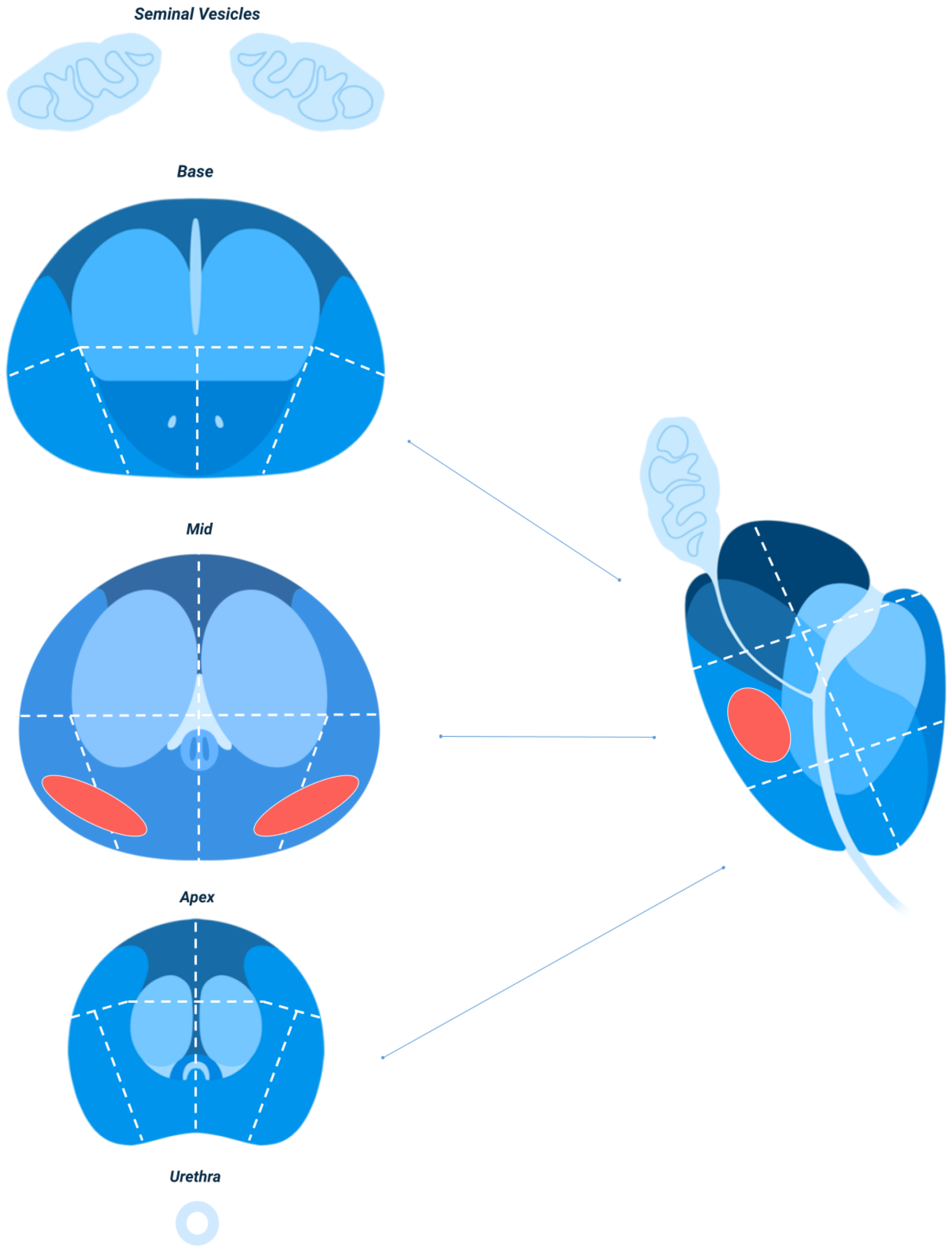

There are **two lesions**: one in right mid-gland, peripheral zone posteromedially and posterolaterally (PZ pm and PZ pl); one in left mid-gland, peripheral zone posteromedially and posterolaterally (PZ pm and PZ pl).

- Take **4 targeted biopsies** from ***each*** Target – *i.e.* **8 targeted biopsies** in **total**.

Designed by Aqua Asif

- **Do not take any systematic biopsies** as targeted biopsies are taken from both sides of the prostate.

4C. Lesion crossing midline example.

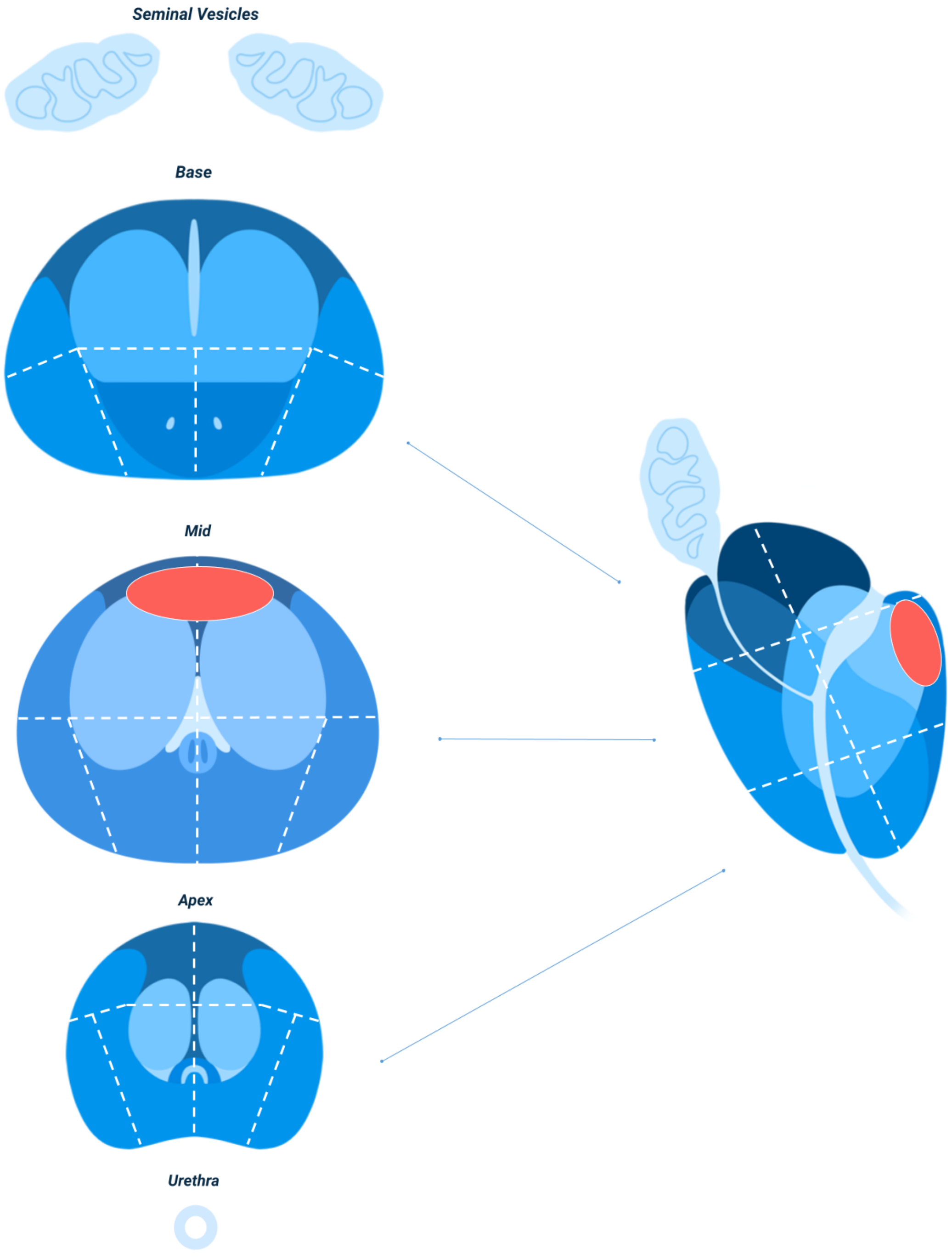

This is one lesion crossing the midline in the mid-gland, anterior fibromuscular stroma.

- Take **4 targeted biopsies** from the Target.

- **Do not take any systematic biopsies** as targeted biopsies are taken from both sides of the prostate.

4D. Bilateral diffuse change on Likert scoring example.

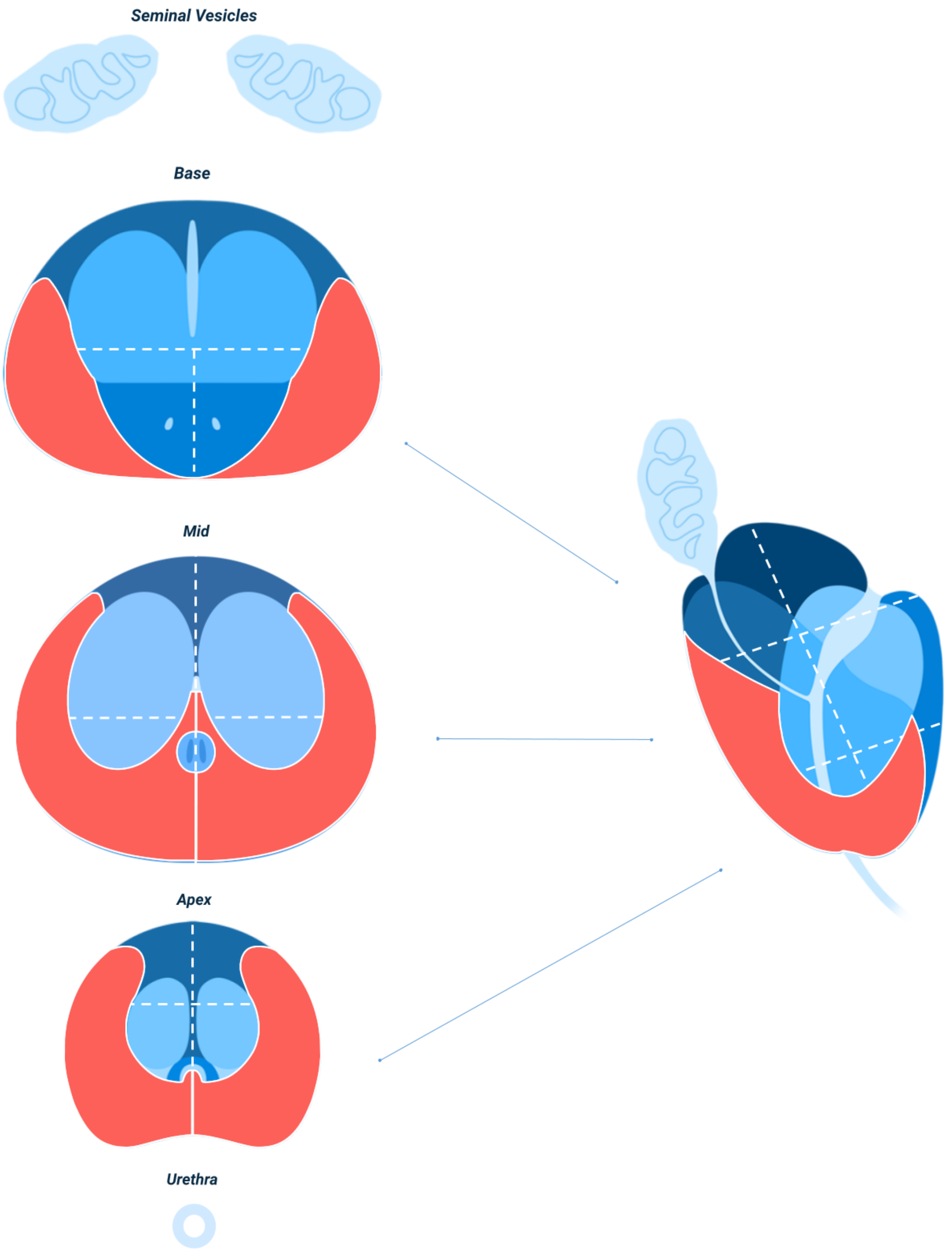

In the circumstance where on Likert scoring, the peripheral zone gives diffuse change, scoring 3 out of 5, arbitrarily **treat each peripheral zone** as a **different Target**.

Designed by Aqua Asif

- Take **4 targeted biopsies** from *each half* of the peripheral zone – *i.e.* **8 biopsies** in total.
- **Do not take any systematic biopsies** as targeted biopsies are taken from both sides of the prostate.

4E. A new lesion is revealed on DCE sequence example.

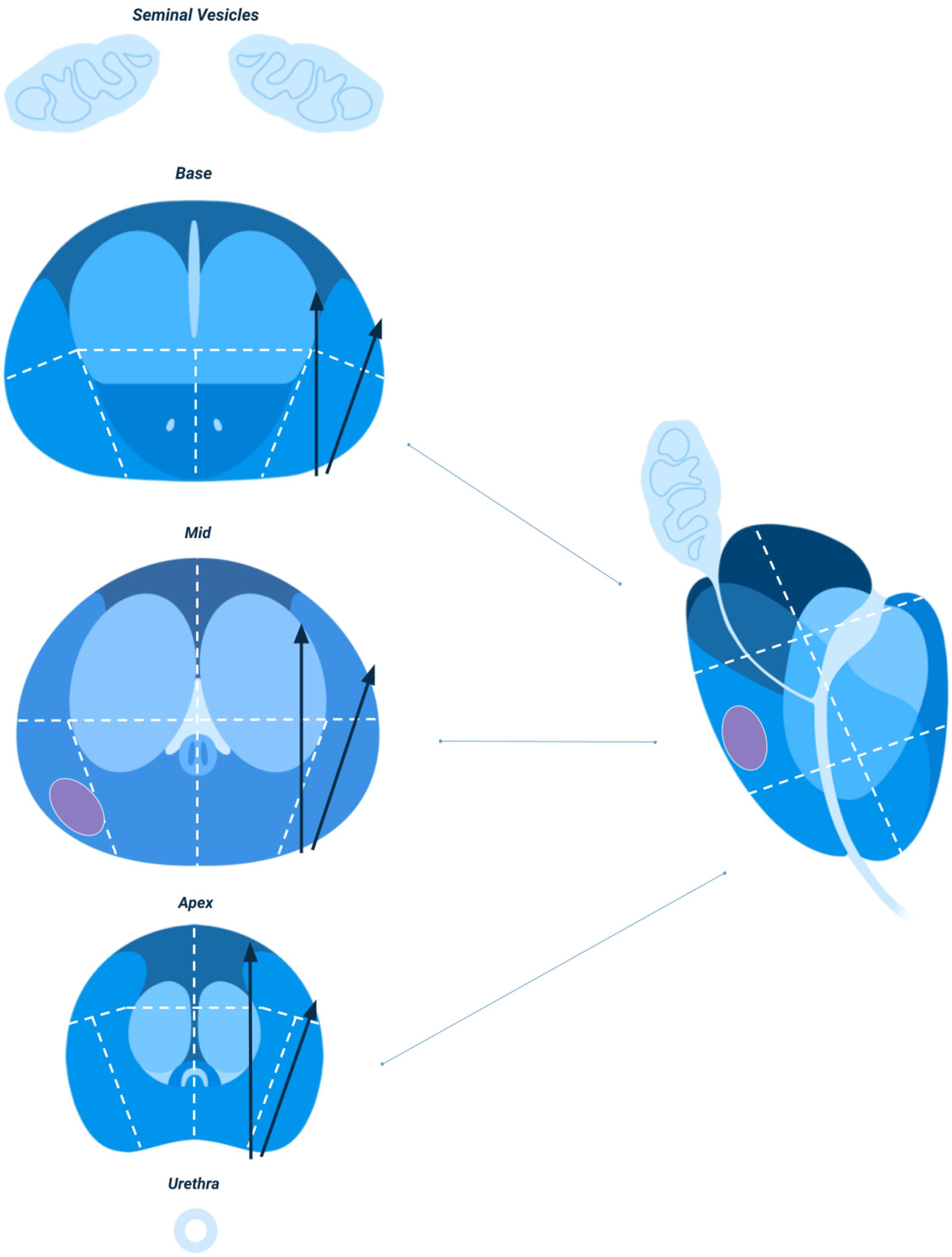

This is one lesion in the right mid-gland, peripheral zone posterolaterally. This **new Target** was specifically *not* suspicious (scored 1 or 2 on both Likert and PI-RADS v2.1) on bpMRI sequences (T2W and DWI). However, when the contrast sequence is revealed, the lesion

appears to be suspicious (scored 3, 4 or 5 on Likert) on the dynamic contrast-enhanced (DCE) sequence than on the bpMRI.

- Thus, label the **new lesion** as a **DCE-Target**.
- Take **4 targeted biopsies** from **DCE-Target-1**.
- Then take **6 peripheral zone focused biopsies** from the **contralateral** side of the prostate.
- Do **not** resample the targeted biopsy side.

4F. A new **part** of an *existing* lesion is revealed on DCE sequence example.

There are two lesions in this example. **Target 1** (**red**) was suspicious on **both** bpMRI and mpMRI. It is in the right mid-gland, peripheral zone, posterolaterally (PZ pl). It scores Likert 4 and PI-RADS v2.1 4.

However, when the contrast sequence is revealed, this lesion appears to be larger on the DCE sequence than on bpMRI. The part of the lesion that is **non-overlapping** would **not** have been target biopsied if bpMRI alone was used. Thus, the second lesion (the non-overlapping part, **purple**) is called **DCE Target 1**. It is in the right mid-gland, peripheral zone, posteromedially (PZ pm).

Thus, the instructions are as follows in this instance:

- Take **4 targeted biopsies** from **Target 1**.
- Take **4 targeted biopsies** from **DCE Target 1**.
- Take **6 peripheral zone focused biopsies** from the **contralateral** side of the prostate.
- Do **not** resample the targeted biopsy side.

For peer review only

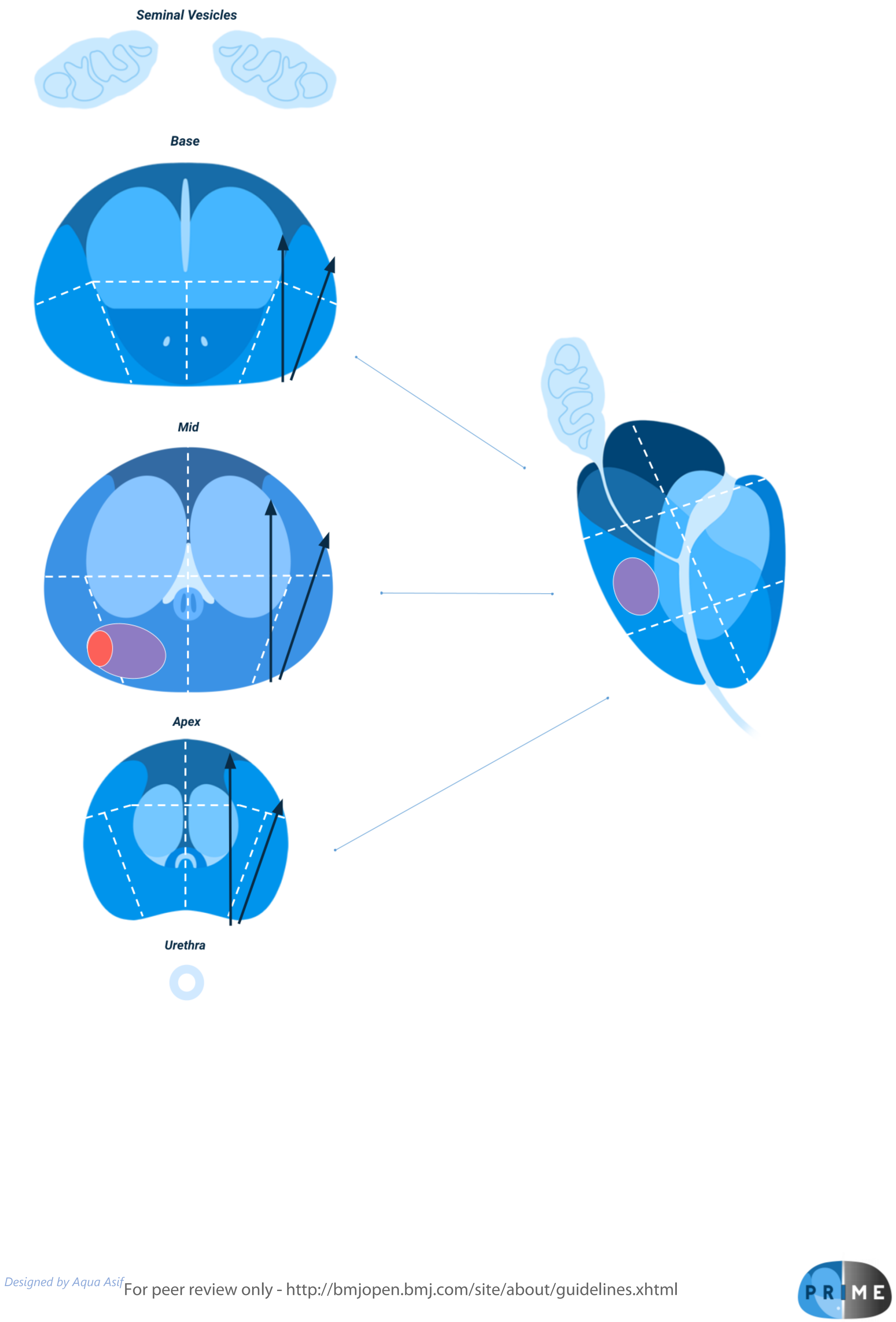
Seminal Vesicles
Base
Mid
Apex
Urethra
Designed by Aqua Asif
PRIME

Summary Biopsy Guidelines

Number of MRI targets	Location of MRI targets in prostate	Number of MRI-targeted biopsy cores	Number of contralateral systematic cores	Total number of biopsy cores
**0**	If PSA Density is < 0.15ng/ml/ml			0
**0**	If PSA Density is ≥ 0.15ng/ml/ml, then 12 systematic biopsy cores are taken (6 from each side)			12
**1**	Unilateral	4	6	10
**2**	Unilateral	8	6	14
**3**	Unilateral	12	6	18
**4–8**	Unilateral	16–32	6	22–38
**1**	Bilateral (*e.g.* crossing midline)	4	0	4
**2**	Bilateral	8	0	8
**3**	Bilateral	12	0	12
**4–8**	Bilateral	16–32	0	16–32

Designed by Aqua Asif

Please present on local headed paper

REC Number:
IRAS Number: 282789

Subject Identification: ____________________
Study Number ;__________________________

CONSENT FORM

Title of Project: PRostate **I**maging using **M**RI +/- contrast **E**nhancement (PRIME)

Name of Researcher:

Please initial box

1. I confirm that I have read and understand the information sheet dated.................... (version...........) for the above study. I have had the opportunity to consider the information, ask questions and have had these answered satisfactorily. ☐

2. I understand that my participation is voluntary and that I am free to withdraw at any time without giving any reason, without my medical care or legal rights being affected. ☐

3. I understand that relevant sections of my medical notes and data collected during the study, may be looked at by individuals from the sponsor of the trial (University College London), responsible persons authorised by the sponsor, from regulatory authorities, from the NHS Trust and from PRIME study researchers who may be outside of my local centre, where it is relevant to my taking part in this research. I give permission for these individuals to have access to my records. ☐

4. I agree to my GP being informed of my participation in the study. ☐

5. I give my permission for the PRIME research team at my local centre to hold identifiable information such as my name, address, date of birth, email address, mobile phone number, NHS number or other applicable hospital identifier. I understand this may be used to collect longer term healthcare information on me from national records, such as the Office for National Statistics, NHS Digital, Public Health England, and other applicable NHS information systems, or other relevant national databases. This data may be linked to my data from the PRIME study in future research. ☐

For peer review only - http://bmjopen.bmj.com/site/about/guidelines.xhtml

For peer review only

6. I give permission to be contacted for further information in the future. This may include requests to complete quality of life questionnaires or for ascertaining future health status, if required. ☐

7. I give permission for my samples to be sent to UCL by courier for quality control assessments. ☐

8. I give permission for my anonymized data to be used for teaching and educational purposes for healthcare professionals. ☐

9. I give my permission for my anonymized data to be shared with affiliated researchers and commercial partners who are approved by the PRIME study team for future research if deemed suitable by the PRIME Chief Investigator ☐

10. I give my permission to be approached for other studies in the future that may be relevant to me, and for my study data collected in PRIME to be used for this purpose. ☐

11. I agree to take part in the above study and to complete study procedures outlined in the patient information sheet provided. ☐

All boxes above must be initialed for consent to be valid

Name of Participant	Date	Signature
Name of Person taking consent	Date	Signature

When completed: 1 for participant; 1 (original) for researcher site file; 1 to be kept in medical notes.

For peer review only - http://bmjopen.bmj.com/site/about/guidelines.xhtml

PLACE HOSPITAL LETTER HEAD ON FIRST PAGE ONLY.

Affix patient sticker / details here

Version 3.0 8 June 2021

This is the Patient Information Sheet for a Health Research Study called PRIME

Study Short Title: Prostate Imaging using MRI +/- contrast Enhancement

Study acronym: **PRIME**

Chief Investigator: Mr Veeru Kasivisvanathan

UCL Reference number: 135819

REC Reference number: 21/WM/0091

IRAS Number: 282789

We would like to invite you to take part in our research study. Before you decide we would like you to understand why you are being invited, why the research is being done and what it would involve for you. One of our team will go through the information sheet with you and answer any questions you have. Talk to others about the study if you wish.

Part 1 tells you the purpose of this study and what will happen to you if you take part. Part 2 gives you more detailed information about the conduct of the study.

Ask us if there is anything that is not clear. Please take as much time as you need to consider the study.

For peer review only - http://bmjopen.bmj.com/site/about/guidelines.xhtml

Part 1

1. Why have I been invited?

You are being invited because you may require further investigation of your prostate with an MRI scan and / or a prostate biopsy. You have not been diagnosed with cancer but an MRI and / or a biopsy may be required to establish whether you do or do not have cancer. The clinical Urology team that you have been referred to has informed us that you may be eligible for this study.

2. What is the purpose of the study?

The standard way of diagnosing prostate cancer is to carry out a multiparametric prostate MRI scan and prostate biopsy. This type of MRI scan normally involves an injection of contrast into one of your veins.

Another type of MRI scan (biparametric) can be performed that does not require contrast, and therefore does not require the insertion of a cannula. We currently do not know for certain whether using this type of MRI will allow us to detect the same, more or less prostate cancer than if we use the standard (multiparametric) type of MRI. Current evidence supports the idea that using biparametric MRI may detect a similar amount of cancer to when it is not used but one advantage is it may allow a man to have a scan without contrast.

The main purpose of this study is to assess if biparametric MRI can provide similar information to multiparametric MRI. You will undergo a multiparametric MRI with a contrast injection, which is the typical method used for investigating the prostate for the presence of cancer. The doctor reviewing your scan will be asked to review the MRI scan in a particular order so that they can tell whether the additional information given by the contrast injection helps identifies prostate cancer.

If there is a suspicious area in the prostate on the MRI, a few biopsies can be directed at where the suspicious area is thought to be, also using an ultrasound probe in the back passage. If there is no suspicious area on the MRI and if you at low risk of harbouring cancer, which occurs in about 30% of men, then no biopsy will be taken at all.

3. Do I have to take part?

It is up to you to decide to join the study. We will describe the study and go through this information sheet. If you agree to take part, we will then ask you to sign a consent form. You are free to withdraw at any time without giving a reason. This will not affect the standard of care you receive.

4. What are the benefits to me of taking part in this study?

The healthcare team carrying out the tests in the study are experienced in carrying out and interpreting these tests. The research team will ensure your tests are carried out as quickly as possible and will be a point of contact for you should you have any concerns or questions.

The information we get from this study will help improve the diagnosis of prostate cancer for men in the future.

5. What type of study is this?

This is a study evaluating the accuracy of diagnostic tests. In this trial, you will have the same investigation (multiparametric MRI) as your hospital normally does to investigate the prostate, but the doctor interpreting your scan will be asked to report this in a particular order. The full information will be available to the doctors as it would normally be available if you were not taking part in the study.

You will be required to attend a screening visit with a member of the research team who will spend around 40 minutes explaining what is involved in the study and making sure you are

For peer review only - http://bmjopen.bmj.com/site/about/guidelines.xhtml

eligible for the study. Where possible, all study visits that do not require a journey to the hospital will be performed remotely (e.g. over the phone or video call).

6. What will happen to me if I take part?

After you have attended the screening visit, if you are eligible to take part in the study, you will asked to visit the hospital 2-3 times in total, which is the same as if you were not taking part in the study. After you consent to participating in the study, you will be asked to complete two short questionnaires which will ask about any symptoms related to your prostate that you may be having. These are questionnaires that are typically used as part of routine care. You would only undergo tests that you would normally have as part of routine care if you were not taking part in the study.

If you have not already had a prostate MRI, you will have one within a few weeks after the screening visit. The MRI takes about 40 minutes. Alternatively, it is possible that you are approached for the study after you have had your prostate MRI.

If you have an MRI with a high enough suspicion (MRI Score 3, 4 or 5) you will be booked for a biopsy following the MRI. If the MRI is non-suspicious but you are at high risk of having cancer because of a blood test result, (called your prostate specific antigen density) you will also undergo a prostate biopsy. If you do not need a biopsy (if your MRI is non-suspicious and your prostate specific antigen density is low) then you do not need to undergo a biopsy and we will explain this to you once your MRI results is available.

The biopsy procedure itself takes about 40 minutes and is typically carried out under local or general anaesthetic. Prostate biopsies, which take very small samples of prostate tissue, are taken from the prostate gland and sent to the lab to determine whether there is cancer there or not. If there is a suspicious area on the MRI scan, the MRI information will be used to influence where the biopsies are taken from. Software may be used to transfer additional information from the original MRI onto the screen when the biopsies are taken. In some centres, this would be exactly what you would normally get, and there would be no difference to standard of care. In other centres, their usual practice may be slightly different to this, and you may be required to have a few extra or fewer biopsies than what is typical in your usual centre. After the procedure, we then wait for the results and discuss treatment options with you in clinic at approximately 2-3 weeks after the biopsies.

Please note that the above time frames are suggested time frames and depending on clinical workload within the hospital, the time frame may be shorter or longer. This would be no different than if you were not part of the study.

Being involved in the study does not limit subsequent tests or treatment you may receive. If you do undergo further tests or treatment after the study is complete we may check the results of these on your records. We use the research data we have gathered from your involvement in the study to help us determine how good the diagnostic tests you have had are. We will work with other research teams to do this. We also ask your permission to use research data for teaching and education of other healthcare professionals. After completing the study, we also ask your permission to check your health through national databases. We may also contact you for further information in the future. This may include requests to complete quality of life questionnaires or for ascertaining future health status. All information which is collected about you during the course of the research will be kept strictly confidential, and any information about you which leaves the hospital will have your name and address removed so that you cannot be recognised. Please see Part 2 for further information on this.

7. What data will be collected and use of data

We will need to use information from your medical records for this research project. Your hospital will hold personal identifiable data on you. This information will include information such as age, PSA level, family history of medical conditions such as prostate cancer and examination findings. We allow the PRIME research team at your local site to hold

For peer review only - http://bmjopen.bmj.com/site/about/guidelines.xhtml

identifiable data on you, which will be for 10 years. Longer term data that may be requested from you include information on whether or not you have had further investigations or treatment for prostate problems and what the outcomes of those were as well as quality of life assessments. Non-identifiable data will be stored in the MARVIN database and the database will be transferred and stored at UCL within UCL's data safe haven. You will be given a subject number and a subject identifier, and this will be used on all your study records. The code for this number will be known to the investigators at your site so that the link between your name and the data we hold on the study database is not completely broken. Any paperwork for the study will be kept in locked cupboards, staff access to these cupboards is strictly controlled.

In general, UCL, as a university and a study sponsor, uses personally-identifiable information to conduct research to improve health, care and services. As a publicly-funded organisation, we have to ensure that it is in the public interest when we use personally-identifiable information from people who have agreed to take part in research. This means that when you agree to take part in a research study, we will use your data in the ways needed to conduct and analyse the research study. Your rights to access, change or move your information are limited, as we need to manage your information in specific ways in order for the research to be reliable and accurate. If you withdraw from the study, we will keep the information about you that we have already obtained. To safeguard your rights, we will use the minimum personally-identifiable information possible.

Health and care research should serve the public interest, which means that we have to demonstrate that our research serves the interests of society as a whole. We do this by following the UK Policy Framework for Health and Social Care Research.

All data is managed in line with the Data Protection Act (2018) & General Data Protection Regulations (GDPR).

If you wish to raise a complaint on how we have handled your personal data, you can contact our Data Protection Officer who will investigate the matter. If you are not satisfied with our response or believe we are processing your personal data in a way that is not lawful you can complain to the Information Commissioner's Office (ICO).

UCL Data Protection Officer can be contacted on data-protection@ucl.ac.uk

8. What will I have to do?

You should attend your screening visit and if eligible for the study, await contact from the hospital for further dates of investigations. Unless otherwise advised by a doctor you should carry on with your normal activities and medication. Sometimes before a biopsy your doctor will prescribe you antibiotics and may ask you to stop blood-thinning medications.

You should undergo the necessary tests and biopsy procedures that you are advised to have by your doctor.

You should attend your follow up clinic appointment where we discuss your results. Treatment options will be discussed with you at the results clinic. In total you will typically be required to attend the hospital 2-3 times.

9. What are the alternatives for diagnosis?

An MRI scan and biopsies of the prostate if required are the standard ways in which prostate cancer is diagnosed.

10. What are the possible disadvantages and risks of taking part?

For peer review only - http://bmjopen.bmj.com/site/about/guidelines.xhtml

Being involved in the study is unlikely to expose you to additional risk than if you were not involved in the study but underwent the normal procedures for men referred for further investigation of prostate disease.

Risks of prostate biopsy include:

- Temporary discomfort in the back passage (most men)
- Blood in the urine – up to 2 weeks (most men)
- Blood in the semen – up to 3 months (most men)
- Blood in the back passage – up to 1 week (most men)
- Infection in the blood stream – 1-4 out of 100 men
- Urinary tract infection – 4 out of 100 men
- Urinary retention – 1 out of 100 men
- Adverse reaction to antibiotics – less than 1 in 100 men

Risks of MRI include:

- Discomfort from cannulation
- Allergic reaction:
  - Mild reaction e.g. rash, itching – less than 1 in 250 men
  - Moderate reaction e.g. nausea, omitting – less than 1 in 2000 men
  - Severe reaction e.g. breathing problems – less than 1 in 10000 men

In some centres, you would receive exactly what you would normally get outside of the study. In other centres, their usual practice may be slightly different to this, and you may be required to have a few extra or fewer biopsies than what is typical in your usual centre. However, there is no evidence that a few extra or fewer biopsies within the proposed study would result in additional adverse effects for you.

Before participating you should consider if this will affect any insurance you have and seek advice if necessary.

11. What should you do if you experience any problems during the study?
Though the risk is very low, if you do experience any possible signs of infection after biopsies (fevers and feeling generally unwell) then you should urgently go to your nearest accident and emergency department which is open 24 hours a day. If you are not able to pass urine you should urgently go to your nearest accident and emergency. If you are unsure about what to do or have any questions please call 0207 679 9092 between 9am and 5pm and a member of our team may be able to offer you advice or direct you to someone who can offer you advice.

If you experience any other untoward complication or need to see a doctor we would like to know about this so please let us know on the above number as soon as possible after the complication. For any emergencies at any time or if you are unable to contact a member of the research team, please attend your local accident and emergency for an assessment.

12. What happens when the research study stops?
Once the results of the MRI and, if required, biopsy are available you will be called to clinic to discuss them. Once a treatment decision is made, most men in the study will complete the study and your normal clinical team will continue to look after your care. Being part of the study does not prevent you from undergoing any further diagnostic test or treatment that your clinician would normally recommend.

13. What if there is a problem?
Any complaint about the way you have been dealt with during the clinical study or any possible harm you might suffer will be addressed. The detailed information concerning this is given in Part 2 of this information sheet. If you have any concerns or complaints you should contact a member of the research team in the first instance.

For peer review only - http://bmjopen.bmj.com/site/about/guidelines.xhtml

14. Will my taking part in the study be kept confidential?

Yes. We will follow ethical and legal practice and all information about you will be handled in confidence. The details are included in Part 2.

15. Will any costs I incur in travelling to study visits be reimbursed to me?

Reasonable transport costs that you incur to get to additional study visits (if any further visits are necessary) that are above what you would normally need if you were not part of the study may be reimbursed. Please contact your local study nurse or doctor or the Study Coordinator (details below) for further information on claiming.

16. Contact Details

If you have any further questions or need any further information please do no hesitate to contact the research team.

or the **Chief Investigator:**

Mr Veeru Kasivisvanathan MBBS BSc FRCS MSc PGCert PhD
Division of Surgery and Interventional Science, University College London
3rd floor Charles Bell House, 43-45 Foley Street
London W1W 7TS
T: 0207 679 9092 F: 0207 679 9511 E: veeru.kasi@ucl.ac.uk

This completes Part 1 of the Information Sheet.

If the information in Part 1 has interested you and you are considering participation, please read the additional information in Part 2 before making any decision.

For peer review only

For peer review only - http://bmjopen.bmj.com/site/about/guidelines.xhtml

Part 2

17. What if relevant new information becomes available?

Sometimes we get new information about the procedures being studied. If this happens and we feel it is important to your participation in the study, we will tell you about it and discuss whether you want to or should continue in the study. If you decide not to carry on, we will make arrangements for your care to continue. If you decide to continue in the study, we may ask you to sign an updated consent form. You can also find out if there is any new relevant information by visiting www.ncita.org.uk.

18. What will happen if I don't want to carry on with the study?

You can withdraw from the study at any point and it will not affect the care that you are given. We will use information collected about you up until your withdrawal. Kindly keep in contact with us to let us know your progress.

19. What if there is a problem?

If you have a concern about any aspect of this study, you should ask to speak to your research team who will do their best to answer your questions, please see point number 24. You can also contact the Chief Investigators on the number or address given earlier in this document. If you wish to complain by other means or have any concerns about any aspect of the way you have been approached or treated by members of staff or about any side effects (adverse events) you may have experienced due to your participation in the clinical study, the normal National Health Service complaints mechanisms are available to you. You can contact the hospital Patient Advice and Liaison Service (PALS). Your local PALS team can be contacted at the following number:

Local team to insert contact details of local PALS office here:

You can also contact NHS helpline at 111 which will be able to give you the number of your local PALS office if you are concerned.

Every care will be taken in the course of this clinical study. However in the unlikely event that you are injured by taking part, compensation may be available.

If you suspect that the injury is the result of the Sponsor's (University College London) or the hospital's negligence, then you may be able to claim compensation. After discussing with your study doctor, please make the claim in writing to Mr Veeru Kasivisvanathan who is the Chief Investigator for the clinical study and is based at University College London. The Chief Investigator will then pass the claim to the Sponsor's Insurers, via the Sponsor's office. You may have to bear the costs of the legal action initially, and you should consult a lawyer about this.

20. Will my taking part in this study be kept confidential?

If you consent to take part in this study, the records obtained while you are in this study as well as related health records will remain strictly confidential at all times. The information will be held securely on paper and electronically at your treating hospital under the provisions of the Data Protection Act 2018 and the General Data Protection Regulations 2018. The information will be made available to persons in the clinical and research teams treating you. Your name and personal details will not be passed to anyone else outside the clinical team, research team or the Sponsor, who is not involved in the study. No additional samples will be taken specially for research in this study. All The research team may verify results of tests carried out at your local hospital (for example MRI results or prostate biopsy results) by transferring and analysing a small number of samples collected to UCL. samples and information collected will be de-identified to you prior to transfer to UCL, so only non-identifiable data will be transferred to UCL. This includes some pathology glass slides, which will be reviewed at Dr Alex Freeman's laboratory at University College London (UCL), for quality control. Slides sent to UCL will be

For peer review only - http://bmjopen.bmj.com/site/about/guidelines.xhtml

not have your name assigned. Samples will be sent using one of UCL's preferred couriers, for both pick up and return.

Any data stored by the research team outside of your treating hospital will be kept at a secure location and will not contain information that can directly identify you. You will be allocated a study number, which will be used as a code to identify you on all study forms and data. The information will be linked to you so that if we did need to identify you for your safety or to clarify some information we would be able to by using a unique key, which will be known only to your local hospital team.

Your records will be available to people authorised to work on the study but may also need to be made available to people authorised by the Sponsor, which is the organisation responsible for ensuring that the study is carried out correctly. By signing the consent form, you agree to this access for the current study and any further research that may be conducted in relation to it, even if you withdraw from the current study. All will have a duty of confidentiality to you as a research participant.

If you withdraw consent from further study treatment, your data and samples will remain on file and will be included in the final study analysis.

In line with the regulations, at the end of the study your data will be securely archived for 20 years. Arrangements for confidential destruction will then be made.

Anonymised data collected during the study may be transferred for the purpose of processing or analysis to approved associated researchers and commercial partners within/outside the European Economic Area. The Sponsor of the study will take all reasonable steps to protect your privacy.

In the future we may publish our findings from the study in scientific journals, but you will not be identifiable in any publications.

21. Will my GP be informed of my involvement?
Because this study is not being carried out by your GP, we would like to inform them of your participation. If you agree to take part and agree to us contacting your GP, we will give him or her details of the study and inform them that you have chosen to participate in it. You will not be able to participate in this study if you do not give us this permission to inform your GP.

22. What will happen to the results of the research study?
The results of the study will be available after it finishes and will usually be published online in a medical journal and presented at a scientific conference, they will also be posted to. The data will be anonymous and it will not be possible to identify you in any report or publication. Sometimes the data may be used to teach other healthcare professionals how to treat patients in a similar position to you.

Should you wish to see the results, or the publication, please ask your study doctor or see the trial website on https://www.ucl.ac.uk/surgery/research/research-department-targeted-intervention/prime-trial-information, or the clinical trials units website www.ncita.org.uk.

23. Who is organising and funding the research?
The governance sponsor is University College London. The study is funded by Prostate Cancer UK, the European Association of Urology Research Foundation, the UK National Institute for Health Research via an Academic Clinical Lectureship to Dr Veeru Kasivisvanathan and the UK National Cancer Imaging Translational Accelerator.

24. Who has reviewed the study?

For peer review only - http://bmjopen.bmj.com/site/about/guidelines.xhtml

All research in the NHS is looked at by an independent group of people, called a Research Ethics Committee, to protect your interests. This study has been reviewed and given favorable opinion by National Research Ethics Service Committee _West Midlands - Black Country Research Ethics Committee. Patients and members of the public have also reviewed the study documents to ensure they are appropriate and well written.

25. Further information

You are encouraged to ask any questions you wish, before, during or after your investigations. If you have any questions about the study, please speak to your study nurse or doctor on the numbers specified below, who will be able to provide you with up to date information about the procedures involved. If you wish to read the research on which this study is based, please ask your study nurse or doctor.

Site Study staff contact details:

Principal Investigator (site) details:

Alternatively, if you or your relatives have any questions about this study you may wish to contact one of the following organisations that are independent of the hospital at which you are being treated:

Prostate Cancer UK – 0800 082 1616 - http://prostatecanceruk.org

Macmillan Cancer Support - 0808 808 0000 – http://www.macmillan.org.uk

If you decide you would like to take part then please read and sign the consent form. You will be given a copy of this information sheet and the consent form to keep. A copy of the consent form will be filed in your patient notes, one will be filed with the study records and one may be sent to the Research Sponsor.

You can have more time to think this over if you are at all unsure.

Thank you for taking the time to read this information sheet and to consider this study.

For peer review only

For peer review only - http://bmjopen.bmj.com/site/about/guidelines.xhtml

Reporting checklist for protocol of a clinical trial.

Based on the SPIRIT guidelines.

Instructions to authors

Complete this checklist by entering the page numbers from your manuscript where readers will find each of the items listed below.

Your article may not currently address all the items on the checklist. Please modify your text to include the missing information. If you are certain that an item does not apply, please write "n/a" and provide a short explanation.

Upload your completed checklist as an extra file when you submit to a journal.

In your methods section, say that you used the SPIRITreporting guidelines, and cite them as:

Chan A-W, Tetzlaff JM, Gøtzsche PC, Altman DG, Mann H, Berlin J, Dickersin K, Hróbjartsson A, Schulz KF, Parulekar WR, Krleža-Jerić K, Laupacis A, Moher D. SPIRIT 2013 Explanation and Elaboration: Guidance for protocols of clinical trials. BMJ. 2013;346:e7586

		Reporting Item	Page Number
**Administrative information**			
Title	#1	Descriptive title identifying the study design, population, interventions, and, if applicable, trial acronym	#1
Trial registration	#2a	Trial identifier and registry name. If not yet	#3

For peer review only

		registered, name of intended registry	
Trial registration: data set	#2b	All items from the World Health Organization Trial Registration Data Set	Table 3
Protocol version	#3	Date and version identifier	Table 4
Funding	#4	Sources and types of financial, material, and other support	#13
Roles and responsibilities: contributorship	#5a	Names, affiliations, and roles of protocol contributors	#13
Roles and responsibilities: sponsor contact information	#5b	Name and contact information for the trial sponsor	Table 5
Roles and responsibilities: sponsor and funder	#5c	Role of study sponsor and funders, if any, in study design; collection, management, analysis, and interpretation of data; writing of the report; and the decision to submit the report for publication, including whether they will have ultimate authority over any of these activities	Table 5
Roles and responsibilities: committees	#5d	Composition, roles, and responsibilities of the coordinating centre, steering committee, endpoint adjudication committee, data management team, and other individuals or groups overseeing the trial,	Table 5

For peer review only

		if applicable (see Item 21a for data monitoring committee)	
**Introduction**			
Background and rationale	#6a	Description of research question and justification for undertaking the trial, including summary of relevant studies (published and unpublished) examining benefits and harms for each intervention	#4
Background and rationale: choice of comparators	#6b	Explanation for choice of comparators	#5
Objectives	#7	Specific objectives or hypotheses	#4
Trial design	#8	Description of trial design including type of trial (eg, parallel group, crossover, factorial, single group), allocation ratio, and framework (eg, superiority, equivalence, non-inferiority, exploratory)	#5
**Methods: Participants, interventions, and outcomes**			
Study setting	#9	Description of study settings (eg, community clinic, academic hospital) and list of countries where data will be collected. Reference to where list of study sites can be obtained	#5

For peer review only

Eligibility criteria	#10	Inclusion and exclusion criteria for participants. If applicable, eligibility criteria for study centres and individuals who will perform the interventions (eg, surgeons, psychotherapists)	#5
Interventions: description	#11a	Interventions for each group with sufficient detail to allow replication, including how and when they will be administered	#5
Interventions: modifications	#11b	Criteria for discontinuing or modifying allocated interventions for a given trial participant (eg, drug dose change in response to harms, participant request, or improving / worsening disease)	#11
Interventions: adherance	#11c	Strategies to improve adherence to intervention protocols, and any procedures for monitoring adherence (eg, drug tablet return; laboratory tests)	#9
Interventions: concomitant care	#11d	Relevant concomitant care and interventions that are permitted or prohibited during the trial	#9
Outcomes	#12	Primary, secondary, and other outcomes, including the specific measurement variable (eg, systolic blood pressure), analysis metric (eg, change from baseline, final value, time to event), method of aggregation (eg, median, proportion), and time point for each outcome. Explanation of the clinical relevance of chosen efficacy and harm outcomes is strongly recommended	#9

For peer review only

Participant timeline	#13	Time schedule of enrolment, interventions (including any run-ins and washouts), assessments, and visits for participants. A schematic diagram is highly recommended (see Figure)	Table 2 and Figure 1
Sample size	#14	Estimated number of participants needed to achieve study objectives and how it was determined, including clinical and statistical assumptions supporting any sample size calculations	#9
Recruitment	#15	Strategies for achieving adequate participant enrolment to reach target sample size	#9
**Methods: Assignment of interventions (for controlled trials)**			
Allocation: sequence generation	#16a	Method of generating the allocation sequence (eg, computer-generated random numbers), and list of any factors for stratification. To reduce predictability of a random sequence, details of any planned restriction (eg, blocking) should be provided in a separate document that is unavailable to those who enrol participants or assign interventions	N/A
Allocation concealment mechanism	#16b	Mechanism of implementing the allocation sequence (eg, central telephone; sequentially numbered, opaque, sealed envelopes), describing	N/A

For peer review only

		any steps to conceal the sequence until interventions are assigned	
Allocation: implementation	#16c	Who will generate the allocation sequence, who will enrol participants, and who will assign participants to interventions	N/A
Blinding (masking)	#17a	Who will be blinded after assignment to interventions (eg, trial participants, care providers, outcome assessors, data analysts), and how	N/A
Blinding (masking): emergency unblinding	#17b	If blinded, circumstances under which unblinding is permissible, and procedure for revealing a participant's allocated intervention during the trial	N/A
**Methods: Data collection, management, and analysis**			
Data collection plan	#18a	Plans for assessment and collection of outcome, baseline, and other trial data, including any related processes to promote data quality (eg, duplicate measurements, training of assessors) and a description of study instruments (eg, questionnaires, laboratory tests) along with their reliability and validity, if known. Reference to where data collection forms can be found, if not in the protocol	#9 and Supplementary Appendix 1

For peer review only

Data collection plan: retention	#18b	Plans to promote participant retention and complete follow-up, including list of any outcome data to be collected for participants who discontinue or deviate from intervention protocols	#9
Data management	#19	Plans for data entry, coding, security, and storage, including any related processes to promote data quality (eg, double data entry; range checks for data values). Reference to where details of data management procedures can be found, if not in the protocol	#9
Statistics: outcomes	#20a	Statistical methods for analysing primary and secondary outcomes. Reference to where other details of the statistical analysis plan can be found, if not in the protocol	#10
Statistics: additional analyses	#20b	Methods for any additional analyses (eg, subgroup and adjusted analyses)	#10
Statistics: analysis population and missing data	#20c	Definition of analysis population relating to protocol non-adherence (eg, as randomised analysis), and any statistical methods to handle missing data (eg, multiple imputation)	#10

Methods: Monitoring

Data monitoring: formal committee	#21a	Composition of data monitoring committee (DMC); summary of its role and reporting structure;	#11

For peer review only

		statement of whether it is independent from the sponsor and competing interests; and reference to where further details about its charter can be found, if not in the protocol. Alternatively, an explanation of why a DMC is not needed	
Data monitoring: interim analysis	#21b	Description of any interim analyses and stopping guidelines, including who will have access to these interim results and make the final decision to terminate the trial	#11
Harms	#22	Plans for collecting, assessing, reporting, and managing solicited and spontaneously reported adverse events and other unintended effects of trial interventions or trial conduct	#11
Auditing	#23	Frequency and procedures for auditing trial conduct, if any, and whether the process will be independent from investigators and the sponsor	Table 5
**Ethics and dissemination**			
Research ethics approval	#24	Plans for seeking research ethics committee / institutional review board (REC / IRB) approval	#11
Protocol amendments	#25	Plans for communicating important protocol modifications (eg, changes to eligibility criteria, outcomes, analyses) to relevant parties (eg, investigators, REC / IRBs, trial participants, trial	Table 4

For peer review only

		registries, journals, regulators)	
Consent or assent	#26a	Who will obtain informed consent or assent from potential trial participants or authorised surrogates, and how (see Item 32)	#12
Consent or assent: ancillary studies	#26b	Additional consent provisions for collection and use of participant data and biological specimens in ancillary studies, if applicable	Supplementary Appendix 3
Confidentiality	#27	How personal information about potential and enrolled participants will be collected, shared, and maintained in order to protect confidentiality before, during, and after the trial	#12
Declaration of interests	#28	Financial and other competing interests for principal investigators for the overall trial and each study site	#12
Data access	#29	Statement of who will have access to the final trial dataset, and disclosure of contractual agreements that limit such access for investigators	#12
Ancillary and post trial care	#30	Provisions, if any, for ancillary and post-trial care, and for compensation to those who suffer harm from trial participation	#11
Dissemination policy: trial results	#31a	Plans for investigators and sponsor to communicate trial results to participants, healthcare professionals, the public, and other relevant groups (eg, via publication, reporting in results databases, or other	#12

For peer review only

		data sharing arrangements), including any publication restrictions	
Dissemination policy: authorship	#31b	Authorship eligibility guidelines and any intended use of professional writers	#12
Dissemination policy: reproducible research	#31c	Plans, if any, for granting public access to the full protocol, participant-level dataset, and statistical code	#12

Appendices

Informed consent materials	#32	Model consent form and other related documentation given to participants and authorised surrogates	Supplementary Appendix 3
Biological specimens	#33	Plans for collection, laboratory evaluation, and storage of biological specimens for genetic or molecular analysis in the current trial and for future use in ancillary studies, if applicable	Supplementary Appendix 1 and 2

Notes:

- 18a: #11 and Supplementary Appendix 1
- 26b: Supplementary Appendix 3
- 32: Supplementary Appendix 3
- 33: Supplementary Appendix 1 and 2
- The SPIRIT Explanation and Elaboration paper is distributed under the terms of the Creative Commons Attribution License CC-BY-NC. This checklist was completed on 16. November 2022

For peer review only

using https://www.goodreports.org/, a tool made by the EQUATOR Network in collaboration with Penelope.ai

For peer review only
