# Supplementary material for: Comparing biparametric to multiparametric MRI in the diagnosis of clinically significant prostate cancer in biopsy-naive men (PRIME): a prospective, international, multicentre, non-inferiority within-patient, diagnostic yield trial protocol
Source: BMJ Open. 2023 Apr 5;13(4):e070280. doi: 10.1136/bmjopen-2022-070280 (PMC10083803; doi:10.1136/bmjopen-2022-070280)
Supplement: Reviewer comments [file bmjopen-2022-070280.reviewer_comments.pdf]

PEER REVIEW HISTORY

BMJ Open publishes all reviews undertaken for accepted manuscripts. Reviewers are asked to complete a checklist review form (**http://bmjopen.bmj.com/site/about/resources/checklist.pdf**) and are provided with free text boxes to elaborate on their assessment. These free text comments are reproduced below.

ARTICLE DETAILS

**TITLE (PROVISIONAL)**	Comparing biparametric to multiparametric MRI in the diagnosis of clinically significant prostate cancer in biopsy-naive men (PRIME): a prospective, international, multicentre, non-inferiority, within-patient, diagnostic yield trial protocol
**AUTHORS**	Asif, Aqua; Nathan, Arjun; Ng, Alexander; Khetrapal, Pramit; Chan, Vinson Wai-Shun; Giganti, Francesco; Allen, Clare; Freeman, Alex; Punwani, Shonit; Lorgelly, Paula; Clarke, Caroline; Brew-Graves, Chris; Muirhead, Nicola; Emberton, Mark; Agarwal, Ridhi; Takwoingi, Yemisi; Deeks, Jonathan; Moore, Caroline; Kasivisvanathan, Veeru; Trial Group, PRIME

This article was not externally reviewed at BMJ Open. Protocols that have been independently assessed prior to submission to BMJ Open are usually fast-tracked to publication on the grounds that further substantial changes will not be possible. This independent assessment will usually be external, independent review for both a substantial grant award from a non-commercial or government funder and ethics approval.
